# Deletions across the SARS-CoV-2 Genome: Molecular Mechanisms and Putative Functional Consequences of Deletions in Accessory Genes

**DOI:** 10.3390/microorganisms11010229

**Published:** 2023-01-16

**Authors:** Igor B. Rogozin, Andreu Saura, Anastassia Bykova, Vyacheslav Brover, Vyacheslav Yurchenko

**Affiliations:** 1 National Center for Biotechnology Information, National Library of Medicine, National Institutes of Health, Bethesda, MD 20894, USA; 2 Life Science Research Centre, Faculty of Science, University of Ostrava, 710 00 Ostrava, Czech Republic

**Keywords:** replication, template switch, recurrent deletions, evolution, palindromes, recombination

## Abstract

The analysis of deletions may reveal evolutionary trends and provide new insight into the surprising variability and rapidly spreading capability that SARS-CoV-2 has shown since its emergence. To understand the factors governing genomic stability, it is important to define the molecular mechanisms of deletions in the viral genome. In this work, we performed a statistical analysis of deletions. Specifically, we analyzed correlations between deletions in the SARS-CoV-2 genome and repetitive elements and documented a significant association of deletions with runs of identical (poly-) nucleotides and direct repeats. Our analyses of deletions in the accessory genes of SARS-CoV-2 suggested that there may be a hypervariability in *ORF7A* and *ORF8* that is not associated with repetitive elements. Such recurrent search in a “sequence space” of accessory genes (that might be driven by natural selection) did not yet cause increased viability of the SARS-CoV-2 variants. However, deletions in the accessory genes may ultimately produce new variants that are more successful compared to the viral strains with the conventional architecture of the SARS-CoV-2 accessory genes.

## 1. Introduction

**Figure 1:**
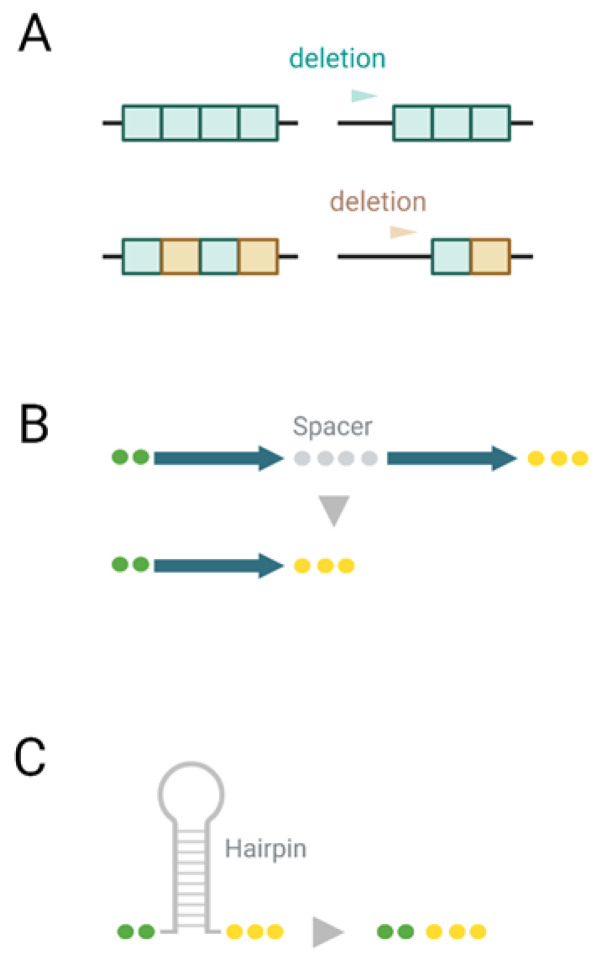
Mechanisms of deletions in DNA. (**A**) Template dislocation model: one (or several) nucleotide deletions in short stretches of identical (poly-)nucleotides. (**B**) Template switch model: deletion between direct repeats that includes the removal of one repeat; (**C**) deletion of hairpin structures.

Repeated DNA sequences are prone to various DNA rearrangements at relatively high frequencies [1,2,3]. Deletions between repeated sequences in the bacterium *Escherichia coli* have been studied systematically and have provided evidence that sufficiently-long homologous sequences (over 200 bp) rearrange, in part, via a RecA-dependent homologous recombination [4]. However, rearrangements can also efficiently occur by a RecA-independent “non-recombinational” mechanism, which involves short stretches of identical (poly-) nucleotides, direct repeats, and hairpin structures (Figure 1). Removal of one or both copies of repeated sequences is the result of so-called illegitimate recombination [1,5]. These rearrangements are dependent upon the close proximity of the repeated sequences [6,7] and can occur between repeats ranging from several to hundreds of nucleotides in length [8,9]. It has been proposed that these non-recombinational rearrangements may occur by a template dislocation (Figure 1A) or a template switch misalignment (Figure 1B) of the repeated sequences during DNA replication. The replication slipped misalignment models (Figure 1A,B) nicely account for the proximity dependence and RecA independence of these events [10,11,12]. A replication mechanism for RecA-independent rearrangements is supported by experimental evidence [13,14]. Furthermore, mutations in many replication components of *E. coli* stimulate such rearrangements [15,16].

The importance of both deletions and duplications of genomic DNA at repeated sequences is widely accepted, because these events (for example, deletions/duplications of trinucleotide repeat arrays) are responsible for several human diseases [17,18,19,20]. 

SARS-CoV-2 has accumulated many variations since its emergence in late 2019 [21]. Nucleotide substitutions that produce amino acid replacements constitute the primary raw material for genetic variation; however, many insertions and deletions (indels) are likely to be critical elements in coronavirus macro- and microevolution [22,23,24,25,26,27]. Although most indels negatively affect viral fitness, a small number of them emerged and spread in viral populations, suggesting a positive effect on viral fitness and adaptive evolution [28].

The analysis of deletions may reveal evolutionary trends and provide new insights into the surprising variability and rapid spreading capability that SARS-CoV-2 has demonstrated since its emergence. Recent evidence established the presence of recurrent deletion regions that map to defined antibody epitopes. An excellent example of these recurrent deletions is those acquired in the N-terminal domain of the S glycoprotein and altering defined antibody epitopes during long-term infections of immunocompromised patients [29]. Deletions also occur frequently in accessory open reading frames (ORFs) with various outcomes and potential effects on virus evolution [30,31,32,33]. It was hypothesized that the increased frequency of indels, their non-random distribution and independent co-occurrence in several lineages are other mechanisms of response to elevated global population immunity [34].

In order to understand the factors governing genomic stability, it is therefore important to define the molecular mechanisms of deletions in the viral genome. We performed a statistical analysis of association of deletions and RNA contexts. Specifically, we analyzed the correlations between deletions and repetitive elements in the SARS-CoV-2 genome. We also analyzed the distribution of deletions across the SARS-CoV-2 genes and regions in the *ORF7a* and *ORF8* genes. Hereafter, gene names are italicized; protein names are not italicized. 

## 2. Materials and Methods

Deletions were delineated from the high-quality SARS-CoV-2 genomic alignments (https://www.ncbi.nlm.nih.gov/data-hub/taxonomy/2697049, accessed on 13 December 2021) using the ASM985889v3 (GenBank NC_045512.2) genome as a reference (NCBI datasets). We used whole-genome maximum parsimony phylogenetic trees to predict the loss/gain events for each deletion. Sequences were downloaded on 11/15/2021 from the NCBI SARS-CoV-s Data hub. The requirements for the sequences to be included in the alignment were: (i) sequence length between 29,600 and 31,000 nt; (ii) available collection date; (iii) fraction of ambiguous nucleotides in sequences below 1%; and (iv) trimmed polyA. The resulting number of sequences in the alignment was 633,995. In order to ensure consistency of the alignment, trees were built by a distance method using https://github.com/ncbi/tree-tool (accessed on 13 December 2021) to control for the presence of unusually long branches. Specifically, for every mutation, the maximum parsimony approach was applied in order to apprehend the number of gained and lost nodes. To decrease probability of sequencing errors, only deletions that were present in the alignment 3 times or more were considered to be true. Each deletion was analyzed as a single event. We excluded the *ORF10* from our analyses because it is likely not a protein-coding gene [35]. Deletions and synonymous mutations in the alpha, beta, gamma, and delta SARS-CoV-2 lineages have been extracted from the CoV-GLUE database (https://cov-glue.cvr.gla.ac.uk; (accessed on 10 January 2023); the number of mutations in CoV-GLUE datasets was chosen to be greater or equal 10). Lineages were defined according to the CDC website (https://www.cdc.gov/coronavirus/2019-ncov/variants/variant-classifications.html [accessed on 10 January 2023]). We excluded deletions and substitutions with a frequency greater than 0.01 in order to minimize the chances of shared events.

Analyses of the association of mutations with direct repeats and palindromes were performed using a shuffling approach, as previously described for the analysis of substitutions, insertions and duplications [22,36]. For N studied deletions, a simple functional F (number of matches between two repeated sequences within a fixed window W, W = 5 or 10) was used for a given deletion. Weight F-observed was summed for N deletions. The same procedure was used for a randomized set of N deletions—for each deletion, a random position of deletions across the genome has been generated and F-random was calculated as above. The procedure was repeated 1000 times. The number of cases (X) where F-random is greater than F-observed was calculated. The probability of observed association between deletions and repeats is *p* = X/1000. If *p* < 0.05, then the association was considered significant. The two-tail Fisher exact test (https://www.langsrud.com/fisher.htm [accessed on 10 January 2023]) was used to study 2 × 2 contingency tables. The 2 × 10 exact test (a modification of the 2 × 2 test as implemented in the COLLAPSE program [37]) was used to study the distribution of deletions across the *ORF7a* and *ORF8* genes.

## 3. Results

### 3.1. Description of Dataset

The dataset of deletions was delineated using SARS-CoV-2 multiple alignments and reconstructed phylogenetic trees. The number of short deletions (operationally defined as 1–6 nucleotides deletions) was larger than the number of long deletions (operationally defined as those over 7 nucleotides). The difference between the number of short and long deletions was not substantial (639 vs. 590, Table 1).

It should be noted that the number of 2 and 3 nucleotide deletions in UTRs is approximately the same, although a drop in the number of deletions was expected. The same tendency was observed for 5- and 6-nucleotide deletions (Table 1). This might indicate that some unknown short ORFs are located in UTRs, although this tendency can also be explained by random deviation. 

### 3.2. Mechanisms of Deletions

Short deletions are well-known to be associated with stretches of identical nucleotides or tandemly arranged di- and tri-nucleotides (Figure 1A). This tendency is also observed for 1 nucleotide deletions in SARS-CoV-2 (Table 2). For example, the number of deletions in stretches of 2 identical nucleotides (28) is similar to that of deletions in stretches of 3 and 4 identical nucleotides (27), although the observed numbers of identical stretches in the SARS-CoV-2 genome is dramatically different (4331 vs. 1455, Table 2). This result is highly significant (*p* < 0.00001, the Fisher’s exact test). The excessive frequency of deletions in longer stretches of identical nucleotides strongly suggests that many short deletions are the result of so-call template misalignment in stretches of identical nucleotides (Figure 1A).

**Figure 2:**
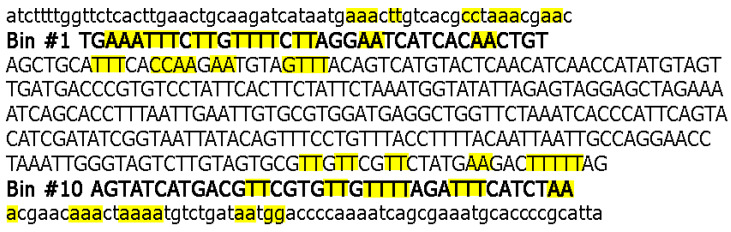
Context properties of the *ORF8* genes. The first bin corresponds to the interval 27,894–27,930; the last bin corresponds to positions 28,222–28,259. The ORF8 protein-coding sequence is shown in capital letters. Runs of identical nucleotides in bins #1 and #10 and flanking regions are shown in yellow.

Nevertheless, more than a half of the single nucleotide deletions (59 out of 103 for coding regions and 37 out of 59 for UTRs) are not associated with stretches of identical nucleotides (examples of such stretches are shown in the Figure 2). A similar tendency was also observed for dinucleotides (only 14–17% of deletions are associated with tandem repletion of dinucleotides, e.g., deletion of GT in the GTGT context, the position 29,759, Table 2, Figures S1–S4) and deletions of length 3, 4, 5, and 6 (Table 2). It should be noted that a substantial fraction of short deletions with a length 3–6 nucleotides (15–16%) is still associated with stretches of identical polynucleotides similar to 1- and 2-nucleotide deletions. All these results are hallmarks of the template dislocation model (Figure 1A).

**Figure 3:**
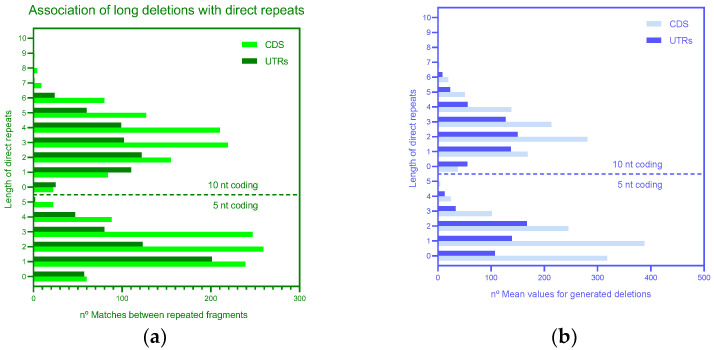
Association of long deletions with direct repeats. (**a**) Number of matches between repeated fragments. (**b**) Mean values for the generated deletions. Heatmap of the association of long deletions with direct repeats in UTR and CDS expressed as the number of matches between repeated fragments is shown in the Figure S5.

**Figure 4:**
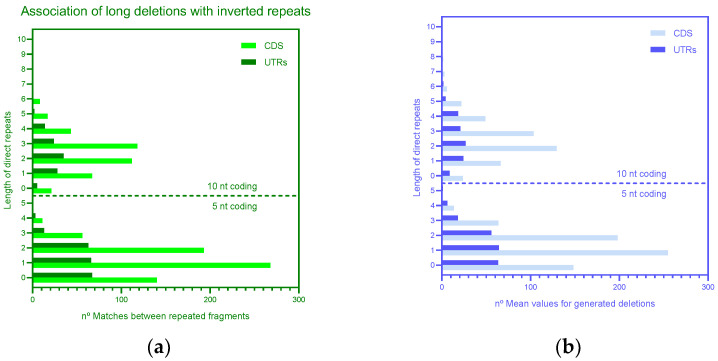
Association of long deletions with inverted repeats. (**a**) Number of matches between repeated fragments. (**b**) Mean values for the generated deletions. Heatmap of the association of long deletions with inverted repeats in UTR and CDS expressed as the number of matches between repeated fragments is shown in the Figure S6.

Analysis of long deletions suggested that many 3′ flanking regions and regions at the ends of deletions are indeed direct repeats with 0 or 1–2 mismatches for both 10 and 5 nucleotide windows and for both the coding regions and UTRs (Figures S2 and S3) according to the template switch model. Strong statistical support of the association between direct repeats (Figure 1B) and deletion (*p* < 0.001 according to the shuffling procedure, Figure 3) suggested that this association reflects real mechanisms of deletions. Overall, all of these results are consistent with the template switch model (Figure 1B). Analyses of inverted repeats according to the hairpin removal model (Figure 1C) did not detect any obvious associations of deletions and inverted repeats (Figure 4); in all these cases, the probability of associations was over 0.05.

### 3.3. Distribution of Deletions across Genes

An important feature of short and long deletions is a substantial excess of short and long deletions in UTRs compared to coding regions: the frequency (per one nucleotide) of all deletions in the coding regions (1227/29,421 = 0.04) is much smaller compared to the corresponding value in UTRS (280/494 = 0.57) (Table 1 and Table 2). This result suggested that the low density of deletions in coding regions reflects true deletion events rather than sequencing error. 

Analysis of in-frame and out-of-frame deletions detected a significant excess of in-frame mutations (Table 1). In-frame deletions are expected to have much smaller functional consequences compared to out-of-frame deletions. The distribution of out-of-frame and in-frame deletions in coding regions is dramatically different from deletions in UTRs. In general, a consistent excess of in-frame deletions is the obvious trend of both long and short deletions (Table 1). 

Analysis of individual genes suggested that just a few long deletions have been detected for *ORF1ab*, *E*, *M*, and *N*. Most short deletions in *ORF1ab* are in-frame, suggesting that at least some of them are real and not just products of sequencing errors. 

**Figure 5:**
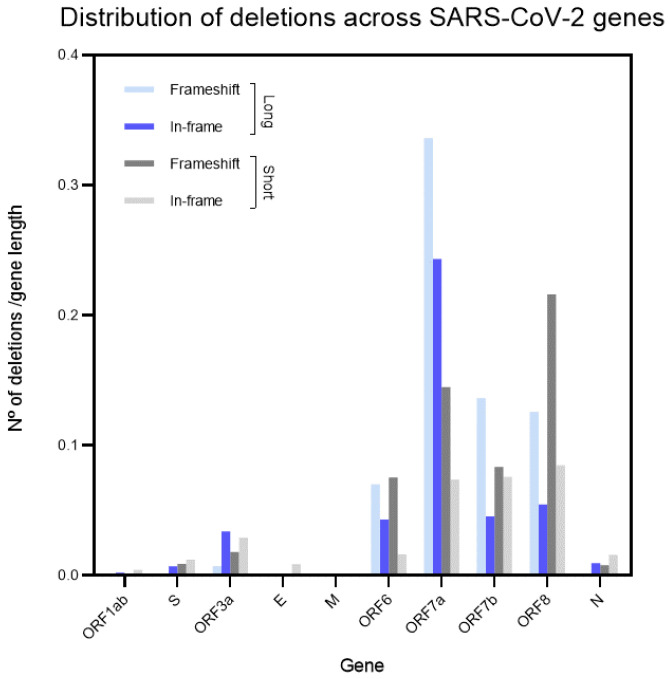
Distribution of deletions across SARS-CoV-2 genes; the Y-axis represents the number of deletions divided by the gene length (the density of deletions).

An interesting property of deletions in the SARS-CoV-2 genome is a dramatic excess of deletion in *ORF7a* and *ORF8* compared to the rest of SARS-CoV-2 (Figure 5). The *ORF7a* is characterized by an excess of both long and short deletions (Figure 5 and Table S1). The *ORF8* is associated mainly with short deletions, although it has the second largest number of long deletions compared to other genes. Out-of-frame long deletions are a prominent feature of the *ORF7a* gene, while in-frame and out-of-frame short deletions in both genes are close to the expected ratio (approximately 2:1 for out-of-frame and in-frame deletions) (Figure 5). *ORF6* and *ORF7b* also have relatively large numbers of short and long deletions, considering that they are the shortest among the studied genes.

Analyses of the association of deletions and direct/inverted repeats suggested that there is indeed a significant association of long deletions in *ORF7a* and direct repeats (consistent with the template switch model, Figure 1B) similar to the whole sequence (Table S2). The *ORF8* has the second-largest number of deletions, however there is no significant association between deletions and direct repeats (Table S2). Deletions in *ORF7a*/*ORF8* are characterized by F-observed/F-random, similar to all other genes (Table S2). These results suggest that the template switch model cannot explain the excessive number of deletions in these genes.

We also performed comparative analyses of the distributions of deletions across genes in four SARS-CoV-2 lineages (mutations in alpha, beta, delta and gamma lineages were extracted from the CoV-GLUE database; Table S3). Synonymous substitutions were used as a control, because this class of mutations is assumed to be effectively neutral except in rare cases [36]. There are obvious differences between lineages (Table S3). However, the density of deletions (the number of deletions divided by the gene length) for *ORF7a* and *ORF8* is always larger (or even much larger) than for other genes, except for the alpha lineage, in which densities of deletions in *ORF7a*, *ORF7b*, and *OFR8* are somewhat similar to densities of deletions in other genes (for example, *ORF1ab*; Table S3). A similar pattern was observed in the gamma strain for the *E* gene only (Table S3). In general, the NCBI and CoV-GLUE datasets produced consistent results: the densities of deletions in studied accessory genes are larger (or even much larger) compared to other genes in both datasets (Figure 5 and Table S3). It should be noted that the alpha lineage shows substantial deviations from the other three lineages (Table S3). For example, the number of deletions (5809) is not dramatically different from the number of synonymous mutations in the alpha lineage (10,334 mutations, the ratio = 0.56, Table S3), whereas this ratio is much smaller (0.12–0.23) for the other three lineages (Table S3). The fraction of deletions in genes other than accessory genes is much higher (for example, *ORF1ab*) compared to such genes in other SARS-CoV-2 lineages (Table S3).

### 3.4. Deletions in ORF7a and ORF8: Putative Functional Consequences

**Figure 6:**
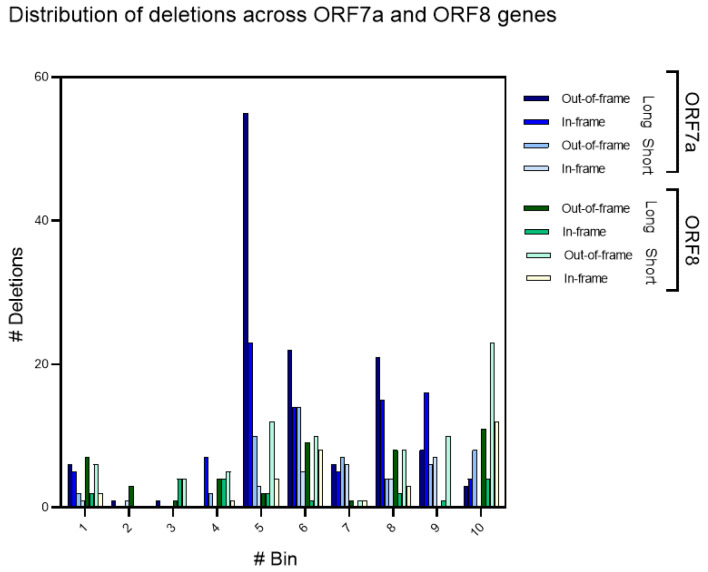
Distribution of deletions across the *ORF7a* and *ORF8* genes. The difference between out-of-frame and in-frame long deletions in *ORF7a* is statistically significant: *p* = 0.003 according to the 2 × 10 exact test. Heatmap of the distribution of in- and out-of-frame deletions in UTR and CDS for each bin (1 to 10) is shown in the Figure S7.

Analyses of the distributions of deletions across genes may provide valuable information about the mechanisms of deletions and illuminate possible functional consequences [38]. The distribution of deletions across the *ORF7a* gene is presented in Figure 6 and Table S4. An excessive amount of out-of-frame long deletions is evident for bin #5 (Figure 6 and Table S4). Analyses of deletions in this bin did not reveal any obvious context properties associated with this hotspot of long deletions: the ratio of F-observed to F-random for association with direct repeats is approximately the same for bin #5 and all other bins (Table S2). Thus, an excessive frequency of deletions in bin #5 is unlikely to be associated with direct repeats. In general, flanking direct repeats have many mismatches and different locations (Figure S3).

Another prominent feature of all studied distributions (in-frame and out-of-frame long and short deletions) is a significantly higher frequency of deletions for the *ORF7a* gene in bins #5–10 compared to bins #1–4 (Figure 6 and Table S4). The probability of such heterogeneity for ORF7a is ~10^−20^ according to the two-tail Fisher exact test (2 × 2, numbers of deletions in bins #1–4 and bins #5–10 [26 and 268] vs. the number of nucleotides within bins #1–4 and #5–10 [146 and 220]) (in-frame and out-of-frame long and short deletions in ORF7a and ORF8 genes were merged) (Figure 6). It should be noted that despite visual similarities of distributions of in-frame and out-of-frame long deletions, there is still a significant difference between them (*p* = 0.003, the 2 × 10 test).

Analyses of the distributions of deletions across the *ORF8* gene suggested that there is a hotspot of in-frame and out-of-frame short deletions in the bin #10 (45 deletions) (Figure 5). Analyses of the short runs of identical (poly)nucleotides in this bin (shown in Figure 2 and Figure S4) did not reveal any obvious context properties that are causing this hotspot of deletions; many short deletions are untemplated (Figure S4). The density of runs of identical nucleotides in bin #10 and the flanking region is similar to bin #1 and the flanking region that has a substantially smaller number of short deletions (6 out-of-frame and 2 in-frame deletions). The difference between bin #1 and bin #10 is significant: *p* = 4 × 10^−5^ according to the Fisher exact test. 

A higher frequency of deletions in *ORF8* bins #5–10 compared to bins #1–4 for in-frame and out-of-frame long and short deletions was found (Figure 6). This feature is highly significant with a *p* of 2 × 10^−5^ according to the two-tail Fisher exact test (numbers of deletions in bins #1–4 and #5–10 [41 and 135] vs. the number of nucleotides within bins #1–4 and #5–10 [146 and 220]). This property of deletions is highly similar to the *ORF7a* gene (Figure 6) suggesting shared mechanisms of the generation of these biased distributions. It should be noted that accessory genes *ORF6* and *ORF7b* have relatively large numbers of short and long deletions (Figure 5). However, the short lengths of these genes (Table S1) do not allow for detailed statistical analyses.

## 4. Discussion

The SARS-CoV-2 genome is a ~30 kb long, single-stranded, positive RNA molecule with the typical gene organization of coronaviruses. There are 12 ORFs that encode 26 proteins, including 16 non-structural proteins (NSP1 to NSP16), four structural proteins (M, N, S, and E), and six accessory proteins (ORF3a, ORF6, ORF7a, ORF7b, ORF8). Accessory proteins are dispensable for replication in cell cultures, but they may play regulatory roles during the viral cycle in host cells and, thus, contribute to fitness of the virus by increasing its ability to evade/modify the host’s immune response [30,39]. Coronaviruses usually differ in those accessory proteins, and more infective species sometimes have specific pathogenic features associated with these proteins [40].

An interesting property of deletions in the SARS-CoV-2 genome is a dramatic excess of deletion in *ORF7a* and *ORF8* compared to other loci. The 122-residue protein ORF7a of SARS-CoV-2 contains a 15 amino acid (aa)-long N-terminal signal peptide, an 81-residue luminal domain (immunoglobulin [Ig]-like domain), a 20 aa transmembrane domain (TMD), and a 5 aa-long cytosolic tail [41]. The luminal domain has a 7-stranded ß-sandwich fold typical of the Ig superfamily [42]. It is highly similar to the SARS-CoV ortholog (85.3%). The product of the *ORF8* gene is a 122 aa protein consisting of an N-terminal signal sequence followed by a predicted Ig-like fold and TMD [41,43]. In general, the domain architectures of ORF7a and ORF8 are similar to each other. With a below 20% sequence identicality to SARS-CoV ORF8, SARS-CoV-2 ORF8 is a fast-evolving protein [44]. Our analyses of deletions suggested that there may an excessive variability in the *ORF7a* and *ORF8* genes; however, this recurrent search in a “sequence space” did not cause increased viability of SARS-CoV-2 variants until now. Still, it is a possibility that at some point in time, deletions can produce some variants that are much more successful compared to the initial variants of SARS-CoV-2. 

Comparative analyses of various SARS-CoV-2 lineages did not reveal any major differences in deletions in accessory proteins; densities of deletions in *ORF6*, *ORF7a*, *ORF7b*, and *ORF8* are always the largest ones in all studied lineages (Table S3), supporting the hypothesis of recurrent searches in a “sequence space” of accessory proteins. The alpha lineage contains an increased number of deletions in genes other than accessory proteins (Table S3). We cannot exclude that genome sequences from the alpha lineage contain a larger fraction of sequencing errors compared to other lineages. Another possible explanation is that the increased variations of the structure of accessory proteins in later SARS-CoV-2 lineages is indeed a response to increasing immunity levels due to the overwhelming spread of COVID-19.

The *ORF6* and *ORF7b* genes (coding for the members of “ORF6-ORF7a-ORF7b-ORF8 complex” of accessory proteins) also have relatively large numbers of short and long deletions considering that these are the shortest genes among those studied (Table S1). A somewhat similar process was documented in the spike protein, where it was found that recurrent deletions arising from diverse genetic and geographic backgrounds can be transmitted efficiently and are present in novel lineages, including those of current global concern [29]. These deletions frequently occupy recurrent deletion regions, which map to the defined spike antibody epitopes. Deletions in recurrent deletion regions may confer resistance to neutralizing antibodies. It is plausible to suggest that these deletions in the SARS-CoV-2 spike glycoprotein drive an escape from host immune systems. By altering subsequences of amino acids, deletions may accelerate SARS-CoV-2 antigenic evolution and might, more generally, drive adaptive evolution [29,34]. Similarly, potentially important signs of natural selection were documented in the *ORF7a* and *ORF8* genes: there are excessive numbers of long and short deletions in the second half of both genes. There is also a significant difference between in-frame and out-of-frame long deletion in *ORF7a* (*p* = 0.003) (Figure 6). 

Although deletions appear to be important functional events, sequencing errors cannot be ruled out. These errors are known to be one of the major problems in comparative genomics. Analyses of in-frame and out-of-frame deletions and the distribution of long deletions across the SARS-CoV-2 genome suggested that long deletions are unlikely to be the result of sequencing errors. In addition, we analyzed only cases of multiple (3 or more) instances of each deletion in order to decrease the chances of such errors [22]. Thus, it is likely that sequencing errors constitute only a small fraction of the studied long deletions. However, short deletions in stretches of identical nucleotides may result in recurrent events and/or are being contaminated with sequencing errors.

Analyses of the mechanisms of deletions (Figure 1, Table 1, Table 2 and Table S2) suggested that direct repeats and stretches of identical nucleotides are associated with deletions and, thus, are likely to play an important role in their generation. Inverted repeats (that are the bases of hairpin structures) show no association with deletions (Figure 4). In general, no overwhelming association with repeats was detected for long deletions, suggesting that the homoplasy of these markers is not substantial (if there is any at all). Sequencing errors and mechanisms of deletions do not seem to be responsible for the explosion of long and short deletion events in accessory genes and the uneven distribution of deletions across *ORF7a* and *ORF8* genes. Thus, the major driver of numerous short and long deletions in the “ORF6-ORF7a-ORF7b-ORF8 complex” of accessory proteins is likely to be natural selection. The functional importance of recombination in SARS-CoV-2 is supported by the PRRA insertion, which is a characteristic feature of SARS-CoV-2. It causes major functional consequences and is associated with various overlapping functions [45,46,47,48]. 

The proposed hypothesis that deletions are likely to be an important factor in the evolution of viruses is further supported by previous analyses of SARS-CoV *ORF8* [31,44,49,50,51,52,53]. It is well-established that one important difference between SARS-CoV-2 and SARS-CoV is the 29-nucleotide deletion in *ORF8* resulting in the splitting of *ORF8* into two smaller ORFs, namely *ORF8a* and *ORF8b*. In our study, the SARS-CoV-2 *ORF8* variability is associated with the end of this gene (Figure 6). Paradoxically, an excess of deletions in bin #5 of *ORF7a* echoes with the SARS-CoV *ORF8* deletions. In other words, the position of this characteristic feature of SARS-CoV *ORF8* is similar to the position of multiple deletions in the *ORF7a* gene (deletions events near the middle of genes, Figure 6). It should be noted that ORF7a and ORF8 share the same domain structure [43]. Thus, the functional similarity of these proteins cannot be excluded. 

Members of the “ORF6-ORF7a-ORF7b-ORF8 complex” of accessory proteins are characterized by excessive numbers of short and long deletions (Figure 5) that have the potential to cause major functional innovations, similar to the PRRA insertion in SARS-CoV-2 and the 29bp deletion in SARS-CoV. Thus, there is a possibility that at some point of time, deletions can produce some variants that are much more successful compared to the initial variants of SARS-CoV-2, although long-term functional consequences of deletions events in the viruses remain to be investigated further.

## Figures and Tables

**Table 1:** Statistics of short and long deletions ^1^.

Statistics of Short Deletions (1–6 nt)			
Coding		UTRs	
Length	# deletions	Length	# deletions
1	103	1	53
2	74	2	29
3	178	3	24
4	36	4	10
5	23	5	17
6	80	6	12
**Total**	**494**	**Total**	**145**
**Statistics of long deletions (>6 nt)**			
**Coding**			
**# deletions**	**Mean length**	**In-frame**	**Out-of-frame**
453	33.1	237	216
**UTRs**			
**# deletions**	**Mean length**	**In-frame**	**Out-of-frame**
137	35.2	48	89

^1^ “In-frame” and “out-of-frame” deletions were produced using starting positions of “ORFs” (ORFs with stop codons allowed to be “translated”) as positions +1 (the first position of UTR), +2 and +3. “#” means the “Number of”.

**Table 2:** Association of short deletion with repetitive elements.

Short Deletions (1 nt)					
Coding			UTRs		
Length	# deletions	# stretches ^1^	Length	# deletions	# stretches ^1^
1 ^2^	59	15,663	1 ^2^	37	265
2	20	4269	2	8	62
3	12	1092	3	4	16
4	8	341	4	3	6
5	4	106			
**Total**	**103**		**Total**	**52**	
**Short deletions (2 nt)**					
**Coding**			**UTRs**		
**Dinucleotide stretches**		**# deletions**	**Dinucleotide stretches**		**# deletions**
Yes		10	Yes		5
No		64	No		24
**Short deletions (3–6 nt)**					
**Coding**			**UTRs**		
**Polynucleotide stretches**		**# deletions**	**Polynucleotide stretches**		**# deletions**
Yes		46	Yes		10
No		271	No		53

^1^ Number of stretches of identical nucleotides; ^2^ no stretches of identical (poly)nucleotides consistent with near positions of deletions were found. “#” means the “Number of”.

## Data Availability

Publicly available datasets were analyzed in this study. These data can be found here: https://www.ncbi.nlm.nih.gov/data-hub/taxonomy/2697049 (accessed on 13 December 2021) and Figures S1 and S2.

## Supplementary Materials

The following supporting information can be downloaded at: https://www.mdpi.com/article/10.3390/microorganisms11010229/s1, Figure S1: Contexts of short deletions in SARS-CoV-2; Figure S2: Contexts of long deletions in SARS-CoV-2; Figure S3: Contexts of long deletions in the bin #5 of ORF7a; Figure S4: Contexts of short deletions in the bin #10 of ORF8; Figure S5: Heatmap of the association of long deletions with direct repeats in UTR and CDS, considering Windows = 5 and 10 nucleotides, expressed as number of matches between repeated fragments; Figure S6: Heatmap of the association of long deletions with inverted repeats in UTR and CDS, considering Windows = 5 and 10 nucleotides, expressed as number of matches between repeated fragments; Figure S7: Heatmap of the distribution of in- and out-of-frame deletions in UTR and CDS for each bin (1 to 10), according to their length (Short and long); Table S1: Distribution of deletions across SARS-CoV-2 genes; Table S2: Statistics of association of direct repeats and long deletions; Table S3: Distribution of deletions across genes in various SARS-CoV-2 lineages; Table S4: Distribution of deletions across *ORF7a* and *ORF8* genes.
