# Supplementary material for: Deletions across the SARS-CoV-2 Genome: Molecular Mechanisms and Putative Functional Consequences of Deletions in Accessory Genes
Source: Microorganisms. 2023 Jan 16;11(1):229. doi: 10.3390/microorganisms11010229 (PMC9862619; doi:10.3390/microorganisms11010229)
Supplement: Supplementary file 1 [file microorganisms-11-00229-s001.zip › Figure S1.pdf]

Supplementary Figure S1. Contexts of short deletions in SARS-CoV-2.
The 1st number is the postion and the 5th number is the number of instances
followed by gene names.

28274 1 159 2594 405321 N-M1fs
TATCATGACGTTCGTGTTGTTTTAGATTTCATCTAAACGAACAAACTAAA
a
TGTCTGATAATGGACCCCAAAATCAGCGAAATGCACCCCGCATTACGTTTG

21994 3 470 1964 331338 S-Y145del
ATTAAAGTCTGTGAATTTCAATTTTGTAATGATCCATTTTTGGGTGTTTA
tta
CCACAAAAACAACAAAAGTTGGATGGAAAGTGAGTTCAGAGTTTATTCTAG

21766 6 401 1382 327847 S-HV69del
CAGGACTTGTTCTTACCTTTCTTTTCCAATGTTACTTGGTTCCATGCTAT
aca tgt
CTCTGGGACCAATGGTACTAAGAGGTTTGATAACCCTGTCCTACCATTTAA

28248 6 25 83 52612 ORF8-DF119del
GTTCGTTCTATGAAGACTTTTTAGAGTATCATGACGTTCGTGTTGTTTTA
gat ttc
ATCTAAACGAACAAACTAAAATGTCTGATAATGGACCCCAAAATCAGCGAA

22029 6 14 70 53385 S-EFR156G
ATTTTTGGGTGTTTATTACCACAAAAACAACAAAAGTTGGATGGAAAGTG
agt tca
GAGTTTATTCTAGTGCGAATAATTGCACTTTTGAATATGTCTCTCAGCCTT

515 3 979 61 2533 ORF1a-V84del
CCTATGTGTTCATCAAACGTTCGGATGCTCGAACTGCACCTCATGGTCAT
gtt
ATGGTTGAGCTGGTAGCAGAACTCGAAGGCATTCAGTACGGTCGTAGTGGT

517 3 1265 59 1862 ORF1a-M85del
TATGTGTTCATCAAACGTTCGGATGCTCGAACTGCACCTCATGGTCATGT
tat
GGTTGAGCTGGTAGCAGAACTCGAAGGCATTCAGTACGGTCGTAGTGGTGA

29769 1 282 28 565
CACCACATTTTCACCGAGGCCACGCGGAGTACGATCGAGTGTACAGTGAA
c
AATGCTAGGGAGAGCTGCCTATATGGAAGAGCCCTAATGTGTAAAATTAAT

28254 1 534 23 3208 ORF8-I121fs
TCTATGAAGACTTTTTAGAGTATCATGACGTTCGTGTTGTTTTAGATTTC
a
TCTAAACGAACAAACTAAAATGTCTGATAATGGACCCCAAAATCAGCGAAA

1606 3 17 7 344 ORF1a-D448del
ATAGGTTGTAACCATACAGGTGTTGTTGGAGAAGGTTCCGAAGGTCTTAA
tga

CAACCTTCTTGAAATACTCCAAAAAGAGAAAGTCAACATCAATATTGTTGG

29729 1 71 7 278
CTTTAATCAGTGTGTAACATTAGGGAGGACTTGAAAGAGCCACCACATTT
t
CACCGAGGCCACGCGGAGTACGATCGAGTGTACAGTGAACAATGCTAGGGA

21764 1 81 5 1723 S-I68fs
CTCAGGACTTGTTCTTACCTTTCTTTTCCAATGTTACTTGGTTCCATGCT
a
TACATGTCTCTGGGACCAATGGTACTAAGAGGTTTGATAACCCTGTCCTAC

21762 1 77 5 1709 S-A67fs
AACTCAGGACTTGTTCTTACCTTTCTTTTCCAATGTTACTTGGTTCCATG
c
TATACATGTCTCTGGGACCAATGGTACTAAGAGGTTTGATAACCCTGTCCT

29711 1 18 5 1442
TTAATCTCACATAGCAATCTTTAATCAGTGTGTAACATTAGGGAGGACTT
g
AAAGAGCCACCACATTTTCACCGAGGCCACGCGGAGTACGATCGAGTGTAC

27205 3 3 5 1091 ORF6-F2del
CAGTAGCAGTGACAATATTGCTTTGCTTGTACAGTAAGTGACAACAGATG
ttt
CATCTCGTTGACTTTCAGGTTACTATAGCAGAGATATTACTAATTATTATG

518 3 255 5 415 ORF1a-M85del
ATGTGTTCATCAAACGTTCGGATGCTCGAACTGCACCTCATGGTCATGTT
atg
GTTGAGCTGGTAGCAGAACTCGAAGGCATTCAGTACGGTCGTAGTGGTGAG

28273 2 5 5 319 N-M1fs
GTATCATGACGTTCGTGTTGTTTTAGATTTCATCTAAACGAACAAACTAA
aa
TGTCTGATAATGGACCCCAAAATCAGCGAAATGCACCCCGCATTACGTTTG

28032 2 4 5 79 ORF8-I47fs
CATATGTAGTTGATGACCCGTGTCCTATTCACTTCTATTCTAAATGGTAT
at
TAGAGTAGGAGCTAGAAAATCAGCACCTTTAATTGAATTGTGCGTGGATGA

21767 4 84 4 1808 S-HV69fs
AGGACTTGTTCTTACCTTTCTTTTCCAATGTTACTTGGTTCCATGCTATA
cat g
TCTCTGGGACCAATGGTACTAAGAGGTTTGATAACCCTGTCCTACCATTTA

28278 3 7 4 1080 N-SD2Y
ATGACGTTCGTGTTGTTTTAGATTTCATCTAAACGAACAAACTAAAATGT
ctg
ATAATGGACCCCAAAATCAGCGAAATGCACCCCGCATTACGTTTGGTGGAC

26158 4 83 4 394 ORF3a-VN256fs
AGCCTGAAGAACATGTCCAAATTCACACAATCGACGGTTCATCCGGAGTT

gtt a
ATCCAGTAATGGAACCAATTTATGATGAACCGACGACGACTACTAGCGTGC

27795 2 63 4 311 ORF7b-L14fs
AGAAAGACAGAATGATTGAACTTTCATTAATTGACTTCTATTTGTGCTTT
tt
AGCCTTTCTGCTATTCCTTGTTTTAATTATGCTTATTATCTTTTGGTTCTC

22191 3 16 3 269 S-I210del
GTTTAAGAATATTGATGGTTATTTTAAAATATATTCTAAGCACACGCCTA
tta
ATTTAGTGCGTGATCTCCCTCAGGGTTTTTCGGCTTTAGAACCATTGGTAG

22289 6 86 3 137 S-AL243del
TGGTAGATTTGCCAATAGGTATTAACATCACTAGGTTTCAAACTTTACTT
gct tta
CATAGAAGTTATTTGACTCCTGGTGATTCTTCTTCAGGTTGGACAGCTGGT

21563 1 24 3 114 S-M1fs
CAACAGAGTTGTTATTTCTAGTGATGTTCTTGTTAACAACTAAACGAACA
a
TGTTTGTTTTTCTTGTTTTATTGCCACTAGTCTCTAGTCAGTGTGTTAATC

22295 2 6 3 51 S-H245fs
ATTTGCCAATAGGTATTAACATCACTAGGTTTCAAACTTTACTTGCTTTA
ca
TAGAAGTTATTTGACTCCTGGTGATTCTTCTTCAGGTTGGACAGCTGGTGC

29771 6 5 3 50
CCACATTTTCACCGAGGCCACGCGGAGTACGATCGAGTGTACAGTGAACA
atg cta
GGGAGAGCTGCCTATATGGAAGAGCCCTAATGTGTAAAATTAATTTTAGTA

518 6 307 2 464 ORF1a-MV85del
ATGTGTTCATCAAACGTTCGGATGCTCGAACTGCACCTCATGGTCATGTT
atg gtt
GAGCTGGTAGCAGAACTCGAAGGCATTCAGTACGGTCGTAGTGGTGAGACA

21766 3 100 2 461 S-H69del
CAGGACTTGTTCTTACCTTTCTTTTCCAATGTTACTTGGTTCCATGCTAT
aca
TGTCTCTGGGACCAATGGTACTAAGAGGTTTGATAACCCTGTCCTACCATT

21770 3 90 2 445 S-V70del
ACTTGTTCTTACCTTTCTTTTCCAATGTTACTTGGTTCCATGCTATACAT
gtc
TCTGGGACCAATGGTACTAAGAGGTTTGATAACCCTGTCCTACCATTTAAT

27745 1 3 2 296 ORF7a-R118fs
TTCTTATTGTTGCGGCAATAGTGTTTATAACACTTTGCTTCACACTCAAA
a
GAAAGACAGAATGATTGAACTTTCATTAATTGACTTCTATTTGTGCTTTTT

27795 3 34 2 114 ORF7b-L14del

AGAAAGACAGAATGATTGAACTTTCATTAATTGACTTCTATTTGTGCTTT
tta
GCCTTTCTGCTATTCCTTGTTTTAATTATGCTTATTATCTTTTGGTTCTCA

28066 4 7 2 53 ORF8-IE58fs
CTATTCTAAATGGTATATTAGAGTAGGAGCTAGAAAATCAGCACCTTTAA
ttg a
ATTGTGCGTGGATGAGGCTGGTTCTAAATCACCCATTCAGTACATCGATAT

29760 1 14 2 41
TGAAAGAGCCACCACATTTTCACCGAGGCCACGCGGAGTACGATCGAGTG
t
ACAGTGAACAATGCTAGGGAGAGCTGCCTATATGGAAGAGCCCTAATGTGT

29758 1 7 2 28
CTTGAAAGAGCCACCACATTTTCACCGAGGCCACGCGGAGTACGATCGAG
t
GTACAGTGAACAATGCTAGGGAGAGCTGCCTATATGGAAGAGCCCTAATGT

28090 6 150 1 864 ORF8-GSK66E
AGGAGCTAGAAAATCAGCACCTTTAATTGAATTGTGCGTGGATGAGGCTG
gtt cta
AATCACCCATTCAGTACATCGATATCGGTAATTATACAGTTTCCTGTTTAC

21993 2 71 1 567 S-Y144fs
TATTAAAGTCTGTGAATTTCAATTTTGTAATGATCCATTTTTGGGTGTTT
at
TACCACAAAAACAACAAAAGTTGGATGGAAAGTGAGTTCAGAGTTTATTCT

21989 1 46 1 476 S-V143fs
TTGTTATTAAAGTCTGTGAATTTCAATTTTGTAATGATCCATTTTTGGGT
g
TTTATTACCACAAAAACAACAAAAGTTGGATGGAAAGTGAGTTCAGAGTTT

21763 4 21 1 378 S-AI67fs
ACTCAGGACTTGTTCTTACCTTTCTTTTCCAATGTTACTTGGTTCCATGC
tat a
CATGTCTCTGGGACCAATGGTACTAAGAGGTTTGATAACCCTGTCCTACCA

27579 3 131 1 364 ORF7a-QF62H
TTTCATCCTCTAGCTGATAACAAATTTGCACTGACTTGCTTTAGCACTCA
att
TGCTTTTGCTTGTCCTGACGGCGTAAAACACGTCTATCAGTTACGTGCCAG

28896 3 23 1 358 N-AR208G
CAACAGTTCAAGAAATTCAACTCCAGGCAGCAGTAGGGGAACTTCTCCTG
cta
GAATGGCTGGCAATGGCGGTGATGCTGCTCTTGCTTTGCTGCTGCTTGACA

28065 3 61 1 210 ORF8-I58del
TCTATTCTAAATGGTATATTAGAGTAGGAGCTAGAAAATCAGCACCTTTA
att
GAATTGTGCGTGGATGAGGCTGGTTCTAAATCACCCATTCAGTACATCGAT

21998 3 78 1 193 S-H146del
AAGTCTGTGAATTTCAATTTTGTAATGATCCATTTTTGGGTGTTTATTAC
cac
AAAAACAACAAAAGTTGGATGGAAAGTGAGTTCAGAGTTTATTCTAGTGCG

26159 3 68 1 186 ORF3a-VN256D
GCCTGAAGAACATGTCCAAATTCACACAATCGACGGTTCATCCGGAGTTG
tta
ATCCAGTAATGGAACCAATTTATGATGAACCGACGACGACTACTAGCGTGC

3952 3 12 1 156 ORF1a-DK1229E
CCATTTATAACTGAAAGTAAACCTTCAGTTGAACAGAGAAAACAAGATGA
taa
GAAAATCAAAGCTTGTGTTGAAGAAGTTACAACAACTCTGGAAGAAACTAA

28097 1 33 1 140 ORF8-K68fs
AGAAAATCAGCACCTTTAATTGAATTGTGCGTGGATGAGGCTGGTTCTAA
a
TCACCCATTCAGTACATCGATATCGGTAATTATACAGTTTCCTGTTTACCT

27700 3 24 1 123 ORF7a-I103del
TGTTCATCAGACAAGAGGAAGTTCAAGAACTTTACTCTCCAATTTTTCTT
att
GTTGCGGCAATAGTGTTTATAACACTTTGCTTCACACTCAAAAGAAAGACA

29728 2 41 1 118
TCTTTAATCAGTGTGTAACATTAGGGAGGACTTGAAAGAGCCACCACATT
tt
CACCGAGGCCACGCGGAGTACGATCGAGTGTACAGTGAACAATGCTAGGGA

29775 1 75 1 92
ATTTTCACCGAGGCCACGCGGAGTACGATCGAGTGTACAGTGAACAATGC
t
AGGGAGAGCTGCCTATATGGAAGAGCCCTAATGTGTAAAATTAATTTTAGT

26162 1 27 1 92 ORF3a-N257fs
TGAAGAACATGTCCAAATTCACACAATCGACGGTTCATCCGGAGTTGTTA
a
TCCAGTAATGGAACCAATTTATGATGAACCGACGACGACTACTAGCGTGCC

28997 3 16 1 91 N-Q242del
ACAGATTGAACCAGCTTGAGAGCAAAATGTCTGGTAAAGGCCAACAACAA
caa
GGCCAAACTGTCACTAAGAAATCTGCTGCTGAGGCTTCTAAGAAGCCTCGG

9860 3 20 1 90 ORF1a-L3199del
CCTTTTTGTTAAATAAAGAAATGTATCTAAAGTTGCGTAGTGATGTGCTA
tta
CCTCTTACGCAATATAATAGATACTTAGCTCTTTATAATAAGTACAAGTAT

22194 3 8 1 87 S-NL211I
TAAGAATATTGATGGTTATTTTAAAATATATTCTAAGCACACGCCTATTA
att
TAGTGCGTGATCTCCCTCAGGGTTTTTCGGCTTTAGAACCATTGGTAGATT

29769 2 67 1 82
CACCACATTTTCACCGAGGCCACGCGGAGTACGATCGAGTGTACAGTGAA
ca
ATGCTAGGGAGAGCTGCCTATATGGAAGAGCCCTAATGTGTAAAATTAATT

28090 3 6 1 56 ORF8-GS66A
AGGAGCTAGAAAATCAGCACCTTTAATTGAATTGTGCGTGGATGAGGCTG
gtt
CTAAATCACCCATTCAGTACATCGATATCGGTAATTATACAGTTTCCTGTT

29682 1 12 1 55
CTACATAGCACAAGTAGATGTAGTTAACTTTAATCTCACATAGCAATCTT
t
AATCAGTGTGTAACATTAGGGAGGACTTGAAAGAGCCACCACATTTTCACC

28248 4 11 1 52 ORF8-DF119fs
GTTCGTTCTATGAAGACTTTTTAGAGTATCATGACGTTCGTGTTGTTTTA
gat t
TCATCTAAACGAACAAACTAAAATGTCTGATAATGGACCCCAAAATCAGCG

21762 2 7 1 30 S-A67fs
AACTCAGGACTTGTTCTTACCTTTCTTTTCCAATGTTACTTGGTTCCATG
ct
ATACATGTCTCTGGGACCAATGGTACTAAGAGGTTTGATAACCCTGTCCTA

28093 3 16 1 28 ORF8-SK67*
AGCTAGAAAATCAGCACCTTTAATTGAATTGTGCGTGGATGAGGCTGGTT
cta
AATCACCCATTCAGTACATCGATATCGGTAATTATACAGTTTCCTGTTTAC

29754 1 13 1 26
AGGACTTGAAAGAGCCACCACATTTTCACCGAGGCCACGCGGAGTACGAT
c
GAGTGTACAGTGAACAATGCTAGGGAGAGCTGCCTATATGGAAGAGCCCTA

29749 6 7 1 24
TAGGGAGGACTTGAAAGAGCCACCACATTTTCACCGAGGCCACGCGGAGT
acg atc
GAGTGTACAGTGAACAATGCTAGGGAGAGCTGCCTATATGGAAGAGCCCTA

29750 3 13 1 23
AGGGAGGACTTGAAAGAGCCACCACATTTTCACCGAGGCCACGCGGAGTA
cga
TCGAGTGTACAGTGAACAATGCTAGGGAGAGCTGCCTATATGGAAGAGCCC

29770 3 2 1 17
ACCACATTTTCACCGAGGCCACGCGGAGTACGATCGAGTGTACAGTGAAC
aat
GCTAGGGAGAGCTGCCTATATGGAAGAGCCCTAATGTGTAAAATTAATTTT

29754 5 3 1 11
AGGACTTGAAAGAGCCACCACATTTTCACCGAGGCCACGCGGAGTACGAT
cga gt

GTACAGTGAACAATGCTAGGGAGAGCTGCCTATATGGAAGAGCCCTAATGT

29732 2 1 1 10
TAATCAGTGTGTAACATTAGGGAGGACTTGAAAGAGCCACCACATTTTCA
cc
GAGGCCACGCGGAGTACGATCGAGTGTACAGTGAACAATGCTAGGGAGAGC

223 1 4 1 9
CGTCTATCTTCTGCAGGCTGCTTACGGTTTCGTCCGTGTTGCAGCCGATC
a
TCAGCACATCTAGGTTTCGTCCGGGTGTGACCGAAAGGTAAGATGGAGAGC

29751 3 2 1 8
GGGAGGACTTGAAAGAGCCACCACATTTTCACCGAGGCCACGCGGAGTAC
gat
CGAGTGTACAGTGAACAATGCTAGGGAGAGCTGCCTATATGGAAGAGCCCT

28092 3 1 1 8 ORF8-S67del
GAGCTAGAAAATCAGCACCTTTAATTGAATTGTGCGTGGATGAGGCTGGT
tct
AAATCACCCATTCAGTACATCGATATCGGTAATTATACAGTTTCCTGTTTA

29750 6 3 1 6
AGGGAGGACTTGAAAGAGCCACCACATTTTCACCGAGGCCACGCGGAGTA
cga tcg
AGTGTACAGTGAACAATGCTAGGGAGAGCTGCCTATATGGAAGAGCCCTAA

21995 3 80 0 921 S-Y145del
TTAAAGTCTGTGAATTTCAATTTTGTAATGATCCATTTTTGGGTGTTTAT
tac
CACAAAAACAACAAAAGTTGGATGGAAAGTGAGTTCAGAGTTTATTCTAGT

21766 1 45 0 771 S-I68fs
CAGGACTTGTTCTTACCTTTCTTTTCCAATGTTACTTGGTTCCATGCTAT
a
CATGTCTCTGGGACCAATGGTACTAAGAGGTTTGATAACCCTGTCCTACCA

21768 1 23 0 672 S-H69fs
GGACTTGTTCTTACCTTTCTTTTCCAATGTTACTTGGTTCCATGCTATAC
a
TGTCTCTGGGACCAATGGTACTAAGAGGTTTGATAACCCTGTCCTACCATT

21770 1 23 0 672 S-V70fs
ACTTGTTCTTACCTTTCTTTTCCAATGTTACTTGGTTCCATGCTATACAT
g
TCTCTGGGACCAATGGTACTAAGAGGTTTGATAACCCTGTCCTACCATTTA

21772 3 23 0 671 S-S71del
TTGTTCTTACCTTTCTTTTCCAATGTTACTTGGTTCCATGCTATACATGT
ctc
TGGGACCAATGGTACTAAGAGGTTTGATAACCCTGTCCTACCATTTAATGA

26158 3 138 0 324 ORF3a-V256del
AGCCTGAAGAACATGTCCAAATTCACACAATCGACGGTTCATCCGGAGTT

gtt
AATCCAGTAATGGAACCAATTTATGATGAACCGACGACGACTACTAGCGTG

21770 2 30 0 321 S-V70fs
ACTTGTTCTTACCTTTCTTTTCCAATGTTACTTGGTTCCATGCTATACAT
gt
CTCTGGGACCAATGGTACTAAGAGGTTTGATAACCCTGTCCTACCATTTAA

21767 6 80 0 214 S-HV69del
AGGACTTGTTCTTACCTTTCTTTTCCAATGTTACTTGGTTCCATGCTATA
cat gtc
TCTGGGACCAATGGTACTAAGAGGTTTGATAACCCTGTCCTACCATTTAAT

29728 3 10 0 211
TCTTTAATCAGTGTGTAACATTAGGGAGGACTTGAAAGAGCCACCACATT
ttc
ACCGAGGCCACGCGGAGTACGATCGAGTGTACAGTGAACAATGCTAGGGAG

28150 3 2 0 206 ORF8-FT86S
ACCCATTCAGTACATCGATATCGGTAATTATACAGTTTCCTGTTTACCTT
tta
CAATTAATTGCCAGGAACCTAAATTGGGTAGTCTTGTAGTGCGTTGTTCGT

27794 3 56 0 201 ORF7b-F13del
AAGAAAGACAGAATGATTGAACTTTCATTAATTGACTTCTATTTGTGCTT
ttt
AGCCTTTCTGCTATTCCTTGTTTTAATTATGCTTATTATCTTTTGGTTCTC

28914 6 17 0 178 N-GG214del
AACTCCAGGCAGCAGTAGGGGAACTTCTCCTGCTAGAATGGCTGGCAATG
gcg gtg
ATGCTGCTCTTGCTTTGCTGCTGCTTGACAGATTGAACCAGCTTGAGAGCA

28094 3 2 0 122 ORF8-K68del
GCTAGAAAATCAGCACCTTTAATTGAATTGTGCGTGGATGAGGCTGGTTC
taa
ATCACCCATTCAGTACATCGATATCGGTAATTATACAGTTTCCTGTTTACC

28240 2 4 0 115 ORF8-V116fs
AGTGCGTTGTTCGTTCTATGAAGACTTTTTAGAGTATCATGACGTTCGTG
tt
GTTTTAGATTTCATCTAAACGAACAAACTAAAATGTCTGATAATGGACCCC

27697 6 31 0 109 ORF7a-LI102del
AACTGTTCATCAGACAAGAGGAAGTTCAAGAACTTTACTCTCCAATTTTT
ctt att
GTTGCGGCAATAGTGTTTATAACACTTTGCTTCACACTCAAAAGAAAGACA

28247 1 5 0 104 ORF8-L118fs
TGTTCGTTCTATGAAGACTTTTTAGAGTATCATGACGTTCGTGTTGTTTT
a
GATTTCATCTAAACGAACAAACTAAAATGTCTGATAATGGACCCCAAAATC

28249 2 4 0 100 ORF8-D119fs

TTCGTTCTATGAAGACTTTTTAGAGTATCATGACGTTCGTGTTGTTTTAG
at
TTCATCTAAACGAACAAACTAAAATGTCTGATAATGGACCCCAAAATCAGC

28028 3 18 0 95 ORF8-WY45C
CAACCATATGTAGTTGATGACCCGTGTCCTATTCACTTCTATTCTAAATG
gta
TATTAGAGTAGGAGCTAGAAAATCAGCACCTTTAATTGAATTGTGCGTGGA

26159 2 7 0 94 ORF3a-V256fs
GCCTGAAGAACATGTCCAAATTCACACAATCGACGGTTCATCCGGAGTTG
tt
AATCCAGTAATGGAACCAATTTATGATGAACCGACGACGACTACTAGCGTG

204 3 5 0 90
TGACAGGACACGAGTAACTCGTCTATCTTCTGCAGGCTGCTTACGGTTTC
gtc
CGTGTTGCAGCCGATCATCAGCACATCTAGGTTTCGTCCGGGTGTGACCGA

6513 3 26 0 89 ORF1a-SL2083I
GAAAACTACCGAAGTTGTAGGAGACATTATACTTAAACCAGCAAATAATA
gtt
TAAAAATTACAGAAGAGGTTGGCCACACAGATCTAATGGCTGCTTATGTAG

519 6 72 0 84 ORF1a-MVE85K
TGTGTTCATCAAACGTTCGGATGCTCGAACTGCACCTCATGGTCATGTTA
tgg ttg
AGCTGGTAGCAGAACTCGAAGGCATTCAGTACGGTCGTAGTGGTGAGACAC

27897 5 2 0 83 ORF8-KF2fs
CACTTGAACTGCAAGATCATAATGAAACTTGTCACGCCTAAACGAACATG
aaa tt
TCTTGTTTTCTTAGGAATCATCACAACTGTAGCTGCATTTCACCAAGAATG

27722 2 25 0 81 ORF7a-I110fs
TCAAGAACTTTACTCTCCAATTTTTCTTATTGTTGCGGCAATAGTGTTTA
ta
ACACTTTGCTTCACACTCAAAAGAAAGACAGAATGATTGAACTTTCATTAA

222 1 44 0 78
TCGTCTATCTTCTGCAGGCTGCTTACGGTTTCGTCCGTGTTGCAGCCGAT
c
ATCAGCACATCTAGGTTTCGTCCGGGTGTGACCGAAAGGTAAGATGGAGAG

21982 3 43 0 76 S-F140del
ACTAATGTTGTTATTAAAGTCTGTGAATTTCAATTTTGTAATGATCCATT
ttt
GGGTGTTTATTACCACAAAAACAACAAAAGTTGGATGGAAAGTGAGTTCAG

522 3 65 0 74 ORF1a-V86del
GTTCATCAAACGTTCGGATGCTCGAACTGCACCTCATGGTCATGTTATGG
ttg
AGCTGGTAGCAGAACTCGAAGGCATTCAGTACGGTCGTAGTGGTGAGACAC

21990 3 36 0 74 S-VY143D
TGTTATTAAAGTCTGTGAATTTCAATTTTGTAATGATCCATTTTTGGGTG
ttt
ATTACCACAAAAACAACAAAAGTTGGATGGAAAGTGAGTTCAGAGTTTATT

25431 6 16 0 73 ORF3a-TL14del
ATAAACGAACTTATGGATTTGTTTATGAGAATCTTCACAATTGGAACTGT
aac ttt
GAAGCAAGGTGAAATCAAGGATGCTACTCCTTCAGATTTTGTTCGCGCTAC

29773 2 5 0 73
ACATTTTCACCGAGGCCACGCGGAGTACGATCGAGTGTACAGTGAACAAT
gc
TAGGGAGAGCTGCCTATATGGAAGAGCCCTAATGTGTAAAATTAATTTTAG

27622 3 4 0 73 ORF7a-L77del
GCACTCAATTTGCTTTTGCTTGTCCTGACGGCGTAAAACACGTCTATCAG
tta
CGTGCCAGATCAGTTTCACCTAAACTGTTCATCAGACAAGAGGAAGTTCAA

26485 2 8 0 72
ATCTGAATTCTTCTAGAGTTCCTGATCTTCTGGTCTAAACGAACTAAATA
tt
ATATTAGTTTTTCTGTTTGGAACTTTAATTTTAGCCATGGCAGATTCCAAC

3546 3 57 0 71 ORF1a-NG1094R
GGCTACTAACAATGCCATGCAAGTTGAATCTGATGATTACATAGCTACTA
atg
GACCACTTAAAGTGGGTGGTAGTTGTGTTTTAAGCGGACACAATCTTGCTA

27694 6 30 0 70 ORF7a-FL101del
CTAAACTGTTCATCAGACAAGAGGAAGTTCAAGAACTTTACTCTCCAATT
ttt ctt
ATTGTTGCGGCAATAGTGTTTATAACACTTTGCTTCACACTCAAAAGAAAG

510 6 46 0 68 ORF1a-GH82del
ACAGCCCTATGTGTTCATCAAACGTTCGGATGCTCGAACTGCACCTCATG
gtc atg
TTATGGTTGAGCTGGTAGCAGAACTCGAAGGCATTCAGTACGGTCGTAGTG

27388 1 18 0 65
AGAATAAATATTCTCAATTAGATGAAGAGCAACCAATGGAGATTGATTAA
a
CGAACATGAAAATTATTCTTTTCTTGGCACTGATAACACTCGCTACTTGTG

21982 4 1 0 64 S-FL140fs
ACTAATGTTGTTATTAAAGTCTGTGAATTTCAATTTTGTAATGATCCATT
ttt g
GGTGTTTATTACCACAAAAACAACAAAAGTTGGATGGAAAGTGAGTTCAGA

26498 1 47 0 63
TAGAGTTCCTGATCTTCTGGTCTAAACGAACTAAATATTATATTAGTTTT
t
CTGTTTGGAACTTTAATTTTAGCCATGGCAGATTCCAACGGTACTATTACC

1606 6 2 0 62 ORF1a-DN448del
ATAGGTTGTAACCATACAGGTGTTGTTGGAGAAGGTTCCGAAGGTCTTAA
tga caa
CCTTCTTGAAATACTCCAAAAAGAGAAAGTCAACATCAATATTGTTGGTGA

27562 3 1 0 59 ORF7a-T57del
ACGAGGGCAATTCACCATTTCATCCTCTAGCTGATAACAAATTTGCACTG
act
TGCTTTAGCACTCAATTTGCTTTTGCTTGTCCTGACGGCGTAAAACACGTC

27568 5 1 0 59 ORF7a-FS59fs
GCAATTCACCATTTCATCCTCTAGCTGATAACAAATTTGCACTGACTTGC
ttt ag
CACTCAATTTGCTTTTGCTTGTCCTGACGGCGTAAAACACGTCTATCAGTT

28976 3 23 0 58 N-S235del
CTCTTGCTTTGCTGCTGCTTGACAGATTGAACCAGCTTGAGAGCAAAATG
tct
GGTAAAGGCCAACAACAACAAGGCCAAACTGTCACTAAGAAATCTGCTGCT

11283 3 16 0 57 ORF1a-SL3673M
TGCTAGTTGGGTGATGCGTATTATGACATGGTTGGATATGGTTGATACTA
gtt
TGTCTGGTTTTAAGCTAAAAGACTGTGTTATGTATGCATCAGCTGTAGTGT

27560 1 1 0 57 ORF7a-L56fs
ATACGAGGGCAATTCACCATTTCATCCTCTAGCTGATAACAAATTTGCAC
t
GACTTGCTTTAGCACTCAATTTGCTTTTGCTTGTCCTGACGGCGTAAAACA

28242 3 29 0 56 ORF8-V117del
TGCGTTGTTCGTTCTATGAAGACTTTTTAGAGTATCATGACGTTCGTGTT
gtt
TTAGATTTCATCTAAACGAACAAACTAAAATGTCTGATAATGGACCCCAAA

3333 3 10 0 55 ORF1a-IE1023K
TGAGGTTCAACCTCAATTAGAGATGGAACTTACACCAGTTGTTCAGACTA
ttg
AAGTGAATAGTTTTAGTGGTTATTTAAAACTTACTGACAATGTATACATTA

28219 2 8 0 55 ORF8-L109fs
ACCTAAATTGGGTAGTCTTGTAGTGCGTTGTTCGTTCTATGAAGACTTTT
ta
GAGTATCATGACGTTCGTGTTGTTTTAGATTTCATCTAAACGAACAAACTA

27624 1 6 0 55 ORF7a-L77fs
ACTCAATTTGCTTTTGCTTGTCCTGACGGCGTAAAACACGTCTATCAGTT
a
CGTGCCAGATCAGTTTCACCTAAACTGTTCATCAGACAAGAGGAAGTTCAA

27556 2 1 0 55 ORF7a-A55fs
GAACATACGAGGGCAATTCACCATTTCATCCTCTAGCTGATAACAAATTT
gc

ACTGACTTGCTTTAGCACTCAATTTGCTTTTGCTTGTCCTGACGGCGTAAA

29750 5 20 0 52
AGGGAGGACTTGAAAGAGCCACCACATTTTCACCGAGGCCACGCGGAGTA
cga tc
GAGTGTACAGTGAACAATGCTAGGGAGAGCTGCCTATATGGAAGAGCCCTA

27884 1 9 0 51 ORF7b-A43fs
ATCTTTTGGTTCTCACTTGAACTGCAAGATCATAATGAAACTTGTCACGC
c
TAAACGAACATGAAATTTCTTGTTTTCTTAGGAATCATCACAACTGTAGCT

29741 1 14 0 49
TGTAACATTAGGGAGGACTTGAAAGAGCCACCACATTTTCACCGAGGCCA
c
GCGGAGTACGATCGAGTGTACAGTGAACAATGCTAGGGAGAGCTGCCTATA

3335 6 10 0 49 ORF1a-EV1024del
AGGTTCAACCTCAATTAGAGATGGAACTTACACCAGTTGTTCAGACTATT
gaa gtg
AATAGTTTTAGTGGTTATTTAAAACTTACTGACAATGTATACATTAAAAAT

27796 1 18 0 48 ORF7b-L14fs
GAAAGACAGAATGATTGAACTTTCATTAATTGACTTCTATTTGTGCTTTT
t
AGCCTTTCTGCTATTCCTTGTTTTAATTATGCTTATTATCTTTTGGTTCTC

21994 6 18 0 48 S-YH145del
ATTAAAGTCTGTGAATTTCAATTTTGTAATGATCCATTTTTGGGTGTTTA
tta cca
CAAAAACAACAAAAGTTGGATGGAAAGTGAGTTCAGAGTTTATTCTAGTGC

6510 3 16 0 48 ORF1a-N2082del
TGTGAAAACTACCGAAGTTGTAGGAGACATTATACTTAAACCAGCAAATA
ata
GTTTAAAAATTACAGAAGAGGTTGGCCACACAGATCTAATGGCTGCTTATG

29762 6 7 0 48
AAAGAGCCACCACATTTTCACCGAGGCCACGCGGAGTACGATCGAGTGTA
cag tga
ACAATGCTAGGGAGAGCTGCCTATATGGAAGAGCCCTAATGTGTAAAATTA

426 3 33 0 47 ORF1a-V54del
GGTCTTATCAGAGGCACGTCAACATCTTAAAGATGGCACTTGTGGCTTAG
tag
AAGTTGAAAAAGGCGTTTTGCCTCAACTTGAACAGCCCTATGTGTTCATCA

27683 3 23 0 47 ORF7a-Y97del
ATCAGTTTCACCTAAACTGTTCATCAGACAAGAGGAAGTTCAAGAACTTT
act
CTCCAATTTTTCTTATTGTTGCGGCAATAGTGTTTATAACACTTTGCTTCA

28242 4 20 0 47 ORF8-VL117fs
TGCGTTGTTCGTTCTATGAAGACTTTTTAGAGTATCATGACGTTCGTGTT

gtt t
TAGATTTCATCTAAACGAACAAACTAAAATGTCTGATAATGGACCCCAAAA

25516 6 2 0 46 ORF3a-PF42del
CTTCAGATTTTGTTCGCGCTACTGCAACGATACCGATACAAGCCTCACTC
cct ttc
GGATGGCTTATTGTTGGCGTTGCACTTCTTGCTGTTTTTCAGAGCGCTTCC

25718 6 1 0 46 ORF3a-YA109del
ACACCTTTTGCTCGTTGCTGCTGGCCTTGAAGCCCCTTTTCTCTATCTTT
atg ctt
TAGTCTACTTCTTGCAGAGTATAAACTTTGTAAGAATAATAATGAGGCTTT

21765 3 43 0 45 S-IH68N
TCAGGACTTGTTCTTACCTTTCTTTTCCAATGTTACTTGGTTCCATGCTA
tac
ATGTCTCTGGGACCAATGGTACTAAGAGGTTTGATAACCCTGTCCTACCAT

669 3 29 0 45 ORF1a-SY135N
CCGCAAGGTTCTTCTTCGTAAGAACGGTAATAAAGGAGCTGGTGGCCATA
gtt
ACGGCGCCGATCTAAAGTCATTTGACTTAGGCGACGAGCTTGGCACTGATC

21990 6 26 0 45 S-VYY143D
TGTTATTAAAGTCTGTGAATTTCAATTTTGTAATGATCCATTTTTGGGTG
ttt att
ACCACAAAAACAACAAAAGTTGGATGGAAAGTGAGTTCAGAGTTTATTCTA

6813 3 6 0 43 ORF1a-T2183del
TAATTATATGCCTTATTTCTTTACTTTATTGCTACAATTGTGTACTTTTA
cta
GAAGTACAAATTCTAGAATTAAAGCATCTATGCCGACTACTATAGCAAAGA

28254 3 5 0 43 ORF8-I121del
TCTATGAAGACTTTTTAGAGTATCATGACGTTCGTGTTGTTTTAGATTTC
atc
TAAACGAACAAACTAAAATGTCTGATAATGGACCCCAAAATCAGCGAAATG

28219 1 14 0 40 ORF8-L109fs
ACCTAAATTGGGTAGTCTTGTAGTGCGTTGTTCGTTCTATGAAGACTTTT
t
AGAGTATCATGACGTTCGTGTTGTTTTAGATTTCATCTAAACGAACAAACT

28242 2 3 0 40 ORF8-V117fs
TGCGTTGTTCGTTCTATGAAGACTTTTTAGAGTATCATGACGTTCGTGTT
gt
TTTAGATTTCATCTAAACGAACAAACTAAAATGTCTGATAATGGACCCCAA

25714 6 8 0 39 ORF3a-LY108del
ACTCACACCTTTTGCTCGTTGCTGCTGGCCTTGAAGCCCCTTTTCTCTAT
ctt tat
GCTTTAGTCTACTTCTTGCAGAGTATAAACTTTGTAAGAATAATAATGAGG

241 2 12 0 38

TGCTTACGGTTTCGTCCGTGTTGCAGCCGATCATCAGCACATCTAGGTTT
cg
TCCGGGTGTGACCGAAAGGTAAGATGGAGAGCCTTGTCCCTGGTTTCAACG

27383 1 9 0 38 ORF6-D61fs
AACTGAGAATAAATATTCTCAATTAGATGAAGAGCAACCAATGGAGATTG
a
TTAAACGAACATGAAAATTATTCTTTTCTTGGCACTGATAACACTCGCTAC

29749 1 8 0 38
TAGGGAGGACTTGAAAGAGCCACCACATTTTCACCGAGGCCACGCGGAGT
a
CGATCGAGTGTACAGTGAACAATGCTAGGGAGAGCTGCCTATATGGAAGAG

29762 2 8 0 37
AAAGAGCCACCACATTTTCACCGAGGCCACGCGGAGTACGATCGAGTGTA
ca
GTGAACAATGCTAGGGAGAGCTGCCTATATGGAAGAGCCCTAATGTGTAAA

29752 3 8 0 36
GGAGGACTTGAAAGAGCCACCACATTTTCACCGAGGCCACGCGGAGTACG
atc
GAGTGTACAGTGAACAATGCTAGGGAGAGCTGCCTATATGGAAGAGCCCTA

27986 4 5 0 36 ORF8-YV31fs
GCATTTCACCAAGAATGTAGTTTACAGTCATGTACTCAACATCAACCATA
tgt a
GTTGATGACCCGTGTCCTATTCACTTCTATTCTAAATGGTATATTAGAGTA

241 1 27 0 35
TGCTTACGGTTTCGTCCGTGTTGCAGCCGATCATCAGCACATCTAGGTTT
c
GTCCGGGTGTGACCGAAAGGTAAGATGGAGAGCCTTGTCCCTGGTTTCAAC

1598 6 15 0 35 ORF1a-GL445del
GCGCTAACATAGGTTGTAACCATACAGGTGTTGTTGGAGAAGGTTCCGAA
ggt ctt
AATGACAACCTTCTTGAAATACTCCAAAAAGAGAAAGTCAACATCAATATT

21768 3 10 0 34 S-HV69L
GGACTTGTTCTTACCTTTCTTTTCCAATGTTACTTGGTTCCATGCTATAC
atg
TCTCTGGGACCAATGGTACTAAGAGGTTTGATAACCCTGTCCTACCATTTA

21808 3 1 0 34 S-V83del
CATGCTATACATGTCTCTGGGACCAATGGTACTAAGAGGTTTGATAACCC
tgt
CCTACCATTTAATGATGGTGTTTATTTTGCTTCCACTGAGAAGTCTAACAT

29742 6 5 0 33
GTAACATTAGGGAGGACTTGAAAGAGCCACCACATTTTCACCGAGGCCAC
gcg gag
TACGATCGAGTGTACAGTGAACAATGCTAGGGAGAGCTGCCTATATGGAAG

29734 1 3 0 33
ATCAGTGTGTAACATTAGGGAGGACTTGAAAGAGCCACCACATTTTCACC
g
AGGCCACGCGGAGTACGATCGAGTGTACAGTGAACAATGCTAGGGAGAGCT

28242 5 14 0 32 ORF8-VL117fs
TGCGTTGTTCGTTCTATGAAGACTTTTTAGAGTATCATGACGTTCGTGTT
gtt tt
AGATTTCATCTAAACGAACAAACTAAAATGTCTGATAATGGACCCCAAAAT

27759 3 11 0 32 ORF7a-*122fs
GCAATAGTGTTTATAACACTTTGCTTCACACTCAAAAGAAAGACAGAATG
att
GAACTTTCATTAATTGACTTCTATTTGTGCTTTTTAGCCTTTCTGCTATTC

27759 3 11 0 32 ORF7b-I2del
GCAATAGTGTTTATAACACTTTGCTTCACACTCAAAAGAAAGACAGAATG
att
GAACTTTCATTAATTGACTTCTATTTGTGCTTTTTAGCCTTTCTGCTATTC

28994 6 3 0 32 N-QQ241del
TTGACAGATTGAACCAGCTTGAGAGCAAAATGTCTGGTAAAGGCCAACAA
caa caa
GGCCAAACTGTCACTAAGAAATCTGCTGCTGAGGCTTCTAAGAAGCCTCGG

28901 3 2 0 32 N-M210del
GTTCAAGAAATTCAACTCCAGGCAGCAGTAGGGGAACTTCTCCTGCTAGA
atg
GCTGGCAATGGCGGTGATGCTGCTCTTGCTTTGCTGCTGCTTGACAGATTG

21769 5 2 0 32 S-HV69fs
GACTTGTTCTTACCTTTCTTTTCCAATGTTACTTGGTTCCATGCTATACA
tgt ct
CTGGGACCAATGGTACTAAGAGGTTTGATAACCCTGTCCTACCATTTAATG

29747 3 12 0 31
ATTAGGGAGGACTTGAAAGAGCCACCACATTTTCACCGAGGCCACGCGGA
gta
CGATCGAGTGTACAGTGAACAATGCTAGGGAGAGCTGCCTATATGGAAGAG

27267 1 5 0 31 ORF6-F22fs
GACTTTCAGGTTACTATAGCAGAGATATTACTAATTATTATGAGGACTTT
t
AAAGTTTCCATTTGGAATCTTGATTACATCATAAACCTCATAATTAAAAAT

29759 2 4 0 31
TTGAAAGAGCCACCACATTTTCACCGAGGCCACGCGGAGTACGATCGAGT
gt
ACAGTGAACAATGCTAGGGAGAGCTGCCTATATGGAAGAGCCCTAATGTGT

21774 1 7 0 30 S-S71fs
GTTCTTACCTTTCTTTTCCAATGTTACTTGGTTCCATGCTATACATGTCT
c
TGGGACCAATGGTACTAAGAGGTTTGATAACCCTGTCCTACCATTTAATGA

21778 1 7 0 30 S-G72fs
TTACCTTTCTTTTCCAATGTTACTTGGTTCCATGCTATACATGTCTCTGG
g
ACCAATGGTACTAAGAGGTTTGATAACCCTGTCCTACCATTTAATGATGGT

515 1 5 0 30 ORF1a-V84fs
CCTATGTGTTCATCAAACGTTCGGATGCTCGAACTGCACCTCATGGTCAT
g
TTATGGTTGAGCTGGTAGCAGAACTCGAAGGCATTCAGTACGGTCGTAGTG

27577 1 4 0 30 ORF7a-Q62fs
CATTTCATCCTCTAGCTGATAACAAATTTGCACTGACTTGCTTTAGCACT
c
AATTTGCTTTTGCTTGTCCTGACGGCGTAAAACACGTCTATCAGTTACGTG

27267 2 3 0 30 ORF6-F22fs
GACTTTCAGGTTACTATAGCAGAGATATTACTAATTATTATGAGGACTTT
ta
AAGTTTCCATTTGGAATCTTGATTACATCATAAACCTCATAATTAAAAATT

23480 3 2 0 30 S-S640del
CTATTCATGCAGATCAACTTACTCCTACTTGGCGTGTTTATTCTACAGGT
tct
AATGTTTTTCAAACACGTGCAGGCTGTTTAATAGGGGCTGAACATGTCAAC

21768 4 14 0 29 S-HV69fs
GGACTTGTTCTTACCTTTCTTTTCCAATGTTACTTGGTTCCATGCTATAC
atg t
CTCTGGGACCAATGGTACTAAGAGGTTTGATAACCCTGTCCTACCATTTAA

29750 2 12 0 29
AGGGAGGACTTGAAAGAGCCACCACATTTTCACCGAGGCCACGCGGAGTA
cg
ATCGAGTGTACAGTGAACAATGCTAGGGAGAGCTGCCTATATGGAAGAGCC

4881 3 14 0 28 ORF1a-S1539del
ACTAGGTATAGAATTTCTTAAGAGAGGTGATAAAAGTGTATATTACACTA
gta
ATCCTACCACATTCCACCTAGATGGTGAAGTTATCACCTTTGACAATCTTA

199 1 3 0 27
GTCGTTGACAGGACACGAGTAACTCGTCTATCTTCTGCAGGCTGCTTACG
g
TTTCGTCCGTGTTGCAGCCGATCATCAGCACATCTAGGTTTCGTCCGGGTG

29764 4 2 0 27
AGAGCCACCACATTTTCACCGAGGCCACGCGGAGTACGATCGAGTGTACA
gtg a
ACAATGCTAGGGAGAGCTGCCTATATGGAAGAGCCCTAATGTGTAAAATTA

29755 1 12 0 26
GGACTTGAAAGAGCCACCACATTTTCACCGAGGCCACGCGGAGTACGATC
g

AGTGTACAGTGAACAATGCTAGGGAGAGCTGCCTATATGGAAGAGCCCTAA

27704 3 11 0 26 ORF7a-V104del
CATCAGACAAGAGGAAGTTCAAGAACTTTACTCTCCAATTTTTCTTATTG
ttg
CGGCAATAGTGTTTATAACACTTTGCTTCACACTCAAAAGAAAGACAGAAT

27555 1 6 0 26 ORF7a-F54fs
GGAACATACGAGGGCAATTCACCATTTCATCCTCTAGCTGATAACAAATT
t
GCACTGACTTGCTTTAGCACTCAATTTGCTTTTGCTTGTCCTGACGGCGTA

27870 5 1 0 26 ORF7b-ET39fs
TAATTATGCTTATTATCTTTTGGTTCTCACTTGAACTGCAAGATCATAAT
gaa ac
TTGTCACGCCTAAACGAACATGAAATTTCTTGTTTTCTTAGGAATCATCAC

29868 1 20 0 25
AATTTTAGTAGTGCTATCCCCATGTGATTTTAATAGCTTCTTAGGAGAAT
g
ACAAAAAAAAAAAAAAAAAAAAAAAAAAAAAAAAAA

29838 1 15 0 25
CTATATGGAAGAGCCCTAATGTGTAAAATTAATTTTAGTAGTGCTATCCC
c
ATGTGATTTTAATAGCTTCTTAGGAGAATGACAAAAAAAAAAAAAAAAAAA

29724 2 15 0 25
GCAATCTTTAATCAGTGTGTAACATTAGGGAGGACTTGAAAGAGCCACCA
ca
TTTTCACCGAGGCCACGCGGAGTACGATCGAGTGTACAGTGAACAATGCTA

28892 6 14 0 25 N-PA207del
GTCGCAACAGTTCAAGAAATTCAACTCCAGGCAGCAGTAGGGGAACTTCT
cct gct
AGAATGGCTGGCAATGGCGGTGATGCTGCTCTTGCTTTGCTGCTGCTTGAC

9852 3 3 0 25 ORF1a-D3196del
GCTGTGCACCTTTTTGTTAAATAAAGAAATGTATCTAAAGTTGCGTAGTG
atg
TGCTATTACCTCTTACGCAATATAATAGATACTTAGCTCTTTATAATAAGT

1650 3 2 0 25 ORF1a-N462del
TCTTAATGACAACCTTCTTGAAATACTCCAAAAAGAGAAAGTCAACATCA
ata
TTGTTGGTGACTTTAAACTTAATGAAGAGATCGCCATTATTTTGGCATCTT

21765 1 1 0 25 S-I68fs
TCAGGACTTGTTCTTACCTTTCTTTTCCAATGTTACTTGGTTCCATGCTA
t
ACATGTCTCTGGGACCAATGGTACTAAGAGGTTTGATAACCCTGTCCTACC

29781 1 21 0 23
ACCGAGGCCACGCGGAGTACGATCGAGTGTACAGTGAACAATGCTAGGGA

g
AGCTGCCTATATGGAAGAGCCCTAATGTGTAAAATTAATTTTAGTAGTGCT

18898 3 17 0 23 ORF1b-V1811del
CTAGTTGTGATGCAATCATGACTAGGTGTCTAGCTGTCCACGAGTGCTTT
gtt
AAGCGTGTTGACTGGACTATTGAATATCCTATAATTGGTGATGAACTGAAG

29760 2 11 0 23
TGAAAGAGCCACCACATTTTCACCGAGGCCACGCGGAGTACGATCGAGTG
ta
CAGTGAACAATGCTAGGGAGAGCTGCCTATATGGAAGAGCCCTAATGTGTA

27700 6 7 0 23 ORF7a-IV103del
TGTTCATCAGACAAGAGGAAGTTCAAGAACTTTACTCTCCAATTTTTCTT
att gtt
GCGGCAATAGTGTTTATAACACTTTGCTTCACACTCAAAAGAAAGACAGAA

28093 4 6 0 23 ORF8-SK67fs
AGCTAGAAAATCAGCACCTTTAATTGAATTGTGCGTGGATGAGGCTGGTT
cta a
ATCACCCATTCAGTACATCGATATCGGTAATTATACAGTTTCCTGTTTACC

21583 3 5 0 23 S-L8del
GTGATGTTCTTGTTAACAACTAAACGAACAATGTTTGTTTTTCTTGTTTT
att
GCCACTAGTCTCTAGTCAGTGTGTTAATCTTACAACCAGAACTCAATTACC

28248 1 14 0 22 ORF8-D119fs
GTTCGTTCTATGAAGACTTTTTAGAGTATCATGACGTTCGTGTTGTTTTA
g
ATTTCATCTAAACGAACAAACTAAAATGTCTGATAATGGACCCCAAAATCA

8586 3 12 0 22 ORF1a-I2774del
GATAGCACTTAAGGGTGGTAAAATTGTTAATAATTGGTTGAAGCAGTTAA
tta
AAGTTACACTTGTGTTCCTTTTTGTTGCTGCTATTTTCTATTTAATAACAC

6519 6 11 0 22 ORF1a-KI2085del
TACCGAAGTTGTAGGAGACATTATACTTAAACCAGCAAATAATAGTTTAA
aaa tta
CAGAAGAGGTTGGCCACACAGATCTAATGGCTGCTTATGTAGACAATTCTA

21773 1 9 0 22 S-S71fs
TGTTCTTACCTTTCTTTTCCAATGTTACTTGGTTCCATGCTATACATGTC
t
CTGGGACCAATGGTACTAAGAGGTTTGATAACCCTGTCCTACCATTTAATG

29792 2 6 0 22
GCGGAGTACGATCGAGTGTACAGTGAACAATGCTAGGGAGAGCTGCCTAT
at
GGAAGAGCCCTAATGTGTAAAATTAATTTTAGTAGTGCTATCCCCATGTGA

28089 5 6 0 22 ORF8-GS66fs

TAGGAGCTAGAAAATCAGCACCTTTAATTGAATTGTGCGTGGATGAGGCT
ggt tc
TAAATCACCCATTCAGTACATCGATATCGGTAATTATACAGTTTCCTGTTT

27987 2 6 0 22 ORF8-V32fs
CATTTCACCAAGAATGTAGTTTACAGTCATGTACTCAACATCAACCATAT
gt
AGTTGATGACCCGTGTCCTATTCACTTCTATTCTAAATGGTATATTAGAGT

28899 3 3 0 22 N-R209del
CAGTTCAAGAAATTCAACTCCAGGCAGCAGTAGGGGAACTTCTCCTGCTA
gaa
TGGCTGGCAATGGCGGTGATGCTGCTCTTGCTTTGCTGCTGCTTGACAGAT

28069 2 1 0 22 ORF8-E59fs
TTCTAAATGGTATATTAGAGTAGGAGCTAGAAAATCAGCACCTTTAATTG
aa
TTGTGCGTGGATGAGGCTGGTTCTAAATCACCCATTCAGTACATCGATATC

28073 3 1 0 22 ORF8-LC60F
AAATGGTATATTAGAGTAGGAGCTAGAAAATCAGCACCTTTAATTGAATT
gtg
CGTGGATGAGGCTGGTTCTAAATCACCCATTCAGTACATCGATATCGGTAA

315 3 15 0 21 ORF1a-SL17M
GATGGAGAGCCTTGTCCCTGGTTTCAACGAGAAAACACACGTCCAACTCA
gtt
TGCCTGTTTTACAGGTTCGCGACGTGCTCGTACGTGGCTTTGGAGACTCCG

29834 1 6 0 21
CTGCCTATATGGAAGAGCCCTAATGTGTAAAATTAATTTTAGTAGTGCTA
t
CCCCATGTGATTTTAATAGCTTCTTAGGAGAATGACAAAAAAAAAAAAAA

26488 5 6 0 21
TGAATTCTTCTAGAGTTCCTGATCTTCTGGTCTAAACGAACTAAATATTA
tat ta
GTTTTTCTGTTTGGAACTTTAATTTTAGCCATGGCAGATTCCAACGGTACT

25704 4 4 0 21 ORF3a-PF104fs
GTAACAGTTTACTCACACCTTTTGCTCGTTGCTGCTGGCCTTGAAGCCCC
ttt t
CTCTATCTTTATGCTTTAGTCTACTTCTTGCAGAGTATAAACTTTGTAAGA

27702 1 1 0 21 ORF7a-I103fs
TTCATCAGACAAGAGGAAGTTCAAGAACTTTACTCTCCAATTTTTCTTAT
t
GTTGCGGCAATAGTGTTTATAACACTTTGCTTCACACTCAAAAGAAAGACA

28025 1 19 0 20 ORF8-K44fs
CATCAACCATATGTAGTTGATGACCCGTGTCCTATTCACTTCTATTCTAA
a
TGGTATATTAGAGTAGGAGCTAGAAAATCAGCACCTTTAATTGAATTGTGC

28093 2 3 0 20 ORF8-S67fs
AGCTAGAAAATCAGCACCTTTAATTGAATTGTGCGTGGATGAGGCTGGTT
ct
AAATCACCCATTCAGTACATCGATATCGGTAATTATACAGTTTCCTGTTTA

25710 5 3 0 20 ORF3a-LY106fs
GTTTACTCACACCTTTTGCTCGTTGCTGCTGGCCTTGAAGCCCCTTTTCT
cta tc
TTTATGCTTTAGTCTACTTCTTGCAGAGTATAAACTTTGTAAGAATAATAA

26224 2 2 0 20
AACCAATTTATGATGAACCGACGACGACTACTAGCGTGCCTTTGTAAGCA
ca
AGCTGATGAGTACGAACTTATGTACTCATTCGTTTCGGAAGAGACAGGTAC

1432 3 16 0 19 ORF1a-NE389K
TGTCACAATTCAGAAGTAGGACCTGAGCATAGTCTTGCCGAATACCATAA
tga
ATCTGGCTTGAAAACCATTCTTCGTAAGGGTGGTCGCACTATTGCCTTTGG

28069 5 13 0 19 ORF8-EL59fs
TTCTAAATGGTATATTAGAGTAGGAGCTAGAAAATCAGCACCTTTAATTG
aat tg
TGCGTGGATGAGGCTGGTTCTAAATCACCCATTCAGTACATCGATATCGGT

26466 3 10 0 19 E-V75del
GTTTACTCTCGTGTTAAAAATCTGAATTCTTCTAGAGTTCCTGATCTTCT
ggt
CTAAACGAACTAAATATTATATTAGTTTTTCTGTTTGGAACTTTAATTTTA

29729 2 9 0 19
CTTTAATCAGTGTGTAACATTAGGGAGGACTTGAAAGAGCCACCACATTT
tc
ACCGAGGCCACGCGGAGTACGATCGAGTGTACAGTGAACAATGCTAGGGAG

22294 3 8 0 19 S-LH244F
GATTTGCCAATAGGTATTAACATCACTAGGTTTCAAACTTTACTTGCTTT
aca
TAGAAGTTATTTGACTCCTGGTGATTCTTCTTCAGGTTGGACAGCTGGTGC

29749 5 6 0 19
TAGGGAGGACTTGAAAGAGCCACCACATTTTCACCGAGGCCACGCGGAGT
acg at
CGAGTGTACAGTGAACAATGCTAGGGAGAGCTGCCTATATGGAAGAGCCCT

27882 3 6 0 19 ORF7b-A43del
TTATCTTTTGGTTCTCACTTGAACTGCAAGATCATAATGAAACTTGTCAC
gcc
TAAACGAACATGAAATTTCTTGTTTTCTTAGGAATCATCACAACTGTAGCT

28089 6 4 0 19 ORF8-GS66del
TAGGAGCTAGAAAATCAGCACCTTTAATTGAATTGTGCGTGGATGAGGCT
ggt tct
AAATCACCCATTCAGTACATCGATATCGGTAATTATACAGTTTCCTGTTTA

26487 3 3 0 19
CTGAATTCTTCTAGAGTTCCTGATCTTCTGGTCTAAACGAACTAAATATT
ata
TTAGTTTTTCTGTTTGGAACTTTAATTTTAGCCATGGCAGATTCCAACGGT

22344 3 1 0 19 S-G261del
ACATAGAAGTTATTTGACTCCTGGTGATTCTTCTTCAGGTTGGACAGCTG
gtg
CTGCAGCTTATTATGTGGGTTATCTTCAACCTAGGACTTTTCTATTAAAAT

27722 3 14 0 18 ORF7a-I110del
TCAAGAACTTTACTCTCCAATTTTTCTTATTGTTGCGGCAATAGTGTTTA
taa
CACTTTGCTTCACACTCAAAAGAAAGACAGAATGATTGAACTTTCATTAAT

512 6 13 0 18 ORF1a-HV83del
AGCCCTATGTGTTCATCAAACGTTCGGATGCTCGAACTGCACCTCATGGT
cat gtt
ATGGTTGAGCTGGTAGCAGAACTCGAAGGCATTCAGTACGGTCGTAGTGGT

27621 3 10 0 18 ORF7a-L77del
AGCACTCAATTTGCTTTTGCTTGTCCTGACGGCGTAAAACACGTCTATCA
gtt
ACGTGCCAGATCAGTTTCACCTAAACTGTTCATCAGACAAGAGGAAGTTCA

29749 2 8 0 18
TAGGGAGGACTTGAAAGAGCCACCACATTTTCACCGAGGCCACGCGGAGT
ac
GATCGAGTGTACAGTGAACAATGCTAGGGAGAGCTGCCTATATGGAAGAGC

20409 6 5 0 18 ORF1b-FE2314del
GGTTTACATCTACTGATTGGACTAGCTAAACGTTTTAAGGAATCACCTTT
tga att
AGAAGATTTTATTCCTATGGACAGTACAGTTAAAAACTATTTCATAACAGA

28769 3 4 0 18 N-T166del
GCAATCCTGCTAACAATGCTGCAATCGTGCTACAACTTCCTCAAGGAACA
aca
TTGCCAAAAGGCTTCTACGCAGAAGGGAGCAGAGGCGGCAGTCAAGCCTCT

21768 5 2 0 18 S-HV69fs
GGACTTGTTCTTACCTTTCTTTTCCAATGTTACTTGGTTCCATGCTATAC
atg tc
TCTGGGACCAATGGTACTAAGAGGTTTGATAACCCTGTCCTACCATTTAAT

29819 6 2 0 18
CAATGCTAGGGAGAGCTGCCTATATGGAAGAGCCCTAATGTGTAAAATTA
att tta
GTAGTGCTATCCCCATGTGATTTTAATAGCTTCTTAGGAGAATGACAAAAA

29584 1 12 0 17 ORF10-F9fs
ACTCATGCAGACCACACAAGGCAGATGGGCTATATAAACGTTTTCGCTTT
t

CCGTTTACGATATATAGTCTACTCTTGTGCAGAATGAATTCTCGTAACTAC

28252 2 11 0 17 ORF8-F120fs
GTTCTATGAAGACTTTTTAGAGTATCATGACGTTCGTGTTGTTTTAGATT
tc
ATCTAAACGAACAAACTAAAATGTCTGATAATGGACCCCAAAATCAGCGAA

29750 1 9 0 17
AGGGAGGACTTGAAAGAGCCACCACATTTTCACCGAGGCCACGCGGAGTA
c
GATCGAGTGTACAGTGAACAATGCTAGGGAGAGCTGCCTATATGGAAGAGC

29730 1 7 0 17
TTTAATCAGTGTGTAACATTAGGGAGGACTTGAAAGAGCCACCACATTTT
c
ACCGAGGCCACGCGGAGTACGATCGAGTGTACAGTGAACAATGCTAGGGAG

11083 1 7 0 17 ORF1a-L3606fs
TCACTTTTAGTTTTAGTCCAGAGTACTCAATGGTCTTTGTTCTTTTTTTT
g
TATGAAAATGCCTTTTTACCTTTTGCTATGGGTATTATTGCTATGTCTGCT

27618 6 7 0 17 ORF7a-YQL75*
TTTAGCACTCAATTTGCTTTTGCTTGTCCTGACGGCGTAAAACACGTCTA
tca gtt
ACGTGCCAGATCAGTTTCACCTAAACTGTTCATCAGACAAGAGGAAGTTCA

29756 2 5 0 17
GACTTGAAAGAGCCACCACATTTTCACCGAGGCCACGCGGAGTACGATCG
ag
TGTACAGTGAACAATGCTAGGGAGAGCTGCCTATATGGAAGAGCCCTAATG

27762 1 2 0 17 ORF7b-E3fs
ATAGTGTTTATAACACTTTGCTTCACACTCAAAAGAAAGACAGAATGATT
g
AACTTTCATTAATTGACTTCTATTTGTGCTTTTTAGCCTTTCTGCTATTCC

27618 2 2 0 17 ORF7a-Y75fs
TTTAGCACTCAATTTGCTTTTGCTTGTCCTGACGGCGTAAAACACGTCTA
tc
AGTTACGTGCCAGATCAGTTTCACCTAAACTGTTCATCAGACAAGAGGAAG

29784 1 12 0 16
GAGGCCACGCGGAGTACGATCGAGTGTACAGTGAACAATGCTAGGGAGAG
c
TGCCTATATGGAAGAGCCCTAATGTGTAAAATTAATTTTAGTAGTGCTATC

29788 1 12 0 16
CCACGCGGAGTACGATCGAGTGTACAGTGAACAATGCTAGGGAGAGCTGC
c
TATATGGAAGAGCCCTAATGTGTAAAATTAATTTTAGTAGTGCTATCCCCA

27207 1 9 0 16 ORF6-F2fs
GTAGCAGTGACAATATTGCTTTGCTTGTACAGTAAGTGACAACAGATGTT

t
CATCTCGTTGACTTTCAGGTTACTATAGCAGAGATATTACTAATTATTATG

29848 1 15 0 15
GAGCCCTAATGTGTAAAATTAATTTTAGTAGTGCTATCCCCATGTGATTT
t
AATAGCTTCTTAGGAGAATGACAAAAAAAAAAAAAAAAAAAAAAAAAAAA

6686 3 12 0 15 ORF1a-Y2141del
CTCATGGTTTAGCTGCTGTTAATAGTGTCCCTTGGGATACTATAGCTAAT
tat
GCTAAGCCTTTTCTTAACAAAGTTGTTAGTACAACTACTAACATAGTTACA

20425 3 10 0 15 ORF1b-I2320del
TTGGACTAGCTAAACGTTTTAAGGAATCACCTTTTGAATTAGAAGATTTT
att
CCTATGGACAGTACAGTTAAAAACTATTTCATAACAGATGCGCAAACAGGT

29747 1 9 0 15
ATTAGGGAGGACTTGAAAGAGCCACCACATTTTCACCGAGGCCACGCGGA
g
TACGATCGAGTGTACAGTGAACAATGCTAGGGAGAGCTGCCTATATGGAAG

8567 3 7 0 15 ORF1a-N2768del
TTAATGTTGTAACAACAAAGATAGCACTTAAGGGTGGTAAAATTGTTAAT
aat
TGGTTGAAGCAGTTAATTAAAGTTACACTTGTGTTCCTTTTTGTTGCTGCT

27578 4 7 0 15 ORF7a-QF62fs
ATTTCATCCTCTAGCTGATAACAAATTTGCACTGACTTGCTTTAGCACTC
aat t
TGCTTTTGCTTGTCCTGACGGCGTAAAACACGTCTATCAGTTACGTGCCAG

28247 5 7 0 15 ORF8-LD118fs
TGTTCGTTCTATGAAGACTTTTTAGAGTATCATGACGTTCGTGTTGTTTT
aga tt
TCATCTAAACGAACAAACTAAAATGTCTGATAATGGACCCCAAAATCAGCG

27317 1 6 0 15 ORF6-N39fs
TAAAGTTTCCATTTGGAATCTTGATTACATCATAAACCTCATAATTAAAA
a
TTTATCTAAGTCACTAACTGAGAATAAATATTCTCAATTAGATGAAGAGCA

28877 2 6 0 15 N-S202fs
GTTCCTCATCACGTAGTCGCAACAGTTCAAGAAATTCAACTCCAGGCAGC
ag
TAGGGGAACTTCTCCTGCTAGAATGGCTGGCAATGGCGGTGATGCTGCTCT

1585 6 4 0 15 ORF1a-EG441del
GTTCCACGTGCTAGCGCTAACATAGGTTGTAACCATACAGGTGTTGTTGG
aga agg
TTCCGAAGGTCTTAATGACAACCTTCTTGAAATACTCCAAAAAGAGAAAGT

25517 1 4 0 15 ORF3a-P42fs

TTCAGATTTTGTTCGCGCTACTGCAACGATACCGATACAAGCCTCACTCC
c
TTTCGGATGGCTTATTGTTGGCGTTGCACTTCTTGCTGTTTTTCAGAGCGC

23598 3 3 0 15 S-NS679T
TGACATACCCATTGGTGCAGGTATATGCGCTAGTTATCAGACTCAGACTA
att
CTCCTCGGCGGGCACGTAGTGTAGCTAGTCAATCCATCATTGCCTACACTA

29578 6 3 0 15 ORF10-AF8del
GCCTAAACTCATGCAGACCACACAAGGCAGATGGGCTATATAAACGTTTT
cgc ttt
TCCGTTTACGATATATAGTCTACTCTTGTGCAGAATGAATTCTCGTAACTA

29743 6 3 0 15
TAACATTAGGGAGGACTTGAAAGAGCCACCACATTTTCACCGAGGCCACG
cgg agt
ACGATCGAGTGTACAGTGAACAATGCTAGGGAGAGCTGCCTATATGGAAGA

27683 6 1 0 15 ORF7a-YSP97S
ATCAGTTTCACCTAAACTGTTCATCAGACAAGAGGAAGTTCAAGAACTTT
act ctc
CAATTTTTCTTATTGTTGCGGCAATAGTGTTTATAACACTTTGCTTCACAC

29762 5 9 0 14
AAAGAGCCACCACATTTTCACCGAGGCCACGCGGAGTACGATCGAGTGTA
cag tg
AACAATGCTAGGGAGAGCTGCCTATATGGAAGAGCCCTAATGTGTAAAATT

21764 6 8 0 14 S-IH68del
CTCAGGACTTGTTCTTACCTTTCTTTTCCAATGTTACTTGGTTCCATGCT
ata cat
GTCTCTGGGACCAATGGTACTAAGAGGTTTGATAACCCTGTCCTACCATTT

22161 3 8 0 14 S-Y200del
GGGTAATTTCAAAAATCTTAGGGAATTTGTGTTTAAGAATATTGATGGTT
att
TTAAAATATATTCTAAGCACACGCCTATTAATTTAGTGCGTGATCTCCCTC

23481 3 7 0 14 S-SN640Y
TATTCATGCAGATCAACTTACTCCTACTTGGCGTGTTTATTCTACAGGTT
cta
ATGTTTTTCAAACACGTGCAGGCTGTTTAATAGGGGCTGAACATGTCAACA

25996 3 6 0 14 ORF3a-V202del
AGATTGGTGGTTATACTGAAAAATGGGAATCTGGAGTAAAAGACTGTGTT
gta
TTACACAGTTACTTCACTTCAGACTATTACCAGCTGTACTCAACTCAATTG

7059 3 6 0 14 ORF1a-GY2265D
TGCTTTAGGTGTTTTAATGTCTAATTTAGGCATGCCTTCTTACTGTACTG
gtt
ACAGAGAAGGCTATTTGAACTCTACTAATGTCACTATTGCAACCTACTGTA

21768 2 4 0 14 S-H69fs
GGACTTGTTCTTACCTTTCTTTTCCAATGTTACTTGGTTCCATGCTATAC
at
GTCTCTGGGACCAATGGTACTAAGAGGTTTGATAACCCTGTCCTACCATTT

21771 3 4 0 14 S-VS70A
CTTGTTCTTACCTTTCTTTTCCAATGTTACTTGGTTCCATGCTATACATG
tct
CTGGGACCAATGGTACTAAGAGGTTTGATAACCCTGTCCTACCATTTAATG

12627 3 1 0 14 ORF1a-NL4121I
AGATAGTAAAATTGTTCAACTTAGTGAAATTAGTATGGACAATTCACCTA
att
TAGCATGGCCTCTTATTGTAACAGCTTTAAGGGCCAATTCTGCTGTCAAAT

29745 5 1 0 14
ACATTAGGGAGGACTTGAAAGAGCCACCACATTTTCACCGAGGCCACGCG
gag ta
CGATCGAGTGTACAGTGAACAATGCTAGGGAGAGCTGCCTATATGGAAGAG

27909 6 1 0 14 ORF8-FL6del
AAGATCATAATGAAACTTGTCACGCCTAAACGAACATGAAATTTCTTGTT
ttc tta
GGAATCATCACAACTGTAGCTGCATTTCACCAAGAATGTAGTTTACAGTCA

29773 1 12 0 13
ACATTTTCACCGAGGCCACGCGGAGTACGATCGAGTGTACAGTGAACAAT
g
CTAGGGAGAGCTGCCTATATGGAAGAGCCCTAATGTGTAAAATTAATTTTA

21547 2 11 0 13 ORF1b-N2694fs
TTATAATTAGAGAAAACAACAGAGTTGTTATTTCTAGTGATGTTCTTGTT
aa
CAACTAAACGAACAATGTTTGTTTTTCTTGTTTTATTGCCACTAGTCTCTA

29780 1 10 0 13
CACCGAGGCCACGCGGAGTACGATCGAGTGTACAGTGAACAATGCTAGGG
a
GAGCTGCCTATATGGAAGAGCCCTAATGTGTAAAATTAATTTTAGTAGTGC

203 1 8 0 13
TTGACAGGACACGAGTAACTCGTCTATCTTCTGCAGGCTGCTTACGGTTT
c
GTCCGTGTTGCAGCCGATCATCAGCACATCTAGGTTTCGTCCGGGTGTGAC

29734 5 8 0 13
ATCAGTGTGTAACATTAGGGAGGACTTGAAAGAGCCACCACATTTTCACC
gag gc
CACGCGGAGTACGATCGAGTGTACAGTGAACAATGCTAGGGAGAGCTGCCT

29722 3 5 0 13
TAGCAATCTTTAATCAGTGTGTAACATTAGGGAGGACTTGAAAGAGCCAC
cac
ATTTTCACCGAGGCCACGCGGAGTACGATCGAGTGTACAGTGAACAATGCT

26096   6   4   0   13 ORF3a-KIV235I
ATTGAGTACAGACACTGGTGTTGAACATGTTACCTTCTTCATCTACAATA
aaa ttg
TTGATGAGCCTGAAGAACATGTCCAAATTCACACAATCGACGGTTCATCCG

21766   5   4   0   13 S-IH68fs
CAGGACTTGTTCTTACCTTTCTTTTCCAATGTTACTTGGTTCCATGCTAT
aca tg
TCTCTGGGACCAATGGTACTAAGAGGTTTGATAACCCTGTCCTACCATTTA

29754   3   3   0   13
AGGACTTGAAAGAGCCACCACATTTTCACCGAGGCCACGCGGAGTACGAT
cga
GTGTACAGTGAACAATGCTAGGGAGAGCTGCCTATATGGAAGAGCCCTAAT

6321   3   3   0   13 ORF1a-V2019del
GTATAAACCAAATACCTGGTGTATACGTTGTCTTTGGAGCACAAAACCAG
ttg
AAACATCAAATTCGTTTGATGTACTGAAGTCAGAGGACGCGCAGGGAATGG

6966   1   1   0   13 ORF1a-L2234fs
TTTGAAGTCACCTAATTTTTCTAAACTGATAAATATTATAATTTGGTTTT
t
ACTATTAAGTGTTTGCCTAGGTTCTTTAATCTACTCAACCGCTGCTTTAGG

27579   1   9   0   12 ORF7a-Q62fs
TTTCATCCTCTAGCTGATAACAAATTTGCACTGACTTGCTTTAGCACTCA
a
TTTGCTTTTGCTTGTCCTGACGGCGTAAAACACGTCTATCAGTTACGTGCC

29684   1   8   0   12
ACATAGCACAAGTAGATGTAGTTAACTTTAATCTCACATAGCAATCTTTA
a
TCAGTGTGTAACATTAGGGAGGACTTGAAAGAGCCACCACATTTTCACCGA

29754   4   8   0   12
AGGACTTGAAAGAGCCACCACATTTTCACCGAGGCCACGCGGAGTACGAT
cga g
TGTACAGTGAACAATGCTAGGGAGAGCTGCCTATATGGAAGAGCCCTAATG

28881   4   8   0   12 N-RG203fs
CTCATCACGTAGTCGCAACAGTTCAAGAAATTCAACTCCAGGCAGCAGTA
ggg g
AACTTCTCCTGCTAGAATGGCTGGCAATGGCGGTGATGCTGCTCTTGCTTT

27696   4   7   0   12 ORF7a-FL101fs
AAACTGTTCATCAGACAAGAGGAAGTTCAAGAACTTTACTCTCCAATTTT
tct t
ATTGTTGCGGCAATAGTGTTTATAACACTTTGCTTCACACTCAAAAGAAAG

27612   1   4   0   12 ORF7a-H73fs
ACTTGCTTTAGCACTCAATTTGCTTTTGCTTGTCCTGACGGCGTAAAACA
c

GTCTATCAGTTACGTGCCAGATCAGTTTCACCTAAACTGTTCATCAGACAA

137 3 4 0 12
TGTGGCTGTCACTCGGCTGCATGCTTAGTGCACTCACGCAGTATAATTAA
taa
CTAATTACTGTCGTTGACAGGACACGAGTAACTCGTCTATCTTCTGCAGGC

29406 3 3 0 12 N-ET378A
AACATTCCCACCAACAGAGCCTAAAAAGGACAAAAAGAAGAAGGCTGATG
aaa
CTCAAGCCTTACCGCAGAGACAGAAGAAACAGCAAACTGTGACTCTTCTTC

25438 3 3 0 12 ORF3a-K16del
AACTTATGGATTTGTTTATGAGAATCTTCACAATTGGAACTGTAACTTTG
aag
CAAGGTGAAATCAAGGATGCTACTCCTTCAGATTTTGTTCGCGCTACTGCA

27746 1 3 0 12 ORF7a-R118fs
TCTTATTGTTGCGGCAATAGTGTTTATAACACTTTGCTTCACACTCAAAA
g
AAAGACAGAATGATTGAACTTTCATTAATTGACTTCTATTTGTGCTTTTTA

28086 2 3 0 12 ORF8-A65fs
GAGTAGGAGCTAGAAAATCAGCACCTTTAATTGAATTGTGCGTGGATGAG
gc
TGGTTCTAAATCACCCATTCAGTACATCGATATCGGTAATTATACAGTTTC

6503 3 3 0 12 ORF1a-A2080del
AGTGTAATGTGAAAACTACCGAAGTTGTAGGAGACATTATACTTAAACCA
gca
AATAATAGTTTAAAAATTACAGAAGAGGTTGGCCACACAGATCTAATGGCT

29753 3 3 0 12
GAGGACTTGAAAGAGCCACCACATTTTCACCGAGGCCACGCGGAGTACGA
tcg
AGTGTACAGTGAACAATGCTAGGGAGAGCTGCCTATATGGAAGAGCCCTAA

28869 1 2 0 12 N-P199fs
CTCTTCTCGTTCCTCATCACGTAGTCGCAACAGTTCAAGAAATTCAACTC
c
AGGCAGCAGTAGGGGAACTTCTCCTGCTAGAATGGCTGGCAATGGCGGTGA

16168 3 2 0 12 ORF1b-D901del
ATGAGTTAACAGGACACATGTTAGACATGTATTCTGTTATGCTTACTAAT
gat
AACACTTCAAGGTATTGGGAACCTGAGTTTTATGAGGCTATGTACACACCG

28133 1 1 0 12 ORF8-T80fs
GAGGCTGGTTCTAAATCACCCATTCAGTACATCGATATCGGTAATTATAC
a
GTTTCCTGTTTACCTTTTACAATTAATTGCCAGGAACCTAAATTGGGTAGT

3221 3 1 0 12 ORF1a-Q986del
TTCAACCTGAAGAAGAGCAAGAAGAAGATTGGTTAGATGATGATAGTCAA

caa
ACTGTTGGTCAACAAGACGGCAGTGAGGACAATCAGACAACTACTATTCAA

22286 3 9 0 11 S-L242del
CATTGGTAGATTTGCCAATAGGTATTAACATCACTAGGTTTCAAACTTTA
ctt
GCTTTACATAGAAGTTATTTGACTCCTGGTGATTCTTCTTCAGGTTGGACA

29753 1 9 0 11
GAGGACTTGAAAGAGCCACCACATTTTCACCGAGGCCACGCGGAGTACGA
t
CGAGTGTACAGTGAACAATGCTAGGGAGAGCTGCCTATATGGAAGAGCCCT

240 2 9 0 11
CTGCTTACGGTTTCGTCCGTGTTGCAGCCGATCATCAGCACATCTAGGTT
tc
GTCCGGGTGTGACCGAAAGGTAAGATGGAGAGCCTTGTCCCTGGTTTCAAC

6510 6 8 0 11 ORF1a-NSL2082I
TGTGAAAACTACCGAAGTTGTAGGAGACATTATACTTAAACCAGCAAATA
ata gtt
TAAAAATTACAGAAGAGGTTGGCCACACAGATCTAATGGCTGCTTATGTAG

26199 3 8 0 11 ORF3a-T271del
TCCGGAGTTGTTAATCCAGTAATGGAACCAATTTATGATGAACCGACGAC
gac
TACTAGCGTGCCTTTGTAAGCACAAGCTGATGAGTACGAACTTATGTACTC

21765 2 7 0 11 S-I68fs
TCAGGACTTGTTCTTACCTTTCTTTTCCAATGTTACTTGGTTCCATGCTA
ta
CATGTCTCTGGGACCAATGGTACTAAGAGGTTTGATAACCCTGTCCTACCA

29762 1 6 0 11
AAAGAGCCACCACATTTTCACCGAGGCCACGCGGAGTACGATCGAGTGTA
c
AGTGAACAATGCTAGGGAGAGCTGCCTATATGGAAGAGCCCTAATGTGTAA

3267 3 6 0 11 ORF1a-T1001del
TCAACAAACTGTTGGTCAACAAGACGGCAGTGAGGACAATCAGACAACTA
cta
TTCAAACAATTGTTGAGGTTCAACCTCAATTAGAGATGGAACTTACACCAG

29757 3 5 0 11
ACTTGAAAGAGCCACCACATTTTCACCGAGGCCACGCGGAGTACGATCGA
gtg
TACAGTGAACAATGCTAGGGAGAGCTGCCTATATGGAAGAGCCCTAATGTG

29739 5 4 0 11
TGTGTAACATTAGGGAGGACTTGAAAGAGCCACCACATTTTCACCGAGGC
cac gc
GGAGTACGATCGAGTGTACAGTGAACAATGCTAGGGAGAGCTGCCTATATG

27614 3 4 0 11 ORF7a-VY74D

TTGCTTTAGCACTCAATTTGCTTTTGCTTGTCCTGACGGCGTAAAACACG
tct
ATCAGTTACGTGCCAGATCAGTTTCACCTAAACTGTTCATCAGACAAGAGG

29722 1 3 0 11
TAGCAATCTTTAATCAGTGTGTAACATTAGGGAGGACTTGAAAGAGCCAC
c
ACATTTTCACCGAGGCCACGCGGAGTACGATCGAGTGTACAGTGAACAATG

3805 3 1 0 11 ORF1a-K1180del
GTTTGTGTAGATACTGTTCGCACAAATGTCTACTTAGCTGTCTTTGATAA
aaa
TCTCTATGACAAACTTGTTTCAAGCTTTTTGGAAATGAAGAGTGAAAAGCA

27758 3 1 0 11 ORF7a-*122fs
GGCAATAGTGTTTATAACACTTTGCTTCACACTCAAAAGAAAGACAGAAT
gat
TGAACTTTCATTAATTGACTTCTATTTGTGCTTTTTAGCCTTTCTGCTATT

27758 3 1 0 11 ORF7b-M1del
GGCAATAGTGTTTATAACACTTTGCTTCACACTCAAAAGAAAGACAGAAT
gat
TGAACTTTCATTAATTGACTTCTATTTGTGCTTTTTAGCCTTTCTGCTATT

25710 6 8 0 10 ORF3a-LY108del
GTTTACTCACACCTTTTGCTCGTTGCTGCTGGCCTTGAAGCCCCTTTTCT
cta tct
TTATGCTTTAGTCTACTTCTTGCAGAGTATAAACTTTGTAAGAATAATAAT

447 1 6 0 10 ORF1a-L61fs
ACATCTTAAAGATGGCACTTGTGGCTTAGTAGAAGTTGAAAAAGGCGTTT
t
GCCTCAACTTGAACAGCCCTATGTGTTCATCAAACGTTCGGATGCTCGAAC

28260 1 5 0 10
AAGACTTTTTAGAGTATCATGACGTTCGTGTTGTTTTAGATTTCATCTAA
a
CGAACAAACTAAAATGTCTGATAATGGACCCCAAAATCAGCGAAATGCACC

29760 3 5 0 10
TGAAAGAGCCACCACATTTTCACCGAGGCCACGCGGAGTACGATCGAGTG
tac
AGTGAACAATGCTAGGGAGAGCTGCCTATATGGAAGAGCCCTAATGTGTAA

1604 3 4 0 10 ORF1a-N447del
ACATAGGTTGTAACCATACAGGTGTTGTTGGAGAAGGTTCCGAAGGTCTT
aat
GACAACCTTCTTGAAATACTCCAAAAAGAGAAAGTCAACATCAATATTGTT

22009 3 4 0 10 S-N149del
TTTCAATTTTGTAATGATCCATTTTTGGGTGTTTATTACCACAAAAACAA
caa
AAGTTGGATGGAAAGTGAGTTCAGAGTTTATTCTAGTGCGAATAATTGCAC

29738 2 4 0 10
GTGTGTAACATTAGGGAGGACTTGAAAGAGCCACCACATTTTCACCGAGG
cc
ACGCGGAGTACGATCGAGTGTACAGTGAACAATGCTAGGGAGAGCTGCCTA

27794 4 4 0 10 ORF7b-FL13fs
AAGAAAGACAGAATGATTGAACTTTCATTAATTGACTTCTATTTGTGCTT
ttt a
GCCTTTCTGCTATTCCTTGTTTTAATTATGCTTATTATCTTTTGGTTCTCA

28153 2 3 0 10 ORF8-T87fs
CATTCAGTACATCGATATCGGTAATTATACAGTTTCCTGTTTACCTTTTA
ca
ATTAATTGCCAGGAACCTAAATTGGGTAGTCTTGTAGTGCGTTGTTCGTTC

29001 1 1 0 10 N-G243fs
ATTGAACCAGCTTGAGAGCAAAATGTCTGGTAAAGGCCAACAACAACAAG
g
CCAAACTGTCACTAAGAAATCTGCTGCTGAGGCTTCTAAGAAGCCTCGGCA

27554 2 1 0 10 ORF7a-F54fs
TGGAACATACGAGGGCAATTCACCATTTCATCCTCTAGCTGATAACAAAT
tt
GCACTGACTTGCTTTAGCACTCAATTTGCTTTTGCTTGTCCTGACGGCGTA

29782 1 9 0 9
CCGAGGCCACGCGGAGTACGATCGAGTGTACAGTGAACAATGCTAGGGAG
a
GCTGCCTATATGGAAGAGCCCTAATGTGTAAAATTAATTTTAGTAGTGCTA

27797 6 8 0 9 ORF7b-LA14del
AAAGACAGAATGATTGAACTTTCATTAATTGACTTCTATTTGTGCTTTTT
agc ctt
TCTGCTATTCCTTGTTTTAATTATGCTTATTATCTTTTGGTTCTCACTTGA

27579 4 8 0 9 ORF7a-QF62fs
TTTCATCCTCTAGCTGATAACAAATTTGCACTGACTTGCTTTAGCACTCA
att t
GCTTTTGCTTGTCCTGACGGCGTAAAACACGTCTATCAGTTACGTGCCAGA

3348 3 8 0 9 ORF1a-FS1028C
ATTAGAGATGGAACTTACACCAGTTGTTCAGACTATTGAAGTGAATAGTT
tta
GTGGTTATTTAAAACTTACTGACAATGTATACATTAAAAATGCAGACATTG

28066 3 7 0 9 ORF8-IE58K
CTATTCTAAATGGTATATTAGAGTAGGAGCTAGAAAATCAGCACCTTTAA
ttg
AATTGTGCGTGGATGAGGCTGGTTCTAAATCACCCATTCAGTACATCGATA

27902 1 6 0 9 ORF8-F3fs
GAACTGCAAGATCATAATGAAACTTGTCACGCCTAAACGAACATGAAATT
t
CTTGTTTTCTTAGGAATCATCACAACTGTAGCTGCATTTCACCAAGAATGT

26486 3 6 0 9
TCTGAATTCTTCTAGAGTTCCTGATCTTCTGGTCTAAACGAACTAAATAT
tat
ATTAGTTTTTCTGTTTGGAACTTTAATTTTAGCCATGGCAGATTCCAACGG

27384 4 6 0 9 ORF6-D*61fs
ACTGAGAATAAATATTCTCAATTAGATGAAGAGCAACCAATGGAGATTGA
tta a
ACGAACATGAAAATTATTCTTTTCTTGGCACTGATAACACTCGCTACTTGT

9857 3 5 0 9 ORF1a-L3199del
GCACCTTTTTGTTAAATAAAGAAATGTATCTAAAGTTGCGTAGTGATGTG
cta
TTACCTCTTACGCAATATAATAGATACTTAGCTCTTTATAATAAGTACAAG

28902 3 5 0 9 N-MA210T
TTCAAGAAATTCAACTCCAGGCAGCAGTAGGGGAACTTCTCCTGCTAGAA
tgg
CTGGCAATGGCGGTGATGCTGCTCTTGCTTTGCTGCTGCTTGACAGATTGA

3835 3 5 0 9 ORF1a-F1190del
TACTTAGCTGTCTTTGATAAAAATCTCTATGACAAACTTGTTTCAAGCTT
ttt
GGAAATGAAGAGTGAAAAGCAAGTTGAACAAAAGATCGCTGAGATTCCTAA

28905 3 4 0 9 N-A211del
AAGAAATTCAACTCCAGGCAGCAGTAGGGGAACTTCTCCTGCTAGAATGG
ctg
GCAATGGCGGTGATGCTGCTCTTGCTTTGCTGCTGCTTGACAGATTGAACC

28254 2 3 0 9 ORF8-I121fs
TCTATGAAGACTTTTTAGAGTATCATGACGTTCGTGTTGTTTTAGATTTC
at
CTAAACGAACAAACTAAAATGTCTGATAATGGACCCCAAAATCAGCGAAAT

27669 1 2 0 9 ORF7a-E92fs
CAGTTACGTGCCAGATCAGTTTCACCTAAACTGTTCATCAGACAAGAGGA
a
GTTCAAGAACTTTACTCTCCAATTTTTCTTATTGTTGCGGCAATAGTGTTT

12600 3 2 0 9 ORF1a-SE4112K
GTGGGAAATCCAACAGGTTGTAGATGCAGATAGTAAAATTGTTCAACTTA
gtg
AAATTAGTATGGACAATTCACCTAATTTAGCATGGCCTCTTATTGTAACAG

27682 2 2 0 9 ORF7a-Y97fs
GATCAGTTTCACCTAAACTGTTCATCAGACAAGAGGAAGTTCAAGAACTT
ta
CTCTCCAATTTTTCTTATTGTTGCGGCAATAGTGTTTATAACACTTTGCTT

28058 1 1 0 9 ORF8-A55fs
ATTCACTTCTATTCTAAATGGTATATTAGAGTAGGAGCTAGAAAATCAGC
a

CCTTTAATTGAATTGTGCGTGGATGAGGCTGGTTCTAAATCACCCATTCAG

28056 1 1 0 9 ORF8-A55fs
CTATTCACTTCTATTCTAAATGGTATATTAGAGTAGGAGCTAGAAAATCA
g
CACCTTTAATTGAATTGTGCGTGGATGAGGCTGGTTCTAAATCACCCATTC

28151 2 1 0 9 ORF8-F86fs
CCCATTCAGTACATCGATATCGGTAATTATACAGTTTCCTGTTTACCTTT
ta
CAATTAATTGCCAGGAACCTAAATTGGGTAGTCTTGTAGTGCGTTGTTCGT

28061 4 1 0 9 ORF8-PL56fs
CACTTCTATTCTAAATGGTATATTAGAGTAGGAGCTAGAAAATCAGCACC
ttt a
ATTGAATTGTGCGTGGATGAGGCTGGTTCTAAATCACCCATTCAGTACATC

28256 1 5 0 8 ORF8-I121fs
TATGAAGACTTTTTAGAGTATCATGACGTTCGTGTTGTTTTAGATTTCAT
c
TAAACGAACAAACTAAAATGTCTGATAATGGACCCCAAAATCAGCGAAATG

27701 2 5 0 8 ORF7a-I103fs
GTTCATCAGACAAGAGGAAGTTCAAGAACTTTACTCTCCAATTTTTCTTA
tt
GTTGCGGCAATAGTGTTTATAACACTTTGCTTCACACTCAAAAGAAAGACA

6524 3 4 0 8 ORF1a-T2087del
AAGTTGTAGGAGACATTATACTTAAACCAGCAAATAATAGTTTAAAAATT
aca
GAAGAGGTTGGCCACACAGATCTAATGGCTGCTTATGTAGACAATTCTAGT

3347 6 4 0 8 ORF1a-FS1028del
AATTAGAGATGGAACTTACACCAGTTGTTCAGACTATTGAAGTGAATAGT
ttt agt
GGTTATTTAAAACTTACTGACAATGTATACATTAAAAATGCAGACATTGTG

28243 6 4 0 8 ORF8-VL117del
GCGTTGTTCGTTCTATGAAGACTTTTTAGAGTATCATGACGTTCGTGTTG
ttt tag
ATTTCATCTAAACGAACAAACTAAAATGTCTGATAATGGACCCCAAAATCA

27577 4 3 0 8 ORF7a-QF62fs
CATTTCATCCTCTAGCTGATAACAAATTTGCACTGACTTGCTTTAGCACT
caa t
TTGCTTTTGCTTGTCCTGACGGCGTAAAACACGTCTATCAGTTACGTGCCA

29742 4 3 0 8
GTAACATTAGGGAGGACTTGAAAGAGCCACCACATTTTCACCGAGGCCAC
gcg g
AGTACGATCGAGTGTACAGTGAACAATGCTAGGGAGAGCTGCCTATATGGA

28245 3 3 0 8 ORF8-L118del
GTTGTTCGTTCTATGAAGACTTTTTAGAGTATCATGACGTTCGTGTTGTT

tta
GATTTCATCTAAACGAACAAACTAAAATGTCTGATAATGGACCCCAAAATC

28247 6 2 0 8 ORF8-LD118del
TGTTCGTTCTATGAAGACTTTTTAGAGTATCATGACGTTCGTGTTGTTTT
aga ttt
CATCTAAACGAACAAACTAAAATGTCTGATAATGGACCCCAAAATCAGCGA

29819 3 2 0 8
CAATGCTAGGGAGAGCTGCCTATATGGAAGAGCCCTAATGTGTAAAATTA
att
TTAGTAGTGCTATCCCCATGTGATTTTAATAGCTTCTTAGGAGAATGACAA

28250 3 2 0 8 ORF8-F120del
TCGTTCTATGAAGACTTTTTAGAGTATCATGACGTTCGTGTTGTTTTAGA
ttt
CATCTAAACGAACAAACTAAAATGTCTGATAATGGACCCCAAAATCAGCGA

12783 3 1 0 8 ORF1a-N4173del
CGGTACTACACAAACTGCTTGCACTGATGACAATGCGTTAGCTTACTACA
aca
CAACAAAGGGAGGTAGGTTTGTACTTGCACTGTTATCCGATTTACAGGATT

27735 1 1 0 8 ORF7a-F114fs
TCTCCAATTTTTCTTATTGTTGCGGCAATAGTGTTTATAACACTTTGCTT
c
ACACTCAAAAGAAAGACAGAATGATTGAACTTTCATTAATTGACTTCTATT

28350 3 1 0 8 N-S26del
GCGAAATGCACCCCGCATTACGTTTGGTGGACCCTCAGATTCAACTGGCA
gta
ACCAGAATGGAGAACGCAGTGGGGCGCGATCAAAACAACGTCGGCCCCAAG

16853 3 1 0 8 ORF1b-G1129del
AAACAGTAAAGTACAAATAGGAGAGTACACCTTTGAAAAAGGTGACTATG
gtg
ATGCTGTTGTTTACCGAGGTACAACAACTTACAAATTAAATGTTGGTGATT

29867 1 7 0 7
TAATTTTAGTAGTGCTATCCCCATGTGATTTTAATAGCTTCTTAGGAGAA
t
GACAAAAAAAAAAAAAAAAAAAAAAAAAAAAAAAAAA

523 1 7 0 7 ORF1a-V86fs
TTCATCAAACGTTCGGATGCTCGAACTGCACCTCATGGTCATGTTATGGT
t
GAGCTGGTAGCAGAACTCGAAGGCATTCAGTACGGTCGTAGTGGTGAGACA

25535 3 7 0 7 ORF3a-V48del
TACTGCAACGATACCGATACAAGCCTCACTCCCTTTCGGATGGCTTATTG
ttg
GCGTTGCACTTCTTGCTGTTTTTCAGAGCGCTTCCAAAATCATAACCCTCA

28253 5 6 0 7 ORF8-FI120fs

TTCTATGAAGACTTTTTAGAGTATCATGACGTTCGTGTTGTTTTAGATTT
cat ct
AAACGAACAAACTAAAATGTCTGATAATGGACCCCAAAATCAGCGAAATGC

29779 1 6 0 7
TCACCGAGGCCACGCGGAGTACGATCGAGTGTACAGTGAACAATGCTAGG
g
AGAGCTGCCTATATGGAAGAGCCCTAATGTGTAAAATTAATTTTAGTAGTG

3345 3 6 0 7 ORF1a-SF1027I
TCAATTAGAGATGGAACTTACACCAGTTGTTCAGACTATTGAAGTGAATA
gtt
TTAGTGGTTATTTAAAACTTACTGACAATGTATACATTAAAAATGCAGACA

20339 3 6 0 7 ORF1b-FS2291C
ACGGTATAAATTAGAAGGCTATGCCTTCGAACATATCGTTTATGGAGATT
tta
GTCATAGTCAGTTAGGTGGTTTACATCTACTGATTGGACTAGCTAAACGTT

28103 2 5 0 7 ORF8-P70fs
TCAGCACCTTTAATTGAATTGTGCGTGGATGAGGCTGGTTCTAAATCACC
ca
TTCAGTACATCGATATCGGTAATTATACAGTTTCCTGTTTACCTTTTACAA

28072 4 5 0 7 ORF8-LC60fs
TAAATGGTATATTAGAGTAGGAGCTAGAAAATCAGCACCTTTAATTGAAT
tgt g
CGTGGATGAGGCTGGTTCTAAATCACCCATTCAGTACATCGATATCGGTAA

29585 6 4 0 7 ORF10-PF10del
CTCATGCAGACCACACAAGGCAGATGGGCTATATAAACGTTTTCGCTTTT
ccg ttt
ACGATATATAGTCTACTCTTGTGCAGAATGAATTCTCGTAACTACATAGCA

29404 3 4 0 7 N-D377del
AAAACATTCCCACCAACAGAGCCTAAAAAGGACAAAAAGAAGAAGGCTGA
tga
AACTCAAGCCTTACCGCAGAGACAGAAGAAACAGCAAACTGTGACTCTTCT

21762 6 3 0 7 S-AIH67D
AACTCAGGACTTGTTCTTACCTTTCTTTTCCAATGTTACTTGGTTCCATG
cta tac
ATGTCTCTGGGACCAATGGTACTAAGAGGTTTGATAACCCTGTCCTACCAT

27584 4 3 0 7 ORF7a-AF64fs
TCCTCTAGCTGATAACAAATTTGCACTGACTTGCTTTAGCACTCAATTTG
ctt t
TGCTTGTCCTGACGGCGTAAAACACGTCTATCAGTTACGTGCCAGATCAGT

28248 5 3 0 7 ORF8-DF119fs
GTTCGTTCTATGAAGACTTTTTAGAGTATCATGACGTTCGTGTTGTTTTA
gat tt
CATCTAAACGAACAAACTAAAATGTCTGATAATGGACCCCAAAATCAGCGA

29682 2 3 0 7
CTACATAGCACAAGTAGATGTAGTTAACTTTAATCTCACATAGCAATCTT
ta
ATCAGTGTGTAACATTAGGGAGGACTTGAAAGAGCCACCACATTTTCACCG

27741 1 2 0 7 ORF7a-L116fs
ATTTTTCTTATTGTTGCGGCAATAGTGTTTATAACACTTTGCTTCACACT
c
AAAAGAAAGACAGAATGATTGAACTTTCATTAATTGACTTCTATTTGTGCT

27583 5 2 0 7 ORF7a-AF64fs
ATCCTCTAGCTGATAACAAATTTGCACTGACTTGCTTTAGCACTCAATTT
gct tt
TGCTTGTCCTGACGGCGTAAAACACGTCTATCAGTTACGTGCCAGATCAGT

29778 4 2 0 7
TTCACCGAGGCCACGCGGAGTACGATCGAGTGTACAGTGAACAATGCTAG
gga g
AGCTGCCTATATGGAAGAGCCCTAATGTGTAAAATTAATTTTAGTAGTGCT

29823 5 2 0 7
GCTAGGGAGAGCTGCCTATATGGAAGAGCCCTAATGTGTAAAATTAATTT
tag ta
GTGCTATCCCCATGTGATTTTAATAGCTTCTTAGGAGAATGACAAAAAAAA

27266 2 2 0 7 ORF6-F22fs
TGACTTTCAGGTTACTATAGCAGAGATATTACTAATTATTATGAGGACTT
tt
AAAGTTTCCATTTGGAATCTTGATTACATCATAAACCTCATAATTAAAAAT

27392 1 1 0 7
TAAATATTCTCAATTAGATGAAGAGCAACCAATGGAGATTGATTAAACGA
a
CATGAAAATTATTCTTTTCTTGGCACTGATAACACTCGCTACTTGTGAGCT

1948 3 1 0 7 ORF1a-L562del
CGATCAATTTTCTCCCGCACTCTTGAAACTGCTCAAAATTCTGTGCGTGT
ttt
ACAGAAGGCCGCTATAACAATACTAGATGGAATTTCACAGTATTCACTGAG

518 2 6 0 6 ORF1a-M85fs
ATGTGTTCATCAAACGTTCGGATGCTCGAACTGCACCTCATGGTCATGTT
at
GGTTGAGCTGGTAGCAGAACTCGAAGGCATTCAGTACGGTCGTAGTGGTGA

25394 2 6 0 6 ORF3a-M1fs
CTGAGCCAGTGCTCAAAGGAGTCAAATTACATTACACATAAACGAACTTA
tg
GATTTGTTTATGAGAATCTTCACAATTGGAACTGTAACTTTGAAGCAAGGT

29772 2 6 0 6
CACATTTTCACCGAGGCCACGCGGAGTACGATCGAGTGTACAGTGAACAA
tg
CTAGGGAGAGCTGCCTATATGGAAGAGCCCTAATGTGTAAAATTAATTTTA

27291 3 6 0 6 ORF6-Y31del
ATATTACTAATTATTATGAGGACTTTTAAAGTTTCCATTTGGAATCTTGA
tta
CATCATAAACCTCATAATTAAAAATTTATCTAAGTCACTAACTGAGAATAA

25432 4 5 0 6 ORF3a-TL14fs
TAAACGAACTTATGGATTTGTTTATGAGAATCTTCACAATTGGAACTGTA
act t
TGAAGCAAGGTGAAATCAAGGATGCTACTCCTTCAGATTTTGTTCGCGCTA

25552 6 5 0 6 ORF3a-AV54del
TACAAGCCTCACTCCCTTTCGGATGGCTTATTGTTGGCGTTGCACTTCTT
gct gtt
TTTCAGAGCGCTTCCAAAATCATAACCCTCAAAAAGAGATGGCAACTAGCA

29764 3 5 0 6
AGAGCCACCACATTTTCACCGAGGCCACGCGGAGTACGATCGAGTGTACA
gtg
AACAATGCTAGGGAGAGCTGCCTATATGGAAGAGCCCTAATGTGTAAAATT

7116 6 5 0 6 ORF1a-GSI2284V
AGAAGGCTATTTGAACTCTACTAATGTCACTATTGCAACCTACTGTACTG
gtt cta
TACCTTGTAGTGTTTGTCTTAGTGGTTTAGATTCTTTAGACACCTATCCTT

29793 1 5 0 6
CGGAGTACGATCGAGTGTACAGTGAACAATGCTAGGGAGAGCTGCCTATA
t
GGAAGAGCCCTAATGTGTAAAATTAATTTTAGTAGTGCTATCCCCATGTGA

29785 2 5 0 6
AGGCCACGCGGAGTACGATCGAGTGTACAGTGAACAATGCTAGGGAGAGC
tg
CCTATATGGAAGAGCCCTAATGTGTAAAATTAATTTTAGTAGTGCTATCCC

77 2 5 0 6
ACAAACCAACCAACTTTCGATCTCTTGTAGATCTGTTCTCTAAACGAACT
tt
AAAATCTGTGTGGCTGTCACTCGGCTGCATGCTTAGTGCACTCACGCAGTA

28244 3 5 0 6 ORF8-L118del
CGTTGTTCGTTCTATGAAGACTTTTTAGAGTATCATGACGTTCGTGTTGT
ttt
AGATTTCATCTAAACGAACAAACTAAAATGTCTGATAATGGACCCCAAAAT

29749 4 4 0 6
TAGGGAGGACTTGAAAGAGCCACCACATTTTCACCGAGGCCACGCGGAGT
acg a
TCGAGTGTACAGTGAACAATGCTAGGGAGAGCTGCCTATATGGAAGAGCCC

27590 4 4 0 6 ORF7a-AC66fs
AGCTGATAACAAATTTGCACTGACTTGCTTTAGCACTCAATTTGCTTTTG
ctt g

TCCTGACGGCGTAAAACACGTCTATCAGTTACGTGCCAGATCAGTTTCACC

28068 1 4 0 6 ORF8-E59fs
ATTCTAAATGGTATATTAGAGTAGGAGCTAGAAAATCAGCACCTTTAATT
g
AATTGTGCGTGGATGAGGCTGGTTCTAAATCACCCATTCAGTACATCGATA

27217 6 4 0 6 ORF6-DF6del
CAATATTGCTTTGCTTGTACAGTAAGTGACAACAGATGTTTCATCTCGTT
gac ttt
CAGGTTACTATAGCAGAGATATTACTAATTATTATGAGGACTTTTAAAGTT

21763 1 4 0 6 S-A67fs
ACTCAGGACTTGTTCTTACCTTTCTTTTCCAATGTTACTTGGTTCCATGC
t
ATACATGTCTCTGGGACCAATGGTACTAAGAGGTTTGATAACCCTGTCCTA

27682 1 4 0 6 ORF7a-Y97fs
GATCAGTTTCACCTAAACTGTTCATCAGACAAGAGGAAGTTCAAGAACTT
t
ACTCTCCAATTTTTCTTATTGTTGCGGCAATAGTGTTTATAACACTTTGCT

27796 2 4 0 6 ORF7b-L14fs
GAAAGACAGAATGATTGAACTTTCATTAATTGACTTCTATTTGTGCTTTT
ta
GCCTTTCTGCTATTCCTTGTTTTAATTATGCTTATTATCTTTTGGTTCTCA

27207 2 4 0 6 ORF6-F2fs
GTAGCAGTGACAATATTGCTTTGCTTGTACAGTAAGTGACAACAGATGTT
tc
ATCTCGTTGACTTTCAGGTTACTATAGCAGAGATATTACTAATTATTATGA

27695 5 4 0 6 ORF7a-FL101fs
TAAACTGTTCATCAGACAAGAGGAAGTTCAAGAACTTTACTCTCCAATTT
ttc tt
ATTGTTGCGGCAATAGTGTTTATAACACTTTGCTTCACACTCAAAAGAAAG

27697 1 3 0 6 ORF7a-L102fs
AACTGTTCATCAGACAAGAGGAAGTTCAAGAACTTTACTCTCCAATTTTT
c
TTATTGTTGCGGCAATAGTGTTTATAACACTTTGCTTCACACTCAAAAGAA

3236 3 3 0 6 ORF1a-Q991del
AGCAAGAAGAAGATTGGTTAGATGATGATAGTCAACAAACTGTTGGTCAA
caa
GACGGCAGTGAGGACAATCAGACAACTACTATTCAAACAATTGTTGAGGTT

6573 3 3 0 6 ORF1a-SS2103C
TACAGAAGAGGTTGGCCACACAGATCTAATGGCTGCTTATGTAGACAATT
cta
GTCTTACTATTAAGAAACCTAATGAATTATCTAGAGTATTAGGTTTGAAAA

27602 3 3 0 6 ORF7a-G70del
ATTTGCACTGACTTGCTTTAGCACTCAATTTGCTTTTGCTTGTCCTGACG

gcg
TAAAACACGTCTATCAGTTACGTGCCAGATCAGTTTCACCTAAACTGTTCA

11292 5 3 0 6 ORF1a-GF3676fs
GGTGATGCGTATTATGACATGGTTGGATATGGTTGATACTAGTTTGTCTG
gtt tt
AAGCTAAAAGACTGTGTTATGTATGCATCAGCTGTAGTGTTACTAATCCTT

27581 2 3 0 6 ORF7a-F63fs
TCATCCTCTAGCTGATAACAAATTTGCACTGACTTGCTTTAGCACTCAAT
tt
GCTTTTGCTTGTCCTGACGGCGTAAAACACGTCTATCAGTTACGTGCCAGA

11543 3 3 0 6 ORF1a-F3760del
ACTACTCAGGTGTAGTTACAACTGTCATGTTTTTGGCCAGAGGTATTGTT
ttt
ATGTGTGTTGAGTATTGCCCTATTTTCTTCATAACTGGTAATACACTTCAG

29866 1 2 0 6
TTAATTTTAGTAGTGCTATCCCCATGTGATTTTAATAGCTTCTTAGGAGA
a
TGACAAAAAAAAAAAAAAAAAAAAAAAAAAAAAAAAA

27985 2 2 0 6 ORF8-Y31fs
TGCATTTCACCAAGAATGTAGTTTACAGTCATGTACTCAACATCAACCAT
at
GTAGTTGATGACCCGTGTCCTATTCACTTCTATTCTAAATGGTATATTAGA

29719 1 2 0 6
ACATAGCAATCTTTAATCAGTGTGTAACATTAGGGAGGACTTGAAAGAGC
c
ACCACATTTTCACCGAGGCCACGCGGAGTACGATCGAGTGTACAGTGAACA

1457 3 2 0 6 ORF1a-R398del
AGCATAGTCTTGCCGAATACCATAATGAATCTGGCTTGAAAACCATTCTT
cgt
AAGGGTGGTCGCACTATTGCCTTTGGAGGCTGTGTGTTCTCTTATGTTGGT

29764 1 2 0 6
AGAGCCACCACATTTTCACCGAGGCCACGCGGAGTACGATCGAGTGTACA
g
TGAACAATGCTAGGGAGAGCTGCCTATATGGAAGAGCCCTAATGTGTAAAA

27676 3 2 0 6 ORF7a-E95del
GTGCCAGATCAGTTTCACCTAAACTGTTCATCAGACAAGAGGAAGTTCAA
gaa
CTTTACTCTCCAATTTTTCTTATTGTTGCGGCAATAGTGTTTATAACACTT

29766 4 2 0 6
AGCCACCACATTTTCACCGAGGCCACGCGGAGTACGATCGAGTGTACAGT
gaa c
AATGCTAGGGAGAGCTGCCTATATGGAAGAGCCCTAATGTGTAAAATTAAT

25437 6 2 0 6 ORF3a-KQ16del

GAACTTATGGATTTGTTTATGAGAATCTTCACAATTGGAACTGTAACTTT
gaa gca
AGGTGAAATCAAGGATGCTACTCCTTCAGATTTTGTTCGCGCTACTGCAAC

923 6 2 0 6 ORF1a-DF220del
ACCTTCTAGCACGTGCTGGTAAAGCTTCATGCACTTTGTCCGAACAACTG
gac ttt
ATTGACACTAAGAGGGGTGTATACTGCTGCCGTGAACATGAGCATGAAATT

29751 5 2 0 6
GGGAGGACTTGAAAGAGCCACCACATTTTCACCGAGGCCACGCGGAGTAC
gat cg
AGTGTACAGTGAACAATGCTAGGGAGAGCTGCCTATATGGAAGAGCCCTAA

27382 4 2 0 6 ORF6-D*61fs
TAACTGAGAATAAATATTCTCAATTAGATGAAGAGCAACCAATGGAGATT
gat t
AAACGAACATGAAAATTATTCTTTTCTTGGCACTGATAACACTCGCTACTT

28028 5 2 0 6 ORF8-WY45fs
CAACCATATGTAGTTGATGACCCGTGTCCTATTCACTTCTATTCTAAATG
gta ta
TTAGAGTAGGAGCTAGAAAATCAGCACCTTTAATTGAATTGTGCGTGGATG

27614 1 2 0 6 ORF7a-V74fs
TTGCTTTAGCACTCAATTTGCTTTTGCTTGTCCTGACGGCGTAAAACACG
t
CTATCAGTTACGTGCCAGATCAGTTTCACCTAAACTGTTCATCAGACAAGA

27910 1 2 0 6 ORF8-F6fs
AGATCATAATGAAACTTGTCACGCCTAAACGAACATGAAATTTCTTGTTT
t
CTTAGGAATCATCACAACTGTAGCTGCATTTCACCAAGAATGTAGTTTACA

27616 3 2 0 6 ORF7a-Y75del
GCTTTAGCACTCAATTTGCTTTTGCTTGTCCTGACGGCGTAAAACACGTC
tat
CAGTTACGTGCCAGATCAGTTTCACCTAAACTGTTCATCAGACAAGAGGAA

26514 1 1 0 6
CTGGTCTAAACGAACTAAATATTATATTAGTTTTTCTGTTTGGAACTTTA
a
TTTTAGCCATGGCAGATTCCAACGGTACTATTACCGTTGAAGAGCTTAAAA

28103 1 1 0 6 ORF8-P70fs
TCAGCACCTTTAATTGAATTGTGCGTGGATGAGGCTGGTTCTAAATCACC
c
ATTCAGTACATCGATATCGGTAATTATACAGTTTCCTGTTTACCTTTTACA

27762 6 1 0 6 ORF7b-EL3del
ATAGTGTTTATAACACTTTGCTTCACACTCAAAAGAAAGACAGAATGATT
gaa ctt
TCATTAATTGACTTCTATTTGTGCTTTTTAGCCTTTCTGCTATTCCTTGTT

28975 3 1 0 6 N-MS234I
GCTCTTGCTTTGCTGCTGCTTGACAGATTGAACCAGCTTGAGAGCAAAAT
gtc
TGGTAAAGGCCAACAACAACAAGGCCAAACTGTCACTAAGAAATCTGCTGC

28842 6 1 0 6 N-SR190del
AGAAGGGAGCAGAGGCGGCAGTCAAGCCTCTTCTCGTTCCTCATCACGTA
gtc gca
ACAGTTCAAGAAATTCAACTCCAGGCAGCAGTAGGGGAACTTCTCCTGCTA

25520 1 1 0 6 ORF3a-F43fs
AGATTTTGTTCGCGCTACTGCAACGATACCGATACAAGCCTCACTCCCTT
t
CGGATGGCTTATTGTTGGCGTTGCACTTCTTGCTGTTTTTCAGAGCGCTTC

21993 1 5 0 5 S-Y144fs
TATTAAAGTCTGTGAATTTCAATTTTGTAATGATCCATTTTTGGGTGTTT
a
TTACCACAAAAACAACAAAAGTTGGATGGAAAGTGAGTTCAGAGTTTATTC

28030 1 5 0 5 ORF8-Y46fs
ACCATATGTAGTTGATGACCCGTGTCCTATTCACTTCTATTCTAAATGGT
a
TATTAGAGTAGGAGCTAGAAAATCAGCACCTTTAATTGAATTGTGCGTGGA

28032 1 5 0 5 ORF8-I47fs
CATATGTAGTTGATGACCCGTGTCCTATTCACTTCTATTCTAAATGGTAT
a
TTAGAGTAGGAGCTAGAAAATCAGCACCTTTAATTGAATTGTGCGTGGATG

24070 3 5 0 5 S-QY836H
GATCTACTTTTCAACAAAGTGACACTTGCAGATGCTGGCTTCATCAAACA
ata
TGGTGATTGCCTTGGTGATATTGCTGCTAGAGACCTCATTTGTGCACAAAA

23484 3 5 0 5 S-NV641I
TCATGCAGATCAACTTACTCCTACTTGGCGTGTTTATTCTACAGGTTCTA
atg
TTTTTCAAACACGTGCAGGCTGTTTAATAGGGGCTGAACATGTCAACAACT

21989 3 5 0 5 S-V143del
TTGTTATTAAAGTCTGTGAATTTCAATTTTGTAATGATCCATTTTTGGGT
gtt
TATTACCACAAAAACAACAAAAGTTGGATGGAAAGTGAGTTCAGAGTTTAT

21715 1 5 0 5 S-T51fs
GGTGTTTATTACCCTGACAAAGTTTTCAGATCCTCAGTTTTACATTCAAC
t
CAGGACTTGTTCTTACCTTTCTTTTCCAATGTTACTTGGTTCCATGCTATA

29741 3 4 0 5
TGTAACATTAGGGAGGACTTGAAAGAGCCACCACATTTTCACCGAGGCCA
cgc
GGAGTACGATCGAGTGTACAGTGAACAATGCTAGGGAGAGCTGCCTATATG

11289 2 4 0 5 ORF1a-S3675fs
TTGGGTGATGCGTATTATGACATGGTTGGATATGGTTGATACTAGTTTGT
ct
GGTTTTAAGCTAAAAGACTGTGTTATGTATGCATCAGCTGTAGTGTTACTA

242 3 4 0 5
GCTTACGGTTTCGTCCGTGTTGCAGCCGATCATCAGCACATCTAGGTTTC
gtc
CGGGTGTGACCGAAAGGTAAGATGGAGAGCCTTGTCCCTGGTTTCAACGAG

3711 3 4 0 5 ORF1a-L1149del
GAGTGCTTATGAAAATTTTAATCAGCACGAAGTTCTACTTGCACCATTAT
tat
CAGCTGGTATTTTTGGTGCTGACCCTATACATTCTTTAAGAGTTTGTGTAG

21994 2 4 0 5 S-Y144fs
ATTAAAGTCTGTGAATTTCAATTTTGTAATGATCCATTTTTGGGTGTTTA
tt
ACCACAAAAACAACAAAAGTTGGATGGAAAGTGAGTTCAGAGTTTATTCTA

29745 3 3 0 5
ACATTAGGGAGGACTTGAAAGAGCCACCACATTTTCACCGAGGCCACGCG
gag
TACGATCGAGTGTACAGTGAACAATGCTAGGGAGAGCTGCCTATATGGAAG

25477 3 3 0 5 ORF3a-V29del
CTGTAACTTTGAAGCAAGGTGAAATCAAGGATGCTACTCCTTCAGATTTT
gtt
CGCGCTACTGCAACGATACCGATACAAGCCTCACTCCCTTTCGGATGGCTT

28151 1 3 0 5 ORF8-F86fs
CCCATTCAGTACATCGATATCGGTAATTATACAGTTTCCTGTTTACCTTT
t
ACAATTAATTGCCAGGAACCTAAATTGGGTAGTCTTGTAGTGCGTTGTTCG

27720 4 3 0 5 ORF7a-FI109fs
GTTCAAGAACTTTACTCTCCAATTTTTCTTATTGTTGCGGCAATAGTGTT
tat a
ACACTTTGCTTCACACTCAAAAGAAAGACAGAATGATTGAACTTTCATTAA

27425 1 2 0 5 ORF7a-T11fs
GGAGATTGATTAAACGAACATGAAAATTATTCTTTTCTTGGCACTGATAA
c
ACTCGCTACTTGTGAGCTTTATCACTACCAAGAGTGTGTTAGAGGTACAAC

28253 1 2 0 5 ORF8-F120fs
TTCTATGAAGACTTTTTAGAGTATCATGACGTTCGTGTTGTTTTAGATTT
c
ATCTAAACGAACAAACTAAAATGTCTGATAATGGACCCCAAAATCAGCGAA

26158 1 2 0 5 ORF3a-V256fs
AGCCTGAAGAACATGTCCAAATTCACACAATCGACGGTTCATCCGGAGTT
g

TTAATCCAGTAATGGAACCAATTTATGATGAACCGACGACGACTACTAGCG

28198 1 2 0 5 ORF8-C102fs
TTTTACAATTAATTGCCAGGAACCTAAATTGGGTAGTCTTGTAGTGCGTT
g
TTCGTTCTATGAAGACTTTTTAGAGTATCATGACGTTCGTGTTGTTTTAGA

29841 1 2 0 5
TATGGAAGAGCCCTAATGTGTAAAATTAATTTTAGTAGTGCTATCCCCAT
g
TGATTTTAATAGCTTCTTAGGAGAATGACAAAAAAAAAAAAAAAAAAAAAA

27422 1 2 0 5 ORF7a-I10fs
AATGGAGATTGATTAAACGAACATGAAAATTATTCTTTTCTTGGCACTGA
t
AACACTCGCTACTTGTGAGCTTTATCACTACCAAGAGTGTGTTAGAGGTAC

27511 2 2 0 5 ORF7a-Y40fs
GTGTTAGAGGTACAACAGTACTTTTAAAAGAACCTTGCTCTTCTGGAACA
ta
CGAGGGCAATTCACCATTTCATCCTCTAGCTGATAACAAATTTGCACTGAC

221 2 2 0 5
CTCGTCTATCTTCTGCAGGCTGCTTACGGTTTCGTCCGTGTTGCAGCCGA
tc
ATCAGCACATCTAGGTTTCGTCCGGGTGTGACCGAAAGGTAAGATGGAGAG

1602 3 2 0 5 ORF1a-LN446H
TAACATAGGTTGTAACCATACAGGTGTTGTTGGAGAAGGTTCCGAAGGTC
tta
ATGACAACCTTCTTGAAATACTCCAAAAAGAGAAAGTCAACATCAATATTG

21632 3 2 0 5 S-L24del
TATTGCCACTAGTCTCTAGTCAGTGTGTTAATCTTACAACCAGAACTCAA
tta
CCCCCTGCATACACTAATTCTTTCACACGTGGTGTTTATTACCCTGACAAA

27515 4 1 0 5 ORF7a-EG41fs
TAGAGGTACAACAGTACTTTTAAAAGAACCTTGCTCTTCTGGAACATACG
agg g
CAATTCACCATTTCATCCTCTAGCTGATAACAAATTTGCACTGACTTGCTT

25718 3 1 0 5 ORF3a-YA109S
ACACCTTTTGCTCGTTGCTGCTGGCCTTGAAGCCCCTTTTCTCTATCTTT
atg
CTTTAGTCTACTTCTTGCAGAGTATAAACTTTGTAAGAATAATAATGAGGC

22295 6 1 0 5 S-HR245del
ATTTGCCAATAGGTATTAACATCACTAGGTTTCAAACTTTACTTGCTTTA
cat aga
AGTTATTTGACTCCTGGTGATTCTTCTTCAGGTTGGACAGCTGGTGCTGCA

27595 3 1 0 5 ORF7a-P68del
ATAACAAATTTGCACTGACTTGCTTTAGCACTCAATTTGCTTTTGCTTGT

cct
GACGGCGTAAAACACGTCTATCAGTTACGTGCCAGATCAGTTTCACCTAAA

25714 3 1 0 5 ORF3a-L108del
ACTCACACCTTTTGCTCGTTGCTGCTGGCCTTGAAGCCCCTTTTCTCTAT
ctt
TATGCTTTAGTCTACTTCTTGCAGAGTATAAACTTTGTAAGAATAATAATG

2933 3 1 0 5 ORF1a-L890del
GTGTTGTGGCAGATGCTGTCATAAAAACTTTGCAACCAGTATCTGAATTA
ctt
ACACCACTGGGCATTGATTTAGATGAGTGGAGTATGGCTACATACTACTTA

27382 1 1 0 5 ORF6-D61fs
TAACTGAGAATAAATATTCTCAATTAGATGAAGAGCAACCAATGGAGATT
g
ATTAAACGAACATGAAAATTATTCTTTTCTTGGCACTGATAACACTCGCTA

29543 5 1 0 5
TGCAACAATCCATGAGCAGTGCTGACTCAACTCAGGCCTAAACTCATGCA
gac ca
CACAAGGCAGATGGGCTATATAAACGTTTTCGCTTTTCCGTTTACGATATA

8963 3 1 0 5 ORF1a-S2900del
ATTTCTTACCTAGAGTTTTTAGTGCAGTTGGTAACATCTGTTACACACCA
tca
AAACTTATAGAGTACACTGACTTTGCAACATCAGCTTGTGTTTTGGCTGCT

3905 3 1 0 5 ORF1a-F1214del
AGCAAGTTGAACAAAAGATCGCTGAGATTCCTAAAGAGGAAGTTAAGCCA
ttt
ATAACTGAAAGTAAACCTTCAGTTGAACAGAGAAAACAAGATGATAAGAAA

26487 1 4 0 4
CTGAATTCTTCTAGAGTTCCTGATCTTCTGGTCTAAACGAACTAAATATT
a
TATTAGTTTTTCTGTTTGGAACTTTAATTTTAGCCATGGCAGATTCCAACG

28416 3 4 0 4 N-N48del
ACGCAGTGGGGCGCGATCAAAACAACGTCGGCCCCAAGGTTTACCCAATA
ata
CTGCGTCTTGGTTCACCGCTCTCACTCAACATGGCAAGGAAGACCTTAAAT

21612 6 4 0 4 S-NL17del
AATGTTTGTTTTTCTTGTTTTATTGCCACTAGTCTCTAGTCAGTGTGTTA
atc tta
CAACCAGAACTCAATTACCCCCTGCATACACTAATTCTTTCACACGTGGTG

513 6 4 0 4 ORF1a-HVM83L
GCCCTATGTGTTCATCAAACGTTCGGATGCTCGAACTGCACCTCATGGTC
atg tta
TGGTTGAGCTGGTAGCAGAACTCGAAGGCATTCAGTACGGTCGTAGTGGTG

21575 1 4 0 4 S-L5fs

TATTTCTAGTGATGTTCTTGTTAACAACTAAACGAACAATGTTTGTTTTT
c
TTGTTTTATTGCCACTAGTCTCTAGTCAGTGTGTTAATCTTACAACCAGAA

25433 6 4 0 4 ORF3a-TL14del
AAACGAACTTATGGATTTGTTTATGAGAATCTTCACAATTGGAACTGTAA
ctt tga
AGCAAGGTGAAATCAAGGATGCTACTCCTTCAGATTTTGTTCGCGCTACTG

28242 1 4 0 4 ORF8-V117fs
TGCGTTGTTCGTTCTATGAAGACTTTTTAGAGTATCATGACGTTCGTGTT
g
TTTTAGATTTCATCTAAACGAACAAACTAAAATGTCTGATAATGGACCCCA

29783 1 4 0 4
CGAGGCCACGCGGAGTACGATCGAGTGTACAGTGAACAATGCTAGGGAGA
g
CTGCCTATATGGAAGAGCCCTAATGTGTAAAATTAATTTTAGTAGTGCTAT

521 1 4 0 4 ORF1a-V86fs
TGTTCATCAAACGTTCGGATGCTCGAACTGCACCTCATGGTCATGTTATG
g
TTGAGCTGGTAGCAGAACTCGAAGGCATTCAGTACGGTCGTAGTGGTGAGA

29757 5 4 0 4
ACTTGAAAGAGCCACCACATTTTCACCGAGGCCACGCGGAGTACGATCGA
gtg ta
CAGTGAACAATGCTAGGGAGAGCTGCCTATATGGAAGAGCCCTAATGTGTA

26284 3 4 0 4 E-V14del
AGTACGAACTTATGTACTCATTCGTTTCGGAAGAGACAGGTACGTTAATA
gtt
AATAGCGTACTTCTTTTTCTTGCTTTCGTGGTATTCTTGCTAGTTACACTA

28252 1 4 0 4 ORF8-F120fs
GTTCTATGAAGACTTTTTAGAGTATCATGACGTTCGTGTTGTTTTAGATT
t
CATCTAAACGAACAAACTAAAATGTCTGATAATGGACCCCAAAATCAGCGA

28094 2 4 0 4 ORF8-S67fs
GCTAGAAAATCAGCACCTTTAATTGAATTGTGCGTGGATGAGGCTGGTTC
ta
AATCACCCATTCAGTACATCGATATCGGTAATTATACAGTTTCCTGTTTAC

29568 2 4 0 4 ORF10-I4fs
CTCAACTCAGGCCTAAACTCATGCAGACCACACAAGGCAGATGGGCTATA
ta
AACGTTTTCGCTTTTCCGTTTACGATATATAGTCTACTCTTGTGCAGAATG

6654 3 4 0 4 ORF1a-VN2130D
ATCTAGAGTATTAGGTTTGAAAACCCTTGCTACTCATGGTTTAGCTGCTG
tta
ATAGTGTCCCTTGGGATACTATAGCTAATTATGCTAAGCCTTTTCTTAACA

27578 2 3 0 4 ORF7a-Q62fs
ATTTCATCCTCTAGCTGATAACAAATTTGCACTGACTTGCTTTAGCACTC
aa
TTTGCTTTTGCTTGTCCTGACGGCGTAAAACACGTCTATCAGTTACGTGCC

686 2 3 0 4 ORF1a-K141fs
GTAAGAACGGTAATAAAGGAGCTGGTGGCCATAGTTACGGCGCCGATCTA
aa
GTCATTTGACTTAGGCGACGAGCTTGGCACTGATCCTTATGAAGATTTTCA

20065 3 3 0 4 ORF1b-K2200del
ACTTATTTAGAAATGCCCGTAATGGTGTTCTTATTACAGAAGGTAGTGTT
aaa
GGTTTACAACCATCTGTAGGTCCCAAACAAGCTAGTCTTAATGGAGTCACA

28069 4 3 0 4 ORF8-EL59fs
TTCTAAATGGTATATTAGAGTAGGAGCTAGAAAATCAGCACCTTTAATTG
aat t
GTGCGTGGATGAGGCTGGTTCTAAATCACCCATTCAGTACATCGATATCGG

25393 3 3 0 4 ORF3a-M1del
TCTGAGCCAGTGCTCAAAGGAGTCAAATTACATTACACATAAACGAACTT
atg
GATTTGTTTATGAGAATCTTCACAATTGGAACTGTAACTTTGAAGCAAGGT

28253 2 3 0 4 ORF8-F120fs
TTCTATGAAGACTTTTTAGAGTATCATGACGTTCGTGTTGTTTTAGATTT
ca
TCTAAACGAACAAACTAAAATGTCTGATAATGGACCCCAAAATCAGCGAAA

7858 6 3 0 4 ORF1a-NT2531del
AGACATTCTCTCTCTCATTTTGTTAACTTAGACAACCTGAGAGCTAATAA
cac taa
AGGTTCATTGCCTATTAATGTTATAGTTTTTGATGGTAAATCAAAATGTGA

28107 3 3 0 4 ORF8-Q72del
CACCTTTAATTGAATTGTGCGTGGATGAGGCTGGTTCTAAATCACCCATT
cag
TACATCGATATCGGTAATTATACAGTTTCCTGTTTACCTTTTACAATTAAT

28915 3 3 0 4 N-G215del
ACTCCAGGCAGCAGTAGGGGAACTTCTCCTGCTAGAATGGCTGGCAATGG
cgg
TGATGCTGCTCTTGCTTTGCTGCTGCTTGACAGATTGAACCAGCTTGAGAG

22740 3 3 0 4 S-T393del
TTTTAAGTGTTATGGAGTGTCTCCTACTAAATTAAATGATCTCTGCTTTA
cta
ATGTCTATGCAGATTCATTTGTAATTAGAGGTGATGAAGTCAGACAAATCG

21992 1 3 0 4 S-Y144fs
TTATTAAAGTCTGTGAATTTCAATTTTGTAATGATCCATTTTTGGGTGTT
t
ATTACCACAAAAACAACAAAAGTTGGATGGAAAGTGAGTTCAGAGTTTATT

13937 3 3 0 4 ORF1b-V157del
CAATTGTTGTGATGATGATTATTTCAATAAAAAGGACTGGTATGATTTTG
tag
AAAACCCAGATATATTACGCGTATACGCCAACTTAGGTGAACGTGTACGCC

27768 6 3 0 4 ORF7b-SL5del
TTTATAACACTTTGCTTCACACTCAAAAGAAAGACAGAATGATTGAACTT
tca tta
ATTGACTTCTATTTGTGCTTTTTAGCCTTTCTGCTATTCCTTGTTTTAATT

25393 1 2 0 4 ORF3a-M1fs
TCTGAGCCAGTGCTCAAAGGAGTCAAATTACATTACACATAAACGAACTT
a
TGGATTTGTTTATGAGAATCTTCACAATTGGAACTGTAACTTTGAAGCAAG

27774 1 2 0 4 ORF7b-I7fs
ACACTTTGCTTCACACTCAAAAGAAAGACAGAATGATTGAACTTTCATTA
a
TTGACTTCTATTTGTGCTTTTTAGCCTTTCTGCTATTCCTTGTTTTAATTA

28065 1 2 0 4 ORF8-I58fs
TCTATTCTAAATGGTATATTAGAGTAGGAGCTAGAAAATCAGCACCTTTA
a
TTGAATTGTGCGTGGATGAGGCTGGTTCTAAATCACCCATTCAGTACATCG

28910 2 2 0 4 N-N213fs
ATTCAACTCCAGGCAGCAGTAGGGGAACTTCTCCTGCTAGAATGGCTGGC
aa
TGGCGGTGATGCTGCTCTTGCTTTGCTGCTGCTTGACAGATTGAACCAGCT

3341 6 2 0 4 ORF1a-NS1026del
AACCTCAATTAGAGATGGAACTTACACCAGTTGTTCAGACTATTGAAGTG
aat agt
TTTAGTGGTTATTTAAAACTTACTGACAATGTATACATTAAAAATGCAGAC

28158 4 2 0 4 ORF8-NC89fs
AGTACATCGATATCGGTAATTATACAGTTTCCTGTTTACCTTTTACAATT
aat t
GCCAGGAACCTAAATTGGGTAGTCTTGTAGTGCGTTGTTCGTTCTATGAAG

29814 6 2 0 4
GTGAACAATGCTAGGGAGAGCTGCCTATATGGAAGAGCCCTAATGTGTAA
aat taa
TTTTAGTAGTGCTATCCCCATGTGATTTTAATAGCTTCTTAGGAGAATGAC

6524 6 2 0 4 ORF1a-TE2087del
AAGTTGTAGGAGACATTATACTTAAACCAGCAAATAATAGTTTAAAAATT
aca gaa
GAGGTTGGCCACACAGATCTAATGGCTGCTTATGTAGACAATTCTAGTCTT

27636 3 2 0 4 ORF7a-V82del
TTTGCTTGTCCTGACGGCGTAAAACACGTCTATCAGTTACGTGCCAGATC
agt

TTCACCTAAACTGTTCATCAGACAAGAGGAAGTTCAAGAACTTTACTCTCC

27633 2 2 0 4 ORF7a-R80fs
GCTTTTGCTTGTCCTGACGGCGTAAAACACGTCTATCAGTTACGTGCCAG
at
CAGTTTCACCTAAACTGTTCATCAGACAAGAGGAAGTTCAAGAACTTTACT

11586 3 2 0 4 ORF1a-N3774del
TATTGTTTTTATGTGTGTTGAGTATTGCCCTATTTTCTTCATAACTGGTA
ata
CACTTCAGTGTATAATGCTAGTTTATTGTTTCTTAGGCTATTTTTGTACTT

27371 1 2 0 4 ORF6-P57fs
ATCTAAGTCACTAACTGAGAATAAATATTCTCAATTAGATGAAGAGCAAC
c
AATGGAGATTGATTAAACGAACATGAAAATTATTCTTTTCTTGGCACTGAT

27903 1 2 0 4 ORF8-L4fs
AACTGCAAGATCATAATGAAACTTGTCACGCCTAAACGAACATGAAATTT
c
TTGTTTTCTTAGGAATCATCACAACTGTAGCTGCATTTCACCAAGAATGTA

98 1 2 0 4
CTCTTGTAGATCTGTTCTCTAAACGAACTTTAAAATCTGTGTGGCTGTCA
c
TCGGCTGCATGCTTAGTGCACTCACGCAGTATAATTAATAACTAATTACTG

203 2 2 0 4
TTGACAGGACACGAGTAACTCGTCTATCTTCTGCAGGCTGCTTACGGTTT
cg
TCCGTGTTGCAGCCGATCATCAGCACATCTAGGTTTCGTCCGGGTGTGACC

27625 2 2 0 4 ORF7a-R78fs
CTCAATTTGCTTTTGCTTGTCCTGACGGCGTAAAACACGTCTATCAGTTA
cg
TGCCAGATCAGTTTCACCTAAACTGTTCATCAGACAAGAGGAAGTTCAAGA

25499 6 2 0 4 ORF3a-PI36del
AATCAAGGATGCTACTCCTTCAGATTTTGTTCGCGCTACTGCAACGATAC
cga tac
AAGCCTCACTCCCTTTCGGATGGCTTATTGTTGGCGTTGCACTTCTTGCTG

27804 2 2 0 4 ORF7b-L17fs
GAATGATTGAACTTTCATTAATTGACTTCTATTTGTGCTTTTTAGCCTTT
ct
GCTATTCCTTGTTTTAATTATGCTTATTATCTTTTGGTTCTCACTTGAACT

27906 1 2 0 4 ORF8-V5fs
TGCAAGATCATAATGAAACTTGTCACGCCTAAACGAACATGAAATTTCTT
g
TTTTCTTAGGAATCATCACAACTGTAGCTGCATTTCACCAAGAATGTAGTT

28090 1 2 0 4 ORF8-G66fs
AGGAGCTAGAAAATCAGCACCTTTAATTGAATTGTGCGTGGATGAGGCTG

g
TTCTAAATCACCCATTCAGTACATCGATATCGGTAATTATACAGTTTCCTG

29757 1 2 0 4
ACTTGAAAGAGCCACCACATTTTCACCGAGGCCACGCGGAGTACGATCGA
g
TGTACAGTGAACAATGCTAGGGAGAGCTGCCTATATGGAAGAGCCCTAATG

27289 4 2 0 4 ORF6-DY30fs
AGATATTACTAATTATTATGAGGACTTTTAAAGTTTCCATTTGGAATCTT
gat t
ACATCATAAACCTCATAATTAAAAATTTATCTAAGTCACTAACTGAGAATA

27628 2 2 0 4 ORF7a-A79fs
AATTTGCTTTTGCTTGTCCTGACGGCGTAAAACACGTCTATCAGTTACGT
gc
CAGATCAGTTTCACCTAAACTGTTCATCAGACAAGAGGAAGTTCAAGAACT

28916 2 2 0 4 N-G215fs
CTCCAGGCAGCAGTAGGGGAACTTCTCCTGCTAGAATGGCTGGCAATGGC
gg
TGATGCTGCTCTTGCTTTGCTGCTGCTTGACAGATTGAACCAGCTTGAGAG

29736 3 2 0 4
CAGTGTGTAACATTAGGGAGGACTTGAAAGAGCCACCACATTTTCACCGA
ggc
CACGCGGAGTACGATCGAGTGTACAGTGAACAATGCTAGGGAGAGCTGCCT

1599 6 2 0 4 ORF1a-GLN445D
CGCTAACATAGGTTGTAACCATACAGGTGTTGTTGGAGAAGGTTCCGAAG
gtc tta
ATGACAACCTTCTTGAAATACTCCAAAAAGAGAAAGTCAACATCAATATTG

27906 3 2 0 4 ORF8-V5del
TGCAAGATCATAATGAAACTTGTCACGCCTAAACGAACATGAAATTTCTT
gtt
TTCTTAGGAATCATCACAACTGTAGCTGCATTTCACCAAGAATGTAGTTTA

25520 2 2 0 4 ORF3a-F43fs
AGATTTTGTTCGCGCTACTGCAACGATACCGATACAAGCCTCACTCCCTT
tc
GGATGGCTTATTGTTGGCGTTGCACTTCTTGCTGTTTTTCAGAGCGCTTCC

27622 2 2 0 4 ORF7a-L77fs
GCACTCAATTTGCTTTTGCTTGTCCTGACGGCGTAAAACACGTCTATCAG
tt
ACGTGCCAGATCAGTTTCACCTAAACTGTTCATCAGACAAGAGGAAGTTCA

28171 6 1 0 4 ORF8-PK93del
CGGTAATTATACAGTTTCCTGTTTACCTTTTACAATTAATTGCCAGGAAC
cta aat
TGGGTAGTCTTGTAGTGCGTTGTTCGTTCTATGAAGACTTTTTAGAGTATC

29774 4 1 0 4

CATTTTCACCGAGGCCACGCGGAGTACGATCGAGTGTACAGTGAACAATG
cta g
GGAGAGCTGCCTATATGGAAGAGCCCTAATGTGTAAAATTAATTTTAGTAG

1938 3 1 0 4 ORF1a-SV558L
TCGTGTTGTACGATCAATTTTCTCCCGCACTCTTGAAACTGCTCAAAATT
ctg
TGCGTGTTTTACAGAAGGCCGCTATAACAATACTAGATGGAATTTCACAGT

22127 6 1 0 4 S-LR189del
CTCAGCCTTTTCTTATGGACCTTGAAGGAAAACAGGGTAATTTCAAAAAT
ctt agg
GAATTTGTGTTTAAGAATATTGATGGTTATTTAAAATATATTCTAAGCAC

27572 1 1 0 4 ORF7a-S60fs
TTCACCATTTCATCCTCTAGCTGATAACAAATTTGCACTGACTTGCTTTA
g
CACTCAATTTGCTTTTGCTTGTCCTGACGGCGTAAAACACGTCTATCAGTT

25855 3 1 0 4 ORF3a-D155del
CATTACTTTATGATGCCAACTATTTTCTTTGCTGGCATACTAATTGTTAC
gac
TATTGTATACCTTACAATAGTGTAACTTCTTCAATTGTCATTACTTCAGGT

21771 1 1 0 4 S-V70fs
CTTGTTCTTACCTTTCTTTTCCAATGTTACTTGGTTCCATGCTATACATG
t
CTCTGGGACCAATGGTACTAAGAGGTTTGATAACCCTGTCCTACCATTTAA

519 1 1 0 4 ORF1a-M85fs
TGTGTTCATCAAACGTTCGGATGCTCGAACTGCACCTCATGGTCATGTTA
t
GGTTGAGCTGGTAGCAGAACTCGAAGGCATTCAGTACGGTCGTAGTGGTGA

28246 2 1 0 4 ORF8-L118fs
TTGTTCGTTCTATGAAGACTTTTTAGAGTATCATGACGTTCGTGTTGTTT
ta
GATTTCATCTAAACGAACAAACTAAAATGTCTGATAATGGACCCCAAAATC

21765 5 1 0 4 S-IH68fs
TCAGGACTTGTTCTTACCTTTCTTTTCCAATGTTACTTGGTTCCATGCTA
tac at
GTCTCTGGGACCAATGGTACTAAGAGGTTTGATAACCCTGTCCTACCATTT

29748 6 1 0 4
TTAGGGAGGACTTGAAAGAGCCACCACATTTTCACCGAGGCCACGCGGAG
tac gat
CGAGTGTACAGTGAACAATGCTAGGGAGAGCTGCCTATATGGAAGAGCCCT

28206 4 1 0 4 ORF8-YE105fs
TTAATTGCCAGGAACCTAAATTGGGTAGTCTTGTAGTGCGTTGTTCGTTC
tat g
AAGACTTTTTAGAGTATCATGACGTTCGTGTTGTTTTAGATTTCATCTAAA

27293 1 3 0 3 ORF6-Y31fs
ATTACTAATTATTATGAGGACTTTTAAAGTTTCCATTTGGAATCTTGATT
a
CATCATAAACCTCATAATTAAAAATTTATCTAAGTCACTAACTGAGAATAA

29860 1 3 0 3
GTAAAATTAATTTTAGTAGTGCTATCCCCATGTGATTTTAATAGCTTCTT
a
GGAGAATGACAAAAAAAAAAAAAAAAAAAAAAAAAAAAAAAAAAA

29869 1 3 0 3
ATTTTAGTAGTGCTATCCCCATGTGATTTTAATAGCTTCTTAGGAGAATG
a
CAAAAAAAAAAAAAAAAAAAAAAAAAAAAAAAAAAA

29767 2 3 0 3
GCCACCACATTTTCACCGAGGCCACGCGGAGTACGATCGAGTGTACAGTG
aa
CAATGCTAGGGAGAGCTGCCTATATGGAAGAGCCCTAATGTGTAAAATTAA

3672 6 3 0 3 ORF1a-ENF1136V
CCCAAATGTTAACAAAGGTGAAGACATTCAACTTCTTAAGAGTGCTTATG
aaa att
TTAATCAGCACGAAGTTCTACTTGCACCATTATTATCAGCTGGTATTTTTG

29768 4 3 0 3
CCACCACATTTTCACCGAGGCCACGCGGAGTACGATCGAGTGTACAGTGA
aca a
TGCTAGGGAGAGCTGCCTATATGGAAGAGCCCTAATGTGTAAAATTAATTT

9859 2 3 0 3 ORF1a-L3198fs
ACCTTTTTGTTAAATAAAGAAATGTATCTAAAGTTGCGTAGTGATGTGCT
at
TACCTCTTACGCAATATAATAGATACTTAGCTCTTTATAATAAGTACAAGT

15824 1 3 0 3 ORF1b-S786fs
CATAAAGAACTTTAAGTCAGTTCTTTATTATCAAAACAATGTTTTTATGT
c
TGAAGCAAAATGTTGGACTGAGACTGACCTTACTAAAGGACCTCATGAATT

29743 5 3 0 3
TAACATTAGGGAGGACTTGAAAGAGCCACCACATTTTCACCGAGGCCACG
cgg ag
TACGATCGAGTGTACAGTGAACAATGCTAGGGAGAGCTGCCTATATGGAAG

2574 3 3 0 3 ORF1a-T770del
AGAGGAAGTTGTCTTGAAAACTGGTGATTTACAACCATTAGAACAACCTA
cta
GTGAAGCTGTTGAAGCTCCATTGGTTGGTACACCAGTTTGTATTAACGGGC

29751 1 3 0 3
GGGAGGACTTGAAAGAGCCACCACATTTTCACCGAGGCCACGCGGAGTAC
g
ATCGAGTGTACAGTGAACAATGCTAGGGAGAGCTGCCTATATGGAAGAGCC

29759 1 3 0 3
TTGAAAGAGCCACCACATTTTCACCGAGGCCACGCGGAGTACGATCGAGT
g
TACAGTGAACAATGCTAGGGAGAGCTGCCTATATGGAAGAGCCCTAATGTG

12041 6 3 0 3 ORF1a-DI3926del
AAAAAATGGTTTCACTACTTTCTGTTTTGCTTTCCATGCAGGGTGCTGTA
gac ata
AACAAGCTTTGTGAAGAAATGCTGGACAACAGGGCAACCTTACAAGCTATA

29734 6 3 0 3
ATCAGTGTGTAACATTAGGGAGGACTTGAAAGAGCCACCACATTTTCACC
gag gcc
ACGCGGAGTACGATCGAGTGTACAGTGAACAATGCTAGGGAGAGCTGCCTA

21985 6 3 0 3 S-LGV141F
AATGTTGTTATTAAAGTCTGTGAATTTCAATTTTGTAATGATCCATTTTT
ggg tgt
TTATTACCACAAAAACAACAAAAGTTGGATGGAAAGTGAGTTCAGAGTTTA

3353 3 3 0 3 ORF1a-G1030del
AGATGGAACTTACACCAGTTGTTCAGACTATTGAAGTGAATAGTTTTAGT
ggt
TATTTAAAACTTACTGACAATGTATACATTAAAAATGCAGACATTGTGGAA

25244 3 3 0 3 S-V1228del
AATGGCCATGGTACATTTGGCTAGGTTTTATAGCTGGCTTGATTGCCATA
gta
ATGGTGACAATTATGCTTTGCTGTATGACCAGTTGCTGTAGTTGTCTCAAG

25429 6 3 0 3 ORF3a-VT13del
ACATAAACGAACTTATGGATTTGTTTATGAGAATCTTCACAATTGGAACT
gta act
TTGAAGCAAGGTGAAATCAAGGATGCTACTCCTTCAGATTTTGTTCGCGCT

510 3 3 0 3 ORF1a-GH82D
ACAGCCCTATGTGTTCATCAAACGTTCGGATGCTCGAACTGCACCTCATG
gtc
ATGTTATGGTTGAGCTGGTAGCAGAACTCGAAGGCATTCAGTACGGTCGTA

27588 1 3 0 3 ORF7a-F65fs
CTAGCTGATAACAAATTTGCACTGACTTGCTTTAGCACTCAATTTGCTTT
t
GCTTGTCCTGACGGCGTAAAACACGTCTATCAGTTACGTGCCAGATCAGTT

202 2 3 0 3
GTTGACAGGACACGAGTAACTCGTCTATCTTCTGCAGGCTGCTTACGGTT
tc
GTCCGTGTTGCAGCCGATCATCAGCACATCTAGGTTTCGTCCGGGTGTGAC

27555 2 3 0 3 ORF7a-F54fs
GGAACATACGAGGGCAATTCACCATTTCATCCTCTAGCTGATAACAAATT
tg

CACTGACTTGCTTTAGCACTCAATTTGCTTTTGCTTGTCCTGACGGCGTAA

25475 2 3 0 3 ORF3a-F28fs
AACTGTAACTTTGAAGCAAGGTGAAATCAAGGATGCTACTCCTTCAGATT
tt
GTTCGCGCTACTGCAACGATACCGATACAAGCCTCACTCCCTTTCGGATGG

25532 2 3 0 3 ORF3a-I47fs
CGCTACTGCAACGATACCGATACAAGCCTCACTCCCTTTCGGATGGCTTA
tt
GTTGGCGTTGCACTTCTTGCTGTTTTTCAGAGCGCTTCCAAAATCATAACC

25829 2 3 0 3 ORF3a-F146fs
TTGCTGGAAATGCCGTTCCAAAAACCCATTACTTTATGATGCCAACTATT
tt
CTTTGCTGGCATACTAATTGTTACGACTATTGTATACCTTACAATAGTGTA

28066 2 3 0 3 ORF8-I58fs
CTATTCTAAATGGTATATTAGAGTAGGAGCTAGAAAATCAGCACCTTTAA
tt
GAATTGTGCGTGGATGAGGCTGGTTCTAAATCACCCATTCAGTACATCGAT

27553 3 3 0 3 ORF7a-F54del
CTGGAACATACGAGGGCAATTCACCATTTCATCCTCTAGCTGATAACAAA
ttt
GCACTGACTTGCTTTAGCACTCAATTTGCTTTTGCTTGTCCTGACGGCGTA

11293 4 3 0 3 ORF1a-GF3676fs
GTGATGCGTATTATGACATGGTTGGATATGGTTGATACTAGTTTGTCTGG
ttt t
AAGCTAAAAGACTGTGTTATGTATGCATCAGCTGTAGTGTTACTAATCCTT

139 1 2 0 3
TGGCTGTCACTCGGCTGCATGCTTAGTGCACTCACGCAGTATAATTAATA
a
CTAATTACTGTCGTTGACAGGACACGAGTAACTCGTCTATCTTCTGCAGGC

21611 3 2 0 3 S-N17del
CAATGTTTGTTTTTCTTGTTTTATTGCCACTAGTCTCTAGTCAGTGTGTT
aat
CTTACAACCAGAACTCAATTACCCCCTGCATACACTAATTCTTTCACACGT

27578 3 2 0 3 ORF7a-QF62L
ATTTCATCCTCTAGCTGATAACAAATTTGCACTGACTTGCTTTAGCACTC
aat
TTGCTTTTGCTTGTCCTGACGGCGTAAAACACGTCTATCAGTTACGTGCCA

22193 6 2 0 3 S-NL211del
TTAAGAATATTGATGGTTATTTTAAAATATATTCTAAGCACACGCCTATT
aat tta
GTGCGTGATCTCCCTCAGGGTTTTTCGGCTTTAGAACCATTGGTAGATTTG

19865 3 2 0 3 ORF1b-N2133del
AGTACCAGAGGTGAAAATACTCAATAATTTGGGTGTGGACATTGCTGCTA

file:///C|/...ers/Yurchenko.MS/OneDrive%20-%20Ostravska%20univerzita/Papers%20in%20progress/Rogozin%20Deletions/Fig%20S1.txt[12/21/2022 10:57:53 AM]

ata
CTGTGATCTGGGACTACAAAAGAGATGCTCCAGCACATATATCTACTATTG

29815 5 2 0 3
TGAACAATGCTAGGGAGAGCTGCCTATATGGAAGAGCCCTAATGTGTAAA
att aa
TTTTAGTAGTGCTATCCCCATGTGATTTTAATAGCTTCTTAGGAGAATGAC

29857 1 2 0 3
TGTGTAAAATTAATTTTAGTAGTGCTATCCCCATGTGATTTTAATAGCTT
c
TTAGGAGAATGACAAAAAAAAAAAAAAAAAAAAAAAAAAAAAAAAAAAA

697 1 2 0 3 ORF1a-D144fs
AATAAAGGAGCTGGTGGCCATAGTTACGGCGCCGATCTAAAGTCATTTGA
c
TTAGGCGACGAGCTTGGCACTGATCCTTATGAAGATTTTCAAGAAAACTGG

12789 3 2 0 3 ORF1a-T4175del
TACACAAACTGCTTGCACTGATGACAATGCGTTAGCTTACTACAACACAA
caa
AGGGAGGTAGGTTTGTACTTGCACTGTTATCCGATTTACAGGATTTGAAAT

29730 4 2 0 3
TTTAATCAGTGTGTAACATTAGGGAGGACTTGAAAGAGCCACCACATTTT
cac c
GAGGCCACGCGGAGTACGATCGAGTGTACAGTGAACAATGCTAGGGAGAGC

29686 4 2 0 3
ATAGCACAAGTAGATGTAGTTAACTTTAATCTCACATAGCAATCTTTAAT
cag t
GTGTAACATTAGGGAGGACTTGAAAGAGCCACCACATTTTCACCGAGGCCA

29733 2 2 0 3
AATCAGTGTGTAACATTAGGGAGGACTTGAAAGAGCCACCACATTTTCAC
cg
AGGCCACGCGGAGTACGATCGAGTGTACAGTGAACAATGCTAGGGAGAGCT

28236 6 2 0 3 ORF8-RV115del
TTGTAGTGCGTTGTTCGTTCTATGAAGACTTTTTAGAGTATCATGACGTT
cgt gtt
GTTTTAGATTTCATCTAAACGAACAAACTAAAATGTCTGATAATGGACCCC

27434 3 2 0 3 ORF7a-TC14S
TTAAACGAACATGAAAATTATTCTTTTCTTGGCACTGATAACACTCGCTA
ctt
GTGAGCTTTATCACTACCAAGAGTGTGTTAGAGGTACAACAGTACTTTTAA

27877 1 2 0 3 ORF7b-C41fs
GCTTATTATCTTTTGGTTCTCACTTGAACTGCAAGATCATAATGAAACTT
g
TCACGCCTAAACGAACATGAAATTTCTTGTTTTCTTAGGAATCATCACAAC

28914 1 2 0 3 N-G214fs

AACTCCAGGCAGCAGTAGGGGAACTTCTCCTGCTAGAATGGCTGGCAATG
g
CGGTGATGCTGCTCTTGCTTTGCTGCTGCTTGACAGATTGAACCAGCTTGA

28248 2 2 0 3 ORF8-D119fs
GTTCGTTCTATGAAGACTTTTTAGAGTATCATGACGTTCGTGTTGTTTTA
ga
TTTCATCTAAACGAACAAACTAAAATGTCTGATAATGGACCCCAAAATCAG

25424 6 2 0 3 ORF3a-GT11del
ATTACACATAAACGAACTTATGGATTTGTTTATGAGAATCTTCACAATTG
gaa ctg
TAACTTTGAAGCAAGGTGAAATCAAGGATGCTACTCCTTCAGATTTTGTTC

28907 5 2 0 3 N-GN212fs
GAAATTCAACTCCAGGCAGCAGTAGGGGAACTTCTCCTGCTAGAATGGCT
ggc aa
TGGCGGTGATGCTGCTCTTGCTTTGCTGCTGCTTGACAGATTGAACCAGCT

21538 3 2 0 3 ORF1b-V2691del
AAGGTAGACTTATAATTAGAGAAAACAACAGAGTTGTTATTTCTAGTGAT
gtt
CTTGTTAACAACTAAACGAACAATGTTTGTTTTTCTTGTTTTATTGCCACT

25650 3 2 0 3 ORF3a-L86del
CAACTAGCACTCTCCAAGGGTGTTCACTTTGTTTGCAACTTGCTGTTGTT
gtt
TGTAACAGTTTACTCACACCTTTTGCTCGTTGCTGCTGGCCTTGAAGCCCC

21543 1 2 0 3 ORF1b-L2692fs
AGACTTATAATTAGAGAAAACAACAGAGTTGTTATTTCTAGTGATGTTCT
t
GTTAACAACTAAACGAACAATGTTTGTTTTTCTTGTTTTATTGCCACTAGT

21775 1 2 0 3 S-S71fs
TTCTTACCTTTCTTTTCCAATGTTACTTGGTTCCATGCTATACATGTCTC
t
GGGACCAATGGTACTAAGAGGTTTGATAACCCTGTCCTACCATTTAATGAT

28094 1 2 0 3 ORF8-S67fs
GCTAGAAAATCAGCACCTTTAATTGAATTGTGCGTGGATGAGGCTGGTTC
t
AAATCACCCATTCAGTACATCGATATCGGTAATTATACAGTTTCCTGTTTA

28257 1 2 0 3 ORF8-*122fs
ATGAAGACTTTTTAGAGTATCATGACGTTCGTGTTGTTTTAGATTTCATC
t
AAACGAACAAACTAAAATGTCTGATAATGGACCCCAAAATCAGCGAAATGC

29772 1 2 0 3
CACATTTTCACCGAGGCCACGCGGAGTACGATCGAGTGTACAGTGAACAA
t
GCTAGGGAGAGCTGCCTATATGGAAGAGCCCTAATGTGTAAAATTAATTTT

27299 2 2 0 3 ORF6-I33fs
AATTATTATGAGGACTTTTAAAGTTTCCATTTGGAATCTTGATTACATCA
ta
AACCTCATAATTAAAAATTTATCTAAGTCACTAACTGAGAATAAATATTCT

27570 2 2 0 3 ORF7a-F59fs
AATTCACCATTTCATCCTCTAGCTGATAACAAATTTGCACTGACTTGCTT
ta
GCACTCAATTTGCTTTTGCTTGTCCTGACGGCGTAAAACACGTCTATCAGT

28063 2 2 0 3 ORF8-L57fs
CTTCTATTCTAAATGGTATATTAGAGTAGGAGCTAGAAAATCAGCACCTT
ta
ATTGAATTGTGCGTGGATGAGGCTGGTTCTAAATCACCCATTCAGTACATC

21864 3 2 0 3 S-I101del
ACCATTTAATGATGGTGTTTATTTTGCTTCCACTGAGAAGTCTAACATAA
taa
GAGGCTGGATTTTTGGTACTACTTTAGATTCGAAGACCCAGTCCCTACTTA

22192 6 2 0 3 S-NL211del
TTTAAGAATATTGATGGTTATTTTAAAATATATTCTAAGCACACGCCTAT
taa ttt
AGTGCGTGATCTCCCTCAGGGTTTTTCGGCTTTAGAACCATTGGTAGATTT

27720 3 2 0 3 ORF7a-FI109L
GTTCAAGAACTTTACTCTCCAATTTTTCTTATTGTTGCGGCAATAGTGTT
tat
AACACTTTGCTTCACACTCAAAAGAAAGACAGAATGATTGAACTTTCATTA

689 2 2 0 3 ORF1a-S142fs
AGAACGGTAATAAAGGAGCTGGTGGCCATAGTTACGGCGCCGATCTAAAG
tc
ATTTGACTTAGGCGACGAGCTTGGCACTGATCCTTATGAAGATTTTCAAGA

224 3 2 0 3
GTCTATCTTCTGCAGGCTGCTTACGGTTTCGTCCGTGTTGCAGCCGATCA
tca
GCACATCTAGGTTTCGTCCGGGTGTGACCGAAAGGTAAGATGGAGAGCCTT

21534 2 2 0 3 ORF1b-S2689fs
AGTAAAGGTAGACTTATAATTAGAGAAAACAACAGAGTTGTTATTTCTAG
tg
ATGTTCTTGTTAACAACTAAACGAACAATGTTTGTTTTTCTTGTTTTATTG

28241 6 2 0 3 ORF8-VL117del
GTGCGTTGTTCGTTCTATGAAGACTTTTTAGAGTATCATGACGTTCGTGT
tgt ttt
AGATTTCATCTAAACGAACAAACTAAAATGTCTGATAATGGACCCCAAAAT

2586 3 2 0 3 ORF1a-V774del
CTTGAAAACTGGTGATTTACAACCATTAGAACAACCTACTAGTGAAGCTG
ttg
AAGCTCCATTGGTTGGTACACCAGTTTGTATTAACGGGCTTATGTTGCTCG

692 4 2 0 3 ORF1a-FD143fs
ACGGTAATAAAGGAGCTGGTGGCCATAGTTACGGCGCCGATCTAAAGTCA
ttt g
ACTTAGGCGACGAGCTTGGCACTGATCCTTATGAAGATTTTCAAGAAAACT

28216 5 2 0 3 ORF8-FL108fs
GGAACCTAAATTGGGTAGTCTTGTAGTGCGTTGTTCGTTCTATGAAGACT
ttt ta
GAGTATCATGACGTTCGTGTTGTTTTAGATTTCATCTAAACGAACAAACTA

27927 1 1 0 3 ORF8-T12fs
GTCACGCCTAAACGAACATGAAATTTCTTGTTTTCTTAGGAATCATCACA
a
CTGTAGCTGCATTTCACCAAGAATGTAGTTTACAGTCATGTACTCAACATC

28228 1 1 0 3 ORF8-H112fs
GGGTAGTCTTGTAGTGCGTTGTTCGTTCTATGAAGACTTTTTAGAGTATC
a
TGACGTTCGTGTTGTTTTAGATTTCATCTAAACGAACAAACTAAAATGTCT

27763 6 1 0 3 ORF7b-ELS3A
TAGTGTTTATAACACTTTGCTTCACACTCAAAAGAAAGACAGAATGATTG
aac ttt
CATTAATTGACTTCTATTTGTGCTTTTTAGCCTTTCTGCTATTCCTTGTTT

28910 6 1 0 3 N-NG213del
ATTCAACTCCAGGCAGCAGTAGGGGAACTTCTCCTGCTAGAATGGCTGGC
aat ggc
GGTGATGCTGCTCTTGCTTTGCTGCTGCTTGACAGATTGAACCAGCTTGAG

29731 2 1 0 3
TTAATCAGTGTGTAACATTAGGGAGGACTTGAAAGAGCCACCACATTTTC
ac
CGAGGCCACGCGGAGTACGATCGAGTGTACAGTGAACAATGCTAGGGAGAG

28231 3 1 0 3 ORF8-D113del
TAGTCTTGTAGTGCGTTGTTCGTTCTATGAAGACTTTTTAGAGTATCATG
acg
TTCGTGTTGTTTTAGATTTCATCTAAACGAACAAACTAAAATGTCTGATAA

27574 3 1 0 3 ORF7a-T61del
CACCATTTCATCCTCTAGCTGATAACAAATTTGCACTGACTTGCTTTAGC
act
CAATTTGCTTTTGCTTGTCCTGACGGCGTAAAACACGTCTATCAGTTACGT

27571 2 1 0 3 ORF7a-S60fs
ATTCACCATTTCATCCTCTAGCTGATAACAAATTTGCACTGACTTGCTTT
ag
CACTCAATTTGCTTTTGCTTGTCCTGACGGCGTAAAACACGTCTATCAGTT

28190 2 1 0 3 ORF8-V99fs
TGTTTACCTTTTACAATTAATTGCCAGGAACCTAAATTGGGTAGTCTTGT
ag

TGCGTTGTTCGTTCTATGAAGACTTTTTAGAGTATCATGACGTTCGTGTTG

29763 2 1 0 3
AAGAGCCACCACATTTTCACCGAGGCCACGCGGAGTACGATCGAGTGTAC
ag
TGAACAATGCTAGGGAGAGCTGCCTATATGGAAGAGCCCTAATGTGTAAAA

29756 6 1 0 3
GACTTGAAAGAGCCACCACATTTTCACCGAGGCCACGCGGAGTACGATCG
agt gta
CAGTGAACAATGCTAGGGAGAGCTGCCTATATGGAAGAGCCCTAATGTGTA

1850 3 1 0 3 ORF1a-I529del
CAAAAGGAAAAGCTAAAAAAGGTGCCTGGAATATTGGTGAACAGAAATCA
ata
CTGAGTCCTCTTTATGCATTTGCATCAGAGGCTGCTCGTGTTGTACGATCA

27403 3 1 0 3 ORF7a-I4del
AATTAGATGAAGAGCAACCAATGGAGATTGATTAAACGAACATGAAAATT
att
CTTTTCTTGGCACTGATAACACTCGCTACTTGTGAGCTTTATCACTACCAA

8568 3 1 0 3 ORF1a-NW2768R
TAATGTTGTAACAACAAAGATAGCACTTAAGGGTGGTAAAATTGTTAATA
att
GGTTGAAGCAGTTAATTAAAGTTACACTTGTGTTCCTTTTTGTTGCTGCTA

27700 4 1 0 3 ORF7a-IV103fs
TGTTCATCAGACAAGAGGAAGTTCAAGAACTTTACTCTCCAATTTTTCTT
att g
TTGCGGCAATAGTGTTTATAACACTTTGCTTCACACTCAAAAGAAAGACAG

27552 4 1 0 3 ORF7a-KF53fs
TCTGGAACATACGAGGGCAATTCACCATTTCATCCTCTAGCTGATAACAA
att t
GCACTGACTTGCTTTAGCACTCAATTTGCTTTTGCTTGTCCTGACGGCGTA

29725 5 1 0 3
CAATCTTTAATCAGTGTGTAACATTAGGGAGGACTTGAAAGAGCCACCAC
att tt
CACCGAGGCCACGCGGAGTACGATCGAGTGTACAGTGAACAATGCTAGGGA

25350 1 1 0 3 S-P1263fs
CTGTTGTTCTTGTGGATCCTGCTGCAAATTTGATGAAGACGACTCTGAGC
c
AGTGCTCAAAGGAGTCAAATTACATTACACATAAACGAACTTATGGATTTG

28201 1 1 0 3 ORF8-S103fs
TACAATTAATTGCCAGGAACCTAAATTGGGTAGTCTTGTAGTGCGTTGTT
c
GTTCTATGAAGACTTTTTAGAGTATCATGACGTTCGTGTTGTTTTAGATTT

29686 1 1 0 3
ATAGCACAAGTAGATGTAGTTAACTTTAATCTCACATAGCAATCTTTAAT

c
AGTGTGTAACATTAGGGAGGACTTGAAAGAGCCACCACATTTTCACCGAGG

27577 2 1 0 3 ORF7a-Q62fs
CATTTCATCCTCTAGCTGATAACAAATTTGCACTGACTTGCTTTAGCACT
ca
ATTTGCTTTTGCTTGTCCTGACGGCGTAAAACACGTCTATCAGTTACGTGC

29732 3 1 0 3
TAATCAGTGTGTAACATTAGGGAGGACTTGAAAGAGCCACCACATTTTCA
ccg
AGGCCACGCGGAGTACGATCGAGTGTACAGTGAACAATGCTAGGGAGAGCT

29754 6 1 0 3
AGGACTTGAAAGAGCCACCACATTTTCACCGAGGCCACGCGGAGTACGAT
cga gtg
TACAGTGAACAATGCTAGGGAGAGCTGCCTATATGGAAGAGCCCTAATGTG

28171 4 1 0 3 ORF8-PK93fs
CGGTAATTATACAGTTTCCTGTTTACCTTTTACAATTAATTGCCAGGAAC
cta a
ATTGGGTAGTCTTGTAGTGCGTTGTTCGTTCTATGAAGACTTTTTAGAGTA

29774 5 1 0 3
CATTTTCACCGAGGCCACGCGGAGTACGATCGAGTGTACAGTGAACAATG
cta gg
GAGAGCTGCCTATATGGAAGAGCCCTAATGTGTAAAATTAATTTTAGTAGT

28205 4 1 0 3 ORF8-FY104fs
ATTAATTGCCAGGAACCTAAATTGGGTAGTCTTGTAGTGCGTTGTTCGTT
cta t
GAAGACTTTTTAGAGTATCATGACGTTCGTGTTGTTTTAGATTTCATCTAA

27684 6 1 0 3 ORF7a-YSP97*
TCAGTTTCACCTAAACTGTTCATCAGACAAGAGGAAGTTCAAGAACTTTA
ctc tcc
AATTTTTCTTATTGTTGCGGCAATAGTGTTTATAACACTTTGCTTCACACT

28139 6 1 0 3 ORF8-CL83del
GGTTCTAAATCACCCATTCAGTACATCGATATCGGTAATTATACAGTTTC
ctg ttt
ACCTTTTACAATTAATTGCCAGGAACCTAAATTGGGTAGTCTTGTAGTGCG

26493 1 1 0 3
TCTTCTAGAGTTCCTGATCTTCTGGTCTAAACGAACTAAATATTATATTA
g
TTTTTCTGTTTGGAACTTTAATTTTAGCCATGGCAGATTCCAACGGTACTA

28193 1 1 0 3 ORF8-V100fs
TTACCTTTTACAATTAATTGCCAGGAACCTAAATTGGGTAGTCTTGTAGT
g
CGTTGTTCGTTCTATGAAGACTTTTTAGAGTATCATGACGTTCGTGTTGTT

29766 5 1 0 3

AGCCACCACATTTTCACCGAGGCCACGCGGAGTACGATCGAGTGTACAGT
gaa ca
ATGCTAGGGAGAGCTGCCTATATGGAAGAGCCCTAATGTGTAAAATTAATT

3800 3 1 0 3 ORF1a-D1179del
TAAGAGTTTGTGTAGATACTGTTCGCACAAATGTCTACTTAGCTGTCTTT
gat
AAAAATCTCTATGACAAACTTGTTTCAAGCTTTTTGGAAATGAAGAGTGAA

29717 3 1 0 3
TCACATAGCAATCTTTAATCAGTGTGTAACATTAGGGAGGACTTGAAAGA
gcc
ACCACATTTTCACCGAGGCCACGCGGAGTACGATCGAGTGTACAGTGAACA

27798 5 1 0 3 ORF7b-AF15fs
AAGACAGAATGATTGAACTTTCATTAATTGACTTCTATTTGTGCTTTTTA
gcc tt
TCTGCTATTCCTTGTTTTAATTATGCTTATTATCTTTTGGTTCTCACTTGA

27589 5 1 0 3 ORF7a-AC66fs
TAGCTGATAACAAATTTGCACTGACTTGCTTTAGCACTCAATTTGCTTTT
gct tg
TCCTGACGGCGTAAAACACGTCTATCAGTTACGTGCCAGATCAGTTTCACC

28179 3 1 0 3 ORF8-G96del
ATACAGTTTCCTGTTTACCTTTTACAATTAATTGCCAGGAACCTAAATTG
ggt
AGTCTTGTAGTGCGTTGTTCGTTCTATGAAGACTTTTTAGAGTATCATGAC

28183 2 1 0 3 ORF8-S97fs
AGTTTCCTGTTTACCTTTTACAATTAATTGCCAGGAACCTAAATTGGGTA
gt
CTTGTAGTGCGTTGTTCGTTCTATGAAGACTTTTTAGAGTATCATGACGTT

22113 3 1 0 3 S-GN184D
TTTTGAATATGTCTCTCAGCCTTTTCTTATGGACCTTGAAGGAAAACAGG
gta
ATTTCAAAAATCTTAGGGAATTTGTGTTTAAGAATATTGATGGTTATTTTA

28223 3 1 0 3 ORF8-EY110D
AAATTGGGTAGTCTTGTAGTGCGTTGTTCGTTCTATGAAGACTTTTTAGA
gta
TCATGACGTTCGTGTTGTTTTAGATTTCATCTAAACGAACAAACTAAAATG

27604 6 1 0 3 ORF7a-VK71del
TTGCACTGACTTGCTTTAGCACTCAATTTGCTTTTGCTTGTCCTGACGGC
gta aaa
CACGTCTATCAGTTACGTGCCAGATCAGTTTCACCTAAACTGTTCATCAGA

9854 6 1 0 3 ORF1a-VL3197del
TGTGCACCTTTTTGTTAAATAAAGAAATGTATCTAAAGTTGCGTAGTGAT
gtg cta
TTACCTCTTACGCAATATAATAGATACTTAGCTCTTTATAATAAGTACAAG

28177 1 1 0 3 ORF8-L95fs
TTATACAGTTTCCTGTTTACCTTTTACAATTAATTGCCAGGAACCTAAAT
t
GGGTAGTCTTGTAGTGCGTTGTTCGTTCTATGAAGACTTTTTAGAGTATCA

28197 1 1 0 3 ORF8-C102fs
CTTTTACAATTAATTGCCAGGAACCTAAATTGGGTAGTCTTGTAGTGCGT
t
GTTCGTTCTATGAAGACTTTTTAGAGTATCATGACGTTCGTGTTGTTTTAG

27968 2 1 0 3 ORF8-C25fs
ATCATCACAACTGTAGCTGCATTTCACCAAGAATGTAGTTTACAGTCATG
ta
CTCAACATCAACCATATGTAGTTGATGACCCGTGTCCTATTCACTTCTATT

27672 5 1 0 3 ORF7a-VQ93fs
TTACGTGCCAGATCAGTTTCACCTAAACTGTTCATCAGACAAGAGGAAGT
tca ag
AACTTTACTCTCCAATTTTTCTTATTGTTGCGGCAATAGTGTTTATAACAC

28067 6 1 0 3 ORF8-IEL58M
TATTCTAAATGGTATATTAGAGTAGGAGCTAGAAAATCAGCACCTTTAAT
tga att
GTGCGTGGATGAGGCTGGTTCTAAATCACCCATTCAGTACATCGATATCGG

11271 6 1 0 3 ORF1a-MVD3669N
GGTCTATATGCCTGCTAGTTGGGTGATGCGTATTATGACATGGTTGGATA
tgg ttg
ATACTAGTTTGTCTGGTTTTAAGCTAAAAGACTGTGTTATGTATGCATCAG

28186 2 1 0 3 ORF8-L98fs
TTCCTGTTTACCTTTTACAATTAATTGCCAGGAACCTAAATTGGGTAGTC
tt
GTAGTGCGTTGTTCGTTCTATGAAGACTTTTTAGAGTATCATGACGTTCGT

27771 3 1 0 3 ORF7b-L6del
ATAACACTTTGCTTCACACTCAAAAGAAAGACAGAATGATTGAACTTTCA
tta
ATTGACTTCTATTTGTGCTTTTTAGCCTTTCTGCTATTCCTTGTTTTAATT

28091 6 1 0 3 ORF8-KS68del
GGAGCTAGAAAATCAGCACCTTTAATTGAATTGTGCGTGGATGAGGCTGG
ttc taa
ATCACCCATTCAGTACATCGATATCGGTAATTATACAGTTTCCTGTTTACC
