# Supplementary material for: Deletions across the SARS-CoV-2 Genome: Molecular Mechanisms and Putative Functional Consequences of Deletions in Accessory Genes
Source: Microorganisms. 2023 Jan 16;11(1):229. doi: 10.3390/microorganisms11010229 (PMC9862619; doi:10.3390/microorganisms11010229)
Supplement: Supplementary file 1 [file microorganisms-11-00229-s001.zip › Figure S2.pdf]

Supplementary Figure S2. Contexts of long deletions in SARS-CoV-2.
The 1st number is the postion and the 5th number is the number of instances
followed by gene name.

27923 35 7 10 968 ORF8-ITTVAAFHQECS10fs
ACTTGTCACGCCTAAACGAACATGAAATTTCTTGTTTTCTTAGGAATCAT
cac aac tgt agc tgc att tca cca aga atg tag tt
TACAGTCATGTACTCAACATCAACCATATGTAGTTGATGACCCGTGTCCTA

27406 57 5 5 3365 ORF7a-LFLALITLATCELYHYQEC5del
TAGATGAAGAGCAACCAATGGAGATTGATTAAACGAACATGAAAATTATT
ctt ttc ttg gca ctg ata aca ctc gct act tgt gag ctt tat cac tac caa gag tgt
GTTAGAGGTACAACAGTACTTTTAAAAGAACCTTGCTCTTCTGGAACATAC

29730 37 12 4 76
TTTAATCAGTGTGTAACATTAGGGAGGACTTGAAAGAGCCACCACATTTT
cac cga ggc cac gcg gag tac gat cga gtg tac agt g
AACAATGCTAGGGAGAGCTGCCTATATGGAAGAGCCCTAATGTGTAAAATT

27579 46 12 1 144 ORF7a-QFAFACPDGVKHVYQL62fs
TTTCATCCTCTAGCTGATAACAAATTTGCACTGACTTGCTTTAGCACTCA
att tgc ttt tgc ttg tcc tga cgg cgt aaa aca cgt cta tca gtt a
CGTGCCAGATCAGTTTCACCTAAACTGTTCATCAGACAAGAGGAAGTTCAA

27569 55 2 1 90 ORF7a-FSTQFAFACPDGVKHVYQL59fs
CAATTCACCATTTCATCCTCTAGCTGATAACAAATTTGCACTGACTTGCT
tta gca ctc aat ttg ctt ttg ctt gtc ctg acg gcg taa aac acg tct atc agt t
ACGTGCCAGATCAGTTTCACCTAAACTGTTCATCAGACAAGAGGAAGTTCA

27555 69 14 1 61 ORF7a-FALTCFSTQFAFACPDGVKHVYQ54del
GGAACATACGAGGGCAATTCACCATTTCATCCTCTAGCTGATAACAAATT
tgc act gac ttg ctt tag cac tca att tgc ttt tgc ttg tcc tga cgg cgt aaa aca cgt cta tca gtt
ACGTGCCAGATCAGTTTCACCTAAACTGTTCATCAGACAAGAGGAAGTTCA

29732 35 2 1 33
TAATCAGTGTGTAACATTAGGGAGGACTTGAAAGAGCCACCACATTTTCA
ccg agg cca cgc gga gta cga tcg agt gta cag tg
AACAATGCTAGGGAGAGCTGCCTATATGGAAGAGCCCTAATGTGTAAAATT

29728 49 7 30 204
TCTTTAATCAGTGTGTAACATTAGGGAGGACTTGAAAGAGCCACCACATT
ttc acc gag gcc acg cgg agt acg atc gag tgt aca gtg aac aat gct a
GGGAGAGCTGCCTATATGGAAGAGCCCTAATGTGTAAAATTAATTTTAGTA

27531 152 1 1 15 ORF7a-FHPLADNKFALTCFSTQFAFACPDGVKHVYQLRARSVSPKLFIRQ
CTTTTAAAAGAACCTTGCTCTTCTGGAACATACGAGGGCAATTCACCATT
tca tcc tct agc tga taa caa att tgc act gac ttg ctt tag cac tca att tgc ttt tgc ttg tcc tga cgg cgt aaa aca cgt cta tca gtt acg tgc
cag atc agt ttc acc taa act gtt cat cag aca aga gga agt tca aga act tt
ACTCTCCAATTTTTCTTATTGTTGCGGCAATAGTGTTTATAACACTTTGCT

28009 137 3 1 12 ORF8-IHFYSKWYIRVGARKSAPLIELCVDEAGSKSPIQYIDIGNYTVSCL
ACAGTCATGTACTCAACATCAACCATATGTAGTTGATGACCCGTGTCCTA

ttc act tct att cta aat ggt ata tta gag tag gag cta gaa aat cag cac ctt taa ttg aat tgt gcg tgg atg agg ctg gtt cta aat cac cca ttc agt aca tcg ata tcg gta att ata cag ttt cct gtt ta
CCTTTTACAATTAATTGCCAGGAACCTAAATTGGGTAGTCTTGTAGTGCGT

27686 62 4 1 6 ORF7a-SPIFLIVAAIVFITLCFTLKR98fs
AGTTTCACCTAAACTGTTCATCAGACAAGAGGAAGTTCAAGAACTTTACT
ctc caa ttt ttc tta ttg ttg cgg caa tag tgt tta taa cac ttt gct tca cac tca aaa ga
AAGACAGAATGATTGAACTTTCATTAATTGACTTCTATTTGTGCTTTTTAG

28004 76 1 1 5 ORF8-CPIHFYSKWYIRVGARKSAPLIELCV37fs
AGTTTACAGTCATGTACTCAACATCAACCATATGTAGTTGATGACCCGTG
tcc tat tca ctt cta ttc taa atg gta tat tag agt agg agc tag aaa atc agc acc ttt aat tga att gtg cgt g
GATGAGGCTGGTTCTAAATCACCCATTCAGTACATCGATATCGGTAATTAT

27673 78 1 0 281 ORF7a-QELYSPIFLIVAAIVFITLCFTLKRK94del
TACGTGCCAGATCAGTTTCACCTAAACTGTTCATCAGACAAGAGGAAGTT
caa gaa ctt tac tct cca att ttt ctt att gtt gcg gca ata gtg ttt ata aca ctt tgc ttc aca ctc aaa aga aag
ACAGAATGATTGAACTTTCATTAATTGACTTCTATTTGTGCTTTTTAGCCT

27555 92 13 0 200 ORF7a-FALTCFSTQFAFACPDGVKHVYQLRARSVSP54fs
GGAACATACGAGGGCAATTCACCATTTCATCCTCTAGCTGATAACAAATT
tgc act gac ttg ctt tag cac tca att tgc ttt tgc ttg tcc tga cgg cgt aaa aca cgt cta tca gtt acg tgc cag atc agt ttc acc ta
AACTGTTCATCAGACAAGAGGAAGTTCAAGAACTTTACTCTCCAATTTTTC

29731 44 9 0 143
TTAATCAGTGTGTAACATTAGGGAGGACTTGAAAGAGCCACCACATTTTC
acc gag gcc acg cgg agt acg atc gag tgt aca gtg aac aat gc
TAGGGAGAGCTGCCTATATGGAAGAGCCCTAATGTGTAAAATTAATTTTAG

27555 52 3 0 54 ORF7a-FALTCFSTQFAFACPDGV54fs
GGAACATACGAGGGCAATTCACCATTTCATCCTCTAGCTGATAACAAATT
tgc act gac ttg ctt tag cac tca att tgc ttt tgc ttg tcc tga cgg cgt a
AAACACGTCTATCAGTTACGTGCCAGATCAGTTTCACCTAAACTGTTCATC

27554 92 2 0 41 ORF7a-FALTCFSTQFAFACPDGVKHVYQLRARSVSP54fs
TGGAACATACGAGGGCAATTCACCATTTCATCCTCTAGCTGATAACAAAT
ttg cac tga ctt gct tta gca ctc aat ttg ctt ttg ctt gtc ctg acg gcg taa aac acg tct atc agt tac gtg cca gat cag ttt cac ct
AAACTGTTCATCAGACAAGAGGAAGTTCAAGAACTTTACTCTCCAATTTTT

27555 51 1 0 39 ORF7a-FALTCFSTQFAFACPDGV54L
GGAACATACGAGGGCAATTCACCATTTCATCCTCTAGCTGATAACAAATT
tgc act gac ttg ctt tag cac tca att tgc ttt tgc ttg tcc tga cgg cgt
AAAACACGTCTATCAGTTACGTGCCAGATCAGTTTCACCTAAACTGTTCAT

27697 54 8 0 36 ORF7a-LIVAAIVFITLCFTLKRK102del
AACTGTTCATCAGACAAGAGGAAGTTCAAGAACTTTACTCTCCAATTTTT
ctt att gtt gcg gca ata gtg ttt ata aca ctt tgc ttc aca ctc aaa aga aag
ACAGAATGATTGAACTTTCATTAATTGACTTCTATTTGTGCTTTTTAGCCT

27579 53 1 0 35 ORF7a-QFAFACPDGVKHVYQLRA62fs
TTTCATCCTCTAGCTGATAACAAATTTGCACTGACTTGCTTTAGCACTCA
att tgc ttt tgc ttg tcc tga cgg cgt aaa aca cgt cta tca gtt acg tgc ca
GATCAGTTTCACCTAAACTGTTCATCAGACAAGAGGAAGTTCAAGAACTTT

27556 51 1 0 32 ORF7a-ALTCFSTQFAFACPDGV55del
GAACATACGAGGGCAATTCACCATTTCATCCTCTAGCTGATAACAAATTT
gca ctg act tgc ttt agc act caa ttt gct ttt gct tgt cct gac ggc gta
AAACACGTCTATCAGTTACGTGCCAGATCAGTTTCACCTAAACTGTTCATC

27684 76 1 0 26 ORF7a-YSPIFLIVAAIVFITLCFTLKRKTE*97fs
TCAGTTTCACCTAAACTGTTCATCAGACAAGAGGAAGTTCAAGAACTTTA
ctc tcc aat ttt tct tat tgt tgc ggc aat agt gtt tat aac act ttg ctt cac act caa aag aaa gac aga atg a
TTGAACTTTCATTAATTGACTTCTATTTGTGCTTTTTAGCCTTTCTGCTAT

27684 76 1 0 26 ORF7b-MI1fs
TCAGTTTCACCTAAACTGTTCATCAGACAAGAGGAAGTTCAAGAACTTTA
ctc tcc aat ttt tct tat tgt tgc ggc aat agt gtt tat aac act ttg ctt cac act caa aag aaa gac aga atg a
TTGAACTTTCATTAATTGACTTCTATTTGTGCTTTTTAGCCTTTCTGCTAT

29738 41 7 0 25
GTGTGTAACATTAGGGAGGACTTGAAAGAGCCACCACATTTTCACCGAGG
cca cgc gga gta cga tcg agt gta cag tga aca atg cta gg
GAGAGCTGCCTATATGGAAGAGCCCTAATGTGTAAAATTAATTTTAGTAGT

27556 67 2 0 23 ORF7a-ALTCFSTQFAFACPDGVKHVYQL55fs
GAACATACGAGGGCAATTCACCATTTCATCCTCTAGCTGATAACAAATTT
gca ctg act tgc ttt agc act caa ttt gct ttt gct tgt cct gac ggc gta aaa cac gtc tat cag t
TACGTGCCAGATCAGTTTCACCTAAACTGTTCATCAGACAAGAGGAAGTTC

27555 44 3 0 22 ORF7a-FALTCFSTQFAFACP54fs
GGAACATACGAGGGCAATTCACCATTTCATCCTCTAGCTGATAACAAATT
tgc act gac ttg ctt tag cac tca att tgc ttt tgc ttg tcc tg
ACGGCGTAAAACACGTCTATCAGTTACGTGCCAGATCAGTTTCACCTAAAC

27556 85 9 0 20 ORF7a-ALTCFSTQFAFACPDGVKHVYQLRARSVS55fs
GAACATACGAGGGCAATTCACCATTTCATCCTCTAGCTGATAACAAATTT
gca ctg act tgc ttt agc act caa ttt gct ttt gct tgt cct gac ggc gta aaa cac gtc tat cag tta cgt gcc aga tca gtt t
CACCTAAACTGTTCATCAGACAAGAGGAAGTTCAAGAACTTTACTCTCCAA

27579 39 5 0 20 ORF7a-QFAFACPDGVKHVY62H
TTTCATCCTCTAGCTGATAACAAATTTGCACTGACTTGCTTTAGCACTCA
att tgc ttt tgc ttg tcc tga cgg cgt aaa aca cgt cta
TCAGTTACGTGCCAGATCAGTTTCACCTAAACTGTTCATCAGACAAGAGGA

27572 53 5 0 19 ORF7a-STQFAFACPDGVKHVYQL60fs
TTCACCATTTCATCCTCTAGCTGATAACAAATTTGCACTGACTTGCTTTA
gca ctc aat ttg ctt ttg ctt gtc ctg acg gcg taa aac acg tct atc agt ta
CGTGCCAGATCAGTTTCACCTAAACTGTTCATCAGACAAGAGGAAGTTCAA

28008 75 4 0 19 ORF8-IHFYSKWYIRVGARKSAPLIELCVD39del
TACAGTCATGTACTCAACATCAACCATATGTAGTTGATGACCCGTGTCCT
att cac ttc tat tct aaa tgg tat att aga gta gga gct aga aaa tca gca cct tta att gaa ttg tgc gtg gat
GAGGCTGGTTCTAAATCACCCATTCAGTACATCGATATCGGTAATTATACA

27407 54 1 0 19 ORF7a-LFLALITLATCELYHYQEC5R
AGATGAAGAGCAACCAATGGAGATTGATTAAACGAACATGAAAATTATTC
ttt tct tgg cac tga taa cac tcg cta ctt gtg agc ttt atc act acc aag agt
GTGTTAGAGGTACAACAGTACTTTTAAAAGAACCTTGCTCTTCTGGAACAT

28073 47 2 0 18 ORF8-LCVDEAGSKSPIQYID60fs
AAATGGTATATTAGAGTAGGAGCTAGAAAATCAGCACCTTTAATTGAATT
gtg cgt gga tga ggc tgg ttc taa atc acc cat tca gta cat cga ta
TCGGTAATTATACAGTTTCCTGTTTACCTTTTACAATTAATTGCCAGGAAC

29728 39 4 0 17
TCTTTAATCAGTGTGTAACATTAGGGAGGACTTGAAAGAGCCACCACATT
ttc acc gag gcc acg cgg agt acg atc gag tgt aca gtg
AACAATGCTAGGGAGAGCTGCCTATATGGAAGAGCCCTAATGTGTAAAATT

27582 42 3 0 17 ORF7a-FAFACPDGVKHVYQ63del
CATCCTCTAGCTGATAACAAATTTGCACTGACTTGCTTTAGCACTCAATT
tgc ttt tgc ttg tcc tga cgg cgt aaa aca cgt cta tca gtt
ACGTGCCAGATCAGTTTCACCTAAACTGTTCATCAGACAAGAGGAAGTTCA

27686 76 1 0 16 ORF7a-SPIFLIVAAIVFITLCFTLKRKTE*98fs
AGTTTCACCTAAACTGTTCATCAGACAAGAGGAAGTTCAAGAACTTTACT
ctc caa ttt ttc tta ttg ttg cgg caa tag tgt tta taa cac ttt gct tca cac tca aaa gaa aga cag aat gat t
GAACTTTCATTAATTGACTTCTATTTGTGCTTTTTAGCCTTTCTGCTATTC

27686 76 1 0 16 ORF7b-MI1del
AGTTTCACCTAAACTGTTCATCAGACAAGAGGAAGTTCAAGAACTTTACT
ctc caa ttt ttc tta ttg ttg cgg caa tag tgt tta taa cac ttt gct tca cac tca aaa gaa aga cag aat gat t
GAACTTTCATTAATTGACTTCTATTTGTGCTTTTTAGCCTTTCTGCTATTC

21747 39 15 0 15 S-VTWFHAIHVSGTN62del
CTCAGTTTTACATTCAACTCAGGACTTGTTCTTACCTTTCTTTTCCAATG
tta ctt ggt tcc atg cta tac atg tct ctg gga cca atg
GTACTAAGAGGTTTGATAACCCTGTCCTACCATTTAATGATGGTGTTTATT

27580 45 12 0 15 ORF7a-FAFACPDGVKHVYQL63del
TTCATCCTCTAGCTGATAACAAATTTGCACTGACTTGCTTTAGCACTCAA
ttt gct ttt gct tgt cct gac ggc gta aaa cac gtc tat cag tta
CGTGCCAGATCAGTTTCACCTAAACTGTTCATCAGACAAGAGGAAGTTCAA

29739 36 6 0 15
TGTGTAACATTAGGGAGGACTTGAAAGAGCCACCACATTTTCACCGAGGC
cac gcg gag tac gat cga gtg tac agt gaa caa tgc
TAGGGAGAGCTGCCTATATGGAAGAGCCCTAATGTGTAAAATTAATTTTAG

29731 35 1 0 15
TTAATCAGTGTGTAACATTAGGGAGGACTTGAAAGAGCCACCACATTTTC
acc gag gcc acg cgg agt acg atc gag tgt aca gt
GAACAATGCTAGGGAGAGCTGCCTATATGGAAGAGCCCTAATGTGTAAAAT

27686 73 1 0 15 ORF7a-SPIFLIVAAIVFITLCFTLKRKTE*98fs
AGTTTCACCTAAACTGTTCATCAGACAAGAGGAAGTTCAAGAACTTTACT
ctc caa ttt ttc tta ttg ttg cgg caa tag tgt tta taa cac ttt gct tca cac tca aaa gaa aga cag aat g
ATTGAACTTTCATTAATTGACTTCTATTTGTGCTTTTTAGCCTTTCTGCTA

27686 73 1 0 15 ORF7b-M1del
AGTTTCACCTAAACTGTTCATCAGACAAGAGGAAGTTCAAGAACTTTACT
ctc caa ttt ttc tta ttg ttg cgg caa tag tgt tta taa cac ttt gct tca cac tca aaa gaa aga cag aat g

ATTGAACTTTCATTAATTGACTTCTATTTGTGCTTTTTAGCCTTTCTGCTA

27536 81 1 0 15 ORF7a-PLADNKFALTCFSTQFAFACPDGVKHVY48H
AAAAGAACCTTGCTCTTCTGGAACATACGAGGGCAATTCACCATTTCATC
ctc tag ctg ata aca aat ttg cac tga ctt gct tta gca ctc aat ttg ctt ttg ctt gtc ctg acg gcg taa aac acg tct
ATCAGTTACGTGCCAGATCAGTTTCACCTAAACTGTTCATCAGACAAGAGG

27555 82 4 0 14 ORF7a-FALTCFSTQFAFACPDGVKHVYQLRARS54fs
GGAACATACGAGGGCAATTCACCATTTCATCCTCTAGCTGATAACAAATT
tgc act gac ttg ctt tag cac tca att tgc ttt tgc ttg tcc tga cgg cgt aaa aca cgt cta tca gtt acg tgc cag atc a
GTTTCACCTAAACTGTTCATCAGACAAGAGGAAGTTCAAGAACTTTACTCT

25504 33 3 0 14 ORF3a-QASLPFGWLIV38del
AGGATGCTACTCCTTCAGATTTTGTTCGCGCTACTGCAACGATACCGATA
caa gcc tca ctc cct ttc gga tgg ctt att gtt
GGCGTTGCACTTCTTGCTGTTTTTCAGAGCGCTTCCAAAATCATAACCCTC

27572 71 3 0 14 ORF7a-STQFAFACPDGVKHVYQLRARSVS60fs
TTCACCATTTCATCCTCTAGCTGATAACAAATTTGCACTGACTTGCTTTA
gca ctc aat ttg ctt ttg ctt gtc ctg acg gcg taa aac acg tct atc agt tac gtg cca gat cag ttt ca
CCTAAACTGTTCATCAGACAAGAGGAAGTTCAAGAACTTTACTCTCCAATT

29729 42 1 0 13
CTTTAATCAGTGTGTAACATTAGGGAGGACTTGAAAGAGCCACCACATTT
tca ccg agg cca cgc gga gta cga tcg agt gta cag tga aca
ATGCTAGGGAGAGCTGCCTATATGGAAGAGCCCTAATGTGTAAAATTAATT

27579 42 4 0 12 ORF7a-FAFACPDGVKHVYQ63del
TTTCATCCTCTAGCTGATAACAAATTTGCACTGACTTGCTTTAGCACTCA
att tgc ttt tgc ttg tcc tga cgg cgt aaa aca cgt cta tca
GTTACGTGCCAGATCAGTTTCACCTAAACTGTTCATCAGACAAGAGGAAGT

27686 77 4 0 11 ORF7a-SPIFLIVAAIVFITLCFTLKRKTE*98fs
AGTTTCACCTAAACTGTTCATCAGACAAGAGGAAGTTCAAGAACTTTACT
ctc caa ttt ttc tta ttg ttg cgg caa tag tgt tta taa cac ttt gct tca cac tca aaa gaa aga cag aat gat tg
AACTTTCATTAATTGACTTCTATTTGTGCTTTTTAGCCTTTCTGCTATTCC

27686 77 4 0 11 ORF7b-MIE1fs
AGTTTCACCTAAACTGTTCATCAGACAAGAGGAAGTTCAAGAACTTTACT
ctc caa ttt ttc tta ttg ttg cgg caa tag tgt tta taa cac ttt gct tca cac tca aaa gaa aga cag aat gat tg
AACTTTCATTAATTGACTTCTATTTGTGCTTTTTAGCCTTTCTGCTATTCC

27570 55 2 0 11 ORF7a-FSTQFAFACPDGVKHVYQL59fs
AATTCACCATTTCATCCTCTAGCTGATAACAAATTTGCACTGACTTGCTT
tag cac tca att tgc ttt tgc ttg tcc tga cgg cgt aaa aca cgt cta tca gtt a
CGTGCCAGATCAGTTTCACCTAAACTGTTCATCAGACAAGAGGAAGTTCAA

27705 36 1 0 11 ORF7a-AAIVFITLCFTL105del
ATCAGACAAGAGGAAGTTCAAGAACTTTACTCTCCAATTTTTCTTATTGT
tgc ggc aat agt gtt tat aac act ttg ctt cac act
CAAAAGAAAGACAGAATGATTGAACTTTCATTAATTGACTTCTATTTGTGC

29726 45 8 0 10
AATCTTTAATCAGTGTGTAACATTAGGGAGGACTTGAAAGAGCCACCACA

ttt tca ccg agg cca cgc gga gta cga tcg agt gta cag tga aca
ATGCTAGGGAGAGCTGCCTATATGGAAGAGCCCTAATGTGTAAAATTAATT

27562 79 3 0 10 ORF7a-TCFSTQFAFACPDGVKHVYQLRARSVS57fs
ACGAGGGCAATTCACCATTTCATCCTCTAGCTGATAACAAATTTGCACTG
act tgc ttt agc act caa ttt gct ttt gct tgt cct gac ggc gta aaa cac gtc tat cag tta cgt gcc aga tca gtt t
CACCTAAACTGTTCATCAGACAAGAGGAAGTTCAAGAACTTTACTCTCCAA

27577 59 3 0 10 ORF7a-QFAFACPDGVKHVYQLRARS62fs
CATTTCATCCTCTAGCTGATAACAAATTTGCACTGACTTGCTTTAGCACT
caa ttt gct ttt gct tgt cct gac ggc gta aaa cac gtc tat cag tta cgt gcc aga tc
AGTTTCACCTAAACTGTTCATCAGACAAGAGGAAGTTCAAGAACTTTACTC

27556 63 2 0 10 ORF7a-ALTCFSTQFAFACPDGVKHVY55del
GAACATACGAGGGCAATTCACCATTTCATCCTCTAGCTGATAACAAATTT
gca ctg act tgc ttt agc act caa ttt gct ttt gct tgt cct gac ggc gta aaa cac gtc tat
CAGTTACGTGCCAGATCAGTTTCACCTAAACTGTTCATCAGACAAGAGGAA

27688 63 6 0 9 ORF7a-PIFLIVAAIVFITLCFTLKRK99del
TTTCACCTAAACTGTTCATCAGACAAGAGGAAGTTCAAGAACTTTACTCT
cca att ttt ctt att gtt gcg gca ata gtg ttt ata aca ctt tgc ttc aca ctc aaa aga aag
ACAGAATGATTGAACTTTCATTAATTGACTTCTATTTGTGCTTTTTAGCCT

27684 72 6 0 9 ORF7a-YSPIFLIVAAIVFITLCFTLKRKTE97*
TCAGTTTCACCTAAACTGTTCATCAGACAAGAGGAAGTTCAAGAACTTTA
ctc tcc aat ttt tct tat tgt tgc ggc aat agt gtt tat aac act ttg ctt cac act caa aag aaa gac aga
ATGATTGAACTTTCATTAATTGACTTCTATTTGTGCTTTTTAGCCTTTCTG

27560 84 5 0 9 ORF7a-LTCFSTQFAFACPDGVKHVYQLRARSVS56del
ATACGAGGGCAATTCACCATTTCATCCTCTAGCTGATAACAAATTTGCAC
tga ctt gct tta gca ctc aat ttg ctt ttg ctt gtc ctg acg gcg taa aac acg tct atc agt tac gtg cca gat cag ttt cac
CTAAACTGTTCATCAGACAAGAGGAAGTTCAAGAACTTTACTCTCCAATTT

27683 52 3 0 9 ORF7a-YSPIFLIVAAIVFITLCF97fs
ATCAGTTTCACCTAAACTGTTCATCAGACAAGAGGAAGTTCAAGAACTTT
act ctc caa ttt ttc tta ttg ttg cgg caa tag tgt tta taa cac ttt gct t
CACACTCAAAAGAAAGACAGAATGATTGAACTTTCATTAATTGACTTCTAT

27686 61 2 0 9 ORF7a-SPIFLIVAAIVFITLCFTLKR98fs
AGTTTCACCTAAACTGTTCATCAGACAAGAGGAAGTTCAAGAACTTTACT
ctc caa ttt ttc tta ttg ttg cgg caa tag tgt tta taa cac ttt gct tca cac tca aaa g
AAAGACAGAATGATTGAACTTTCATTAATTGACTTCTATTTGTGCTTTTTA

27566 65 2 0 9 ORF7a-CFSTQFAFACPDGVKHVYQLRA58fs
GGGCAATTCACCATTTCATCCTCTAGCTGATAACAAATTTGCACTGACTT
gct tta gca ctc aat ttg ctt ttg ctt gtc ctg acg gcg taa aac acg tct atc agt tac gtg cc
AGATCAGTTTCACCTAAACTGTTCATCAGACAAGAGGAAGTTCAAGAACTT

27566 76 1 0 9 ORF7a-CFSTQFAFACPDGVKHVYQLRARSVS58fs
GGGCAATTCACCATTTCATCCTCTAGCTGATAACAAATTTGCACTGACTT
gct tta gca ctc aat ttg ctt ttg ctt gtc ctg acg gcg taa aac acg tct atc agt tac gtg cca gat cag ttt c
ACCTAAACTGTTCATCAGACAAGAGGAAGTTCAAGAACTTTACTCTCCAAT

27555 91 1 0 9 ORF7a-FALTCFSTQFAFACPDGVKHVYQLRARSVSP54fs

GGAACATACGAGGGCAATTCACCATTTCATCCTCTAGCTGATAACAAATT
tgc act gac ttg ctt tag cac tca att tgc ttt tgc ttg tcc tga cgg cgt aaa aca cgt cta tca gtt acg tgc cag atc agt ttc acc t
AAACTGTTCATCAGACAAGAGGAAGTTCAAGAACTTTACTCTCCAATTTTT

29724 47 2 0 8
GCAATCTTTAATCAGTGTGTAACATTAGGGAGGACTTGAAAGAGCCACCA
cat ttt cac cga ggc cac gcg gag tac gat cga gtg tac agt gaa ca
ATGCTAGGGAGAGCTGCCTATATGGAAGAGCCCTAATGTGTAAAATTAATT

27549 115 1 0 8 ORF7a-NKFALTCFSTQFAFACPDGVKHVYQLRARSVSPKLFIRQ52fs
TCTTCTGGAACATACGAGGGCAATTCACCATTTCATCCTCTAGCTGATAA
caa att tgc act gac ttg ctt tag cac tca att tgc ttt tgc ttg tcc tga cgg cgt aaa aca cgt cta tca gtt acg tgc cag atc agt ttc acc taa act gtt cat cag aca a
GAGGAAGTTCAAGAACTTTACTCTCCAATTTTTCTTATTGTTGCGGCAATA

28077 36 1 0 8 ORF8-VDEAGSKSPIQY62del
GGTATATTAGAGTAGGAGCTAGAAAATCAGCACCTTTAATTGAATTGTGC
gtg gat gag gct ggt tct aaa tca ccc att cag tac
ATCGATATCGGTAATTATACAGTTTCCTGTTTACCTTTTACAATTAATTGC

28090 69 1 0 8 ORF8-GSKSPIQYIDIGNYTVSCLPFTIN66D
AGGAGCTAGAAAATCAGCACCTTTAATTGAATTGTGCGTGGATGAGGCTG
gtt cta aat cac cca ttc agt aca tcg ata tcg gta att ata cag ttt cct gtt tac ctt tta caa tta
ATTGCCAGGAACCTAAATTGGGTAGTCTTGTAGTGCGTTGTTCGTTCTATG

27703 40 1 0 8 ORF7a-VAAIVFITLCFTLK104fs
TCATCAGACAAGAGGAAGTTCAAGAACTTTACTCTCCAATTTTTCTTATT
gtt gcg gca ata gtg ttt ata aca ctt tgc ttc aca ctc a
AAAGAAAGACAGAATGATTGAACTTTCATTAATTGACTTCTATTTGTGCTT

27573 60 4 0 7 ORF7a-STQFAFACPDGVKHVYQLRA60del
TCACCATTTCATCCTCTAGCTGATAACAAATTTGCACTGACTTGCTTTAG
cac tca att tgc ttt tgc ttg tcc tga cgg cgt aaa aca cgt cta tca gtt acg tgc cag
ATCAGTTTCACCTAAACTGTTCATCAGACAAGAGGAAGTTCAAGAACTTTA

27703 39 4 0 7 ORF7a-VAAIVFITLCFTL104del
TCATCAGACAAGAGGAAGTTCAAGAACTTTACTCTCCAATTTTTCTTATT
gtt gcg gca ata gtg ttt ata aca ctt tgc ttc aca ctc
AAAAGAAAGACAGAATGATTGAACTTTCATTAATTGACTTCTATTTGTGCT

29729 33 4 0 7
CTTTAATCAGTGTGTAACATTAGGGAGGACTTGAAAGAGCCACCACATTT
tca ccg agg cca cgc gga gta cga tcg agt gta
CAGTGAACAATGCTAGGGAGAGCTGCCTATATGGAAGAGCCCTAATGTGTA

27687 49 3 0 7 ORF7a-SPIFLIVAAIVFITLCF98fs
GTTTCACCTAAACTGTTCATCAGACAAGAGGAAGTTCAAGAACTTTACTC
tcc aat ttt tct tat tgt tgc ggc aat agt gtt tat aac act ttg ctt c
ACACTCAAAAGAAAGACAGAATGATTGAACTTTCATTAATTGACTTCTATT

27329 65 2 0 7 ORF6-SLTENKYSQLDEEQPMEID*43fs
TTGGAATCTTGATTACATCATAAACCTCATAATTAAAAATTTATCTAAGT
cac taa ctg aga ata aat att ctc aat tag atg aag agc aac caa tgg aga ttg att aaa cga ac
ATGAAAATTATTCTTTTCTTGGCACTGATAACACTCGCTACTTGTGAGCTT

28010 111 2 0 7 ORF8-HFYSKWYIRVGARKSAPLIELCVDEAGSKSPIQYIDI40del
CAGTCATGTACTCAACATCAACCATATGTAGTTGATGACCCGTGTCCTAT
tca ctt cta ttc taa atg gta tat tag agt agg agc tag aaa atc agc acc ttt aat tga att gtg cgt gga tga ggc tgg ttc taa atc acc cat tca gta cat cga tat
CGGTAATTATACAGTTTCCTGTTTACCTTTTACAATTAATTGCCAGGAACC

29728 42 2 0 7
TCTTTAATCAGTGTGTAACATTAGGGAGGACTTGAAAGAGCCACCACATT
ttc acc gag gcc acg cgg agt acg atc gag tgt aca gtg aac
AATGCTAGGGAGAGCTGCCTATATGGAAGAGCCCTAATGTGTAAAATTAAT

29732 36 3 0 6
TAATCAGTGTGTAACATTAGGGAGGACTTGAAAGAGCCACCACATTTTCA
ccg agg cca cgc gga gta cga tcg agt gta cag tga
ACAATGCTAGGGAGAGCTGCCTATATGGAAGAGCCCTAATGTGTAAAATTA

27684 80 3 0 6 ORF7a-YSPIFLIVAAIVFITLCFTLKRKTE*97fs
TCAGTTTCACCTAAACTGTTCATCAGACAAGAGGAAGTTCAAGAACTTTA
ctc tcc aat ttt tct tat tgt tgc ggc aat agt gtt tat aac act ttg ctt cac act caa aag aaa gac aga atg att ga
ACTTTCATTAATTGACTTCTATTTGTGCTTTTTAGCCTTTCTGCTATTCCT

27684 80 3 0 6 ORF7b-MIE1fs
TCAGTTTCACCTAAACTGTTCATCAGACAAGAGGAAGTTCAAGAACTTTA
ctc tcc aat ttt tct tat tgt tgc ggc aat agt gtt tat aac act ttg ctt cac act caa aag aaa gac aga atg att ga
ACTTTCATTAATTGACTTCTATTTGTGCTTTTTAGCCTTTCTGCTATTCCT

28068 52 3 0 6 ORF8-ELCVDEAGSKSPIQYIDI59fs
ATTCTAAATGGTATATTAGAGTAGGAGCTAGAAAATCAGCACCTTTAATT
gaa ttg tgc gtg gat gag gct ggt tct aaa tca ccc att cag tac atc gat a
TCGGTAATTATACAGTTTCCTGTTTACCTTTTACAATTAATTGCCAGGAAC

28008 74 2 0 6 ORF8-IHFYSKWYIRVGARKSAPLIELCVD39fs
TACAGTCATGTACTCAACATCAACCATATGTAGTTGATGACCCGTGTCCT
att cac ttc tat tct aaa tgg tat att aga gta gga gct aga aaa tca gca cct tta att gaa ttg tgc gtg ga
TGAGGCTGGTTCTAAATCACCCATTCAGTACATCGATATCGGTAATTATAC

27573 49 2 0 6 ORF7a-STQFAFACPDGVKHVYQ60fs
TCACCATTTCATCCTCTAGCTGATAACAAATTTGCACTGACTTGCTTTAG
cac tca att tgc ttt tgc ttg tcc tga cgg cgt aaa aca cgt cta tca g
TTACGTGCCAGATCAGTTTCACCTAAACTGTTCATCAGACAAGAGGAAGTT

27556 84 2 0 6 ORF7a-ALTCFSTQFAFACPDGVKHVYQLRARSV55del
GAACATACGAGGGCAATTCACCATTTCATCCTCTAGCTGATAACAAATTT
gca ctg act tgc ttt agc act caa ttt gct ttt gct tgt cct gac ggc gta aaa cac gtc tat cag tta cgt gcc aga tca gtt
TCACCTAAACTGTTCATCAGACAAGAGGAAGTTCAAGAACTTTACTCTCCA

27570 71 2 0 6 ORF7a-FSTQFAFACPDGVKHVYQLRARSV59fs
AATTCACCATTTCATCCTCTAGCTGATAACAAATTTGCACTGACTTGCTT
tag cac tca att tgc ttt tgc ttg tcc tga cgg cgt aaa aca cgt cta tca gtt acg tgc cag atc agt tt
CACCTAAACTGTTCATCAGACAAGAGGAAGTTCAAGAACTTTACTCTCCAA

27583 58 1 0 6 ORF7a-AFACPDGVKHVYQLRARSVS64fs
ATCCTCTAGCTGATAACAAATTTGCACTGACTTGCTTTAGCACTCAATTT

gct ttt gct tgt cct gac ggc gta aaa cac gtc tat cag tta cgt gcc aga tca gtt t
CACCTAAACTGTTCATCAGACAAGAGGAAGTTCAAGAACTTTACTCTCCAA

27580 44 4 0 5 ORF7a-FAFACPDGVKHVYQL63fs
TTCATCCTCTAGCTGATAACAAATTTGCACTGACTTGCTTTAGCACTCAA
ttt gct ttt gct tgt cct gac ggc gta aaa cac gtc tat cag tt
ACGTGCCAGATCAGTTTCACCTAAACTGTTCATCAGACAAGAGGAAGTTCA

27683 58 3 0 5 ORF7a-YSPIFLIVAAIVFITLCFTL97fs
ATCAGTTTCACCTAAACTGTTCATCAGACAAGAGGAAGTTCAAGAACTTT
act ctc caa ttt ttc tta ttg ttg cgg caa tag tgt tta taa cac ttt gct tca cac t
CAAAAGAAAGACAGAATGATTGAACTTTCATTAATTGACTTCTATTTGTGC

29732 39 2 0 5
TAATCAGTGTGTAACATTAGGGAGGACTTGAAAGAGCCACCACATTTTCA
ccg agg cca cgc gga gta cga tcg agt gta cag tga aca
ATGCTAGGGAGAGCTGCCTATATGGAAGAGCCCTAATGTGTAAAATTAATT

27521 45 1 0 5 ORF7a-NSPFHPLADNKFALTC43S
TACAACAGTACTTTTAAAAGAACCTTGCTCTTCTGGAACATACGAGGGCA
att cac cat ttc atc ctc tag ctg ata aca aat ttg cac tga ctt
GCTTTAGCACTCAATTTGCTTTTGCTTGTCCTGACGGCGTAAAACACGTCT

27238 88 1 0 5 ORF6-EILLIIMRTFKVSIWNLDYIINLIIKNLSK13fs
GTAAGTGACAACAGATGTTTCATCTCGTTGACTTTCAGGTTACTATAGCA
gag ata tta cta att att atg agg act ttt aaa gtt tcc att tgg aat ctt gat tac atc ata aac ctc ata att aaa aat tta tct a
AGTCACTAACTGAGAATAAATATTCTCAATTAGATGAAGAGCAACCAATGG

27556 64 1 0 5 ORF7a-ALTCFSTQFAFACPDGVKHVYQ55fs
GAACATACGAGGGCAATTCACCATTTCATCCTCTAGCTGATAACAAATTT
gca ctg act tgc ttt agc act caa ttt gct ttt gct tgt cct gac ggc gta aaa cac gtc tat c
AGTTACGTGCCAGATCAGTTTCACCTAAACTGTTCATCAGACAAGAGGAAG

27566 64 1 0 5 ORF7a-CFSTQFAFACPDGVKHVYQLRA58fs
GGGCAATTCACCATTTCATCCTCTAGCTGATAACAAATTTGCACTGACTT
gct tta gca ctc aat ttg ctt ttg ctt gtc ctg acg gcg taa aac acg tct atc agt tac gtg c
CAGATCAGTTTCACCTAAACTGTTCATCAGACAAGAGGAAGTTCAAGAACT

27566 58 3 0 4 ORF7a-CFSTQFAFACPDGVKHVYQL58fs
GGGCAATTCACCATTTCATCCTCTAGCTGATAACAAATTTGCACTGACTT
gct tta gca ctc aat ttg ctt ttg ctt gtc ctg acg gcg taa aac acg tct atc agt t
ACGTGCCAGATCAGTTTCACCTAAACTGTTCATCAGACAAGAGGAAGTTCA

28027 131 3 0 4 ORF8-WYIRVGARKSAPLIELCVDEAGSKSPIQYIDIGNYTVSCLPFTI45
TCAACCATATGTAGTTGATGACCCGTGTCCTATTCACTTCTATTCTAAAT
ggt ata tta gag tag gag cta gaa aat cag cac ctt taa ttg aat tgt gcg tgg atg agg ctg gtt cta aat cac cca ttc agt aca tcg ata tcg
gta att ata cag ttt cct gtt tac ctt tta caa tt
AATTGCCAGGAACCTAAATTGGGTAGTCTTGTAGTGCGTTGTTCGTTCTAT

29729 38 3 0 4
CTTTAATCAGTGTGTAACATTAGGGAGGACTTGAAAGAGCCACCACATTT
tca ccg agg cca cgc gga gta cga tcg agt gta cag tg
AACAATGCTAGGGAGAGCTGCCTATATGGAAGAGCCCTAATGTGTAAAATT

29726 51 3 0 4
AATCTTTAATCAGTGTGTAACATTAGGGAGGACTTGAAAGAGCCACCACA
ttt tca ccg agg cca cgc gga gta cga tcg agt gta cag tga aca atg cta
GGGAGAGCTGCCTATATGGAAGAGCCCTAATGTGTAAAATTAATTTTAGTA

27577 34 2 0 4 ORF7a-QFAFACPDGVKH62fs
CATTTCATCCTCTAGCTGATAACAAATTTGCACTGACTTGCTTTAGCACT
caa ttt gct ttt gct tgt cct gac ggc gta aaa c
ACGTCTATCAGTTACGTGCCAGATCAGTTTCACCTAAACTGTTCATCAGAC

27555 70 2 0 4 ORF7a-FALTCFSTQFAFACPDGVKHVYQL54fs
GGAACATACGAGGGCAATTCACCATTTCATCCTCTAGCTGATAACAAATT
tgc act gac ttg ctt tag cac tca att tgc ttt tgc ttg tcc tga cgg cgt aaa aca cgt cta tca gtt a
CGTGCCAGATCAGTTTCACCTAAACTGTTCATCAGACAAGAGGAAGTTCAA

27694 49 2 0 4 ORF7a-FLIVAAIVFITLCFTLK101fs
CTAAACTGTTCATCAGACAAGAGGAAGTTCAAGAACTTTACTCTCCAATT
ttt ctt att gtt gcg gca ata gtg ttt ata aca ctt tgc ttc aca ctc a
AAAGAAAGACAGAATGATTGAACTTTCATTAATTGACTTCTATTTGTGCTT

27599 42 1 0 4 ORF7a-DGVKHVYQLRARSVS69A
CAAATTTGCACTGACTTGCTTTAGCACTCAATTTGCTTTTGCTTGTCCTG
acg gcg taa aac acg tct atc agt tac gtg cca gat cag ttt
CACCTAAACTGTTCATCAGACAAGAGGAAGTTCAAGAACTTTACTCTCCAA

28043 69 1 0 4 ORF8-ARKSAPLIELCVDEAGSKSPIQY51del
GATGACCCGTGTCCTATTCACTTCTATTCTAAATGGTATATTAGAGTAGG
agc tag aaa atc agc acc ttt aat tga att gtg cgt gga tga ggc tgg ttc taa atc acc cat tca gta
CATCGATATCGGTAATTATACAGTTTCCTGTTTACCTTTTACAATTAATTG

27579 77 1 0 4 ORF7a-QFAFACPDGVKHVYQLRARSVSPKLF62fs
TTTCATCCTCTAGCTGATAACAAATTTGCACTGACTTGCTTTAGCACTCA
att tgc ttt tgc ttg tcc tga cgg cgt aaa aca cgt cta tca gtt acg tgc cag atc agt ttc acc taa act gtt ca
TCAGACAAGAGGAAGTTCAAGAACTTTACTCTCCAATTTTTCTTATTGTTG

27559 88 1 0 4 ORF7a-LTCFSTQFAFACPDGVKHVYQLRARSVSPK56fs
CATACGAGGGCAATTCACCATTTCATCCTCTAGCTGATAACAAATTTGCA
ctg act tgc ttt agc act caa ttt gct ttt gct tgt cct gac ggc gta aaa cac gtc tat cag tta cgt gcc aga tca gtt tca cct a
AACTGTTCATCAGACAAGAGGAAGTTCAAGAACTTTACTCTCCAATTTTTC

28003 162 1 0 4 ORF8-CPIHFYSKWYIRVGARKSAPLIELCVDEAGSKSPIQYIDIGNYTVS
TAGTTTACAGTCATGTACTCAACATCAACCATATGTAGTTGATGACCCGT
gtc cta ttc act tct att cta aat ggt ata tta gag tag gag cta gaa aat cag cac ctt taa ttg aat tgt gcg tgg atg agg ctg gtt cta aat
cac cca ttc agt aca tcg ata tcg gta att ata cag ttt cct gtt tac ctt tta caa tta att gcc
AGGAACCTAAATTGGGTAGTCTTGTAGTGCGTTGTTCGTTCTATGAAGACT

27656 87 1 0 4 ORF7a-IRQEEVQELYSPIFLIVAAIVFITLCFTL88del
AAAACACGTCTATCAGTTACGTGCCAGATCAGTTTCACCTAAACTGTTCA
tca gac aag agg aag ttc aag aac ttt act ctc caa ttt ttc tta ttg ttg cgg caa tag tgt tta taa cac ttt gct tca cac tca
AAAGAAAGACAGAATGATTGAACTTTCATTAATTGACTTCTATTTGTGCTT

27553 56 1 0 4 ORF7a-FALTCFSTQFAFACPDGVK54fs
CTGGAACATACGAGGGCAATTCACCATTTCATCCTCTAGCTGATAACAAA
ttt gca ctg act tgc ttt agc act caa ttt gct ttt gct tgt cct gac ggc gta aa

ACACGTCTATCAGTTACGTGCCAGATCAGTTTCACCTAAACTGTTCATCAG

27586 33 1 0 4 ORF7a-FACPDGVKHVY65del
CTCTAGCTGATAACAAATTTGCACTGACTTGCTTTAGCACTCAATTTGCT
ttt gct tgt cct gac ggc gta aaa cac gtc tat
CAGTTACGTGCCAGATCAGTTTCACCTAAACTGTTCATCAGACAAGAGGAA

27693 33 1 0 4 ORF7a-FLIVAAIVFIT101del
CCTAAACTGTTCATCAGACAAGAGGAAGTTCAAGAACTTTACTCTCCAAT
ttt tct tat tgt tgc ggc aat agt gtt tat aac
ACTTTGCTTCACACTCAAAAGAAAGACAGAATGATTGAACTTTCATTAATT

5652 36 3 0 3 ORF1a-YLVQQESPFVMM1796del
TAAGAAAGGTGTTCAGATACCTTGTACGTGTGGTAAACAAGCTACAAAAT
atc tag tac aac agg agt cac ctt ttg tta tga tgt
CAGCACCACCTGCTCAGTATGAACTTAAGCATGGTACATTTACTTGTGCTA

27406 56 3 0 3 ORF7a-LFLALITLATCELYHYQEC5fs
TAGATGAAGAGCAACCAATGGAGATTGATTAAACGAACATGAAAATTATT
ctt ttc ttg gca ctg ata aca ctc gct act tgt gag ctt tat cac tac caa gag tg
TGTTAGAGGTACAACAGTACTTTTAAAAGAACCTTGCTCTTCTGGAACATA

27583 36 3 0 3 ORF7a-AFACPDGVKHVY64del
ATCCTCTAGCTGATAACAAATTTGCACTGACTTGCTTTAGCACTCAATTT
gct ttt gct tgt cct gac ggc gta aaa cac gtc tat
CAGTTACGTGCCAGATCAGTTTCACCTAAACTGTTCATCAGACAAGAGGAA

426 33 3 0 3 ORF1a-VEVEKGVLPQL54del
GGTCTTATCAGAGGCACGTCAACATCTTAAAGATGGCACTTGTGGCTTAG
tag aag ttg aaa aag gcg ttt tgc ctc aac ttg
AACAGCCCTATGTGTTCATCAAACGTTCGGATGCTCGAACTGCACCTCATG

27565 66 3 0 3 ORF7a-CFSTQFAFACPDGVKHVYQLRA58del
AGGGCAATTCACCATTTCATCCTCTAGCTGATAACAAATTTGCACTGACT
tgc ttt agc act caa ttt gct ttt gct tgt cct gac ggc gta aaa cac gtc tat cag tta cgt gcc
AGATCAGTTTCACCTAAACTGTTCATCAGACAAGAGGAAGTTCAAGAACTT

27553 96 3 0 3 ORF7a-FALTCFSTQFAFACPDGVKHVYQLRARSVSPK54del
CTGGAACATACGAGGGCAATTCACCATTTCATCCTCTAGCTGATAACAAA
ttt gca ctg act tgc ttt agc act caa ttt gct ttt gct tgt cct gac ggc gta aaa cac gtc tat cag tta cgt gcc aga tca gtt tca cct aaa
CTGTTCATCAGACAAGAGGAAGTTCAAGAACTTTACTCTCCAATTTTTCTT

29733 43 2 0 3
AATCAGTGTGTAACATTAGGGAGGACTTGAAAGAGCCACCACATTTTCAC
cga ggc cac gcg gag tac gat cga gtg tac agt gaa caa tgc t
AGGGAGAGCTGCCTATATGGAAGAGCCCTAATGTGTAAAATTAATTTTAGT

27575 43 2 0 3 ORF7a-TQFAFACPDGVKHVY61fs
ACCATTTCATCCTCTAGCTGATAACAAATTTGCACTGACTTGCTTTAGCA
ctc aat ttg ctt ttg ctt gtc ctg acg gcg taa aac acg tct a
TCAGTTACGTGCCAGATCAGTTTCACCTAAACTGTTCATCAGACAAGAGGA

27684 73 2 0 3 ORF7a-YSPIFLIVAAIVFITLCFTLKRKTE97fs
TCAGTTTCACCTAAACTGTTCATCAGACAAGAGGAAGTTCAAGAACTTTA

ctc tcc aat ttt tct tat tgt tgc ggc aat agt gtt tat aac act ttg ctt cac act caa aag aaa gac aga a
TGATTGAACTTTCATTAATTGACTTCTATTTGTGCTTTTTAGCCTTTCTGC

27684 73 2 0 3 ORF7b-M1fs
TCAGTTTCACCTAAACTGTTCATCAGACAAGAGGAAGTTCAAGAACTTTA
ctc tcc aat ttt tct tat tgt tgc ggc aat agt gtt tat aac act ttg ctt cac act caa aag aaa gac aga a
TGATTGAACTTTCATTAATTGACTTCTATTTGTGCTTTTTAGCCTTTCTGC

27566 51 2 0 3 ORF7a-CFSTQFAFACPDGVKHV58del
GGGCAATTCACCATTTCATCCTCTAGCTGATAACAAATTTGCACTGACTT
gct tta gca ctc aat ttg ctt ttg ctt gtc ctg acg gcg taa aac acg tct
ATCAGTTACGTGCCAGATCAGTTTCACCTAAACTGTTCATCAGACAAGAGG

188 36 2 0 3
AACTAATTACTGTCGTTGACAGGACACGAGTAACTCGTCTATCTTCTGCA
ggc tgc tta cgg ttt cgt ccg tgt tgc agc cga tca
TCAGCACATCTAGGTTTCGTCCGGGTGTGACCGAAAGGTAAGATGGAGAGC

27576 50 2 0 3 ORF7a-TQFAFACPDGVKHVYQL61fs
CCATTTCATCCTCTAGCTGATAACAAATTTGCACTGACTTGCTTTAGCAC
tca att tgc ttt tgc ttg tcc tga cgg cgt aaa aca cgt cta tca gtt ac
GTGCCAGATCAGTTTCACCTAAACTGTTCATCAGACAAGAGGAAGTTCAAG

27555 86 2 0 3 ORF7a-FALTCFSTQFAFACPDGVKHVYQLRARSV54fs
GGAACATACGAGGGCAATTCACCATTTCATCCTCTAGCTGATAACAAATT
tgc act gac ttg ctt tag cac tca att tgc ttt tgc ttg tcc tga cgg cgt aaa aca cgt cta tca gtt acg tgc cag atc agt tt
CACCTAAACTGTTCATCAGACAAGAGGAAGTTCAAGAACTTTACTCTCCAA

27555 87 2 0 3 ORF7a-FALTCFSTQFAFACPDGVKHVYQLRARSVS54L
GGAACATACGAGGGCAATTCACCATTTCATCCTCTAGCTGATAACAAATT
tgc act gac ttg ctt tag cac tca att tgc ttt tgc ttg tcc tga cgg cgt aaa aca cgt cta tca gtt acg tgc cag atc agt ttc
ACCTAAACTGTTCATCAGACAAGAGGAAGTTCAAGAACTTTACTCTCCAAT

27716 39 2 0 3 ORF7a-VFITLCFTLKRKT108del
GGAAGTTCAAGAACTTTACTCTCCAATTTTTCTTATTGTTGCGGCAATAG
tgt tta taa cac ttt gct tca cac tca aaa gaa aga cag
AATGATTGAACTTTCATTAATTGACTTCTATTTGTGCTTTTTAGCCTTTCT

27397 122 1 0 3 ORF7a-KIILFLALITLATCELYHYQECVRGTTVLLKEPCSSGTYEG2fs
ATTCTCAATTAGATGAAGAGCAACCAATGGAGATTGATTAAACGAACATG
aaa att att ctt ttc ttg gca ctg ata aca ctc gct act tgt gag ctt tat cac tac caa gag tgt gtt aga ggt aca aca gta ctt tta aaa gaa cct tgc tct tct gga aca tac gag gg
CAATTCACCATTTCATCCTCTAGCTGATAACAAATTTGCACTGACTTGCTT

27551 70 1 0 3 ORF7a-KFALTCFSTQFAFACPDGVKHVYQ53fs
TTCTGGAACATACGAGGGCAATTCACCATTTCATCCTCTAGCTGATAACA
aat ttg cac tga ctt gct tta gca ctc aat ttg ctt ttg ctt gtc ctg acg gcg taa aac acg tct atc a
GTTACGTGCCAGATCAGTTTCACCTAAACTGTTCATCAGACAAGAGGAAGT

27548 108 1 0 3 ORF7a-NKFALTCFSTQFAFACPDGVKHVYQLRARSVSPKLF52del
CTCTTCTGGAACATACGAGGGCAATTCACCATTTCATCCTCTAGCTGATA
aca aat ttg cac tga ctt gct tta gca ctc aat ttg ctt ttg ctt gtc ctg acg gcg taa aac acg tct atc agt tac gtg cca gat cag ttt cac cta aac tgt tca
TCAGACAAGAGGAAGTTCAAGAACTTTACTCTCCAATTTTTCTTATTGTTG

27558 92 1 0 3 ORF7a-ALTCFSTQFAFACPDGVKHVYQLRARSVSPK55fs
ACATACGAGGGCAATTCACCATTTCATCCTCTAGCTGATAACAAATTTGC
act gac ttg ctt tag cac tca att tgc ttt tgc ttg tcc tga cgg cgt aaa aca cgt cta tca gtt acg tgc cag atc agt ttc acc taa ac
TGTTCATCAGACAAGAGGAAGTTCAAGAACTTTACTCTCCAATTTTTCTTA

27270 33 1 0 3 ORF6-KVSIWNLDYII23del
TTTCAGGTTACTATAGCAGAGATATTACTAATTATTATGAGGACTTTTAA
agt ttc cat ttg gaa tct tga tta cat cat aaa
CCTCATAATTAAAAATTTATCTAAGTCACTAACTGAGAATAAATATTCTCA

28006 49 1 0 3 ORF8-PIHFYSKWYIRVGARKS38fs
TTTACAGTCATGTACTCAACATCAACCATATGTAGTTGATGACCCGTGTC
cta ttc act tct att cta aat ggt ata tta gag tag gag cta gaa aat c
AGCACCTTTAATTGAATTGTGCGTGGATGAGGCTGGTTCTAAATCACCCAT

27542 75 1 0 3 ORF7a-ADNKFALTCFSTQFAFACPDGVKHVY50D
ACCTTGCTCTTCTGGAACATACGAGGGCAATTCACCATTTCATCCTCTAG
ctg ata aca aat ttg cac tga ctt gct tta gca ctc aat ttg ctt ttg ctt gtc ctg acg gcg taa aac acg tct
ATCAGTTACGTGCCAGATCAGTTTCACCTAAACTGTTCATCAGACAAGAGG

27572 48 1 0 3 ORF7a-STQFAFACPDGVKHVYQ60K
TTCACCATTTCATCCTCTAGCTGATAACAAATTTGCACTGACTTGCTTTA
gca ctc aat ttg ctt ttg ctt gtc ctg acg gcg taa aac acg tct atc
AGTTACGTGCCAGATCAGTTTCACCTAAACTGTTCATCAGACAAGAGGAAG

27556 43 1 0 3 ORF7a-ALTCFSTQFAFACPD55fs
GAACATACGAGGGCAATTCACCATTTCATCCTCTAGCTGATAACAAATTT
gca ctg act tgc ttt agc act caa ttt gct ttt gct tgt cct g
ACGGCGTAAAACACGTCTATCAGTTACGTGCCAGATCAGTTTCACCTAAAC

27566 59 1 0 3 ORF7a-CFSTQFAFACPDGVKHVYQL58fs
GGGCAATTCACCATTTCATCCTCTAGCTGATAACAAATTTGCACTGACTT
gct tta gca ctc aat ttg ctt ttg ctt gtc ctg acg gcg taa aac acg tct atc agt ta
CGTGCCAGATCAGTTTCACCTAAACTGTTCATCAGACAAGAGGAAGTTCAA

27913 81 1 0 3 ORF8-LGIITTVAAFHQECSLQSCTQHQPYVVD7Y
TCATAATGAAACTTGTCACGCCTAAACGAACATGAAATTTCTTGTTTTCT
tag gaa tca tca caa ctg tag ctg cat ttc acc aag aat gta gtt tac agt cat gta ctc aac atc aac cat atg tag ttg
ATGACCCGTGTCCTATTCACTTCTATTCTAAATGGTATATTAGAGTAGGAG

28031 42 1 0 3 ORF8-YIRVGARKSAPLIEL46*
CCATATGTAGTTGATGACCCGTGTCCTATTCACTTCTATTCTAAATGGTA
tat tag agt agg agc tag aaa atc agc acc ttt aat tga att
GTGCGTGGATGAGGCTGGTTCTAAATCACCCATTCAGTACATCGATATCGG

27696 55 1 0 3 ORF7a-FLIVAAIVFITLCFTLKRK101fs
AAACTGTTCATCAGACAAGAGGAAGTTCAAGAACTTTACTCTCCAATTTT
tct tat tgt tgc ggc aat agt gtt tat aac act ttg ctt cac act caa aag aaa g
ACAGAATGATTGAACTTTCATTAATTGACTTCTATTTGTGCTTTTTAGCCT

27698 55 1 0 3 ORF7a-LIVAAIVFITLCFTLKRKT102fs
ACTGTTCATCAGACAAGAGGAAGTTCAAGAACTTTACTCTCCAATTTTTC
tta ttg ttg cgg caa tag tgt tta taa cac ttt gct tca cac tca aaa gaa aga c

AGAATGATTGAACTTTCATTAATTGACTTCTATTTGTGCTTTTTAGCCTTT

29728 35 1 0 3
TCTTTAATCAGTGTGTAACATTAGGGAGGACTTGAAAGAGCCACCACATT
ttc acc gag gcc acg cgg agt acg atc gag tgt ac
AGTGAACAATGCTAGGGAGAGCTGCCTATATGGAAGAGCCCTAATGTGTAA

27909 60 1 0 3 ORF8-FLGIITTVAAFHQECSLQSC6del
AAGATCATAATGAAACTTGTCACGCCTAAACGAACATGAAATTTCTTGTT
ttc tta gga atc atc aca act gta gct gca ttt cac caa gaa tgt agt tta cag tca tgt
ACTCAACATCAACCATATGTAGTTGATGACCCGTGTCCTATTCACTTCTAT

27568 52 1 0 3 ORF7a-FSTQFAFACPDGVKHVYQ59fs
GCAATTCACCATTTCATCCTCTAGCTGATAACAAATTTGCACTGACTTGC
ttt agc act caa ttt gct ttt gct tgt cct gac ggc gta aaa cac gtc tat c
AGTTACGTGCCAGATCAGTTTCACCTAAACTGTTCATCAGACAAGAGGAAG

11288 9 75 434 365348 ORF1a-SGF3675del
GTTGGGTGATGCGTATTATGACATGGTTGGATATGGTTGATACTAGTTTG
tct ggt ttt
AAGCTAAAAGACTGTGTTATGTATGCATCAGCTGTAGTGTTACTAATCCTT

29738 23 102 280 5476
GTGTGTAACATTAGGGAGGACTTGAAAGAGCCACCACATTTTCACCGAGG
cca cgc gga gta cga tcg agt gt
ACAGTGAACAATGCTAGGGAGAGCTGCCTATATGGAAGAGCCCTAATGTGT

22287 9 78 54 2097 S-LAL242del
ATTGGTAGATTTGCCAATAGGTATTAACATCACTAGGTTTCAAACTTTAC
ttg ctt tac
ATAGAAGTTATTTGACTCCTGGTGATTCTTCTTCAGGTTGGACAGCTGGTG

686 9 727 16 2001 ORF1a-KSF141del
GTAAGAACGGTAATAAAGGAGCTGGTGGCCATAGTTACGGCGCCGATCTA
aag tca ttt
GACTTAGGCGACGAGCTTGGCACTGATCCTTATGAAGATTTTCAAGAAAAC

25448 30 2 11 158 ORF3a-EIKDATPSDF19del
TTTGTTTATGAGAATCTTCACAATTGGAACTGTAACTTTGAAGCAAGGTG
aaa tca agg atg cta ctc ctt cag att ttg
TTCGCGCTACTGCAACGATACCGATACAAGCCTCACTCCCTTTCGGATGGC

27571 12 30 10 556 ORF7a-STQF60del
ATTCACCATTTCATCCTCTAGCTGATAACAAATTTGCACTGACTTGCTTT
agc act caa ttt
GCTTTTGCTTGTCCTGACGGCGTAAAACACGTCTATCAGTTACGTGCCAGA

29738 11 103 9 504
GTGTGTAACATTAGGGAGGACTTGAAAGAGCCACCACATTTTCACCGAGG
cca cgc gga gt
ACGATCGAGTGTACAGTGAACAATGCTAGGGAGAGCTGCCTATATGGAAGA

22299 21 1 6 465 S-RSYLTPGD246N
GCCAATAGGTATTAACATCACTAGGTTTCAAACTTTACTTGCTTTACATA

gaa gtt att tga ctc ctg gtg
ATTCTTCTTCAGGTTGGACAGCTGGTGCTGCAGCTTATTATGTGGGTTATC

21984 9 22 6 306 S-LGV141del
TAATGTTGTTATTAAAGTCTGTGAATTTCAATTTTGTAATGATCCATTTT
tgg gtg ttt
ATTACCACAAAAACAACAAAAGTTGGATGGAAAGTGAGTTCAGAGTTTATT

27887 15 1 6 296 ORF7b-*44fs
TTTTGGTTCTCACTTGAACTGCAAGATCATAATGAAACTTGTCACGCCTA
aac gaa cat gaa att
TCTTGTTTTCTTAGGAATCATCACAACTGTAGCTGCATTTCACCAAGAATG

27887 15 1 6 296 ORF8-MKF1fs
TTTTGGTTCTCACTTGAACTGCAAGATCATAATGAAACTTGTCACGCCTA
aac gaa cat gaa att
TCTTGTTTTCTTAGGAATCATCACAACTGTAGCTGCATTTCACCAAGAATG

29729 18 2 6 141
CTTTAATCAGTGTGTAACATTAGGGAGGACTTGAAAGAGCCACCACATTT
tca ccg agg cca cgc gga
GTACGATCGAGTGTACAGTGAACAATGCTAGGGAGAGCTGCCTATATGGAA

29750 7 9 5 74
AGGGAGGACTTGAAAGAGCCACCACATTTTCACCGAGGCCACGCGGAGTA
cga tcg a
GTGTACAGTGAACAATGCTAGGGAGAGCTGCCTATATGGAAGAGCCCTAAT

27677 30 1 5 62 ORF7a-ELYSPIFLIV95del
TGCCAGATCAGTTTCACCTAAACTGTTCATCAGACAAGAGGAAGTTCAAG
aac ttt act ctc caa ttt ttc tta ttg ttg
CGGCAATAGTGTTTATAACACTTTGCTTCACACTCAAAAGAAAGACAGAAT

510 15 388 4 745 ORF1a-GHVMV82del
ACAGCCCTATGTGTTCATCAAACGTTCGGATGCTCGAACTGCACCTCATG
gtc atg tta tgg ttg
AGCTGGTAGCAGAACTCGAAGGCATTCAGTACGGTCGTAGTGGTGAGACAC

510 9 300 4 483 ORF1a-GHVM82V
ACAGCCCTATGTGTTCATCAAACGTTCGGATGCTCGAACTGCACCTCATG
gtc atg tta
TGGTTGAGCTGGTAGCAGAACTCGAAGGCATTCAGTACGGTCGTAGTGGTG

29760 7 133 4 453
TGAAAGAGCCACCACATTTTCACCGAGGCCACGCGGAGTACGATCGAGTG
tac agt g
AACAATGCTAGGGAGAGCTGCCTATATGGAAGAGCCCTAATGTGTAAAATT

29730 31 18 4 112
TTTAATCAGTGTGTAACATTAGGGAGGACTTGAAAGAGCCACCACATTTT
cac cga ggc cac gcg gag tac gat cga gtg t
ACAGTGAACAATGCTAGGGAGAGCTGCCTATATGGAAGAGCCCTAATGTGT

27674 24 10 4 94 ORF7a-QELYSPIF94del

ACGTGCCAGATCAGTTTCACCTAAACTGTTCATCAGACAAGAGGAAGTTC
aag aac ttt act ctc caa ttt ttc
TTATTGTTGCGGCAATAGTGTTTATAACACTTTGCTTCACACTCAAAAGAA

29751 12 43 3 320
GGGAGGACTTGAAAGAGCCACCACATTTTCACCGAGGCCACGCGGAGTAC
gat cga gtg tac
AGTGAACAATGCTAGGGAGAGCTGCCTATATGGAAGAGCCCTAATGTGTAA

29732 32 4 3 62
TAATCAGTGTGTAACATTAGGGAGGACTTGAAAGAGCCACCACATTTTCA
ccg agg cca cgc gga gta cga tcg agt gta ca
GTGAACAATGCTAGGGAGAGCTGCCTATATGGAAGAGCCCTAATGTGTAAA

29759 8 4 3 30
TTGAAAGAGCCACCACATTTTCACCGAGGCCACGCGGAGTACGATCGAGT
gta cag tg
AACAATGCTAGGGAGAGCTGCCTATATGGAAGAGCCCTAATGTGTAAAATT

29750 10 33 2 112
AGGGAGGACTTGAAAGAGCCACCACATTTTCACCGAGGCCACGCGGAGTA
cga tcg agt g
TACAGTGAACAATGCTAGGGAGAGCTGCCTATATGGAAGAGCCCTAATGTG

28242 12 3 2 66 ORF8-VLDF117del
TGCGTTGTTCGTTCTATGAAGACTTTTTAGAGTATCATGACGTTCGTGTT
gtt tta gat ttc
ATCTAAACGAACAAACTAAAATGTCTGATAATGGACCCCAAAATCAGCGAA

22029 9 2 2 58 S-EFR156del
ATTTTTGGGTGTTTATTACCACAAAAACAACAAAAGTTGGATGGAAAGTG
agt tca gag
TTTATTCTAGTGCGAATAATTGCACTTTTGAATATGTCTCTCAGCCTTTTC

29730 19 20 2 57
TTTAATCAGTGTGTAACATTAGGGAGGACTTGAAAGAGCCACCACATTTT
cac cga ggc cac gcg gag t
ACGATCGAGTGTACAGTGAACAATGCTAGGGAGAGCTGCCTATATGGAAGA

27570 13 4 2 54 ORF7a-FSTQF59fs
AATTCACCATTTCATCCTCTAGCTGATAACAAATTTGCACTGACTTGCTT
tag cac tca att t
GCTTTTGCTTGTCCTGACGGCGTAAAACACGTCTATCAGTTACGTGCCAGA

29749 10 11 2 36
TAGGGAGGACTTGAAAGAGCCACCACATTTTCACCGAGGCCACGCGGAGT
acg atc gag t
GTACAGTGAACAATGCTAGGGAGAGCTGCCTATATGGAAGAGCCCTAATGT

27594 30 4 2 34 ORF7a-CPDGVKHVYQL67*
GATAACAAATTTGCACTGACTTGCTTTAGCACTCAATTTGCTTTTGCTTG
tcc tga cgg cgt aaa aca cgt cta tca gtt
ACGTGCCAGATCAGTTTCACCTAAACTGTTCATCAGACAAGAGGAAGTTCA

29741 19 5 2 27
TGTAACATTAGGGAGGACTTGAAAGAGCCACCACATTTTCACCGAGGCCA
cgc gga gta cga tcg agt g
TACAGTGAACAATGCTAGGGAGAGCTGCCTATATGGAAGAGCCCTAATGTG

29747 22 1 2 12
ATTAGGGAGGACTTGAAAGAGCCACCACATTTTCACCGAGGCCACGCGGA
gta cga tcg agt gta cag tga a
CAATGCTAGGGAGAGCTGCCTATATGGAAGAGCCCTAATGTGTAAAATTAA

27751 18 3 1 164 ORF7a-TE*120del
TTGTTGCGGCAATAGTGTTTATAACACTTTGCTTCACACTCAAAAGAAAG
aca gaa tga ttg aac ttt
CATTAATTGACTTCTATTTGTGCTTTTTAGCCTTTCTGCTATTCCTTGTTT

27751 18 3 1 164 ORF7b-MIELS1fs
TTGTTGCGGCAATAGTGTTTATAACACTTTGCTTCACACTCAAAAGAAAG
aca gaa tga ttg aac ttt
CATTAATTGACTTCTATTTGTGCTTTTTAGCCTTTCTGCTATTCCTTGTTT

21984 12 86 1 156 S-LGVY141del
TAATGTTGTTATTAAAGTCTGTGAATTTCAATTTTGTAATGATCCATTTT
tgg gtg ttt att
ACCACAAAAACAACAAAAGTTGGATGGAAAGTGAGTTCAGAGTTTATTCTA

29738 21 36 1 156
GTGTGTAACATTAGGGAGGACTTGAAAGAGCCACCACATTTTCACCGAGG
cca cgc gga gta cga tcg agt
GTACAGTGAACAATGCTAGGGAGAGCTGCCTATATGGAAGAGCCCTAATGT

29743 18 5 1 112
TAACATTAGGGAGGACTTGAAAGAGCCACCACATTTTCACCGAGGCCACG
cgg agt acg atc gag tgt
ACAGTGAACAATGCTAGGGAGAGCTGCCTATATGGAAGAGCCCTAATGTGT

28900 9 12 1 79 N-RMAG209S
AGTTCAAGAAATTCAACTCCAGGCAGCAGTAGGGGAACTTCTCCTGCTAG
aat ggc tgg
CAATGGCGGTGATGCTGCTCTTGCTTTGCTGCTGCTTGACAGATTGAACCA

29730 30 24 1 63
TTTAATCAGTGTGTAACATTAGGGAGGACTTGAAAGAGCCACCACATTTT
cac cga ggc cac gcg gag tac gat cga gtg
TACAGTGAACAATGCTAGGGAGAGCTGCCTATATGGAAGAGCCCTAATGTG

29741 23 6 1 45
TGTAACATTAGGGAGGACTTGAAAGAGCCACCACATTTTCACCGAGGCCA
cgc gga gta cga tcg agt gta ca
GTGAACAATGCTAGGGAGAGCTGCCTATATGGAAGAGCCCTAATGTGTAAA

27788 9 6 1 32 ORF7b-CFL12del
ACTCAAAAGAAAGACAGAATGATTGAACTTTCATTAATTGACTTCTATTT
gtg ctt ttt
AGCCTTTCTGCTATTCCTTGTTTTAATTATGCTTATTATCTTTTGGTTCTC

29733 15 5 1 21
AATCAGTGTGTAACATTAGGGAGGACTTGAAAGAGCCACCACATTTTCAC
cga ggc cac gcg gag
TACGATCGAGTGTACAGTGAACAATGCTAGGGAGAGCTGCCTATATGGAAG

27712 15 1 1 20 ORF7a-IVFIT107del
AAGAGGAAGTTCAAGAACTTTACTCTCCAATTTTTCTTATTGTTGCGGCA
ata gtg ttt ata aca
CTTTGCTTCACACTCAAAAGAAAGACAGAATGATTGAACTTTCATTAATTG

29752 8 7 1 13
GGAGGACTTGAAAGAGCCACCACATTTTCACCGAGGCCACGCGGAGTACG
atc gag tg
TACAGTGAACAATGCTAGGGAGAGCTGCCTATATGGAAGAGCCCTAATGTG

27764 15 5 1 13 ORF7b-ELSLI3del
AGTGTTTATAACACTTTGCTTCACACTCAAAAGAAAGACAGAATGATTGA
act ttc att aat tga
CTTCTATTTGTGCTTTTTAGCCTTTCTGCTATTCCTTGTTTTAATTATGCT

29734 27 4 1 12
ATCAGTGTGTAACATTAGGGAGGACTTGAAAGAGCCACCACATTTTCACC
gag gcc acg cgg agt acg atc gag tgt
ACAGTGAACAATGCTAGGGAGAGCTGCCTATATGGAAGAGCCCTAATGTGT

29730 18 4 1 11
TTTAATCAGTGTGTAACATTAGGGAGGACTTGAAAGAGCCACCACATTTT
cac cga ggc cac gcg gag
TACGATCGAGTGTACAGTGAACAATGCTAGGGAGAGCTGCCTATATGGAAG

29724 25 2 1 11
GCAATCTTTAATCAGTGTGTAACATTAGGGAGGACTTGAAAGAGCCACCA
cat ttt cac cga ggc cac gcg gag t
ACGATCGAGTGTACAGTGAACAATGCTAGGGAGAGCTGCCTATATGGAAGA

29760 19 3 1 10
TGAAAGAGCCACCACATTTTCACCGAGGCCACGCGGAGTACGATCGAGTG
tac agt gaa caa tgc tag g
GAGAGCTGCCTATATGGAAGAGCCCTAATGTGTAAAATTAATTTTAGTAGT

29733 30 2 1 9
AATCAGTGTGTAACATTAGGGAGGACTTGAAAGAGCCACCACATTTTCAC
cga ggc cac gcg gag tac gat cga gtg tac
AGTGAACAATGCTAGGGAGAGCTGCCTATATGGAAGAGCCCTAATGTGTAA

29750 17 2 1 9
AGGGAGGACTTGAAAGAGCCACCACATTTTCACCGAGGCCACGCGGAGTA
cga tcg agt gta cag tg
AACAATGCTAGGGAGAGCTGCCTATATGGAAGAGCCCTAATGTGTAAAATT

29813 9 1 1 9
AGTGAACAATGCTAGGGAGAGCTGCCTATATGGAAGAGCCCTAATGTGTA
aaa tta att

TTAGTAGTGCTATCCCCATGTGATTTTAATAGCTTCTTAGGAGAATGACAA

29764 7 5 1 7
AGAGCCACCACATTTTCACCGAGGCCACGCGGAGTACGATCGAGTGTACA
gtg aac a
ATGCTAGGGAGAGCTGCCTATATGGAAGAGCCCTAATGTGTAAAATTAATT

22293 12 5 1 7 S-LHRS244del
AGATTTGCCAATAGGTATTAACATCACTAGGTTTCAAACTTTACTTGCTT
tac ata gaa gtt
ATTTGACTCCTGGTGATTCTTCTTCAGGTTGGACAGCTGGTGCTGCAGCTT

23585 15 3 1 7 S-QTQTN675del
ACTCATATGAGTGTGACATACCCATTGGTGCAGGTATATGCGCTAGTTAT
cag act cag act aat
TCTCCTCGGCGGGCACGTAGTGTAGCTAGTCAATCCATCATTGCCTACACT

3876 18 2 1 3 ORF1a-AEIPKE1204del
TTCAAGCTTTTTGGAAATGAAGAGTGAAAAGCAAGTTGAACAAAAGATCG
ctg aga ttc cta aag agg
AAGTTAAGCCATTTATAACTGAAAGTAAACCTTCAGTTGAACAGAGAAAAC

27602 25 1 1 3 ORF7a-GVKHVYQLR70fs
ATTTGCACTGACTTGCTTTAGCACTCAATTTGCTTTTGCTTGTCCTGACG
gcg taa aac acg tct atc agt tac g
TGCCAGATCAGTTTCACCTAAACTGTTCATCAGACAAGAGGAAGTTCAAGA

29738 14 32 0 403
GTGTGTAACATTAGGGAGGACTTGAAAGAGCCACCACATTTTCACCGAGG
cca cgc gga gta cg
ATCGAGTGTACAGTGAACAATGCTAGGGAGAGCTGCCTATATGGAAGAGCC

29767 7 2 0 209
GCCACCACATTTTCACCGAGGCCACGCGGAGTACGATCGAGTGTACAGTG
aac aat g
CTAGGGAGAGCTGCCTATATGGAAGAGCCCTAATGTGTAAAATTAATTTTA

29732 29 2 0 200
TAATCAGTGTGTAACATTAGGGAGGACTTGAAAGAGCCACCACATTTTCA
ccg agg cca cgc gga gta cga tcg agt gt
ACAGTGAACAATGCTAGGGAGAGCTGCCTATATGGAAGAGCCCTAATGTGT

29736 24 23 0 183
CAGTGTGTAACATTAGGGAGGACTTGAAAGAGCCACCACATTTTCACCGA
ggc cac gcg gag tac gat cga gtg
TACAGTGAACAATGCTAGGGAGAGCTGCCTATATGGAAGAGCCCTAATGTG

27266 27 71 0 125 ORF6-FKVSIWNLD22del
TGACTTTCAGGTTACTATAGCAGAGATATTACTAATTATTATGAGGACTT
tta aag ttt cca ttt gga atc ttg att
ACATCATAAACCTCATAATTAAAAATTTATCTAAGTCACTAACTGAGAATA

29738 22 7 0 120
GTGTGTAACATTAGGGAGGACTTGAAAGAGCCACCACATTTTCACCGAGG

cca cgc gga gta cga tcg agt g
TACAGTGAACAATGCTAGGGAGAGCTGCCTATATGGAAGAGCCCTAATGTG

29731 8 38 0 118
TTAATCAGTGTGTAACATTAGGGAGGACTTGAAAGAGCCACCACATTTTC
acc gag gc
CACGCGGAGTACGATCGAGTGTACAGTGAACAATGCTAGGGAGAGCTGCCT

29762 15 2 0 105
AAAGAGCCACCACATTTTCACCGAGGCCACGCGGAGTACGATCGAGTGTA
cag tga aca atg cta
GGGAGAGCTGCCTATATGGAAGAGCCCTAATGTGTAAAATTAATTTTAGTA

26164 8 12 0 103 ORF3a-PVM258fs
AAGAACATGTCCAAATTCACACAATCGACGGTTCATCCGGAGTTGTTAAT
cca gta at
GGAACCAATTTATGATGAACCGACGACGACTACTAGCGTGCCTTTGTAAGC

26159 11 3 0 103 ORF3a-VNPV256fs
GCCTGAAGAACATGTCCAAATTCACACAATCGACGGTTCATCCGGAGTTG
tta atc cag ta
ATGGAACCAATTTATGATGAACCGACGACGACTACTAGCGTGCCTTTGTAA

27757 7 1 0 99 ORF7a-*122del
CGGCAATAGTGTTTATAACACTTTGCTTCACACTCAAAAGAAAGACAGAA
tga ttg a
ACTTTCATTAATTGACTTCTATTTGTGCTTTTTAGCCTTTCTGCTATTCCT

27757 7 1 0 99 ORF7b-MIE1fs
CGGCAATAGTGTTTATAACACTTTGCTTCACACTCAAAAGAAAGACAGAA
tga ttg a
ACTTTCATTAATTGACTTCTATTTGTGCTTTTTAGCCTTTCTGCTATTCCT

27571 16 5 0 97 ORF7a-STQFAF60fs
ATTCACCATTTCATCCTCTAGCTGATAACAAATTTGCACTGACTTGCTTT
agc act caa ttt gct t
TTGCTTGTCCTGACGGCGTAAAACACGTCTATCAGTTACGTGCCAGATCAG

29762 11 1 0 91
AAAGAGCCACCACATTTTCACCGAGGCCACGCGGAGTACGATCGAGTGTA
cag tga aca at
GCTAGGGAGAGCTGCCTATATGGAAGAGCCCTAATGTGTAAAATTAATTTT

28090 7 31 0 90 ORF8-GSK66fs
AGGAGCTAGAAAATCAGCACCTTTAATTGAATTGTGCGTGGATGAGGCTG
gtt cta a
ATCACCCATTCAGTACATCGATATCGGTAATTATACAGTTTCCTGTTTACC

28882 18 13 0 86 N-GTSPAR204del
TCATCACGTAGTCGCAACAGTTCAAGAAATTCAACTCCAGGCAGCAGTAG
ggg aac ttc tcc tgc tag
AATGGCTGGCAATGGCGGTGATGCTGCTCTTGCTTTGCTGCTGCTTGACAG

6656 24 4 0 79 ORF1a-NSVPWDTI2131del

CTAGAGTATTAGGTTTGAAAACCCTTGCTACTCATGGTTTAGCTGCTGTT
aat agt gtc cct tgg gat act ata
GCTAATTATGCTAAGCCTTTTCTTAACAAAGTTGTTAGTACAACTACTAAC

516 9 45 0 77 ORF1a-VMV84del
CTATGTGTTCATCAAACGTTCGGATGCTCGAACTGCACCTCATGGTCATG
tta tgg ttg
AGCTGGTAGCAGAACTCGAAGGCATTCAGTACGGTCGTAGTGGTGAGACAC

22286 7 4 0 75 S-LAL242fs
CATTGGTAGATTTGCCAATAGGTATTAACATCACTAGGTTTCAAACTTTA
ctt gct t
TACATAGAAGTTATTTGACTCCTGGTGATTCTTCTTCAGGTTGGACAGCTG

29767 10 13 0 73
GCCACCACATTTTCACCGAGGCCACGCGGAGTACGATCGAGTGTACAGTG
aac aat gct a
GGGAGAGCTGCCTATATGGAAGAGCCCTAATGTGTAAAATTAATTTTAGTA

11287 9 7 0 73 ORF1a-LSG3674del
AGTTGGGTGATGCGTATTATGACATGGTTGGATATGGTTGATACTAGTTT
gtc tgg ttt
TAAGCTAAAAGACTGTGTTATGTATGCATCAGCTGTAGTGTTACTAATCCT

27626 13 4 0 73 ORF7a-RARSV78fs
TCAATTTGCTTTTGCTTGTCCTGACGGCGTAAAACACGTCTATCAGTTAC
gtg cca gat cag t
TTCACCTAAACTGTTCATCAGACAAGAGGAAGTTCAAGAACTTTACTCTCC

6659 27 15 0 69 ORF1a-SVPWDTIAN2132del
GAGTATTAGGTTTGAAAACCCTTGCTACTCATGGTTTAGCTGCTGTTAAT
agt gtc cct tgg gat act ata gct aat
TATGCTAAGCCTTTTCTTAACAAAGTTGTTAGTACAACTACTAACATAGTT

26158 12 21 0 68 ORF3a-VNPV256del
AGCCTGAAGAACATGTCCAAATTCACACAATCGACGGTTCATCCGGAGTT
gtt aat cca gta
ATGGAACCAATTTATGATGAACCGACGACGACTACTAGCGTGCCTTTGTAA

28146 16 1 0 68 ORF8-PFTINC85fs
AATCACCCATTCAGTACATCGATATCGGTAATTATACAGTTTCCTGTTTA
cct ttt aca att aat t
GCCAGGAACCTAAATTGGGTAGTCTTGTAGTGCGTTGTTCGTTCTATGAAG

27600 18 1 0 62 ORF7a-GVKHVY70del
AAATTTGCACTGACTTGCTTTAGCACTCAATTTGCTTTTGCTTGTCCTGA
cgg cgt aaa aca cgt cta
TCAGTTACGTGCCAGATCAGTTTCACCTAAACTGTTCATCAGACAAGAGGA

27575 23 1 0 59 ORF7a-TQFAFACP61fs
ACCATTTCATCCTCTAGCTGATAACAAATTTGCACTGACTTGCTTTAGCA
ctc aat ttg ctt ttg ctt gtc ct
GACGGCGTAAAACACGTCTATCAGTTACGTGCCAGATCAGTTTCACCTAAA

27571 18 25 0 50 ORF7a-STQFAF60del
ATTCACCATTTCATCCTCTAGCTGATAACAAATTTGCACTGACTTGCTTT
agc act caa ttt gct ttt
GCTTGTCCTGACGGCGTAAAACACGTCTATCAGTTACGTGCCAGATCAGTT

3333 12 15 0 50 ORF1a-IEVN1023del
TGAGGTTCAACCTCAATTAGAGATGGAACTTACACCAGTTGTTCAGACTA
ttg aag tga ata
GTTTTAGTGGTTATTTAAAACTTACTGACAATGTATACATTAAAAATGCAG

27696 7 17 0 49 ORF7a-FLI101fs
AAACTGTTCATCAGACAAGAGGAAGTTCAAGAACTTTACTCTCCAATTTT
tct tat t
GTTGCGGCAATAGTGTTTATAACACTTTGCTTCACACTCAAAAGAAAGACA

25423 9 25 0 48 ORF3a-GTV11del
CATTACACATAAACGAACTTATGGATTTGTTTATGAGAATCTTCACAATT
gga act gta
ACTTTGAAGCAAGGTGAAATCAAGGATGCTACTCCTTCAGATTTTGTTCGC

29753 9 7 0 48
GAGGACTTGAAAGAGCCACCACATTTTCACCGAGGCCACGCGGAGTACGA
tcg agt gta
CAGTGAACAATGCTAGGGAGAGCTGCCTATATGGAAGAGCCCTAATGTGTA

27574 17 2 0 46 ORF7a-TQFAFA61fs
CACCATTTCATCCTCTAGCTGATAACAAATTTGCACTGACTTGCTTTAGC
act caa ttt gct ttt gc
TTGTCCTGACGGCGTAAAACACGTCTATCAGTTACGTGCCAGATCAGTTTC

29739 16 5 0 43
TGTGTAACATTAGGGAGGACTTGAAAGAGCCACCACATTTTCACCGAGGC
cac gcg gag tac gat c
GAGTGTACAGTGAACAATGCTAGGGAGAGCTGCCTATATGGAAGAGCCCTA

27561 16 2 0 42 ORF7a-LTCFST56fs
TACGAGGGCAATTCACCATTTCATCCTCTAGCTGATAACAAATTTGCACT
gac ttg ctt tag cac t
CAATTTGCTTTTGCTTGTCCTGACGGCGTAAAACACGTCTATCAGTTACGT

27566 28 4 0 41 ORF7a-CFSTQFAFAC58fs
GGGCAATTCACCATTTCATCCTCTAGCTGATAACAAATTTGCACTGACTT
gct tta gca ctc aat ttg ctt ttg ctt g
TCCTGACGGCGTAAAACACGTCTATCAGTTACGTGCCAGATCAGTTTCACC

27877 10 1 0 41 ORF7b-CHA*41fs
GCTTATTATCTTTTGGTTCTCACTTGAACTGCAAGATCATAATGAAACTT
gtc acg cct a
AACGAACATGAAATTTCTTGTTTTCTTAGGAATCATCACAACTGTAGCTGC

26485 8 1 0 40
ATCTGAATTCTTCTAGAGTTCCTGATCTTCTGGTCTAAACGAACTAAATA
tta tat ta
GTTTTTCTGTTTGGAACTTTAATTTTAGCCATGGCAGATTCCAACGGTACT

26162 9 20 0 39 ORF3a-NPV257del
TGAAGAACATGTCCAAATTCACACAATCGACGGTTCATCCGGAGTTGTTA
atc cag taa
TGGAACCAATTTATGATGAACCGACGACGACTACTAGCGTGCCTTTGTAAG

27428 9 1 0 37 ORF7a-LATC12R
GATTGATTAAACGAACATGAAAATTATTCTTTTCTTGGCACTGATAACAC
tcg cta ctt
GTGAGCTTTATCACTACCAAGAGTGTGTTAGAGGTACAACAGTACTTTTAA

27555 24 21 0 36 ORF7a-FALTCFSTQ54L
GGAACATACGAGGGCAATTCACCATTTCATCCTCTAGCTGATAACAAATT
tgc act gac ttg ctt tag cac tca
ATTTGCTTTTGCTTGTCCTGACGGCGTAAAACACGTCTATCAGTTACGTGC

27570 19 1 0 36 ORF7a-FSTQFAF59fs
AATTCACCATTTCATCCTCTAGCTGATAACAAATTTGCACTGACTTGCTT
tag cac tca att tgc ttt t
GCTTGTCCTGACGGCGTAAAACACGTCTATCAGTTACGTGCCAGATCAGTT

27683 12 17 0 35 ORF7a-YSPI97del
ATCAGTTTCACCTAAACTGTTCATCAGACAAGAGGAAGTTCAAGAACTTT
act ctc caa ttt
TTCTTATTGTTGCGGCAATAGTGTTTATAACACTTTGCTTCACACTCAAAA

21974 21 6 0 35 S-DPFLGVY138del
ATAACGCTACTAATGTTGTTATTAAAGTCTGTGAATTTCAATTTTGTAAT
gat cca ttt ttg ggt gtt tat
TACCACAAAAACAACAAAAGTTGGATGGAAAGTGAGTTCAGAGTTTATTCT

29745 15 5 0 32
ACATTAGGGAGGACTTGAAAGAGCCACCACATTTTCACCGAGGCCACGCG
gag tac gat cga gtg
TACAGTGAACAATGCTAGGGAGAGCTGCCTATATGGAAGAGCCCTAATGTG

28230 26 2 0 32 ORF8-DVRVVLDFI113fs
GTAGTCTTGTAGTGCGTTGTTCGTTCTATGAAGACTTTTTAGAGTATCAT
gac gtt cgt gtt gtt tta gat ttc at
CTAAACGAACAAACTAAAATGTCTGATAATGGACCCCAAAATCAGCGAAAT

29738 29 16 0 31
GTGTGTAACATTAGGGAGGACTTGAAAGAGCCACCACATTTTCACCGAGG
cca cgc gga gta cga tcg agt gta cag tg
AACAATGCTAGGGAGAGCTGCCTATATGGAAGAGCCCTAATGTGTAAAATT

27629 11 3 0 31 ORF7a-ARSV79fs
ATTTGCTTTTGCTTGTCCTGACGGCGTAAAACACGTCTATCAGTTACGTG
cca gat cag tt
TCACCTAAACTGTTCATCAGACAAGAGGAAGTTCAAGAACTTTACTCTCCA

27694 9 14 0 30 ORF7a-FLI101del
CTAAACTGTTCATCAGACAAGAGGAAGTTCAAGAACTTTACTCTCCAATT
ttt ctt att

GTTGCGGCAATAGTGTTTATAACACTTTGCTTCACACTCAAAAGAAAGACA

25424 12 11 0 30 ORF3a-GTVTL11V
ATTACACATAAACGAACTTATGGATTTGTTTATGAGAATCTTCACAATTG
gaa ctg taa ctt
TGAAGCAAGGTGAAATCAAGGATGCTACTCCTTCAGATTTTGTTCGCGCTA

25438 18 6 0 30 ORF3a-KQGEIK16del
AACTTATGGATTTGTTTATGAGAATCTTCACAATTGGAACTGTAACTTTG
aag caa ggt gaa atc aag
GATGCTACTCCTTCAGATTTTGTTCGCGCTACTGCAACGATACCGATACAA

21985 9 11 0 29 S-LGVY141F
AATGTTGTTATTAAAGTCTGTGAATTTCAATTTTGTAATGATCCATTTTT
ggg tgt tta
TTACCACAAAAACAACAAAAGTTGGATGGAAAGTGAGTTCAGAGTTTATTC

29577 8 1 0 29 ORF10-FAF7fs
GGCCTAAACTCATGCAGACCACACAAGGCAGATGGGCTATATAAACGTTT
tcg ctt tt
CCGTTTACGATATATAGTCTACTCTTGTGCAGAATGAATTCTCGTAACTAC

28150 12 1 0 29 ORF8-FTIN86del
ACCCATTCAGTACATCGATATCGGTAATTATACAGTTTCCTGTTTACCTT
tta caa tta att
GCCAGGAACCTAAATTGGGTAGTCTTGTAGTGCGTTGTTCGTTCTATGAAG

28144 8 1 0 27 ORF8-LPF84fs
TAAATCACCCATTCAGTACATCGATATCGGTAATTATACAGTTTCCTGTT
tac ctt tt
ACAATTAATTGCCAGGAACCTAAATTGGGTAGTCTTGTAGTGCGTTGTTCG

22286 15 2 0 26 S-LALHR242del
CATTGGTAGATTTGCCAATAGGTATTAACATCACTAGGTTTCAAACTTTA
ctt gct tta cat aga
AGTTATTTGACTCCTGGTGATTCTTCTTCAGGTTGGACAGCTGGTGCTGCA

509 9 8 0 25 ORF1a-GHV82del
AACAGCCCTATGTGTTCATCAAACGTTCGGATGCTCGAACTGCACCTCAT
ggt cat gtt
ATGGTTGAGCTGGTAGCAGAACTCGAAGGCATTCAGTACGGTCGTAGTGGT

29734 29 3 0 25
ATCAGTGTGTAACATTAGGGAGGACTTGAAAGAGCCACCACATTTTCACC
gag gcc acg cgg agt acg atc gag tgt ac
AGTGAACAATGCTAGGGAGAGCTGCCTATATGGAAGAGCCCTAATGTGTAA

29736 14 13 0 24
CAGTGTGTAACATTAGGGAGGACTTGAAAGAGCCACCACATTTTCACCGA
ggc cac gcg gag ta
CGATCGAGTGTACAGTGAACAATGCTAGGGAGAGCTGCCTATATGGAAGAG

28242 10 10 0 24 ORF8-VLDF117fs
TGCGTTGTTCGTTCTATGAAGACTTTTTAGAGTATCATGACGTTCGTGTT

gtt tta gat t
TCATCTAAACGAACAAACTAAAATGTCTGATAATGGACCCCAAAATCAGCG

28245 8 9 0 24 ORF8-LDF118fs
GTTGTTCGTTCTATGAAGACTTTTTAGAGTATCATGACGTTCGTGTTGTT
tta gat tt
CATCTAAACGAACAAACTAAAATGTCTGATAATGGACCCCAAAATCAGCGA

27570 9 6 0 24 ORF7a-FSTQ59L
AATTCACCATTTCATCCTCTAGCTGATAACAAATTTGCACTGACTTGCTT
tag cac tca
ATTTGCTTTTGCTTGTCCTGACGGCGTAAAACACGTCTATCAGTTACGTGC

21762 9 1 0 24 S-AIH67del
AACTCAGGACTTGTTCTTACCTTTCTTTTCCAATGTTACTTGGTTCCATG
cta tac atg
TCTCTGGGACCAATGGTACTAAGAGGTTTGATAACCCTGTCCTACCATTTA

28881 9 12 0 23 N-RGTS203T
CTCATCACGTAGTCGCAACAGTTCAAGAAATTCAACTCCAGGCAGCAGTA
ggg gaa ctt
CTCCTGCTAGAATGGCTGGCAATGGCGGTGATGCTGCTCTTGCTTTGCTGC

27571 17 13 0 22 ORF7a-STQFAF60fs
ATTCACCATTTCATCCTCTAGCTGATAACAAATTTGCACTGACTTGCTTT
agc act caa ttt gct tt
TGCTTGTCCTGACGGCGTAAAACACGTCTATCAGTTACGTGCCAGATCAGT

27626 18 1 0 22 ORF7a-RARSVS78del
TCAATTTGCTTTTGCTTGTCCTGACGGCGTAAAACACGTCTATCAGTTAC
gtg cca gat cag ttt cac
CTAAACTGTTCATCAGACAAGAGGAAGTTCAAGAACTTTACTCTCCAATTT

27677 9 9 0 21 ORF7a-ELYS95A
TGCCAGATCAGTTTCACCTAAACTGTTCATCAGACAAGAGGAAGTTCAAG
aac ttt act
CTCCAATTTTTCTTATTGTTGCGGCAATAGTGTTTATAACACTTTGCTTCA

28068 8 2 0 20 ORF8-ELC59fs
ATTCTAAATGGTATATTAGAGTAGGAGCTAGAAAATCAGCACCTTTAATT
gaa ttg tg
CGTGGATGAGGCTGGTTCTAAATCACCCATTCAGTACATCGATATCGGTAA

11512 9 14 0 19 ORF1a-TVM3750del
ATGTGGGCTCTTATAATCTCTGTTACTTCTAACTACTCAGGTGTAGTTAC
aac tgt cat
GTTTTTGGCCAGAGGTATTGTTTTTATGTGTGTTGAGTATTGCCCTATTTT

29739 10 4 0 19
TGTGTAACATTAGGGAGGACTTGAAAGAGCCACCACATTTTCACCGAGGC
cac gcg gag t
ACGATCGAGTGTACAGTGAACAATGCTAGGGAGAGCTGCCTATATGGAAGA

29578 11 2 0 19 ORF10-FAFP7fs

GCCTAAACTCATGCAGACCACACAAGGCAGATGGGCTATATAAACGTTTT
cgc ttt tcc gt
TTACGATATATAGTCTACTCTTGTGCAGAATGAATTCTCGTAACTACATAG

27689 9 11 0 18 ORF7a-PIF99del
TTCACCTAAACTGTTCATCAGACAAGAGGAAGTTCAAGAACTTTACTCTC
caa ttt ttc
TTATTGTTGCGGCAATAGTGTTTATAACACTTTGCTTCACACTCAAAAGAA

29739 24 8 0 18
TGTGTAACATTAGGGAGGACTTGAAAGAGCCACCACATTTTCACCGAGGC
cac gcg gag tac gat cga gtg tac
AGTGAACAATGCTAGGGAGAGCTGCCTATATGGAAGAGCCCTAATGTGTAA

27797 7 6 0 18 ORF7b-LAF14fs
AAAGACAGAATGATTGAACTTTCATTAATTGACTTCTATTTGTGCTTTTT
agc ctt t
CTGCTATTCCTTGTTTTAATTATGCTTATTATCTTTTGGTTCTCACTTGAA

27266 28 5 0 18 ORF6-FKVSIWNLDY22fs
TGACTTTCAGGTTACTATAGCAGAGATATTACTAATTATTATGAGGACTT
tta aag ttt cca ttt gga atc ttg att a
CATCATAAACCTCATAATTAAAAATTTATCTAAGTCACTAACTGAGAATAA

27677 7 1 0 18 ORF7a-ELY95fs
TGCCAGATCAGTTTCACCTAAACTGTTCATCAGACAAGAGGAAGTTCAAG
aac ttt a
CTCTCCAATTTTTCTTATTGTTGCGGCAATAGTGTTTATAACACTTTGCTT

25423 15 9 0 17 ORF3a-GTVTL11del
CATTACACATAAACGAACTTATGGATTTGTTTATGAGAATCTTCACAATT
gga act gta act ttg
AAGCAAGGTGAAATCAAGGATGCTACTCCTTCAGATTTTGTTCGCGCTACT

28877 12 8 0 17 N-RGTS203del
GTTCCTCATCACGTAGTCGCAACAGTTCAAGAAATTCAACTCCAGGCAGC
agt agg gga act
TCTCCTGCTAGAATGGCTGGCAATGGCGGTGATGCTGCTCTTGCTTTGCTG

28077 7 6 0 16 ORF8-VDE62fs
GGTATATTAGAGTAGGAGCTAGAAAATCAGCACCTTTAATTGAATTGTGC
gtg gat g
AGGCTGGTTCTAAATCACCCATTCAGTACATCGATATCGGTAATTATACAG

29739 12 3 0 16
TGTGTAACATTAGGGAGGACTTGAAAGAGCCACCACATTTTCACCGAGGC
cac gcg gag tac
GATCGAGTGTACAGTGAACAATGCTAGGGAGAGCTGCCTATATGGAAGAGC

25430 18 2 0 16 ORF3a-VTLKQG13del
CATAAACGAACTTATGGATTTGTTTATGAGAATCTTCACAATTGGAACTG
taa ctt tga agc aag gtg
AAATCAAGGATGCTACTCCTTCAGATTTTGTTCGCGCTACTGCAACGATAC

3335 15 9 0 15 ORF1a-EVNSF1024del
AGGTTCAACCTCAATTAGAGATGGAACTTACACCAGTTGTTCAGACTATT
gaa gtg aat agt ttt
AGTGGTTATTTAAAACTTACTGACAATGTATACATTAAAAATGCAGACATT

27923 8 3 0 15 ORF8-ITT10fs
ACTTGTCACGCCTAAACGAACATGAAATTTCTTGTTTTCTTAGGAATCAT
cac aac tg
TAGCTGCATTTCACCAAGAATGTAGTTTACAGTCATGTACTCAACATCAAC

25433 21 3 0 15 ORF3a-TLKQGEI14del
AAACGAACTTATGGATTTGTTTATGAGAATCTTCACAATTGGAACTGTAA
ctt tga agc aag gtg aaa tca
AGGATGCTACTCCTTCAGATTTTGTTCGCGCTACTGCAACGATACCGATAC

27933 20 3 0 15 ORF8-AAFHQEC14fs
CCTAAACGAACATGAAATTTCTTGTTTTCTTAGGAATCATCACAACTGTA
gct gca ttt cac caa gaa tg
TAGTTTACAGTCATGTACTCAACATCAACCATATGTAGTTGATGACCCGTG

27957 7 3 0 15 ORF8-LQS22fs
TTTTCTTAGGAATCATCACAACTGTAGCTGCATTTCACCAAGAATGTAGT
tta cag t
CATGTACTCAACATCAACCATATGTAGTTGATGACCCGTGTCCTATTCACT

27269 22 1 0 15 ORF6-KVSIWNLD23fs
CTTTCAGGTTACTATAGCAGAGATATTACTAATTATTATGAGGACTTTTA
aag ttt cca ttt gga atc ttg a
TTACATCATAAACCTCATAATTAAAAATTTATCTAAGTCACTAACTGAGAA

3900 9 1 0 15 ORF1a-KPF1212del
TGAAAAGCAAGTTGAACAAAAGATCGCTGAGATTCCTAAAGAGGAAGTTA
agc cat tta
TAACTGAAAGTAAACCTTCAGTTGAACAGAGAAAACAAGATGATAAGAAAA

25471 9 1 0 15 ORF3a-DFV27del
TTGGAACTGTAACTTTGAAGCAAGGTGAAATCAAGGATGCTACTCCTTCA
gat ttt gtt
CGCGCTACTGCAACGATACCGATACAAGCCTCACTCCCTTTCGGATGGCTT

27683 14 9 0 14 ORF7a-YSPIF97fs
ATCAGTTTCACCTAAACTGTTCATCAGACAAGAGGAAGTTCAAGAACTTT
act ctc caa ttt tt
CTTATTGTTGCGGCAATAGTGTTTATAACACTTTGCTTCACACTCAAAAGA

27688 11 7 0 14 ORF7a-PIFL99fs
TTTCACCTAAACTGTTCATCAGACAAGAGGAAGTTCAAGAACTTTACTCT
cca att ttt ct
TATTGTTGCGGCAATAGTGTTTATAACACTTTGCTTCACACTCAAAAGAAA

27267 27 7 0 14 ORF6-KVSIWNLDY23del
GACTTTCAGGTTACTATAGCAGAGATATTACTAATTATTATGAGGACTTT
taa agt ttc cat ttg gaa tct tga tta
CATCATAAACCTCATAATTAAAAATTTATCTAAGTCACTAACTGAGAATAA

3549 9 6 0 14 ORF1a-GPLK1095E
TACTAACAATGCCATGCAAGTTGAATCTGATGATTACATAGCTACTAATG
gac cac tta
AAGTGGGTGGTAGTTGTGTTTTAAGCGGACACAATCTTGCTAAACACTGTC

29754 9 1 0 14
AGGACTTGAAAGAGCCACCACATTTTCACCGAGGCCACGCGGAGTACGAT
cga gtg tac
AGTGAACAATGCTAGGGAGAGCTGCCTATATGGAAGAGCCCTAATGTGTAA

27752 12 9 0 13 ORF7a-TE*120fs
TGTTGCGGCAATAGTGTTTATAACACTTTGCTTCACACTCAAAAGAAAGA
cag aat gat tga
ACTTTCATTAATTGACTTCTATTTGTGCTTTTTAGCCTTTCTGCTATTCCT

27752 12 9 0 13 ORF7b-MIE1fs
TGTTGCGGCAATAGTGTTTATAACACTTTGCTTCACACTCAAAAGAAAGA
cag aat gat tga
ACTTTCATTAATTGACTTCTATTTGTGCTTTTTAGCCTTTCTGCTATTCCT

8602 12 5 0 13 ORF1a-FLFV2780del
GGTAAAATTGTTAATAATTGGTTGAAGCAGTTAATTAAAGTTACACTTGT
gtt cct ttt tgt
TGCTGCTATTTTCTATTTAATAACACCTGTTCATGTCATGTCTAAACATAC

27682 18 5 0 13 ORF7a-YSPIFL97del
GATCAGTTTCACCTAAACTGTTCATCAGACAAGAGGAAGTTCAAGAACTT
tac tct cca att ttt ctt
ATTGTTGCGGCAATAGTGTTTATAACACTTTGCTTCACACTCAAAAGAAAG

27787 10 4 0 13 ORF7b-LCFL11fs
CACTCAAAAGAAAGACAGAATGATTGAACTTTCATTAATTGACTTCTATT
tgt gct ttt t
AGCCTTTCTGCTATTCCTTGTTTTAATTATGCTTATTATCTTTTGGTTCTC

27683 11 3 0 13 ORF7a-YSPI97fs
ATCAGTTTCACCTAAACTGTTCATCAGACAAGAGGAAGTTCAAGAACTTT
act ctc caa tt
TTTCTTATTGTTGCGGCAATAGTGTTTATAACACTTTGCTTCACACTCAAA

29737 23 3 0 13
AGTGTGTAACATTAGGGAGGACTTGAAAGAGCCACCACATTTTCACCGAG
gcc acg cgg agt acg atc gag tg
TACAGTGAACAATGCTAGGGAGAGCTGCCTATATGGAAGAGCCCTAATGTG

26158 24 8 0 12 ORF3a-VNPVMEPI256del
AGCCTGAAGAACATGTCCAAATTCACACAATCGACGGTTCATCCGGAGTT
gtt aat cca gta atg gaa cca att
TATGATGAACCGACGACGACTACTAGCGTGCCTTTGTAAGCACAAGCTGAT

27210 15 6 0 12 ORF6-HLVDF3del
GCAGTGACAATATTGCTTTGCTTGTACAGTAAGTGACAACAGATGTTTCA
tct cgt tga ctt tca

GGTTACTATAGCAGAGATATTACTAATTATTATGAGGACTTTTAAAGTTTC

29749 17 5 0 12
TAGGGAGGACTTGAAAGAGCCACCACATTTTCACCGAGGCCACGCGGAGT
acg atc gag tgt aca gt
GAACAATGCTAGGGAGAGCTGCCTATATGGAAGAGCCCTAATGTGTAAAAT

27741 10 3 0 12 ORF7a-LKRK116fs
ATTTTTCTTATTGTTGCGGCAATAGTGTTTATAACACTTTGCTTCACACT
caa aag aaa g
ACAGAATGATTGAACTTTCATTAATTGACTTCTATTTGTGCTTTTTAGCCT

29737 21 3 0 12
AGTGTGTAACATTAGGGAGGACTTGAAAGAGCCACCACATTTTCACCGAG
gcc acg cgg agt acg atc gag
TGTACAGTGAACAATGCTAGGGAGAGCTGCCTATATGGAAGAGCCCTAATG

28090 22 3 0 12 ORF8-GSKSPIQY66fs
AGGAGCTAGAAAATCAGCACCTTTAATTGAATTGTGCGTGGATGAGGCTG
gtt cta aat cac cca ttc agt a
CATCGATATCGGTAATTATACAGTTTCCTGTTTACCTTTTACAATTAATTG

27724 15 2 0 12 ORF7a-TLCFT111del
AAGAACTTTACTCTCCAATTTTTCTTATTGTTGCGGCAATAGTGTTTATA
aca ctt tgc ttc aca
CTCAAAAGAAAGACAGAATGATTGAACTTTCATTAATTGACTTCTATTTGT

29579 7 2 0 12 ORF10-AFP8fs
CCTAAACTCATGCAGACCACACAAGGCAGATGGGCTATATAAACGTTTTC
gct ttt c
CGTTTACGATATATAGTCTACTCTTGTGCAGAATGAATTCTCGTAACTACA

25436 21 1 0 12 ORF3a-LKQGEIKD15Y
CGAACTTATGGATTTGTTTATGAGAATCTTCACAATTGGAACTGTAACTT
tga agc aag gtg aaa tca agg
ATGCTACTCCTTCAGATTTTGTTCGCGCTACTGCAACGATACCGATACAAG

21976 21 11 0 11 S-PFLGVYY139del
AACGCTACTAATGTTGTTATTAAAGTCTGTGAATTTCAATTTTGTAATGA
tcc att ttt ggg tgt tta tta
CCACAAAAACAACAAAAGTTGGATGGAAAGTGAGTTCAGAGTTTATTCTAG

28065 8 7 0 11 ORF8-IEL58fs
TCTATTCTAAATGGTATATTAGAGTAGGAGCTAGAAAATCAGCACCTTTA
att gaa tt
GTGCGTGGATGAGGCTGGTTCTAAATCACCCATTCAGTACATCGATATCGG

6654 30 7 0 11 ORF1a-VNSVPWDTIAN2130D
ATCTAGAGTATTAGGTTTGAAAACCCTTGCTACTCATGGTTTAGCTGCTG
tta ata gtg tcc ctt ggg ata cta tag cta
ATTATGCTAAGCCTTTTCTTAACAAAGTTGTTAGTACAACTACTAACATAG

27269 25 6 0 11 ORF6-KVSIWNLDY23fs
CTTTCAGGTTACTATAGCAGAGATATTACTAATTATTATGAGGACTTTTA

aag ttt cca ttt gga atc ttg att a
CATCATAAACCTCATAATTAAAAATTTATCTAAGTCACTAACTGAGAATAA

27558 27 6 0 11 ORF7a-LTCFSTQFA56del
ACATACGAGGGCAATTCACCATTTCATCCTCTAGCTGATAACAAATTTGC
act gac ttg ctt tag cac tca att tgc
TTTTGCTTGTCCTGACGGCGTAAAACACGTCTATCAGTTACGTGCCAGATC

27387 8 5 0 11 ORF6-*62fs
GAGAATAAATATTCTCAATTAGATGAAGAGCAACCAATGGAGATTGATTA
aac gaa ca
TGAAAATTATTCTTTTCTTGGCACTGATAACACTCGCTACTTGTGAGCTTT

27387 8 5 0 11 ORF7a-M1fs
GAGAATAAATATTCTCAATTAGATGAAGAGCAACCAATGGAGATTGATTA
aac gaa ca
TGAAAATTATTCTTTTCTTGGCACTGATAACACTCGCTACTTGTGAGCTTT

27572 7 4 0 11 ORF7a-STQ60fs
TTCACCATTTCATCCTCTAGCTGATAACAAATTTGCACTGACTTGCTTTA
gca ctc a
ATTTGCTTTTGCTTGTCCTGACGGCGTAAAACACGTCTATCAGTTACGTGC

25521 11 2 0 11 ORF3a-FGWL43fs
GATTTTGTTCGCGCTACTGCAACGATACCGATACAAGCCTCACTCCCTTT
cgg atg gct ta
TTGTTGGCGTTGCACTTCTTGCTGTTTTTCAGAGCGCTTCCAAAATCATAA

6377 18 1 0 11 ORF1a-NLACED2038del
CATCAAATTCGTTTGATGTACTGAAGTCAGAGGACGCGCAGGGAATGGAT
aat ctt gcc tgc gaa gat
CTAAAACCAGTCTCTGAAGAAGTAGTGGAAAATCCTACCATACAGAAAGAC

27490 17 1 0 11 ORF7a-EPCSSG33fs
AGCTTTATCACTACCAAGAGTGTGTTAGAGGTACAACAGTACTTTTAAAA
gaa cct tgc tct tct gg
AACATACGAGGGCAATTCACCATTTCATCCTCTAGCTGATAACAAATTTGC

28221 11 1 0 11 ORF8-EYHD110fs
CTAAATTGGGTAGTCTTGTAGTGCGTTGTTCGTTCTATGAAGACTTTTTA
gag tat cat ga
CGTTCGTGTTGTTTTAGATTTCATCTAAACGAACAAACTAAAATGTCTGAT

27267 31 1 0 11 ORF6-FKVSIWNLDYI22fs
GACTTTCAGGTTACTATAGCAGAGATATTACTAATTATTATGAGGACTTT
taa agt ttc cat ttg gaa tct tga tta cat c
ATAAACCTCATAATTAAAAATTTATCTAAGTCACTAACTGAGAATAAATAT

21977 18 9 0 10 S-PFLGVY139del
ACGCTACTAATGTTGTTATTAAAGTCTGTGAATTTCAATTTTGTAATGAT
cca ttt ttg ggt gtt tat
TACCACAAAAACAACAAAAGTTGGATGGAAAGTGAGTTCAGAGTTTATTCT

21987 9 8 0 10 S-GVYY142D

TGTTGTTATTAAAGTCTGTGAATTTCAATTTTGTAATGATCCATTTTTGG
gtg ttt att
ACCACAAAAACAACAAAAGTTGGATGGAAAGTGAGTTCAGAGTTTATTCTA

29749 25 5 0 10
TAGGGAGGACTTGAAAGAGCCACCACATTTTCACCGAGGCCACGCGGAGT
acg atc gag tgt aca gtg aac aat g
CTAGGGAGAGCTGCCTATATGGAAGAGCCCTAATGTGTAAAATTAATTTA

29762 9 4 0 10
AAAGAGCCACCACATTTTCACCGAGGCCACGCGGAGTACGATCGAGTGTA
cag tga aca
ATGCTAGGGAGAGCTGCCTATATGGAAGAGCCCTAATGTGTAAAATTAATT

27618 7 4 0 10 ORF7a-YQL75fs
TTTAGCACTCAATTTGCTTTTGCTTGTCCTGACGGCGTAAAACACGTCTA
tca gtt a
CGTGCCAGATCAGTTTCACCTAAACTGTTCATCAGACAAGAGGAAGTTCAA

27548 7 3 0 10 ORF7a-NKF52fs
CTCTTCTGGAACATACGAGGGCAATTCACCATTTCATCCTCTAGCTGATA
aca aat t
TGCACTGACTTGCTTTAGCACTCAATTTGCTTTTGCTTGTCCTGACGGCGT

29737 19 3 0 10
AGTGTGTAACATTAGGGAGGACTTGAAAGAGCCACCACATTTTCACCGAG
gcc acg cgg agt acg atc g
AGTGTACAGTGAACAATGCTAGGGAGAGCTGCCTATATGGAAGAGCCCTAA

25708 12 2 0 10 ORF3a-LYLY106del
CAGTTTACTCACACCTTTTGCTCGTTGCTGCTGGCCTTGAAGCCCCTTTT
ctc tat ctt tat
GCTTTAGTCTACTTCTTGCAGAGTATAAACTTTGTAAGAATAATAATGAGG

29731 24 1 0 10
TTAATCAGTGTGTAACATTAGGGAGGACTTGAAAGAGCCACCACATTTTC
acc gag gcc acg cgg agt acg atc
GAGTGTACAGTGAACAATGCTAGGGAGAGCTGCCTATATGGAAGAGCCCTA

29749 11 1 0 10
TAGGGAGGACTTGAAAGAGCCACCACATTTTCACCGAGGCCACGCGGAGT
acg atc gag tg
TACAGTGAACAATGCTAGGGAGAGCTGCCTATATGGAAGAGCCCTAATGTG

27615 9 1 0 10 ORF7a-YQL75del
TGCTTTAGCACTCAATTTGCTTTTGCTTGTCCTGACGGCGTAAAACACGT
cta tca gtt
ACGTGCCAGATCAGTTTCACCTAAACTGTTCATCAGACAAGAGGAAGTTCA

27566 13 1 0 10 ORF7a-CFSTQ58fs
GGGCAATTCACCATTTCATCCTCTAGCTGATAACAAATTTGCACTGACTT
gct tta gca ctc a
ATTTGCTTTTGCTTGTCCTGACGGCGTAAAACACGTCTATCAGTTACGTGC

27267 26 8 0 9 ORF6-FKVSIWNLD22fs
GACTTTCAGGTTACTATAGCAGAGATATTACTAATTATTATGAGGACTTT
taa agt ttc cat ttg gaa tct tga tt
ACATCATAAACCTCATAATTAAAAATTTATCTAAGTCACTAACTGAGAATA

28880 7 6 0 9 N-RGT203fs
CCTCATCACGTAGTCGCAACAGTTCAAGAAATTCAACTCCAGGCAGCAGT
agg gga a
CTTCTCCTGCTAGAATGGCTGGCAATGGCGGTGATGCTGCTCTTGCTTTGC

27715 9 5 0 9 ORF7a-VFI108del
AGGAAGTTCAAGAACTTTACTCTCCAATTTTTCTTATTGTTGCGGCAATA
gtg ttt ata
ACACTTTGCTTCACACTCAAAAGAAAGACAGAATGATTGAACTTTCATTAA

28888 11 5 0 9 N-TSPA205fs
CGTAGTCGCAACAGTTCAAGAAATTCAACTCCAGGCAGCAGTAGGGGAAC
ttc tcc tgc ta
GAATGGCTGGCAATGGCGGTGATGCTGCTCTTGCTTTGCTGCTGCTTGACA

27389 11 4 0 9 ORF7a-MK1del
GAATAAATATTCTCAATTAGATGAAGAGCAACCAATGGAGATTGATTAAA
cga aca tga aa
ATTATTCTTTTCTTGGCACTGATAACACTCGCTACTTGTGAGCTTTATCAC

29750 14 4 0 9
AGGGAGGACTTGAAAGAGCCACCACATTTTCACCGAGGCCACGCGGAGTA
cga tcg agt gta ca
GTGAACAATGCTAGGGAGAGCTGCCTATATGGAAGAGCCCTAATGTGTAAA

27697 9 4 0 9 ORF7a-LIV102del
AACTGTTCATCAGACAAGAGGAAGTTCAAGAACTTTACTCTCCAATTTTT
ctt att gtt
GCGGCAATAGTGTTTATAACACTTTGCTTCACACTCAAAAGAAAGACAGAA

27572 8 4 0 9 ORF7a-STQ60fs
TTCACCATTTCATCCTCTAGCTGATAACAAATTTGCACTGACTTGCTTTA
gca ctc aa
TTTGCTTTTGCTTGTCCTGACGGCGTAAAACACGTCTATCAGTTACGTGCC

27630 12 3 0 9 ORF7a-RSVS80del
TTTGCTTTTGCTTGTCCTGACGGCGTAAAACACGTCTATCAGTTACGTGC
cag atc agt ttc
ACCTAAACTGTTCATCAGACAAGAGGAAGTTCAAGAACTTTACTCTCCAAT

21971 24 1 0 9 S-NDPFLGVY137del
TTAATAACGCTACTAATGTTGTTATTAAAGTCTGTGAATTTCAATTTTGT
aat gat cca ttt ttg ggt gtt tat
TACCACAAAAACAACAAAAGTTGGATGGAAAGTGAGTTCAGAGTTTATTCT

28993 9 1 0 9 N-QQQG240H
CTTGACAGATTGAACCAGCTTGAGAGCAAAATGTCTGGTAAAGGCCAACA
aca aca agg
CCAAACTGTCACTAAGAAATCTGCTGCTGAGGCTTCTAAGAAGCCTCGGCA

28247 9 1 0 9 ORF8-LDFI118F
TGTTCGTTCTATGAAGACTTTTTAGAGTATCATGACGTTCGTGTTGTTTT
aga ttt cat
CTAAACGAACAAACTAAAATGTCTGATAATGGACCCCAAAATCAGCGAAAT

28031 21 1 0 9 ORF8-YIRVGARK46*
CCATATGTAGTTGATGACCCGTGTCCTATTCACTTCTATTCTAAATGGTA
tat tag agt agg agc tag aaa
ATCAGCACCTTTAATTGAATTGTGCGTGGATGAGGCTGGTTCTAAATCACC

27530 27 1 0 9 ORF7a-FHPLADNKFA46S
ACTTTTAAAAGAACCTTGCTCTTCTGGAACATACGAGGGCAATTCACCAT
ttc atc ctc tag ctg ata aca aat ttg
CACTGACTTGCTTTAGCACTCAATTTGCTTTTGCTTGTCCTGACGGCGTAA

21602 9 8 0 8 S-QCV14del
CTAAACGAACAATGTTTGTTTTTCTTGTTTTATTGCCACTAGTCTCTAGT
cag tgt gtt
AATCTTACAACCAGAACTCAATTACCCCCTGCATACACTAATTCTTTCACA

21973 24 7 0 8 S-DPFLGVYY138del
AATAACGCTACTAATGTTGTTATTAAAGTCTGTGAATTTCAATTTTGTAA
tga tcc att ttt ggg tgt tta tta
CCACAAAAACAACAAAAGTTGGATGGAAAGTGAGTTCAGAGTTTATTCTAG

514 12 7 0 8 ORF1a-HVMVE83Q
CCCTATGTGTTCATCAAACGTTCGGATGCTCGAACTGCACCTCATGGTCA
tgt tat ggt tga
GCTGGTAGCAGAACTCGAAGGCATTCAGTACGGTCGTAGTGGTGAGACACT

29761 13 5 0 8
GAAAGAGCCACCACATTTTCACCGAGGCCACGCGGAGTACGATCGAGTGT
aca gtg aac aat g
CTAGGGAGAGCTGCCTATATGGAAGAGCCCTAATGTGTAAAATTAATTTTA

27571 11 5 0 8 ORF7a-STQF60fs
ATTCACCATTTCATCCTCTAGCTGATAACAAATTTGCACTGACTTGCTTT
agc act caa tt
TGCTTTTGCTTGTCCTGACGGCGTAAAACACGTCTATCAGTTACGTGCCAG

29750 27 5 0 8
AGGGAGGACTTGAAAGAGCCACCACATTTTCACCGAGGCCACGCGGAGTA
cga tcg agt gta cag tga aca atg cta
GGGAGAGCTGCCTATATGGAAGAGCCCTAATGTGTAAAATTAATTTTAGTA

27617 24 4 0 8 ORF7a-YQLRARSV75del
CTTTAGCACTCAATTTGCTTTTGCTTGTCCTGACGGCGTAAAACACGTCT
atc agt tac gtg cca gat cag ttt
CACCTAAACTGTTCATCAGACAAGAGGAAGTTCAAGAACTTTACTCTCCAA

29725 17 4 0 8
CAATCTTTAATCAGTGTGTAACATTAGGGAGGACTTGAAAGAGCCACCAC
att ttc acc gag gcc ac

GCGGAGTACGATCGAGTGTACAGTGAACAATGCTAGGGAGAGCTGCCTATA

29722 20 4 0 8
TAGCAATCTTTAATCAGTGTGTAACATTAGGGAGGACTTGAAAGAGCCAC
cac att ttc acc gag gcc ac
GCGGAGTACGATCGAGTGTACAGTGAACAATGCTAGGGAGAGCTGCCTATA

29736 12 3 0 8
CAGTGTGTAACATTAGGGAGGACTTGAAAGAGCCACCACATTTTCACCGA
ggc cac gcg gag
TACGATCGAGTGTACAGTGAACAATGCTAGGGAGAGCTGCCTATATGGAAG

25448 29 2 0 8 ORF3a-EIKDATPSDF19fs
TTTGTTTATGAGAATCTTCACAATTGGAACTGTAACTTTGAAGCAAGGTG
aaa tca agg atg cta ctc ctt cag att tt
GTTCGCGCTACTGCAACGATACCGATACAAGCCTCACTCCCTTTCGGATGG

29683 8 2 0 8
TACATAGCACAAGTAGATGTAGTTAACTTTAATCTCACATAGCAATCTTT
aat cag tg
TGTAACATTAGGGAGGACTTGAAAGAGCCACCACATTTTCACCGAGGCCAC

6332 24 2 0 8 ORF1a-NSFDVLKS2023del
ATACCTGGTGTATACGTTGTCTTTGGAGCACAAAACCAGTTGAAACATCA
aat tcg ttt gat gta ctg aag tca
GAGGACGCGCAGGGAATGGATAATCTTGCCTGCGAAGATCTAAAACCAGTC

28151 7 2 0 8 ORF8-FTI86fs
CCCATTCAGTACATCGATATCGGTAATTATACAGTTTCCTGTTTACCTTT
tac aat t
AATTGCCAGGAACCTAAATTGGGTAGTCTTGTAGTGCGTTGTTCGTTCTAT

27677 20 1 0 8 ORF7a-ELYSPIF95fs
TGCCAGATCAGTTTCACCTAAACTGTTCATCAGACAAGAGGAAGTTCAAG
aac ttt act ctc caa ttt tt
CTTATTGTTGCGGCAATAGTGTTTATAACACTTTGCTTCACACTCAAAAGA

28898 21 1 0 8 N-RMAGNGG209del
ACAGTTCAAGAAATTCAACTCCAGGCAGCAGTAGGGGAACTTCTCCTGCT
aga atg gct ggc aat ggc ggt
GATGCTGCTCTTGCTTTGCTGCTGCTTGACAGATTGAACCAGCTTGAGAGC

27737 27 1 0 8 ORF7a-TLKRKTE*115fs
TCCAATTTTTCTTATTGTTGCGGCAATAGTGTTTATAACACTTTGCTTCA
cac tca aaa gaa aga cag aat gat tga
ACTTTCATTAATTGACTTCTATTTGTGCTTTTTAGCCTTTCTGCTATTCCT

27737 27 1 0 8 ORF7b-MIE1fs
TCCAATTTTTCTTATTGTTGCGGCAATAGTGTTTATAACACTTTGCTTCA
cac tca aaa gaa aga cag aat gat tga
ACTTTCATTAATTGACTTCTATTTGTGCTTTTTAGCCTTTCTGCTATTCCT

27676 8 1 0 8 ORF7a-ELY95fs
GTGCCAGATCAGTTTCACCTAAACTGTTCATCAGACAAGAGGAAGTTCAA

gaa ctt ta
CTCTCCAATTTTTCTTATTGTTGCGGCAATAGTGTTTATAACACTTTGCTT

27567 27 6 0 7 ORF7a-FSTQFAFAC59del
GGCAATTCACCATTTCATCCTCTAGCTGATAACAAATTTGCACTGACTTG
ctt tag cac tca att tgc ttt tgc ttg
TCCTGACGGCGTAAAACACGTCTATCAGTTACGTGCCAGATCAGTTTCACC

686 15 5 0 7 ORF1a-KSFDL141del
GTAAGAACGGTAATAAAGGAGCTGGTGGCCATAGTTACGGCGCCGATCTA
aag tca ttt gac tta
GGCGACGAGCTTGGCACTGATCCTTATGAAGATTTTCAAGAAAACTGGAAC

28048 15 5 0 7 ORF8-RKSAPL52I
CCCGTGTCCTATTCACTTCTATTCTAAATGGTATATTAGAGTAGGAGCTA
gaa aat cag cac ctt
TAATTGAATTGTGCGTGGATGAGGCTGGTTCTAAATCACCCATTCAGTACA

28857 9 5 0 7 N-RNS195del
CGGCAGTCAAGCCTCTTCTCGTTCCTCATCACGTAGTCGCAACAGTTCAA
gaa att caa
CTCCAGGCAGCAGTAGGGGAACTTCTCCTGCTAGAATGGCTGGCAATGGCG

28086 13 5 0 7 ORF8-AGSKS65fs
GAGTAGGAGCTAGAAAATCAGCACCTTTAATTGAATTGTGCGTGGATGAG
gct ggt tct aaa t
CACCCATTCAGTACATCGATATCGGTAATTATACAGTTTCCTGTTTACCTT

27268 21 4 0 7 ORF6-KVSIWNL23del
ACTTTCAGGTTACTATAGCAGAGATATTACTAATTATTATGAGGACTTTT
aaa gtt tcc att tgg aat ctt
GATTACATCATAAACCTCATAATTAAAAATTTATCTAAGTCACTAACTGAG

27683 18 4 0 7 ORF7a-YSPIFLI97F
ATCAGTTTCACCTAAACTGTTCATCAGACAAGAGGAAGTTCAAGAACTTT
act ctc caa ttt ttc tta
TTGTTGCGGCAATAGTGTTTATAACACTTTGCTTCACACTCAAAAGAAAGA

25521 15 4 0 7 ORF3a-WLIVG45del
GATTTTGTTCGCGCTACTGCAACGATACCGATACAAGCCTCACTCCCTTT
cgg atg gct tat tgt
TGGCGTTGCACTTCTTGCTGTTTTTCAGAGCGCTTCCAAAATCATAACCCT

27606 7 3 0 7 ORF7a-VKH71fs
GCACTGACTTGCTTTAGCACTCAATTTGCTTTTGCTTGTCCTGACGGCGT
aaa aca c
GTCTATCAGTTACGTGCCAGATCAGTTTCACCTAAACTGTTCATCAGACAA

29738 18 3 0 7
GTGTGTAACATTAGGGAGGACTTGAAAGAGCCACCACATTTTCACCGAGG
cca cgc gga gta cga tcg
AGTGTACAGTGAACAATGCTAGGGAGAGCTGCCTATATGGAAGAGCCCTAA

3348 9 3 0 7 ORF1a-FSG1028del

ATTAGAGATGGAACTTACACCAGTTGTTCAGACTATTGAAGTGAATAGTT
tta gtg gtt
ATTTAAAACTTACTGACAATGTATACATTAAAAATGCAGACATTGTGGAAG

27591 19 3 0 7 ORF7a-ACPDGVK66fs
GCTGATAACAAATTTGCACTGACTTGCTTTAGCACTCAATTTGCTTTTGC
ttg tcc tga cgg cgt aaa a
CACGTCTATCAGTTACGTGCCAGATCAGTTTCACCTAAACTGTTCATCAGA

26158 21 2 0 7 ORF3a-VNPVMEP256del
AGCCTGAAGAACATGTCCAAATTCACACAATCGACGGTTCATCCGGAGTT
gtt aat cca gta atg gaa cca
ATTTATGATGAACCGACGACGACTACTAGCGTGCCTTTGTAAGCACAAGCT

27687 21 2 0 7 ORF7a-PIFLIVA99del
GTTTCACCTAAACTGTTCATCAGACAAGAGGAAGTTCAAGAACTTTACTC
tcc aat ttt tct tat tgt tgc
GGCAATAGTGTTTATAACACTTTGCTTCACACTCAAAAGAAAGACAGAATG

27677 29 1 0 7 ORF7a-ELYSPIFLIV95fs
TGCCAGATCAGTTTCACCTAAACTGTTCATCAGACAAGAGGAAGTTCAAG
aac ttt act ctc caa ttt ttc tta ttg tt
GCGGCAATAGTGTTTATAACACTTTGCTTCACACTCAAAAGAAAGACAGAA

27574 30 1 0 7 ORF7a-TQFAFACPDG61del
CACCATTTCATCCTCTAGCTGATAACAAATTTGCACTGACTTGCTTTAGC
act caa ttt gct ttt gct tgt cct gac ggc
GTAAAACACGTCTATCAGTTACGTGCCAGATCAGTTTCACCTAAACTGTTC

6330 9 1 0 7 ORF1a-SNS2022del
AAATACCTGGTGTATACGTTGTCTTTGGAGCACAAAACCAGTTGAAACAT
caa att cgt
TTGATGTACTGAAGTCAGAGGACGCGCAGGGAATGGATAATCTTGCCTGCG

29762 29 1 0 7
AAAGAGCCACCACATTTTCACCGAGGCCACGCGGAGTACGATCGAGTGTA
cag tga aca atg cta ggg aga gct gcc ta
TATGGAAGAGCCCTAATGTGTAAAATTAATTTTAGTAGTGCTATCCCCATG

26155 7 1 0 7 ORF3a-VVN255fs
ATGAGCCTGAAGAACATGTCCAAATTCACACAATCGACGGTTCATCCGGA
gtt gtt a
ATCCAGTAATGGAACCAATTTATGATGAACCGACGACGACTACTAGCGTGC

27580 20 1 0 7 ORF7a-FAFACPD63fs
TTCATCCTCTAGCTGATAACAAATTTGCACTGACTTGCTTTAGCACTCAA
ttt gct ttt gct tgt cct ga
CGGCGTAAAACACGTCTATCAGTTACGTGCCAGATCAGTTTCACCTAAACT

3342 9 6 0 6 ORF1a-NSF1026del
ACCTCAATTAGAGATGGAACTTACACCAGTTGTTCAGACTATTGAAGTGA
ata gtt tta
GTGGTTATTTAAAACTTACTGACAATGTATACATTAAAAATGCAGACATTG

29736 26 6 0 6
CAGTGTGTAACATTAGGGAGGACTTGAAAGAGCCACCACATTTTCACCGA
ggc cac gcg gag tac gat cga gtg ta
CAGTGAACAATGCTAGGGAGAGCTGCCTATATGGAAGAGCCCTAATGTGTA

28147 7 5 0 6 ORF8-PFT85fs
ATCACCCATTCAGTACATCGATATCGGTAATTATACAGTTTCCTGTTTAC
ctt tta c
AATTAATTGCCAGGAACCTAAATTGGGTAGTCTTGTAGTGCGTTGTTCGTT

26161 21 4 0 6 ORF3a-NPVMEPI257del
CTGAAGAACATGTCCAAATTCACACAATCGACGGTTCATCCGGAGTTGTT
aat cca gta atg gaa cca att
TATGATGAACCGACGACGACTACTAGCGTGCCTTTGTAAGCACAAGCTGAT

29749 13 3 0 6
TAGGGAGGACTTGAAAGAGCCACCACATTTTCACCGAGGCCACGCGGAGT
acg atc gag tgt a
CAGTGAACAATGCTAGGGAGAGCTGCCTATATGGAAGAGCCCTAATGTGTA

27599 26 3 0 6 ORF7a-DGVKHVYQL69fs
CAAATTTGCACTGACTTGCTTTAGCACTCAATTTGCTTTTGCTTGTCCTG
acg gcg taa aac acg tct atc agt ta
CGTGCCAGATCAGTTTCACCTAAACTGTTCATCAGACAAGAGGAAGTTCAA

25432 9 3 0 6 ORF3a-TLK14del
TAAACGAACTTATGGATTTGTTTATGAGAATCTTCACAATTGGAACTGTA
act ttg aag
CAAGGTGAAATCAAGGATGCTACTCCTTCAGATTTTGTTCGCGCTACTGCA

29730 22 3 0 6
TTTAATCAGTGTGTAACATTAGGGAGGACTTGAAAGAGCCACCACATTTT
cac cga ggc cac gcg gag tac g
ATCGAGTGTACAGTGAACAATGCTAGGGAGAGCTGCCTATATGGAAGAGCC

29732 21 3 0 6
TAATCAGTGTGTAACATTAGGGAGGACTTGAAAGAGCCACCACATTTTCA
ccg agg cca cgc gga gta cga
TCGAGTGTACAGTGAACAATGCTAGGGAGAGCTGCCTATATGGAAGAGCCC

27686 13 3 0 6 ORF7a-SPIFL98fs
AGTTTCACCTAAACTGTTCATCAGACAAGAGGAAGTTCAAGAACTTTACT
ctc caa ttt ttc t
TATTGTTGCGGCAATAGTGTTTATAACACTTTGCTTCACACTCAAAAGAAA

27903 7 3 0 6 ORF8-LVF4fs
AACTGCAAGATCATAATGAAACTTGTCACGCCTAAACGAACATGAAATTT
ctt gtt t
TCTTAGGAATCATCACAACTGTAGCTGCATTTCACCAAGAATGTAGTTTAC

3937 9 3 0 6 ORF1a-RKQ1225del
AAAGAGGAAGTTAAGCCATTTATAACTGAAAGTAAACCTTCAGTTGAACA
gag aaa aca
AGATGATAAGAAAATCAAAGCTTGTGTTGAAGAAGTTACAACAACTCTGGA

27715      12      3      0      6 ORF7a-VFIT108del
AGGAAGTTCAAGAACTTTACTCTCCAATTTTTCTTATTGTTGCGGCAATA
gtg ttt ata aca
CTTTGCTTCACACTCAAAAGAAAGACAGAATGATTGAACTTTCATTAATTG

29753      15      3      0      6
GAGGACTTGAAAGAGCCACCACATTTTCACCGAGGCCACGCGGAGTACGA
tcg agt gta cag tga
ACAATGCTAGGGAGAGCTGCCTATATGGAAGAGCCCTAATGTGTAAAATTA

27702      21      3      0      6 ORF7a-VAAIVFI104del
TTCATCAGACAAGAGGAAGTTCAAGAACTTTACTCTCCAATTTTTCTTAT
tgt tgc ggc aat agt gtt tat
AACACTTTGCTTCACACTCAAAAGAAAGACAGAATGATTGAACTTTCATTA

29731      7      2      0      6
TTAATCAGTGTGTAACATTAGGGAGGACTTGAAAGAGCCACCACATTTTC
acc gag g
CCACGCGGAGTACGATCGAGTGTACAGTGAACAATGCTAGGGAGAGCTGCC

27684      22      2      0      6 ORF7a-YSPIFLIV97fs
TCAGTTTCACCTAAACTGTTCATCAGACAAGAGGAAGTTCAAGAACTTTA
ctc tcc aat ttt tct tat tgt t
GCGGCAATAGTGTTTATAACACTTTGCTTCACACTCAAAAGAAAGACAGAA

27596      24      2      0      6 ORF7a-PDGVKHVY68del
TAACAAATTTGCACTGACTTGCTTTAGCACTCAATTTGCTTTTGCTTGTC
ctg acg gcg taa aac acg tct atc
AGTTACGTGCCAGATCAGTTTCACCTAAACTGTTCATCAGACAAGAGGAAG

29747      13      2      0      6
ATTAGGGAGGACTTGAAAGAGCCACCACATTTTCACCGAGGCCACGCGGA
gta cga tcg agt g
TACAGTGAACAATGCTAGGGAGAGCTGCCTATATGGAAGAGCCCTAATGTG

28240      7      2      0      6 ORF8-VVL116fs
AGTGCGTTGTTCGTTCTATGAAGACTTTTTAGAGTATCATGACGTTCGTG
ttg ttt t
AGATTTCATCTAAACGAACAAACTAAAATGTCTGATAATGGACCCCAAAAT

21982      15      2      0      6 S-LGVYY141del
ACTAATGTTGTTATTAAAGTCTGTGAATTTCAATTTTGTAATGATCCATT
ttt ggg tgt tta tta
CCACAAAAACAACAAAAGTTGGATGGAAAGTGAGTTCAGAGTTTATTCTAG

27606      9      1      0      6 ORF7a-KHV72del
GCACTGACTTGCTTTAGCACTCAATTTGCTTTTGCTTGTCCTGACGGCGT
aaa aca cgt
CTATCAGTTACGTGCCAGATCAGTTTCACCTAAACTGTTCATCAGACAAGA

27972      25      1      0      6 ORF8-QHQPYVVDD27fs
TCACAACTGTAGCTGCATTTCACCAAGAATGTAGTTTACAGTCATGTACT
caa cat caa cca tat gta gtt gat g

ACCCGTGTCCTATTCACTTCTATTCTAAATGGTATATTAGAGTAGGAGCTA

27673 21 1 0 6 ORF7a-QELYSPI94del
TACGTGCCAGATCAGTTTCACCTAAACTGTTCATCAGACAAGAGGAAGTT
caa gaa ctt tac tct cca att
TTTCTTATTGTTGCGGCAATAGTGTTTATAACACTTTGCTTCACACTCAAA

27577 29 1 0 6 ORF7a-QFAFACPDGV62fs
CATTTCATCCTCTAGCTGATAACAAATTTGCACTGACTTGCTTTAGCACT
caa ttt gct ttt gct tgt cct gac ggc gt
AAAACACGTCTATCAGTTACGTGCCAGATCAGTTTCACCTAAACTGTTCAT

29733 13 1 0 6
AATCAGTGTGTAACATTAGGGAGGACTTGAAAGAGCCACCACATTTTCAC
cga ggc cac gcg g
AGTACGATCGAGTGTACAGTGAACAATGCTAGGGAGAGCTGCCTATATGGA

22028 12 1 0 6 S-EFRV156del
CATTTTTGGGTGTTTATTACCACAAAAACAACAAAAGTTGGATGGAAAGT
gag ttc aga gtt
TATTCTAGTGCGAATAATTGCACTTTTGAATATGTCTCTCAGCCTTTTCTT

21761 27 1 0 6 S-AIHVSGTNG67del
CAACTCAGGACTTGTTCTTACCTTTCTTTTCCAATGTTACTTGGTTCCAT
gct ata cat gtc tct ggg acc aat ggt
ACTAAGAGGTTTGATAACCCTGTCCTACCATTTAATGATGGTGTTTATTTT

27583 12 1 0 6 ORF7a-AFAC64del
ATCCTCTAGCTGATAACAAATTTGCACTGACTTGCTTTAGCACTCAATTT
gct ttt gct tgt
CCTGACGGCGTAAAACACGTCTATCAGTTACGTGCCAGATCAGTTTCACCT

27267 22 1 0 6 ORF6-FKVSIWNL22fs
GACTTTCAGGTTACTATAGCAGAGATATTACTAATTATTATGAGGACTTT
taa agt ttc cat ttg gaa tct t
GATTACATCATAAACCTCATAATTAAAAATTTATCTAAGTCACTAACTGAG

517 7 1 0 6 ORF1a-VMV84fs
TATGTGTTCATCAAACGTTCGGATGCTCGAACTGCACCTCATGGTCATGT
tat ggt t
GAGCTGGTAGCAGAACTCGAAGGCATTCAGTACGGTCGTAGTGGTGAGACA

27607 11 5 0 5 ORF7a-KHVY72fs
CACTGACTTGCTTTAGCACTCAATTTGCTTTTGCTTGTCCTGACGGCGTA
aaa cac gtc ta
TCAGTTACGTGCCAGATCAGTTTCACCTAAACTGTTCATCAGACAAGAGGA

21974 18 5 0 5 S-DPFLGV138del
ATAACGCTACTAATGTTGTTATTAAAGTCTGTGAATTTCAATTTTGTAAT
gat cca ttt ttg ggt gtt
TATTACCACAAAAACAACAAAAGTTGGATGGAAAGTGAGTTCAGAGTTTAT

29754 12 4 0 5
AGGACTTGAAAGAGCCACCACATTTTCACCGAGGCCACGCGGAGTACGAT

cga gtg tac agt
GAACAATGCTAGGGAGAGCTGCCTATATGGAAGAGCCCTAATGTGTAAAAT

431 30 4 0 5 ORF1a-VEKGVLPQLE56del
TATCAGAGGCACGTCAACATCTTAAAGATGGCACTTGTGGCTTAGTAGAA
gtt gaa aaa ggc gtt ttg cct caa ctt gaa
CAGCCCTATGTGTTCATCAAACGTTCGGATGCTCGAACTGCACCTCATGGT

6513 12 4 0 5 ORF1a-SLKI2083del
GAAAACTACCGAAGTTGTAGGAGACATTATACTTAAACCAGCAAATAATA
gtt taa aaa tta
CAGAAGAGGTTGGCCACACAGATCTAATGGCTGCTTATGTAGACAATTCTA

27683 13 3 0 5 ORF7a-YSPIF97fs
ATCAGTTTCACCTAAACTGTTCATCAGACAAGAGGAAGTTCAAGAACTTT
act ctc caa ttt t
TCTTATTGTTGCGGCAATAGTGTTTATAACACTTTGCTTCACACTCAAAAG

21602 15 3 0 5 S-QCVNL14del
CTAAACGAACAATGTTTGTTTTTCTTGTTTTATTGCCACTAGTCTCTAGT
cag tgt gtt aat ctt
ACAACCAGAACTCAATTACCCCCTGCATACACTAATTCTTTCACACGTGGT

506 12 3 0 5 ORF1a-HGHV81del
TTGAACAGCCCTATGTGTTCATCAAACGTTCGGATGCTCGAACTGCACCT
cat ggt cat gtt
ATGGTTGAGCTGGTAGCAGAACTCGAAGGCATTCAGTACGGTCGTAGTGGT

29738 30 3 0 5
GTGTGTAACATTAGGGAGGACTTGAAAGAGCCACCACATTTTCACCGAGG
cca cgc gga gta cga tcg agt gta cag tga
ACAATGCTAGGGAGAGCTGCCTATATGGAAGAGCCCTAATGTGTAAAATTA

29750 18 3 0 5
AGGGAGGACTTGAAAGAGCCACCACATTTTCACCGAGGCCACGCGGAGTA
cga tcg agt gta cag tga
ACAATGCTAGGGAGAGCTGCCTATATGGAAGAGCCCTAATGTGTAAAATTA

27762 12 3 0 5 ORF7b-ELSL3del
ATAGTGTTTATAACACTTTGCTTCACACTCAAAAGAAAGACAGAATGATT
gaa ctt tca tta
ATTGACTTCTATTTGTGCTTTTTAGCCTTTCTGCTATTCCTTGTTTTAATT

28248 7 3 0 5 ORF8-DFI119fs
GTTCGTTCTATGAAGACTTTTTAGAGTATCATGACGTTCGTGTTGTTTTA
gat ttc a
TCTAAACGAACAAACTAAAATGTCTGATAATGGACCCCAAAATCAGCGAAA

27572 10 3 0 5 ORF7a-STQF60fs
TTCACCATTTCATCCTCTAGCTGATAACAAATTTGCACTGACTTGCTTTA
gca ctc aat t
TGCTTTTGCTTGTCCTGACGGCGTAAAACACGTCTATCAGTTACGTGCCAG

28242 11 3 0 5 ORF8-VLDF117fs

TGCGTTGTTCGTTCTATGAAGACTTTTTAGAGTATCATGACGTTCGTGTT
gtt tta gat tt
CATCTAAACGAACAAACTAAAATGTCTGATAATGGACCCCAAAATCAGCGA

28242 17 3 0 5 ORF8-VLDFI*117fs
TGCGTTGTTCGTTCTATGAAGACTTTTTAGAGTATCATGACGTTCGTGTT
gtt tta gat ttc atc ta
AACGAACAAACTAAAATGTCTGATAATGGACCCCAAAATCAGCGAAATGCA

27906 8 3 0 5 ORF8-VFL5fs
TGCAAGATCATAATGAAACTTGTCACGCCTAAACGAACATGAAATTTCTT
gtt ttc tt
AGGAATCATCACAACTGTAGCTGCATTTCACCAAGAATGTAGTTTACAGTC

11271 9 3 0 5 ORF1a-MVD3669del
GGTCTATATGCCTGCTAGTTGGGTGATGCGTATTATGACATGGTTGGATA
tgg ttg ata
CTAGTTTGTCTGGTTTTAAGCTAAAAGACTGTGTTATGTATGCATCAGCTG

28245 14 3 0 5 ORF8-LDFI*118fs
GTTGTTCGTTCTATGAAGACTTTTTAGAGTATCATGACGTTCGTGTTGTT
tta gat ttc atc ta
AACGAACAAACTAAAATGTCTGATAATGGACCCCAAAATCAGCGAAATGCA

27694 12 3 0 5 ORF7a-FLIV101del
CTAAACTGTTCATCAGACAAGAGGAAGTTCAAGAACTTTACTCTCCAATT
ttt ctt att gtt
GCGGCAATAGTGTTTATAACACTTTGCTTCACACTCAAAAGAAAGACAGAA

196 15 2 0 5
ACTGTCGTTGACAGGACACGAGTAACTCGTCTATCTTCTGCAGGCTGCTT
acg gtt tcg tcc gtg
TTGCAGCCGATCATCAGCACATCTAGGTTTCGTCCGGGTGTGACCGAAAGG

27549 31 2 0 5 ORF7a-NKFALTCFSTQ52fs
TCTTCTGGAACATACGAGGGCAATTCACCATTTCATCCTCTAGCTGATAA
caa att tgc act gac ttg ctt tag cac tca a
TTTGCTTTTGCTTGTCCTGACGGCGTAAAACACGTCTATCAGTTACGTGCC

27448 14 2 0 5 ORF7a-HYQEC19fs
AAATTATTCTTTTCTTGGCACTGATAACACTCGCTACTTGTGAGCTTTAT
cac tac caa gag tg
TGTTAGAGGTACAACAGTACTTTTAAAAGAACCTTGCTCTTCTGGAACATA

25499 15 2 0 5 ORF3a-PIQAS36del
AATCAAGGATGCTACTCCTTCAGATTTTGTTCGCGCTACTGCAACGATAC
cga tac aag cct cac
TCCCTTTCGGATGGCTTATTGTTGGCGTTGCACTTCTTGCTGTTTTTCAGA

27427 17 2 0 5 ORF7a-LATCEL12fs
AGATTGATTAAACGAACATGAAAATTATTCTTTTCTTGGCACTGATAACA
ctc gct act tgt gag ct
TTATCACTACCAAGAGTGTGTTAGAGGTACAACAGTACTTTTAAAAGAACC

29751 19 2 0 5
GGGAGGACTTGAAAGAGCCACCACATTTTCACCGAGGCCACGCGGAGTAC
gat cga gtg tac agt gaa c
AATGCTAGGGAGAGCTGCCTATATGGAAGAGCCCTAATGTGTAAAATTAAT

28080 16 2 0 5 ORF8-DEAGSK63fs
ATATTAGAGTAGGAGCTAGAAAATCAGCACCTTTAATTGAATTGTGCGTG
gat gag gct ggt tct a
AATCACCCATTCAGTACATCGATATCGGTAATTATACAGTTTCCTGTTTAC

28089 8 2 0 5 ORF8-GSK66fs
TAGGAGCTAGAAAATCAGCACCTTTAATTGAATTGTGCGTGGATGAGGCT
ggt tct aa
ATCACCCATTCAGTACATCGATATCGGTAATTATACAGTTTCCTGTTTACC

27757 8 2 0 5 ORF7a-*122del
CGGCAATAGTGTTTATAACACTTTGCTTCACACTCAAAAGAAAGACAGAA
tga ttg aa
CTTTCATTAATTGACTTCTATTTGTGCTTTTTAGCCTTTCTGCTATTCCTT

27757 8 2 0 5 ORF7b-MIE1fs
CGGCAATAGTGTTTATAACACTTTGCTTCACACTCAAAAGAAAGACAGAA
tga ttg aa
CTTTCATTAATTGACTTCTATTTGTGCTTTTTAGCCTTTCTGCTATTCCTT

27581 8 2 0 5 ORF7a-FAF63fs
TCATCCTCTAGCTGATAACAAATTTGCACTGACTTGCTTTAGCACTCAAT
ttg ctt tt
GCTTGTCCTGACGGCGTAAAACACGTCTATCAGTTACGTGCCAGATCAGTT

29749 24 1 0 5
TAGGGAGGACTTGAAAGAGCCACCACATTTTCACCGAGGCCACGCGGAGT
acg atc gag tgt aca gtg aac aat
GCTAGGGAGAGCTGCCTATATGGAAGAGCCCTAATGTGTAAAATTAATTTT

29740 14 1 0 5
GTGTAACATTAGGGAGGACTTGAAAGAGCCACCACATTTTCACCGAGGCC
acg cgg agt acg at
CGAGTGTACAGTGAACAATGCTAGGGAGAGCTGCCTATATGGAAGAGCCCT

27532 24 1 0 5 ORF7a-HPLADNKF47del
TTTTAAAAGAACCTTGCTCTTCTGGAACATACGAGGGCAATTCACCATTT
cat cct cta gct gat aac aaa ttt
GCACTGACTTGCTTTAGCACTCAATTTGCTTTTGCTTGTCCTGACGGCGTA

3092 24 1 0 5 ORF1a-PSTQYEYG943del
CTCCAGATGAGGATGAAGAAGAAGGTGATTGTGAAGAAGAAGAGTTTGAG
cca tca act caa tat gag tat ggt
ACTGAAGATGATTACCAAGGTAAACCTTTGGAATTTGGTGCCACTTCTGCT

29750 22 1 0 5
AGGGAGGACTTGAAAGAGCCACCACATTTTCACCGAGGCCACGCGGAGTA
cga tcg agt gta cag tga aca a
TGCTAGGGAGAGCTGCCTATATGGAAGAGCCCTAATGTGTAAAATTAATTT

3330 15 1 0 5 ORF1a-TIEVN1022del
TGTTGAGGTTCAACCTCAATTAGAGATGGAACTTACACCAGTTGTTCAGA
cta ttg aag tga ata
GTTTTAGTGGTTATTTAAAACTTACTGACAATGTATACATTAAAAATGCAG

510 8 1 0 5 ORF1a-GHV82fs
ACAGCCCTATGTGTTCATCAAACGTTCGGATGCTCGAACTGCACCTCATG
gtc atg tt
ATGGTTGAGCTGGTAGCAGAACTCGAAGGCATTCAGTACGGTCGTAGTGGT

28144 14 1 0 5 ORF8-LPFTI84fs
TAAATCACCCATTCAGTACATCGATATCGGTAATTATACAGTTTCCTGTT
tac ctt tta caa tt
AATTGCCAGGAACCTAAATTGGGTAGTCTTGTAGTGCGTTGTTCGTTCTAT

27384 14 1 0 5 ORF6-D*61fs
ACTGAGAATAAATATTCTCAATTAGATGAAGAGCAACCAATGGAGATTGA
tta aac gaa cat ga
AAATTATTCTTTTCTTGGCACTGATAACACTCGCTACTTGTGAGCTTTATC

27384 14 1 0 5 ORF7a-MK1fs
ACTGAGAATAAATATTCTCAATTAGATGAAGAGCAACCAATGGAGATTGA
tta aac gaa cat ga
AAATTATTCTTTTCTTGGCACTGATAACACTCGCTACTTGTGAGCTTTATC

27680 9 1 0 5 ORF7a-LYS96del
CAGATCAGTTTCACCTAAACTGTTCATCAGACAAGAGGAAGTTCAAGAAC
ttt act ctc
CAATTTTTCTTATTGTTGCGGCAATAGTGTTTATAACACTTTGCTTCACAC

25512 9 4 0 4 ORF3a-LPF41del
ACTCCTTCAGATTTTGTTCGCGCTACTGCAACGATACCGATACAAGCCTC
act ccc ttt
CGGATGGCTTATTGTTGGCGTTGCACTTCTTGCTGTTTTTCAGAGCGCTTC

21975 9 4 0 4 S-DPFL138V
TAACGCTACTAATGTTGTTATTAAAGTCTGTGAATTTCAATTTTGTAATG
atc cat ttt
TGGGTGTTTATTACCACAAAAACAACAAAAGTTGGATGGAAAGTGAGTTCA

3881 21 4 0 4 ORF1a-IPKEEVK1206del
GCTTTTTGGAAATGAAGAGTGAAAAGCAAGTTGAACAAAAGATCGCTGAG
att cct aaa gag gaa gtt aag
CCATTTATAACTGAAAGTAAACCTTCAGTTGAACAGAGAAAACAAGATGAT

27289 12 4 0 4 ORF6-DYII30del
AGATATTACTAATTATTATGAGGACTTTTAAAGTTTCCATTTGGAATCTT
gat tac atc ata
AACCTCATAATTAAAAATTTATCTAAGTCACTAACTGAGAATAAATATTCT

29737 30 4 0 4
AGTGTGTAACATTAGGGAGGACTTGAAAGAGCCACCACATTTTCACCGAG
gcc acg cgg agt acg atc gag tgt aca gtg

AACAATGCTAGGGAGAGCTGCCTATATGGAAGAGCCCTAATGTGTAAAATT

510 12 4 0 4 ORF1a-GHVM82del
ACAGCCCTATGTGTTCATCAAACGTTCGGATGCTCGAACTGCACCTCATG
gtc atg tta tgg
TTGAGCTGGTAGCAGAACTCGAAGGCATTCAGTACGGTCGTAGTGGTGAGA

29760 9 4 0 4
TGAAAGAGCCACCACATTTTCACCGAGGCCACGCGGAGTACGATCGAGTG
tac agt gaa
CAATGCTAGGGAGAGCTGCCTATATGGAAGAGCCCTAATGTGTAAAATTAA

21765 27 4 0 4 S-IHVSGTNGT68del
TCAGGACTTGTTCTTACCTTTCTTTTCCAATGTTACTTGGTTCCATGCTA
tac atg tct ctg gga cca atg gta cta
AGAGGTTTGATAACCCTGTCCTACCATTTAATGATGGTGTTTATTTTGCTT

28407 9 4 0 4 N-LPNN45Y
GAATGGAGAACGCAGTGGGGCGCGATCAAAACAACGTCGGCCCCAAGGTT
tac cca ata
ATACTGCGTCTTGGTTCACCGCTCTCACTCAACATGGCAAGGAAGACCTTA

28150 9 4 0 4 ORF8-FTIN86Y
ACCCATTCAGTACATCGATATCGGTAATTATACAGTTTCCTGTTTACCTT
tta caa tta
ATTGCCAGGAACCTAAATTGGGTAGTCTTGTAGTGCGTTGTTCGTTCTATG

25425 21 3 0 4 ORF3a-TVTLKQG12del
TTACACATAAACGAACTTATGGATTTGTTTATGAGAATCTTCACAATTGG
aac tgt aac ttt gaa gca agg
TGAAATCAAGGATGCTACTCCTTCAGATTTTGTTCGCGCTACTGCAACGAT

25425 27 3 0 4 ORF3a-TVTLKQGEI12del
TTACACATAAACGAACTTATGGATTTGTTTATGAGAATCTTCACAATTGG
aac tgt aac ttt gaa gca agg tga aat
CAAGGATGCTACTCCTTCAGATTTTGTTCGCGCTACTGCAACGATACCGAT

27674 21 3 0 4 ORF7a-QELYSPIF94L
ACGTGCCAGATCAGTTTCACCTAAACTGTTCATCAGACAAGAGGAAGTTC
aag aac ttt act ctc caa ttt
TTCTTATTGTTGCGGCAATAGTGTTTATAACACTTTGCTTCACACTCAAAA

29723 11 3 0 4
AGCAATCTTTAATCAGTGTGTAACATTAGGGAGGACTTGAAAGAGCCACC
aca ttt tca cc
GAGGCCACGCGGAGTACGATCGAGTGTACAGTGAACAATGCTAGGGAGAGC

27525 18 3 0 4 ORF7a-PFHPLA45del
ACAGTACTTTTAAAAGAACCTTGCTCTTCTGGAACATACGAGGGCAATTC
acc att tca tcc tct agc
TGATAACAAATTTGCACTGACTTGCTTTAGCACTCAATTTGCTTTTGCTTG

27599 19 3 0 4 ORF7a-DGVKHVY69fs
CAAATTTGCACTGACTTGCTTTAGCACTCAATTTGCTTTTGCTTGTCCTG

acg gcg taa aac acg tct a
TCAGTTACGTGCCAGATCAGTTTCACCTAAACTGTTCATCAGACAAGAGGA

27571 10 3 0 4 ORF7a-STQF60fs
ATTCACCATTTCATCCTCTAGCTGATAACAAATTTGCACTGACTTGCTTT
agc act caa t
TTGCTTTTGCTTGTCCTGACGGCGTAAAACACGTCTATCAGTTACGTGCCA

6375 9 3 0 4 ORF1a-DNL2037del
AACATCAAATTCGTTTGATGTACTGAAGTCAGAGGACGCGCAGGGAATGG
ata atc ttg
CCTGCGAAGATCTAAAACCAGTCTCTGAAGAAGTAGTGGAAAATCCTACCA

29752 7 3 0 4
GGAGGACTTGAAAGAGCCACCACATTTTCACCGAGGCCACGCGGAGTACG
atc gag t
GTACAGTGAACAATGCTAGGGAGAGCTGCCTATATGGAAGAGCCCTAATGT

27579 9 3 0 4 ORF7a-QFAF62H
TTTCATCCTCTAGCTGATAACAAATTTGCACTGACTTGCTTTAGCACTCA
att tgc ttt
TGCTTGTCCTGACGGCGTAAAACACGTCTATCAGTTACGTGCCAGATCAGT

29441 9 3 0 4 N-QTV390del
AGAAGAAGGCTGATGAAACTCAAGCCTTACCGCAGAGACAGAAGAAACAG
caa act gtg
ACTCTTCTTCCTGCTGCAGATTTGGATGATTTCTCCAAACAATTGCAACAA

29747 15 3 0 4
ATTAGGGAGGACTTGAAAGAGCCACCACATTTTCACCGAGGCCACGCGGA
gta cga tcg agt gta
CAGTGAACAATGCTAGGGAGAGCTGCCTATATGGAAGAGCCCTAATGTGTA

510 13 3 0 4 ORF1a-GHVMV82fs
ACAGCCCTATGTGTTCATCAAACGTTCGGATGCTCGAACTGCACCTCATG
gtc atg tta tgg t
TGAGCTGGTAGCAGAACTCGAAGGCATTCAGTACGGTCGTAGTGGTGAGAC

27788 8 3 0 4 ORF7b-LCF11fs
ACTCAAAAGAAAGACAGAATGATTGAACTTTCATTAATTGACTTCTATTT
gtg ctt tt
TAGCCTTTCTGCTATTCCTTGTTTTAATTATGCTTATTATCTTTTGGTTCT

27796 8 3 0 4 ORF7b-LAF14fs
GAAAGACAGAATGATTGAACTTTCATTAATTGACTTCTATTTGTGCTTTT
tag cct tt
CTGCTATTCCTTGTTTTAATTATGCTTATTATCTTTTGGTTCTCACTTGAA

29729 32 3 0 4
CTTTAATCAGTGTGTAACATTAGGGAGGACTTGAAAGAGCCACCACATTT
tca ccg agg cca cgc gga gta cga tcg agt gt
ACAGTGAACAATGCTAGGGAGAGCTGCCTATATGGAAGAGCCCTAATGTGT

27795 9 3 0 4 ORF7b-AFL15del

AGAAAGACAGAATGATTGAACTTTCATTAATTGACTTCTATTTGTGCTTT
tta gcc ttt
CTGCTATTCCTTGTTTTAATTATGCTTATTATCTTTTGGTTCTCACTTGAA

27608 17 2 0 4 ORF7a-KHVYQL72fs
ACTGACTTGCTTTAGCACTCAATTTGCTTTTGCTTGTCCTGACGGCGTAA
aac acg tct atc agt ta
CGTGCCAGATCAGTTTCACCTAAACTGTTCATCAGACAAGAGGAAGTTCAA

29731 17 2 0 4
TTAATCAGTGTGTAACATTAGGGAGGACTTGAAAGAGCCACCACATTTTC
acc gag gcc acg cgg ag
TACGATCGAGTGTACAGTGAACAATGCTAGGGAGAGCTGCCTATATGGAAG

29741 11 2 0 4
TGTAACATTAGGGAGGACTTGAAAGAGCCACCACATTTTCACCGAGGCCA
cgc gga gta cg
ATCGAGTGTACAGTGAACAATGCTAGGGAGAGCTGCCTATATGGAAGAGCC

27625 9 2 0 4 ORF7a-RAR78del
CTCAATTTGCTTTTGCTTGTCCTGACGGCGTAAAACACGTCTATCAGTTA
cgt gcc aga
TCAGTTTCACCTAAACTGTTCATCAGACAAGAGGAAGTTCAAGAACTTTAC

29755 7 2 0 4
GGACTTGAAAGAGCCACCACATTTTCACCGAGGCCACGCGGAGTACGATC
gag tgt a
CAGTGAACAATGCTAGGGAGAGCTGCCTATATGGAAGAGCCCTAATGTGTA

27556 23 2 0 4 ORF7a-ALTCFSTQ55fs
GAACATACGAGGGCAATTCACCATTTCATCCTCTAGCTGATAACAAATTT
gca ctg act tgc ttt agc act ca
ATTTGCTTTTGCTTGTCCTGACGGCGTAAAACACGTCTATCAGTTACGTGC

27556 32 2 0 4 ORF7a-ALTCFSTQFAF55fs
GAACATACGAGGGCAATTCACCATTTCATCCTCTAGCTGATAACAAATTT
gca ctg act tgc ttt agc act caa ttt gct tt
TGCTTGTCCTGACGGCGTAAAACACGTCTATCAGTTACGTGCCAGATCAGT

29742 21 2 0 4
GTAACATTAGGGAGGACTTGAAAGAGCCACCACATTTTCACCGAGGCCAC
gcg gag tac gat cga gtg tac
AGTGAACAATGCTAGGGAGAGCTGCCTATATGGAAGAGCCCTAATGTGTAA

25423 30 2 0 4 ORF3a-GTVTLKQGEI11del
CATTACACATAAACGAACTTATGGATTTGTTTATGAGAATCTTCACAATT
gga act gta act ttg aag caa ggt gaa atc
AAGGATGCTACTCCTTCAGATTTTGTTCGCGCTACTGCAACGATACCGATA

27682 21 2 0 4 ORF7a-YSPIFLI97del
GATCAGTTTCACCTAAACTGTTCATCAGACAAGAGGAAGTTCAAGAACTT
tac tct cca att ttt ctt att
GTTGCGGCAATAGTGTTTATAACACTTTGCTTCACACTCAAAAGAAAGACA

28243 11 2 0 4 ORF8-VLDF117fs
GCGTTGTTCGTTCTATGAAGACTTTTTAGAGTATCATGACGTTCGTGTTG
ttt tag att tc
ATCTAAACGAACAAACTAAAATGTCTGATAATGGACCCCAAAATCAGCGAA

27574 9 1 0 4 ORF7a-TQF61del
CACCATTTCATCCTCTAGCTGATAACAAATTTGCACTGACTTGCTTTAGC
act caa ttt
GCTTTTGCTTGTCCTGACGGCGTAAAACACGTCTATCAGTTACGTGCCAGA

29746 14 1 0 4
CATTAGGGAGGACTTGAAAGAGCCACCACATTTTCACCGAGGCCACGCGG
agt acg atc gag tg
TACAGTGAACAATGCTAGGGAGAGCTGCCTATATGGAAGAGCCCTAATGTG

3305 21 1 0 4 ORF1a-MELTPVV1014del
ATCAGACAACTACTATTCAAACAATTGTTGAGGTTCAACCTCAATTAGAG
atg gaa ctt aca cca gtt gtt
CAGACTATTGAAGTGAATAGTTTTAGTGGTTATTTAAAACTTACTGACAAT

27610 28 1 0 4 ORF7a-HVYQLRARSV73fs
TGACTTGCTTTAGCACTCAATTTGCTTTTGCTTGTCCTGACGGCGTAAAA
cac gtc tat cag tta cgt gcc aga tca g
TTTCACCTAAACTGTTCATCAGACAAGAGGAAGTTCAAGAACTTTACTCTC

27393 9 1 0 4 ORF7a-MKI1fs
AAATATTCTCAATTAGATGAAGAGCAACCAATGGAGATTGATTAAACGAA
cat gaa aat
TATTCTTTTCTTGGCACTGATAACACTCGCTACTTGTGAGCTTTATCACTA

29754 13 1 0 4
AGGACTTGAAAGAGCCACCACATTTTCACCGAGGCCACGCGGAGTACGAT
cga gtg tac agt g
AACAATGCTAGGGAGAGCTGCCTATATGGAAGAGCCCTAATGTGTAAAATT

27566 16 1 0 4 ORF7a-CFSTQF58fs
GGGCAATTCACCATTTCATCCTCTAGCTGATAACAAATTTGCACTGACTT
gct tta gca ctc aat t
TGCTTTTGCTTGTCCTGACGGCGTAAAACACGTCTATCAGTTACGTGCCAG

3883 12 1 0 4 ORF1a-PKEE1207del
TTTTTGGAAATGAAGAGTGAAAAGCAAGTTGAACAAAAGATCGCTGAGAT
tcc taa aga gga
AGTTAAGCCATTTATAACTGAAAGTAAACCTTCAGTTGAACAGAGAAAACA

28891 12 1 0 4 N-PARM207del
AGTCGCAACAGTTCAAGAAATTCAACTCCAGGCAGCAGTAGGGGAACTTC
tcc tgc tag aat
GGCTGGCAATGGCGGTGATGCTGCTCTTGCTTTGCTGCTGCTTGACAGATT

27405 15 1 0 4 ORF7a-ILFLAL4M
TTAGATGAAGAGCAACCAATGGAGATTGATTAAACGAACATGAAAATTAT
tct ttt ctt ggc act
GATAACACTCGCTACTTGTGAGCTTTATCACTACCAAGAGTGTGTTAGAGG

27530 12 1 0 4 ORF7a-FHPLA46S
ACTTTTAAAAGAACCTTGCTCTTCTGGAACATACGAGGGCAATTCACCAT
ttc atc ctc tag
CTGATAACAAATTTGCACTGACTTGCTTTAGCACTCAATTTGCTTTTGCTT

27287 13 1 0 4 ORF6-LDYII29fs
AGAGATATTACTAATTATTATGAGGACTTTTAAAGTTTCCATTTGGAATC
ttg att aca tca t
AAACCTCATAATTAAAAATTTATCTAAGTCACTAACTGAGAATAAATATTC

435 12 3 0 3 ORF1a-EKGVL57V
AGAGGCACGTCAACATCTTAAAGATGGCACTTGTGGCTTAGTAGAAGTTG
aaa aag gcg ttt
TGCCTCAACTTGAACAGCCCTATGTGTTCATCAAACGTTCGGATGCTCGAA

27607 18 3 0 3 ORF7a-KHVYQL72del
CACTGACTTGCTTTAGCACTCAATTTGCTTTTGCTTGTCCTGACGGCGTA
aaa cac gtc tat cag tta
CGTGCCAGATCAGTTTCACCTAAACTGTTCATCAGACAAGAGGAAGTTCAA

27897 10 3 0 3 ORF8-KFLV2fs
CACTTGAACTGCAAGATCATAATGAAACTTGTCACGCCTAAACGAACATG
aaa ttt ctt g
TTTTCTTAGGAATCATCACAACTGTAGCTGCATTTCACCAAGAATGTAGTT

29731 13 3 0 3
TTAATCAGTGTGTAACATTAGGGAGGACTTGAAAGAGCCACCACATTTTC
acc gag gcc acg c
GGAGTACGATCGAGTGTACAGTGAACAATGCTAGGGAGAGCTGCCTATATG

27624 17 3 0 3 ORF7a-LRARSV77fs
ACTCAATTTGCTTTTGCTTGTCCTGACGGCGTAAAACACGTCTATCAGTT
acg tgc cag atc agt tt
CACCTAAACTGTTCATCAGACAAGAGGAAGTTCAAGAACTTTACTCTCCAA

27673 9 3 0 3 ORF7a-QEL94del
TACGTGCCAGATCAGTTTCACCTAAACTGTTCATCAGACAAGAGGAAGTT
caa gaa ctt
TACTCTCCAATTTTTCTTATTGTTGCGGCAATAGTGTTTATAACACTTTGC

25416 9 3 0 3 ORF3a-FTIG8L
CAAATTACATTACACATAAACGAACTTATGGATTTGTTTATGAGAATCTT
cac aat tgg
AACTGTAACTTTGAAGCAAGGTGAAATCAAGGATGCTACTCCTTCAGATTT

25416 15 3 0 3 ORF3a-FTIGTV8L
CAAATTACATTACACATAAACGAACTTATGGATTTGTTTATGAGAATCTT
cac aat tgg aac tgt
AACTTTGAAGCAAGGTGAAATCAAGGATGCTACTCCTTCAGATTTTGTTCG

28107 11 3 0 3 ORF8-QYID72fs
CACCTTTAATTGAATTGTGCGTGGATGAGGCTGGTTCTAAATCACCCATT
cag tac atc ga

TATCGGTAATTATACAGTTTCCTGTTTACCTTTTACAATTAATTGCCAGGA

29743 9 3 0 3
TAACATTAGGGAGGACTTGAAAGAGCCACCACATTTTCACCGAGGCCACG
cgg agt acg
ATCGAGTGTACAGTGAACAATGCTAGGGAGAGCTGCCTATATGGAAGAGCC

28896 12 3 0 3 N-ARMA208del
CAACAGTTCAAGAAATTCAACTCCAGGCAGCAGTAGGGGAACTTCTCCTG
cta gaa tgg ctg
GCAATGGCGGTGATGCTGCTCTTGCTTTGCTGCTGCTTGACAGATTGAACC

27676 16 3 0 3 ORF7a-ELYSPI95fs
GTGCCAGATCAGTTTCACCTAAACTGTTCATCAGACAAGAGGAAGTTCAA
gaa ctt tac tct cca a
TTTTTCTTATTGTTGCGGCAATAGTGTTTATAACACTTTGCTTCACACTCA

29734 28 3 0 3
ATCAGTGTGTAACATTAGGGAGGACTTGAAAGAGCCACCACATTTTCACC
gag gcc acg cgg agt acg atc gag tgt a
CAGTGAACAATGCTAGGGAGAGCTGCCTATATGGAAGAGCCCTAATGTGTA

27572 15 3 0 3 ORF7a-STQFAF60I
TTCACCATTTCATCCTCTAGCTGATAACAAATTTGCACTGACTTGCTTTA
gca ctc aat ttg ctt
TTGCTTGTCCTGACGGCGTAAAACACGTCTATCAGTTACGTGCCAGATCAG

29742 19 3 0 3
GTAACATTAGGGAGGACTTGAAAGAGCCACCACATTTTCACCGAGGCCAC
gcg gag tac gat cga gtg t
ACAGTGAACAATGCTAGGGAGAGCTGCCTATATGGAAGAGCCCTAATGTGT

27593 25 3 0 3 ORF7a-CPDGVKHVY67fs
TGATAACAAATTTGCACTGACTTGCTTTAGCACTCAATTTGCTTTTGCTT
gtc ctg acg gcg taa aac acg tct a
TCAGTTACGTGCCAGATCAGTTTCACCTAAACTGTTCATCAGACAAGAGGA

29757 10 3 0 3
ACTTGAAAGAGCCACCACATTTTCACCGAGGCCACGCGGAGTACGATCGA
gtg tac agt g
AACAATGCTAGGGAGAGCTGCCTATATGGAAGAGCCCTAATGTGTAAAATT

29748 8 3 0 3
TTAGGGAGGACTTGAAAGAGCCACCACATTTTCACCGAGGCCACGCGGAG
tac gat cg
AGTGTACAGTGAACAATGCTAGGGAGAGCTGCCTATATGGAAGAGCCCTAA

28246 7 3 0 3 ORF8-LDF118fs
TTGTTCGTTCTATGAAGACTTTTTAGAGTATCATGACGTTCGTGTTGTTT
tag att t
CATCTAAACGAACAAACTAAAATGTCTGATAATGGACCCCAAAATCAGCGA

29753 16 3 0 3
GAGGACTTGAAAGAGCCACCACATTTTCACCGAGGCCACGCGGAGTACGA

tcg agt gta cag tga a
CAATGCTAGGGAGAGCTGCCTATATGGAAGAGCCCTAATGTGTAAAATTAA

27266 24 3 0 3 ORF6-FKVSIWNLD22Y
TGACTTTCAGGTTACTATAGCAGAGATATTACTAATTATTATGAGGACTT
tta aag ttt cca ttt gga atc ttg
ATTACATCATAAACCTCATAATTAAAAATTTATCTAAGTCACTAACTGAGA

3836 9 3 0 3 ORF1a-LEM1191del
ACTTAGCTGTCTTTGATAAAAATCTCTATGACAAACTTGTTTCAAGCTTT
ttg gaa atg
AAGAGTGAAAAGCAAGTTGAACAAAAGATCGCTGAGATTCCTAAAGAGGAA

27674 13 2 0 3 ORF7a-QELYS94fs
ACGTGCCAGATCAGTTTCACCTAAACTGTTCATCAGACAAGAGGAAGTTC
aag aac ttt act c
TCCAATTTTTCTTATTGTTGCGGCAATAGTGTTTATAACACTTTGCTTCAC

3336 12 2 0 3 ORF1a-EVNSF1024V
GGTTCAACCTCAATTAGAGATGGAACTTACACCAGTTGTTCAGACTATTG
aag tga ata gtt
TTAGTGGTTATTTAAAACTTACTGACAATGTATACATTAAAAATGCAGACA

27548 8 2 0 3 ORF7a-NKF52fs
CTCTTCTGGAACATACGAGGGCAATTCACCATTTCATCCTCTAGCTGATA
aca aat tt
GCACTGACTTGCTTTAGCACTCAATTTGCTTTTGCTTGTCCTGACGGCGTA

29723 10 2 0 3
AGCAATCTTTAATCAGTGTGTAACATTAGGGAGGACTTGAAAGAGCCACC
aca ttt tca c
CGAGGCCACGCGGAGTACGATCGAGTGTACAGTGAACAATGCTAGGGAGAG

21634 12 2 0 3 S-LPPAY24F
TTGCCACTAGTCTCTAGTCAGTGTGTTAATCTTACAACCAGAACTCAATT
acc ccc tgc ata
CACTAATTCTTTCACACGTGGTGTTTATTACCCTGACAAAGTTTTCAGATC

27574 11 2 0 3 ORF7a-TQFA61fs
CACCATTTCATCCTCTAGCTGATAACAAATTTGCACTGACTTGCTTTAGC
act caa ttt gc
TTTTGCTTGTCCTGACGGCGTAAAACACGTCTATCAGTTACGTGCCAGATC

29752 10 2 0 3
GGAGGACTTGAAAGAGCCACCACATTTTCACCGAGGCCACGCGGAGTACG
atc gag tgt a
CAGTGAACAATGCTAGGGAGAGCTGCCTATATGGAAGAGCCCTAATGTGTA

27770 13 2 0 3 ORF7b-SLIDF5fs
TATAACACTTTGCTTCACACTCAAAAGAAAGACAGAATGATTGAACTTTC
att aat tga ctt c
TATTTGTGCTTTTTAGCCTTTCTGCTATTCCTTGTTTTAATTATGCTTATT

29686 8 2 0 3

ATAGCACAAGTAGATGTAGTTAACTTTAATCTCACATAGCAATCTTTAAT
cag tgt gt
AACATTAGGGAGGACTTGAAAGAGCCACCACATTTTCACCGAGGCCACGCG

28146 14 2 0 3 ORF8-PFTIN85fs
AATCACCCATTCAGTACATCGATATCGGTAATTATACAGTTTCCTGTTTA
cct ttt aca att aa
TTGCCAGGAACCTAAATTGGGTAGTCTTGTAGTGCGTTGTTCGTTCTATGA

29741 7 2 0 3
TGTAACATTAGGGAGGACTTGAAAGAGCCACCACATTTTCACCGAGGCCA
cgc gga g
TACGATCGAGTGTACAGTGAACAATGCTAGGGAGAGCTGCCTATATGGAAG

29741 26 2 0 3
TGTAACATTAGGGAGGACTTGAAAGAGCCACCACATTTTCACCGAGGCCA
cgc gga gta cga tcg agt gta cag tg
AACAATGCTAGGGAGAGCTGCCTATATGGAAGAGCCCTAATGTGTAAAATT

25424 15 2 0 3 ORF3a-GTVTLK11E
ATTACACATAAACGAACTTATGGATTTGTTTATGAGAATCTTCACAATTG
gaa ctg taa ctt tga
AGCAAGGTGAAATCAAGGATGCTACTCCTTCAGATTTTGTTCGCGCTACTG

27376 10 2 0 3 ORF6-EID*59fs
AGTCACTAACTGAGAATAAATATTCTCAATTAGATGAAGAGCAACCAATG
gag att gat t
AAACGAACATGAAAATTATTCTTTTCTTGGCACTGATAACACTCGCTACTT

29745 11 2 0 3
ACATTAGGGAGGACTTGAAAGAGCCACCACATTTTCACCGAGGCCACGCG
gag tac gat cg
AGTGTACAGTGAACAATGCTAGGGAGAGCTGCCTATATGGAAGAGCCCTAA

27566 8 2 0 3 ORF7a-CFS58fs
GGGCAATTCACCATTTCATCCTCTAGCTGATAACAAATTTGCACTGACTT
gct tta gc
ACTCAATTTGCTTTTGCTTGTCCTGACGGCGTAAAACACGTCTATCAGTTA

29736 16 2 0 3
CAGTGTGTAACATTAGGGAGGACTTGAAAGAGCCACCACATTTTCACCGA
ggc cac gcg gag tac g
ATCGAGTGTACAGTGAACAATGCTAGGGAGAGCTGCCTATATGGAAGAGCC

29736 17 2 0 3
CAGTGTGTAACATTAGGGAGGACTTGAAAGAGCCACCACATTTTCACCGA
ggc cac gcg gag tac ga
TCGAGTGTACAGTGAACAATGCTAGGGAGAGCTGCCTATATGGAAGAGCCC

29759 17 2 0 3
TTGAAAGAGCCACCACATTTTCACCGAGGCCACGCGGAGTACGATCGAGT
gta cag tga aca atg ct
AGGGAGAGCTGCCTATATGGAAGAGCCCTAATGTGTAAAATTAATTTTAGT

27576 7 2 0 3 ORF7a-TQF61fs
CCATTTCATCCTCTAGCTGATAACAAATTTGCACTGACTTGCTTTAGCAC
tca att t
GCTTTTGCTTGTCCTGACGGCGTAAAACACGTCTATCAGTTACGTGCCAGA

27640 7 2 0 3 ORF7a-SPK83fs
CTTGTCCTGACGGCGTAAAACACGTCTATCAGTTACGTGCCAGATCAGTT
tca cct a
AACTGTTCATCAGACAAGAGGAAGTTCAAGAACTTTACTCTCCAATTTTTC

28150 8 2 0 3 ORF8-FTI86fs
ACCCATTCAGTACATCGATATCGGTAATTATACAGTTTCCTGTTTACCTT
tta caa tt
AATTGCCAGGAACCTAAATTGGGTAGTCTTGTAGTGCGTTGTTCGTTCTAT

28066 31 2 0 3 ORF8-IELCVDEAGSK58fs
CTATTCTAAATGGTATATTAGAGTAGGAGCTAGAAAATCAGCACCTTTAA
ttg aat tgt gcg tgg atg agg ctg gtt cta a
ATCACCCATTCAGTACATCGATATCGGTAATTATACAGTTTCCTGTTTACC

21981 12 2 0 3 S-FLGV140del
TACTAATGTTGTTATTAAAGTCTGTGAATTTCAATTTTGTAATGATCCAT
ttt tgg gtg ttt
ATTACCACAAAAACAACAAAAGTTGGATGGAAAGTGAGTTCAGAGTTTATT

27677 15 1 0 3 ORF7a-ELYSPI95V
TGCCAGATCAGTTTCACCTAAACTGTTCATCAGACAAGAGGAAGTTCAAG
aac ttt act ctc caa
TTTTTCTTATTGTTGCGGCAATAGTGTTTATAACACTTTGCTTCACACTCA

28210 9 1 0 3 ORF8-EDFL106V
TTGCCAGGAACCTAAATTGGGTAGTCTTGTAGTGCGTTGTTCGTTCTATG
aag act ttt
TAGAGTATCATGACGTTCGTGTTGTTTTAGATTTCATCTAAACGAACAAAC

28231 15 1 0 3 ORF8-DVRVVL113V
TAGTCTTGTAGTGCGTTGTTCGTTCTATGAAGACTTTTTAGAGTATCATG
acg ttc gtg ttg ttt
TAGATTTCATCTAAACGAACAAACTAAAATGTCTGATAATGGACCCCAAAA

6610 9 1 0 3 ORF1a-RVLG2115S
TATGTAGACAATTCTAGTCTTACTATTAAGAAACCTAATGAATTATCTAG
agt att agg
TTTGAAAACCCTTGCTACTCATGGTTTAGCTGCTGTTAATAGTGTCCCTTG

27714 21 1 0 3 ORF7a-VFITLCF108del
GAGGAAGTTCAAGAACTTTACTCTCCAATTTTTCTTATTGTTGCGGCAAT
agt gtt tat aac act ttg ctt
CACACTCAAAAGAAAGACAGAATGATTGAACTTTCATTAATTGACTTCTAT

6551 15 1 0 3 ORF1a-MAAYV2096del
CAGCAAATAATAGTTTAAAAATTACAGAAGAGGTTGGCCACACAGATCTA
atg gct gct tat gta
GACAATTCTAGTCTTACTATTAAGAAACCTAATGAATTATCTAGAGTATTA

27950 7 1 0 3 ORF8-ECS19fs
TTTCTTGTTTCTTAGGAATCATCACAACTGTAGCTGCATTTCACCAAGA
atg tag t
TTACAGTCATGTACTCAACATCAACCATATGTAGTTGATGACCCGTGTCCT

513 15 1 0 3 ORF1a-HVMVE83del
GCCCTATGTGTTCATCAAACGTTCGGATGCTCGAACTGCACCTCATGGTC
atg tta tgg ttg agc
TGGTAGCAGAACTCGAAGGCATTCAGTACGGTCGTAGTGGTGAGACACTTG

27579 19 1 0 3 ORF7a-QFAFACP62fs
TTTCATCCTCTAGCTGATAACAAATTTGCACTGACTTGCTTTAGCACTCA
att tgc ttt tgc ttg tcc t
GACGGCGTAAAACACGTCTATCAGTTACGTGCCAGATCAGTTTCACCTAAA

3290 24 1 0 3 ORF1a-QPQLEMEL1009del
ACGGCAGTGAGGACAATCAGACAACTACTATTCAAACAATTGTTGAGGTT
caa cct caa tta gag atg gaa ctt
ACACCAGTTGTTCAGACTATTGAAGTGAATAGTTTTAGTGGTTATTTAAAA

27920 28 1 0 3 ORF8-IITTVAAFHQ9fs
GAAACTTGTCACGCCTAAACGAACATGAAATTTCTTGTTTTCTTAGGAAT
cat cac aac tgt agc tgc att tca cca a
GAATGTAGTTTACAGTCATGTACTCAACATCAACCATATGTAGTTGATGAC

27370 22 1 0 3 ORF6-PMEID*57del
TATCTAAGTCACTAACTGAGAATAAATATTCTCAATTAGATGAAGAGCAA
cca atg gag att gat taa acg a
ACATGAAAATTATTCTTTTCTTGGCACTGATAACACTCGCTACTTGTGAGC

27275 8 1 0 3 ORF6-SIW25fs
GGTTACTATAGCAGAGATATTACTAATTATTATGAGGACTTTTAAAGTTT
cca ttt gg
AATCTTGATTACATCATAAACCTCATAATTAAAAATTTATCTAAGTCACTA

28005 13 1 0 3 ORF8-PIHFY38fs
GTTTACAGTCATGTACTCAACATCAACCATATGTAGTTGATGACCCGTGT
cct att cac ttc t
ATTCTAAATGGTATATTAGAGTAGGAGCTAGAAAATCAGCACCTTTAATTG

27595 7 1 0 3 ORF7a-PDG68fs
ATAACAAATTTGCACTGACTTGCTTTAGCACTCAATTTGCTTTTGCTTGT
cct gac g
GCGTAAAACACGTCTATCAGTTACGTGCCAGATCAGTTTCACCTAAACTGT

29733 19 1 0 3
AATCAGTGTGTAACATTAGGGAGGACTTGAAAGAGCCACCACATTTTCAC
cga ggc cac gcg gag tac g
ATCGAGTGTACAGTGAACAATGCTAGGGAGAGCTGCCTATATGGAAGAGCC

29578 12 1 0 3 ORF10-AFPF8del
GCCTAAACTCATGCAGACCACACAAGGCAGATGGGCTATATAAACGTTTT
cgc ttt tcc gtt

TACGATATATAGTCTACTCTTGTGCAGAATGAATTCTCGTAACTACATAGC

27625 8 1 0 3 ORF7a-RAR78fs
CTCAATTTGCTTTTGCTTGTCCTGACGGCGTAAAACACGTCTATCAGTTA
cgt gcc ag
ATCAGTTTCACCTAAACTGTTCATCAGACAAGAGGAAGTTCAAGAACTTTA

25549 11 1 0 3 ORF3a-LAVF53fs
CGATACAAGCCTCACTCCCTTTCGGATGGCTTATTGTTGGCGTTGCACTT
ctt gct gtt tt
TCAGAGCGCTTCCAAAATCATAACCCTCAAAAAGAGATGGCAACTAGCACT

27765 10 1 0 3 ORF7b-LSLI4fs
GTGTTTATAACACTTTGCTTCACACTCAAAAGAAAGACAGAATGATTGAA
ctt tca tta a
TTGACTTCTATTTGTGCTTTTTAGCCTTTCTGCTATTCCTTGTTTTAATTA

25517 15 1 0 3 ORF3a-PFGWLI42L
TTCAGATTTTGTTCGCGCTACTGCAACGATACCGATACAAGCCTCACTCC
ctt tcg gat ggc tta
TTGTTGGCGTTGCACTTCTTGCTGTTTTTCAGAGCGCTTCCAAAATCATAA

28899 12 1 0 3 N-RMAG209del
CAGTTCAAGAAATTCAACTCCAGGCAGCAGTAGGGGAACTTCTCCTGCTA
gaa tgg ctg gca
ATGGCGGTGATGCTGCTCTTGCTTTGCTGCTGCTTGACAGATTGAACCAGC

25444 9 1 0 3 ORF3a-GEI18del
TGGATTTGTTTATGAGAATCTTCACAATTGGAACTGTAACTTTGAAGCAA
ggt gaa atc
AAGGATGCTACTCCTTCAGATTTTGTTCGCGCTACTGCAACGATACCGATA

29759 15 1 0 3
TTGAAAGAGCCACCACATTTTCACCGAGGCCACGCGGAGTACGATCGAGT
gta cag tga aca atg
CTAGGGAGAGCTGCCTATATGGAAGAGCCCTAATGTGTAAAATTAATTTTA

6576 21 1 0 3 ORF1a-SLTIKKP2104del
AGAAGAGGTTGGCCACACAGATCTAATGGCTGCTTATGTAGACAATTCTA
gtc tta cta tta aga aac cta
ATGAATTATCTAGAGTATTAGGTTTGAAAACCCTTGCTACTCATGGTTTAG

28237 18 1 0 3 ORF8-RVVLDFI115L
TGTAGTGCGTTGTTCGTTCTATGAAGACTTTTTAGAGTATCATGACGTTC
gtg ttg ttt tag att tca
TCTAAACGAACAAACTAAAATGTCTGATAATGGACCCCAAAATCAGCGAAA

28151 8 1 0 3 ORF8-FTI86fs
CCCATTCAGTACATCGATATCGGTAATTATACAGTTTCCTGTTTACCTTT
tac aat ta
ATTGCCAGGAACCTAAATTGGGTAGTCTTGTAGTGCGTTGTTCGTTCTATG

3907 30 1 0 3 ORF1a-FITESKPSVEQ1214L
CAAGTTGAACAAAAGATCGCTGAGATTCCTAAAGAGGAAGTTAAGCCATT

tat aac tga aag taa acc ttc agt tga aca
GAGAAAACAAGATGATAAGAAAATCAAAGCTTGTGTTGAAGAAGTTACAAC

29729 26 1 0 3
CTTTAATCAGTGTGTAACATTAGGGAGGACTTGAAAGAGCCACCACATTT
tca ccg agg cca cgc gga gta cga tc
GAGTGTACAGTGAACAATGCTAGGGAGAGCTGCCTATATGGAAGAGCCCTA

29753 13 1 0 3
GAGGACTTGAAAGAGCCACCACATTTTCACCGAGGCCACGCGGAGTACGA
tcg agt gta cag t
GAACAATGCTAGGGAGAGCTGCCTATATGGAAGAGCCCTAATGTGTAAAAT

27696 28 1 0 3 ORF7a-FLIVAAIVFI101fs
AAACTGTTCATCAGACAAGAGGAAGTTCAAGAACTTTACTCTCCAATTTT
tct tat tgt tgc ggc aat agt gtt tat a
ACACTTTGCTTCACACTCAAAAGAAAGACAGAATGATTGAACTTTCATTAA

27761 8 1 0 3 ORF7b-IEL2fs
AATAGTGTTTATAACACTTTGCTTCACACTCAAAAGAAAGACAGAATGAT
tga act tt
CATTAATTGACTTCTATTTGTGCTTTTTAGCCTTTCTGCTATTCCTTGTTT

29758 8 1 0 3
CTTGAAAGAGCCACCACATTTTCACCGAGGCCACGCGGAGTACGATCGAG
tgt aca gt
GAACAATGCTAGGGAGAGCTGCCTATATGGAAGAGCCCTAATGTGTAAAAT

29680 7 1 0 3
AACTACATAGCACAAGTAGATGTAGTTAACTTTAATCTCACATAGCAATC
ttt aat c
AGTGTGTAACATTAGGGAGGACTTGAAAGAGCCACCACATTTTCACCGAGG

27585 8 1 0 3 ORF7a-AFA64fs
CCTCTAGCTGATAACAAATTTGCACTGACTTGCTTTAGCACTCAATTTGC
ttt tgc tt
GTCCTGACGGCGTAAAACACGTCTATCAGTTACGTGCCAGATCAGTTTCAC
