# Supplementary material for: Deletions across the SARS-CoV-2 Genome: Molecular Mechanisms and Putative Functional Consequences of Deletions in Accessory Genes
Source: Microorganisms. 2023 Jan 16;11(1):229. doi: 10.3390/microorganisms11010229 (PMC9862619; doi:10.3390/microorganisms11010229)
Supplement: Supplementary file 1 [file microorganisms-11-00229-s001.zip › Figure S3.pdf]

Supplementary Figure S3. Contexts of long deletions in the bin #5 of ORF7a.
The 1st number is the postion and the 5th number is the number of instances
followed by gene names.

27570 13 4 2 54 ORF7a-FSTQF59fs
AATTCACCATTTCATCCTCTAGCTGATAACAAATTTGCACTGACTTGCTT
tag cac tca a
GCTTTTGCTTGTCCTGACGGCGTAAAACACGTCTATCAGTTACGTGCCAGA

27569 55 2 1 90 ORF7a-FSTQFAFACPDGVKHVYQL59fs
CAATTCACCATTTCATCCTCTAGCTGATAACAAATTTGCACTGACTTGCT
tta gca ctc aat ttg ctt ttg ctt gtc ctg acg gcg taa aac
ACGTGCCAGATCAGTTTCACCTAAACTGTTCATCAGACAAGAGGAAGTTCA

GGAACATACGAGGGCAATTCACCATTTCATCCTCTAGCTGATAACAAATT
tgc act gac ttg ctt tag cac tca att tgc ttt tgc ttg tcc tga cgg cgt a
ACGTGCCAGATCAGTTTCACCTAAACTGTTCATCAGACAAGAGGAAGTTCA

27555 92 13 0 200 ORF7a-FALTCFSTQFAFACPDGVKHVYQLRARSVSP54fs
GGAACATACGAGGGCAATTCACCATTTCATCCTCTAGCTGATAACAAATT
tgc act gac ttg ctt tag cac tca att tgc ttt tgc ttg tcc tga cgg cgt aaa aca cgt cta tca gtt
AACTGTTCATCAGACAAGAGGAAGTTCAAGAACTTTACTCTCCAATTTTTC

27571 16 5 0 97 ORF7a-STQFAF60fs
ATTCACCATTTCATCCTCTAGCTGATAACAAATTTGCACTGACTTGCTTT
agc act caa ttt
TTGCTTGTCCTGACGGCGTAAAACACGTCTATCAGTTACGTGCCAGATCAG

27575 23 1 0 59 ORF7a-TQFAFACP61fs
ACCATTTCATCCTCTAGCTGATAACAAATTTGCACTGACTTGCTTTAGCA
ctc aat ttg ctt ttg ctt
GACGGCGTAAAACACGTCTATCAGTTACGTGCCAGATCAGTTTCACCTAAA

27555 52 3 0 54 ORF7a-FALTCFSTQFAFACPDGV54fs
GGAACATACGAGGGCAATTCACCATTTCATCCTCTAGCTGATAACAAATT
tgc act gac ttg ctt tag cac tca att tgc ttt tgc ttg
AAACACGTCTATCAGTTACGTGCCAGATCAGTTTCACCTAAACTGTTCATC

ATTCACCATTTCATCCTCTAGCTGATAACAAATTTGCACTGACTTGCTTT
agc act caa ttt gc
GCTTGTCCTGACGGCGTAAAACACGTCTATCAGTTACGTGCCAGATCAGTT

27574 17 2 0 46 ORF7a-TQFAFA61fs
CACCATTTCATCCTCTAGCTGATAACAAATTTGCACTGACTTGCTTTAGC
act caa ttt gct t
TTGTCCTGACGGCGTAAAACACGTCTATCAGTTACGTGCCAGATCAGTTTC

27561 16 2 0 42 ORF7a-LTCFST56fs
TACGAGGGCAATTCACCATTTCATCCTCTAGCTGATAACAAATTTGCACT
gac ttg ctt tag
CAATTTGCTTTTGCTTGTCCTGACGGCGTAAAACACGTCTATCAGTTACGT

27566 28 4 0 41 ORF7a-CFSTQFAFAC58fs
GGGCAATTCACCATTTCATCCTCTAGCTGATAACAAATTTGCACTGACTT
gct tta gca ctc aat ttg ctt
TCCTGACGGCGTAAAACACGTCTATCAGTTACGTGCCAGATCAGTTTCACC

27554 92 2 0 41 ORF7a-FALTCFSTQFAFACPDGVKHVYQLRARSVSP54fs
TGGAACATACGAGGGCAATTCACCATTTCATCCTCTAGCTGATAACAAAT
ttg cac tga ctt gct tta gca ctc aat ttg ctt ttg ctt gtc ctg acg gcg taa aac acg tct atc agt
AAACTGTTCATCAGACAAGAGGAAGTTCAAGAACTTTACTCTCCAATTTTT

GGAACATACGAGGGCAATTCACCATTTCATCCTCTAGCTGATAACAAATT
tgc act gac ttg ctt tag cac tca att tgc ttt tgc ttg
AAAACACGTCTATCAGTTACGTGCCAGATCAGTTTCACCTAAACTGTTCAT

GGAACATACGAGGGCAATTCACCATTTCATCCTCTAGCTGATAACAAATT
tgc act gac ttg ctt tag
ATTTGCTTTTGCTTGTCCTGACGGCGTAAAACACGTCTATCAGTTACGTGC

27570 19 1 0 36 ORF7a-FSTQFAF59fs
AATTCACCATTTCATCCTCTAGCTGATAACAAATTTGCACTGACTTGCTT
tag cac tca att tgc
GCTTGTCCTGACGGCGTAAAACACGTCTATCAGTTACGTGCCAGATCAGTT

27556 67 2 0 23 ORF7a-ALTCFSTQFAFACPDGVKHVYQL55fs
GAACATACGAGGGCAATTCACCATTTCATCCTCTAGCTGATAACAAATTT
gca ctg act tgc ttt agc act caa ttt gct ttt gct tgt cct gac ggc gta
TACGTGCCAGATCAGTTTCACCTAAACTGTTCATCAGACAAGAGGAAGTTC

27571 17 13 0 22 ORF7a-STQFAF60fs
ATTCACCATTTCATCCTCTAGCTGATAACAAATTTGCACTGACTTGCTTT
agc act caa ttt g
TGCTTGTCCTGACGGCGTAAAACACGTCTATCAGTTACGTGCCAGATCAGT

27555 44 3 0 22 ORF7a-FALTCFSTQFAFACP54fs
GGAACATACGAGGGCAATTCACCATTTCATCCTCTAGCTGATAACAAATT
tgc act gac ttg ctt tag cac tca att tgc ttt
ACGGCGTAAAACACGTCTATCAGTTACGTGCCAGATCAGTTTCACCTAAAC

27556 85 9 0 20 ORF7a-ALTCFSTQFAFACPDGVKHVYQLRARSVS55fs
GAACATACGAGGGCAATTCACCATTTCATCCTCTAGCTGATAACAAATTT
gca ctg act tgc ttt agc act caa ttt gct ttt gct tgt cct gac ggc gta aaa cac gtc tat c
CACCTAAACTGTTCATCAGACAAGAGGAAGTTCAAGAACTTTACTCTCCAA

27572 53 5 0 19 ORF7a-STQFAFACPDGVKHVYQL60fs
TTCACCATTTCATCCTCTAGCTGATAACAAATTTGCACTGACTTGCTTTA
gca ctc aat ttg ctt ttg ctt gtc ctg acg gcg taa aac a
CGTGCCAGATCAGTTTCACCTAAACTGTTCATCAGACAAGAGGAAGTTCAA

27555 82 4 0 14 ORF7a-FALTCFSTQFAFACPDGVKHVYQLRARS54fs
GGAACATACGAGGGCAATTCACCATTTCATCCTCTAGCTGATAACAAATT
tgc act gac ttg ctt tag cac tca att tgc ttt tgc ttg tcc tga cgg cgt aaa aca cgt ct
GTTTCACCTAAACTGTTCATCAGACAAGAGGAAGTTCAAGAACTTTACTCT

27572 71 3 0 14 ORF7a-STQFAFACPDGVKHVYQLRARSVS60fs

TTCACCATTTCATCCTCTAGCTGATAACAAATTTGCACTGACTTGCTTTA
gca ctc aat ttg ctt ttg ctt gtc ctg acg gcg taa aac acg tct atc agt tac
CCTAAACTGTTCATCAGACAAGAGGAAGTTCAAGAACTTTACTCTCCAATT

27572 7 4 0 11 ORF7a-STQ60fs
TTCACCATTTCATCCTCTAGCTGATAACAAATTTGCACTGACTTGCTTTA
gca ctc
ATTTGCTTTTGCTTGTCCTGACGGCGTAAAACACGTCTATCAGTTACGTGC

27570 55 2 0 11 ORF7a-FSTQFAFACPDGVKHVYQL59fs
AATTCACCATTTCATCCTCTAGCTGATAACAAATTTGCACTGACTTGCTT
tag cac tca att tgc ttt tgc ttg tcc tga cgg cgt aaa aca
CGTGCCAGATCAGTTTCACCTAAACTGTTCATCAGACAAGAGGAAGTTCAA

27548 7 3 0 10 ORF7a-NKF52fs
CTCTTCTGGAACATACGAGGGCAATTCACCATTTCATCCTCTAGCTGATA
aca aat
TGCACTGACTTGCTTTAGCACTCAATTTGCTTTTGCTTGTCCTGACGGCGT

27562 79 3 0 10 ORF7a-TCFSTQFAFACPDGVKHVYQLRARSVS57fs
ACGAGGGCAATTCACCATTTCATCCTCTAGCTGATAACAAATTTGCACTG
act tgc ttt agc act caa ttt gct ttt gct tgt cct gac ggc gta aaa cac gtc tat cag
CACCTAAACTGTTCATCAGACAAGAGGAAGTTCAAGAACTTTACTCTCCAA

GAACATACGAGGGCAATTCACCATTTCATCCTCTAGCTGATAACAAATTT
gca ctg act tgc ttt agc act caa ttt gct ttt gct tgt cct gac ggc
CAGTTACGTGCCAGATCAGTTTCACCTAAACTGTTCATCAGACAAGAGGAA

27566 13 1 0 10 ORF7a-CFSTQ58fs
GGGCAATTCACCATTTCATCCTCTAGCTGATAACAAATTTGCACTGACTT
gct tta gca c
ATTTGCTTTTGCTTGTCCTGACGGCGTAAAACACGTCTATCAGTTACGTGC

27572 8 4 0 9 ORF7a-STQ60fs
TTCACCATTTCATCCTCTAGCTGATAACAAATTTGCACTGACTTGCTTTA
gca ctc
TTTGCTTTTGCTTGTCCTGACGGCGTAAAACACGTCTATCAGTTACGTGCC

27566 65 2 0 9 ORF7a-CFSTQFAFACPDGVKHVYQLRA58fs
GGGCAATTCACCATTTCATCCTCTAGCTGATAACAAATTTGCACTGACTT
gct tta gca ctc aat ttg ctt ttg ctt gtc ctg acg gcg taa aac acg t
AGATCAGTTTCACCTAAACTGTTCATCAGACAAGAGGAAGTTCAAGAACTT

27566 76 1 0 9 ORF7a-CFSTQFAFACPDGVKHVYQLRARSVS58fs
GGGCAATTCACCATTTCATCCTCTAGCTGATAACAAATTTGCACTGACTT
gct tta gca ctc aat ttg ctt ttg ctt gtc ctg acg gcg taa aac acg tct atc agt
ACCTAAACTGTTCATCAGACAAGAGGAAGTTCAAGAACTTTACTCTCCAAT

27555 91 1 0 9 ORF7a-FALTCFSTQFAFACPDGVKHVYQLRARSVSP54fs
GGAACATACGAGGGCAATTCACCATTTCATCCTCTAGCTGATAACAAATT
tgc act gac ttg ctt tag cac tca att tgc ttt tgc ttg tcc tga cgg cgt aaa aca cgt cta tca gtt
AAACTGTTCATCAGACAAGAGGAAGTTCAAGAACTTTACTCTCCAATTTTT

27571 11 5 0 8 ORF7a-STQF60fs

ATTCACCATTTCATCCTCTAGCTGATAACAAATTTGCACTGACTTGCTTT
agc act caa
TGCTTTTGCTTGTCCTGACGGCGTAAAACACGTCTATCAGTTACGTGCCAG

27549 115 1 0 8 ORF7a-NKFALTCFSTQFAFACPDGVKHVYQLRARSVSPKLFIRQ52fs
TCTTCTGGAACATACGAGGGCAATTCACCATTTCATCCTCTAGCTGATAA
caa att tgc act gac ttg ctt tag cac tca att tgc ttt tgc ttg tcc tga cgg cgt aaa aca cgt cta tca gtt acg tgc cag atc
GAGGAAGTTCAAGAACTTTACTCTCCAATTTTTCTTATTGTTGCGGCAATA

GGCAATTCACCATTTCATCCTCTAGCTGATAACAAATTTGCACTGACTTG
ctt tag cac tca att tgc ttt
TCCTGACGGCGTAAAACACGTCTATCAGTTACGTGCCAGATCAGTTTCACC

CACCATTTCATCCTCTAGCTGATAACAAATTTGCACTGACTTGCTTTAGC
act caa ttt gct ttt gct tgt cc
GTAAAACACGTCTATCAGTTACGTGCCAGATCAGTTTCACCTAAACTGTTC

27573 49 2 0 6 ORF7a-STQFAFACPDGVKHVYQ60fs
TCACCATTTCATCCTCTAGCTGATAACAAATTTGCACTGACTTGCTTTAG
cac tca att tgc ttt tgc ttg tcc tga cgg cgt aaa a
TTACGTGCCAGATCAGTTTCACCTAAACTGTTCATCAGACAAGAGGAAGTT

GAACATACGAGGGCAATTCACCATTTCATCCTCTAGCTGATAACAAATTT
gca ctg act tgc ttt agc act caa ttt gct ttt gct tgt cct gac ggc gta aaa cac gtc tat
TCACCTAAACTGTTCATCAGACAAGAGGAAGTTCAAGAACTTTACTCTCCA

27570 71 2 0 6 ORF7a-FSTQFAFACPDGVKHVYQLRARSV59fs
AATTCACCATTTCATCCTCTAGCTGATAACAAATTTGCACTGACTTGCTT
tag cac tca att tgc ttt tgc ttg tcc tga cgg cgt aaa aca cgt cta tca gtt
CACCTAAACTGTTCATCAGACAAGAGGAAGTTCAAGAACTTTACTCTCCAA

27572 10 3 0 5 ORF7a-STQF60fs
TTCACCATTTCATCCTCTAGCTGATAACAAATTTGCACTGACTTGCTTTA
gca ctc aa
TGCTTTTGCTTGTCCTGACGGCGTAAAACACGTCTATCAGTTACGTGCCAG

27549 31 2 0 5 ORF7a-NKFALTCFSTQ52fs
TCTTCTGGAACATACGAGGGCAATTCACCATTTCATCCTCTAGCTGATAA
caa att tgc act gac ttg ctt tag
TTTGCTTTTGCTTGTCCTGACGGCGTAAAACACGTCTATCAGTTACGTGCC

27556 64 1 0 5 ORF7a-ALTCFSTQFAFACPDGVKHVYQ55fs
GAACATACGAGGGCAATTCACCATTTCATCCTCTAGCTGATAACAAATTT
gca ctg act tgc ttt agc act caa ttt gct ttt gct tgt cct gac ggc
AGTTACGTGCCAGATCAGTTTCACCTAAACTGTTCATCAGACAAGAGGAAG

27566 64 1 0 5 ORF7a-CFSTQFAFACPDGVKHVYQLRA58fs
GGGCAATTCACCATTTCATCCTCTAGCTGATAACAAATTTGCACTGACTT
gct tta gca ctc aat ttg ctt ttg ctt gtc ctg acg gcg taa aac acg
CAGATCAGTTTCACCTAAACTGTTCATCAGACAAGAGGAAGTTCAAGAACT

27571 10 3 0 4 ORF7a-STQF60fs
ATTCACCATTTCATCCTCTAGCTGATAACAAATTTGCACTGACTTGCTTT
agc act ca

TTGCTTTTGCTTGTCCTGACGGCGTAAAACACGTCTATCAGTTACGTGCCA

27566 58 3 0 4 ORF7a-CFSTQFAFACPDGVKHVYQL58fs
GGGCAATTCACCATTTCATCCTCTAGCTGATAACAAATTTGCACTGACTT
gct tta gca ctc aat ttg ctt ttg ctt gtc ctg acg gcg taa aa
ACGTGCCAGATCAGTTTCACCTAAACTGTTCATCAGACAAGAGGAAGTTCA

27556 23 2 0 4 ORF7a-ALTCFSTQ55fs
GAACATACGAGGGCAATTCACCATTTCATCCTCTAGCTGATAACAAATTT
gca ctg act tgc ttt agc
ATTTGCTTTTGCTTGTCCTGACGGCGTAAAACACGTCTATCAGTTACGTGC

27556 32 2 0 4 ORF7a-ALTCFSTQFAF55fs
GAACATACGAGGGCAATTCACCATTTCATCCTCTAGCTGATAACAAATTT
gca ctg act tgc ttt agc act caa
TGCTTGTCCTGACGGCGTAAAACACGTCTATCAGTTACGTGCCAGATCAGT

27555 70 2 0 4 ORF7a-FALTCFSTQFAFACPDGVKHVYQL54fs
GGAACATACGAGGGCAATTCACCATTTCATCCTCTAGCTGATAACAAATT
tgc act gac ttg ctt tag cac tca att tgc ttt tgc ttg tcc tga cgg cgt aa
CGTGCCAGATCAGTTTCACCTAAACTGTTCATCAGACAAGAGGAAGTTCAA

27559 88 1 0 4 ORF7a-LTCFSTQFAFACPDGVKHVYQLRARSVSPK56fs
CATACGAGGGCAATTCACCATTTCATCCTCTAGCTGATAACAAATTTGCA
ctg act tgc ttt agc act caa ttt gct ttt gct tgt cct gac ggc gta aaa cac gtc tat cag tta
AACTGTTCATCAGACAAGAGGAAGTTCAAGAACTTTACTCTCCAATTTTTC

27566 16 1 0 4 ORF7a-CFSTQF58fs
GGGCAATTCACCATTTCATCCTCTAGCTGATAACAAATTTGCACTGACTT
gct tta gca ctc
TGCTTTTGCTTGTCCTGACGGCGTAAAACACGTCTATCAGTTACGTGCCAG

27553 56 1 0 4 ORF7a-FALTCFSTQFAFACPDGVK54fs
CTGGAACATACGAGGGCAATTCACCATTTCATCCTCTAGCTGATAACAAA
ttt gca ctg act tgc ttt agc act caa ttt gct ttt gct tgt
ACACGTCTATCAGTTACGTGCCAGATCAGTTTCACCTAAACTGTTCATCAG

TTCACCATTTCATCCTCTAGCTGATAACAAATTTGCACTGACTTGCTTTA
gca ctc aat ttg
TTGCTTGTCCTGACGGCGTAAAACACGTCTATCAGTTACGTGCCAGATCAG

27548 8 2 0 3 ORF7a-NKF52fs
CTCTTCTGGAACATACGAGGGCAATTCACCATTTCATCCTCTAGCTGATA
aca aat
GCACTGACTTGCTTTAGCACTCAATTTGCTTTTGCTTGTCCTGACGGCGTA

27574 11 2 0 3 ORF7a-TQFA61fs
CACCATTTCATCCTCTAGCTGATAACAAATTTGCACTGACTTGCTTTAGC
act caa ttt
TTTTGCTTGTCCTGACGGCGTAAAACACGTCTATCAGTTACGTGCCAGATC

27575 43 2 0 3 ORF7a-TQFAFACPDGVKHVY61fs
ACCATTTCATCCTCTAGCTGATAACAAATTTGCACTGACTTGCTTTAGCA
ctc aat ttg ctt ttg ctt gtc ctg acg gcg taa

TCAGTTACGTGCCAGATCAGTTTCACCTAAACTGTTCATCAGACAAGAGGA

27566 8 2 0 3 ORF7a-CFS58fs
GGGCAATTCACCATTTCATCCTCTAGCTGATAACAAATTTGCACTGACTT
gct tta
ACTCAATTTGCTTTTGCTTGTCCTGACGGCGTAAAACACGTCTATCAGTTA

GGGCAATTCACCATTTCATCCTCTAGCTGATAACAAATTTGCACTGACTT
gct tta gca ctc aat ttg ctt ttg ctt gtc ctg acg gcg
ATCAGTTACGTGCCAGATCAGTTTCACCTAAACTGTTCATCAGACAAGAGG

27576 7 2 0 3 ORF7a-TQF61fs
CCATTTCATCCTCTAGCTGATAACAAATTTGCACTGACTTGCTTTAGCAC
tca att
GCTTTTGCTTGTCCTGACGGCGTAAAACACGTCTATCAGTTACGTGCCAGA

27576 50 2 0 3 ORF7a-TQFAFACPDGVKHVYQL61fs
CCATTTCATCCTCTAGCTGATAACAAATTTGCACTGACTTGCTTTAGCAC
tca att tgc ttt tgc ttg tcc tga cgg cgt aaa aca cg
GTGCCAGATCAGTTTCACCTAAACTGTTCATCAGACAAGAGGAAGTTCAAG

27555 86 2 0 3 ORF7a-FALTCFSTQFAFACPDGVKHVYQLRARSV54fs
GGAACATACGAGGGCAATTCACCATTTCATCCTCTAGCTGATAACAAATT
tgc act gac ttg ctt tag cac tca att tgc ttt tgc ttg tcc tga cgg cgt aaa aca cgt cta tc
CACCTAAACTGTTCATCAGACAAGAGGAAGTTCAAGAACTTTACTCTCCAA

GGAACATACGAGGGCAATTCACCATTTCATCCTCTAGCTGATAACAAATT
tgc act gac ttg ctt tag cac tca att tgc ttt tgc ttg tcc tga cgg cgt aaa aca cgt cta tca
ACCTAAACTGTTCATCAGACAAGAGGAAGTTCAAGAACTTTACTCTCCAAT

27551 70 1 0 3 ORF7a-KFALTCFSTQFAFACPDGVKHVYQ53fs
TTCTGGAACATACGAGGGCAATTCACCATTTCATCCTCTAGCTGATAACA
aat ttg cac tga ctt gct tta gca ctc aat ttg ctt ttg ctt gtc ctg acg gc
GTTACGTGCCAGATCAGTTTCACCTAAACTGTTCATCAGACAAGAGGAAGT

CTCTTCTGGAACATACGAGGGCAATTCACCATTTCATCCTCTAGCTGATA
aca aat ttg cac tga ctt gct tta gca ctc aat ttg ctt ttg ctt gtc ctg acg gcg taa aac acg tct atc agt tac gtg
TCAGACAAGAGGAAGTTCAAGAACTTTACTCTCCAATTTTTCTTATTGTTG

27558 92 1 0 3 ORF7a-ALTCFSTQFAFACPDGVKHVYQLRARSVSPK55fs
ACATACGAGGGCAATTCACCATTTCATCCTCTAGCTGATAACAAATTTGC
act gac ttg ctt tag cac tca att tgc ttt tgc ttg tcc tga cgg cgt aaa aca cgt cta tca gtt acg
TGTTCATCAGACAAGAGGAAGTTCAAGAACTTTACTCTCCAATTTTTCTTA

TTCACCATTTCATCCTCTAGCTGATAACAAATTTGCACTGACTTGCTTTA
gca ctc aat ttg ctt ttg ctt gtc ctg acg gcg taa
AGTTACGTGCCAGATCAGTTTCACCTAAACTGTTCATCAGACAAGAGGAAG

27556 43 1 0 3 ORF7a-ALTCFSTQFAFACPD55fs
GAACATACGAGGGCAATTCACCATTTCATCCTCTAGCTGATAACAAATTT
gca ctg act tgc ttt agc act caa ttt gct ttt
ACGGCGTAAAACACGTCTATCAGTTACGTGCCAGATCAGTTTCACCTAAAC

27566 59 1 0 3 ORF7a-CFSTQFAFACPDGVKHVYQL58fs

GGGCAATTCACCATTTCATCCTCTAGCTGATAACAAATTTGCACTGACTT
gct tta gca ctc aat ttg ctt ttg ctt gtc ctg acg gcg taa aac
CGTGCCAGATCAGTTTCACCTAAACTGTTCATCAGACAAGAGGAAGTTCAA

27568 52 1 0 3 ORF7a-FSTQFAFACPDGVKHVYQ59fs
GCAATTCACCATTTCATCCTCTAGCTGATAACAAATTTGCACTGACTTGC
ttt agc act caa ttt gct ttt gct tgt cct gac ggc gta
AGTTACGTGCCAGATCAGTTTCACCTAAACTGTTCATCAGACAAGAGGAAG

27571 12 30 10 556 ORF7a-STQF60del
ATTCACCATTTCATCCTCTAGCTGATAACAAATTTGCACTGACTTGCTTT
agc act caa
GCTTTTGCTTGTCCTGACGGCGTAAAACACGTCTATCAGTTACGTGCCAGA

27555 69 14 1 61 ORF7a-FALTCFSTQFAFACPDGVKHVYQ54del
GGAACATACGAGGGCAATTCACCATTTCATCCTCTAGCTGATAACAAATT
tgc act gac ttg ctt tag cac tca att tgc ttt tgc ttg tcc tga cgg cgt a
ACGTGCCAGATCAGTTTCACCTAAACTGTTCATCAGACAAGAGGAAGTTCA

GGAACATACGAGGGCAATTCACCATTTCATCCTCTAGCTGATAACAAATT
tgc act gac ttg ctt tag cac tca att tgc ttt tgc ttg tcc tga cgg cgt aaa aca cgt cta tca gtt
AACTGTTCATCAGACAAGAGGAAGTTCAAGAACTTTACTCTCCAATTTTTC

ATTCACCATTTCATCCTCTAGCTGATAACAAATTTGCACTGACTTGCTTT
agc act caa ttt
TTGCTTGTCCTGACGGCGTAAAACACGTCTATCAGTTACGTGCCAGATCAG

ACCATTTCATCCTCTAGCTGATAACAAATTTGCACTGACTTGCTTTAGCA
ctc aat ttg ctt ttg ctt
GACGGCGTAAAACACGTCTATCAGTTACGTGCCAGATCAGTTTCACCTAAA

GGAACATACGAGGGCAATTCACCATTTCATCCTCTAGCTGATAACAAATT
tgc act gac ttg ctt tag cac tca att tgc ttt tgc ttg
AAACACGTCTATCAGTTACGTGCCAGATCAGTTTCACCTAAACTGTTCATC

27571 18 25 0 50 ORF7a-STQFAF60del
ATTCACCATTTCATCCTCTAGCTGATAACAAATTTGCACTGACTTGCTTT
agc act caa ttt gc
GCTTGTCCTGACGGCGTAAAACACGTCTATCAGTTACGTGCCAGATCAGTT

CACCATTTCATCCTCTAGCTGATAACAAATTTGCACTGACTTGCTTTAGC
act caa ttt gct t
TTGTCCTGACGGCGTAAAACACGTCTATCAGTTACGTGCCAGATCAGTTTC

TACGAGGGCAATTCACCATTTCATCCTCTAGCTGATAACAAATTTGCACT
gac ttg ctt tag
CAATTTGCTTTTGCTTGTCCTGACGGCGTAAAACACGTCTATCAGTTACGT

GGGCAATTCACCATTTCATCCTCTAGCTGATAACAAATTTGCACTGACTT
gct tta gca ctc aat ttg ctt
TCCTGACGGCGTAAAACACGTCTATCAGTTACGTGCCAGATCAGTTTCACC

TGGAACATACGAGGGCAATTCACCATTTCATCCTCTAGCTGATAACAAAT
ttg cac tga ctt gct tta gca ctc aat ttg ctt ttg ctt gtc ctg acg gcg taa aac acg tct atc agt

AAACTGTTCATCAGACAAGAGGAAGTTCAAGAACTTTACTCTCCAATTTTT

27555 51 1 0 39 ORF7a-FALTCFSTQFAFACPDGV54L
GGAACATACGAGGGCAATTCACCATTTCATCCTCTAGCTGATAACAAATT
tgc act gac ttg ctt tag cac tca att tgc ttt tgc ttg
AAAACACGTCTATCAGTTACGTGCCAGATCAGTTTCACCTAAACTGTTCAT

27555 24 21 0 36 ORF7a-FALTCFSTQ54L
GGAACATACGAGGGCAATTCACCATTTCATCCTCTAGCTGATAACAAATT
tgc act gac ttg ctt tag
ATTTGCTTTTGCTTGTCCTGACGGCGTAAAACACGTCTATCAGTTACGTGC

AATTCACCATTTCATCCTCTAGCTGATAACAAATTTGCACTGACTTGCTT
tag cac tca att tgc
GCTTGTCCTGACGGCGTAAAACACGTCTATCAGTTACGTGCCAGATCAGTT

27556 51 1 0 32 ORF7a-ALTCFSTQFAFACPDGV55del
GAACATACGAGGGCAATTCACCATTTCATCCTCTAGCTGATAACAAATTT
gca ctg act tgc ttt agc act caa ttt gct ttt gct tgt
AAACACGTCTATCAGTTACGTGCCAGATCAGTTTCACCTAAACTGTTCATC

27570 9 6 0 24 ORF7a-FSTQ59L
AATTCACCATTTCATCCTCTAGCTGATAACAAATTTGCACTGACTTGCTT
tag cac t
ATTTGCTTTTGCTTGTCCTGACGGCGTAAAACACGTCTATCAGTTACGTGC

GAACATACGAGGGCAATTCACCATTTCATCCTCTAGCTGATAACAAATTT
gca ctg act tgc ttt agc act caa ttt gct ttt gct tgt cct gac ggc gta aaa cac gtc tat c
CACCTAAACTGTTCATCAGACAAGAGGAAGTTCAAGAACTTTACTCTCCAA

TTCACCATTTCATCCTCTAGCTGATAACAAATTTGCACTGACTTGCTTTA
gca ctc aat ttg ctt ttg ctt gtc ctg acg gcg taa aac a
CGTGCCAGATCAGTTTCACCTAAACTGTTCATCAGACAAGAGGAAGTTCAA

GGAACATACGAGGGCAATTCACCATTTCATCCTCTAGCTGATAACAAATT
tgc act gac ttg ctt tag cac tca att tgc ttt tgc ttg tcc tga cgg cgt aaa aca cgt ct
GTTTCACCTAAACTGTTCATCAGACAAGAGGAAGTTCAAGAACTTTACTCT

TTCACCATTTCATCCTCTAGCTGATAACAAATTTGCACTGACTTGCTTTA
gca ctc aat ttg ctt ttg ctt gtc ctg acg gcg taa aac acg tct atc agt tac
CCTAAACTGTTCATCAGACAAGAGGAAGTTCAAGAACTTTACTCTCCAATT

27558 27 6 0 11 ORF7a-LTCFSTQFA56del
ACATACGAGGGCAATTCACCATTTCATCCTCTAGCTGATAACAAATTTGC
act gac ttg ctt tag cac tca
TTTTGCTTGTCCTGACGGCGTAAAACACGTCTATCAGTTACGTGCCAGATC

TTCACCATTTCATCCTCTAGCTGATAACAAATTTGCACTGACTTGCTTTA
gca ctc
ATTTGCTTTTGCTTGTCCTGACGGCGTAAAACACGTCTATCAGTTACGTGC

AATTCACCATTTCATCCTCTAGCTGATAACAAATTTGCACTGACTTGCTT
tag cac tca att tgc ttt tgc ttg tcc tga cgg cgt aaa aca
CGTGCCAGATCAGTTTCACCTAAACTGTTCATCAGACAAGAGGAAGTTCAA

27556 63 2 0 10 ORF7a-ALTCFSTQFAFACPDGVKHVY55del
GAACATACGAGGGCAATTCACCATTTCATCCTCTAGCTGATAACAAATTT
gca ctg act tgc ttt agc act caa ttt gct ttt gct tgt cct gac ggc
CAGTTACGTGCCAGATCAGTTTCACCTAAACTGTTCATCAGACAAGAGGAA

GGGCAATTCACCATTTCATCCTCTAGCTGATAACAAATTTGCACTGACTT
gct tta gca c
ATTTGCTTTTGCTTGTCCTGACGGCGTAAAACACGTCTATCAGTTACGTGC

27560 84 5 0 9 ORF7a-LTCFSTQFAFACPDGVKHVYQLRARSVS56del
ATACGAGGGCAATTCACCATTTCATCCTCTAGCTGATAACAAATTTGCAC
tga ctt gct tta gca ctc aat ttg ctt ttg ctt gtc ctg acg gcg taa aac acg tct atc agt
CTAAACTGTTCATCAGACAAGAGGAAGTTCAAGAACTTTACTCTCCAATTT

TTCACCATTTCATCCTCTAGCTGATAACAAATTTGCACTGACTTGCTTTA
gca ctc
TTTGCTTTTGCTTGTCCTGACGGCGTAAAACACGTCTATCAGTTACGTGCC

GGGCAATTCACCATTTCATCCTCTAGCTGATAACAAATTTGCACTGACTT
gct tta gca ctc aat ttg ctt ttg ctt gtc ctg acg gcg taa aac acg t
AGATCAGTTTCACCTAAACTGTTCATCAGACAAGAGGAAGTTCAAGAACTT

ATTCACCATTTCATCCTCTAGCTGATAACAAATTTGCACTGACTTGCTTT
agc act caa
TGCTTTTGCTTGTCCTGACGGCGTAAAACACGTCTATCAGTTACGTGCCAG

27567 27 6 0 7 ORF7a-FSTQFAFAC59del
GGCAATTCACCATTTCATCCTCTAGCTGATAACAAATTTGCACTGACTTG
ctt tag cac tca att tgc ttt
TCCTGACGGCGTAAAACACGTCTATCAGTTACGTGCCAGATCAGTTTCACC

27573 60 4 0 7 ORF7a-STQFAFACPDGVKHVYQLRA60del
TCACCATTTCATCCTCTAGCTGATAACAAATTTGCACTGACTTGCTTTAG
cac tca att tgc ttt tgc ttg tcc tga cgg cgt aaa aca cgt cta
ATCAGTTTCACCTAAACTGTTCATCAGACAAGAGGAAGTTCAAGAACTTTA

27574 30 1 0 7 ORF7a-TQFAFACPDG61del
CACCATTTCATCCTCTAGCTGATAACAAATTTGCACTGACTTGCTTTAGC
act caa ttt gct ttt gct tgt cc
GTAAAACACGTCTATCAGTTACGTGCCAGATCAGTTTCACCTAAACTGTTC

TCACCATTTCATCCTCTAGCTGATAACAAATTTGCACTGACTTGCTTTAG
cac tca att tgc ttt tgc ttg tcc tga cgg cgt aaa a
TTACGTGCCAGATCAGTTTCACCTAAACTGTTCATCAGACAAGAGGAAGTT

27556 84 2 0 6 ORF7a-ALTCFSTQFAFACPDGVKHVYQLRARSV55del
GAACATACGAGGGCAATTCACCATTTCATCCTCTAGCTGATAACAAATTT
gca ctg act tgc ttt agc act caa ttt gct ttt gct tgt cct gac ggc gta aaa cac gtc tat
TCACCTAAACTGTTCATCAGACAAGAGGAAGTTCAAGAACTTTACTCTCCA

AATTCACCATTTCATCCTCTAGCTGATAACAAATTTGCACTGACTTGCTT
tag cac tca att tgc ttt tgc ttg tcc tga cgg cgt aaa aca cgt cta tca gtt
CACCTAAACTGTTCATCAGACAAGAGGAAGTTCAAGAACTTTACTCTCCAA

TTCACCATTTCATCCTCTAGCTGATAACAAATTTGCACTGACTTGCTTTA
gca ctc aa
TGCTTTTGCTTGTCCTGACGGCGTAAAACACGTCTATCAGTTACGTGCCAG

TCTTCTGGAACATACGAGGGCAATTCACCATTTCATCCTCTAGCTGATAA
caa att tgc act gac ttg ctt tag
TTTGCTTTTGCTTGTCCTGACGGCGTAAAACACGTCTATCAGTTACGTGCC

GAACATACGAGGGCAATTCACCATTTCATCCTCTAGCTGATAACAAATTT
gca ctg act tgc ttt agc act caa ttt gct ttt gct tgt cct gac ggc
AGTTACGTGCCAGATCAGTTTCACCTAAACTGTTCATCAGACAAGAGGAAG

GGGCAATTCACCATTTCATCCTCTAGCTGATAACAAATTTGCACTGACTT
gct tta gca ctc aat ttg ctt ttg ctt gtc ctg acg gcg taa aac acg
CAGATCAGTTTCACCTAAACTGTTCATCAGACAAGAGGAAGTTCAAGAACT

ATTCACCATTTCATCCTCTAGCTGATAACAAATTTGCACTGACTTGCTTT
agc act ca
TTGCTTTTGCTTGTCCTGACGGCGTAAAACACGTCTATCAGTTACGTGCCA

GAACATACGAGGGCAATTCACCATTTCATCCTCTAGCTGATAACAAATTT
gca ctg act tgc ttt agc
ATTTGCTTTTGCTTGTCCTGACGGCGTAAAACACGTCTATCAGTTACGTGC

GAACATACGAGGGCAATTCACCATTTCATCCTCTAGCTGATAACAAATTT
gca ctg act tgc ttt agc act caa
TGCTTGTCCTGACGGCGTAAAACACGTCTATCAGTTACGTGCCAGATCAGT

GGAACATACGAGGGCAATTCACCATTTCATCCTCTAGCTGATAACAAATT
tgc act gac ttg ctt tag cac tca att tgc ttt tgc ttg tcc tga cgg cgt aa
CGTGCCAGATCAGTTTCACCTAAACTGTTCATCAGACAAGAGGAAGTTCAA

27574 9 1 0 4 ORF7a-TQF61del
CACCATTTCATCCTCTAGCTGATAACAAATTTGCACTGACTTGCTTTAGC
act caa t
GCTTTTGCTTGTCCTGACGGCGTAAAACACGTCTATCAGTTACGTGCCAGA

CATACGAGGGCAATTCACCATTTCATCCTCTAGCTGATAACAAATTTGCA
ctg act tgc ttt agc act caa ttt gct ttt gct tgt cct gac ggc gta aaa cac gtc tat cag tta
AACTGTTCATCAGACAAGAGGAAGTTCAAGAACTTTACTCTCCAATTTTTC

GGGCAATTCACCATTTCATCCTCTAGCTGATAACAAATTTGCACTGACTT
gct tta gca ctc
TGCTTTTGCTTGTCCTGACGGCGTAAAACACGTCTATCAGTTACGTGCCAG

CTGGAACATACGAGGGCAATTCACCATTTCATCCTCTAGCTGATAACAAA
ttt gca ctg act tgc ttt agc act caa ttt gct ttt gct tgt
ACACGTCTATCAGTTACGTGCCAGATCAGTTTCACCTAAACTGTTCATCAG

27572 15 3 0 3 ORF7a-STQFAF60I
TTCACCATTTCATCCTCTAGCTGATAACAAATTTGCACTGACTTGCTTTA
gca ctc aat ttg
TTGCTTGTCCTGACGGCGTAAAACACGTCTATCAGTTACGTGCCAGATCAG

27565 66 3 0 3 ORF7a-CFSTQFAFACPDGVKHVYQLRA58del
AGGGCAATTCACCATTTCATCCTCTAGCTGATAACAAATTTGCACTGACT
tgc ttt agc act caa ttt gct ttt gct tgt cct gac ggc gta aaa cac gt
AGATCAGTTTCACCTAAACTGTTCATCAGACAAGAGGAAGTTCAAGAACTT

27553 96 3 0 3 ORF7a-FALTCFSTQFAFACPDGVKHVYQLRARSVSPK54del
CTGGAACATACGAGGGCAATTCACCATTTCATCCTCTAGCTGATAACAAA
ttt gca ctg act tgc ttt agc act caa ttt gct ttt gct tgt cct gac ggc gta aaa cac gtc tat cag tta
CTGTTCATCAGACAAGAGGAAGTTCAAGAACTTTACTCTCCAATTTTTCTT

CTCTTCTGGAACATACGAGGGCAATTCACCATTTCATCCTCTAGCTGATA
aca aat
GCACTGACTTGCTTTAGCACTCAATTTGCTTTTGCTTGTCCTGACGGCGTA

GGGCAATTCACCATTTCATCCTCTAGCTGATAACAAATTTGCACTGACTT
gct tta
ACTCAATTTGCTTTTGCTTGTCCTGACGGCGTAAAACACGTCTATCAGTTA

27566 51 2 0 3 ORF7a-CFSTQFAFACPDGVKHV58del
GGGCAATTCACCATTTCATCCTCTAGCTGATAACAAATTTGCACTGACTT
gct tta gca ctc aat ttg ctt ttg ctt gtc ctg acg gcg
ATCAGTTACGTGCCAGATCAGTTTCACCTAAACTGTTCATCAGACAAGAGG

CCATTTCATCCTCTAGCTGATAACAAATTTGCACTGACTTGCTTTAGCAC
tca att
GCTTTTGCTTGTCCTGACGGCGTAAAACACGTCTATCAGTTACGTGCCAGA

CCATTTCATCCTCTAGCTGATAACAAATTTGCACTGACTTGCTTTAGCAC
tca att tgc ttt tgc ttg tcc tga cgg cgt aaa aca cg
GTGCCAGATCAGTTTCACCTAAACTGTTCATCAGACAAGAGGAAGTTCAAG

GGAACATACGAGGGCAATTCACCATTTCATCCTCTAGCTGATAACAAATT
tgc act gac ttg ctt tag cac tca att tgc ttt tgc ttg tcc tga cgg cgt aaa aca cgt cta tc
CACCTAAACTGTTCATCAGACAAGAGGAAGTTCAAGAACTTTACTCTCCAA

27555 87 2 0 3 ORF7a-FALTCFSTQFAFACPDGVKHVYQLRARSVS54L
GGAACATACGAGGGCAATTCACCATTTCATCCTCTAGCTGATAACAAATT
tgc act gac ttg ctt tag cac tca att tgc ttt tgc ttg tcc tga cgg cgt aaa aca cgt cta tca
ACCTAAACTGTTCATCAGACAAGAGGAAGTTCAAGAACTTTACTCTCCAAT

27548 108 1 0 3 ORF7a-NKFALTCFSTQFAFACPDGVKHVYQLRARSVSPKLF52del
CTCTTCTGGAACATACGAGGGCAATTCACCATTTCATCCTCTAGCTGATA
aca aat ttg cac tga ctt gct tta gca ctc aat ttg ctt ttg ctt gtc ctg acg gcg taa aac acg tct atc agt tac gtg
TCAGACAAGAGGAAGTTCAAGAACTTTACTCTCCAATTTTTCTTATTGTTG

27542 75 1 0 3 ORF7a-ADNKFALTCFSTQFAFACPDGVKHVY50D
ACCTTGCTCTTCTGGAACATACGAGGGCAATTCACCATTTCATCCTCTAG
ctg ata aca aat ttg cac tga ctt gct tta gca ctc aat ttg ctt ttg ctt gtc ctg
ATCAGTTACGTGCCAGATCAGTTTCACCTAAACTGTTCATCAGACAAGAGG

27572 48 1 0 3 ORF7a-STQFAFACPDGVKHVYQ60K
TTCACCATTTCATCCTCTAGCTGATAACAAATTTGCACTGACTTGCTTTA
gca ctc aat ttg ctt ttg ctt gtc ctg acg gcg taa

AGTTACGTGCCAGATCAGTTTCACCTAAACTGTTCATCAGACAAGAGGAAG

GAACATACGAGGGCAATTCACCATTTCATCCTCTAGCTGATAACAAATTT
gca ctg act tgc ttt agc act caa ttt gct ttt
ACGGCGTAAAACACGTCTATCAGTTACGTGCCAGATCAGTTTCACCTAAAC

GGGCAATTCACCATTTCATCCTCTAGCTGATAACAAATTTGCACTGACTT
gct tta gca ctc aat ttg ctt ttg ctt gtc ctg acg gcg taa aac
CGTGCCAGATCAGTTTCACCTAAACTGTTCATCAGACAAGAGGAAGTTCAA
