# Supplementary material for: Deletions across the SARS-CoV-2 Genome: Molecular Mechanisms and Putative Functional Consequences of Deletions in Accessory Genes
Source: Microorganisms. 2023 Jan 16;11(1):229. doi: 10.3390/microorganisms11010229 (PMC9862619; doi:10.3390/microorganisms11010229)
Supplement: Supplementary file 1 [file microorganisms-11-00229-s001.zip › Figure S4.pdf]

Supplementary Figure S4. Contexts of short deletions in the bin #10 of ORF8.
The 1st number is the postion and the 5th number is the number of instances followed by gene names.

28223 3 1 0 3 ORF8-EY110D
AAATTGGGTAGTCTTGTAGTGCGTTGTTCGTTCTATGAAGACTTTTTAGA
gta
TCATGACGTTCGTGTTGTTTTAGATTTCATCTAAACGAACAAACTAAAATG

28228 1 1 0 3 ORF8-H112fs
GGGTAGTCTTGTAGTGCGTTGTTCGTTCTATGAAGACTTTTTAGAGTATC
a
TGACGTTCGTGTTGTTTTAGATTTCATCTAAACGAACAAACTAAAATGTCT

28231 3 1 0 3 ORF8-D113
TAGTCTTGTAGTGCGTTGTTCGTTCTATGAAGACTTTTTAGAGTATCATG
acg
TTCGTGTTGTTTTAGATTTCATCTAAACGAACAAACTAAAATGTCTGATAA

28236 6 2 0 3 ORF8-RV115
TTGTAGTGCGTTGTTCGTTCTATGAAGACTTTTTAGAGTATCATGACGTT
cgt gtt
GTTTTAGATTTCATCTAAACGAACAAACTAAAATGTCTGATAATGGACCCC

28240 2 4 0 115 ORF8-V116fs
AGTGCGTTGTTCGTTCTATGAAGACTTTTTAGAGTATCATGACGTTCGTG
tt
GTTTTAGATTTCATCTAAACGAACAAACTAAAATGTCTGATAATGGACCCC

28241 6 2 0 3 ORF8-VL117
GTGCGTTGTTCGTTCTATGAAGACTTTTTAGAGTATCATGACGTTCGTGT
tgt ttt
AGATTTCATCTAAACGAACAAACTAAAATGTCTGATAATGGACCCCAAAAT

28242 3 29 0 56 ORF8-V117
TGCGTTGTTCGTTCTATGAAGACTTTTTAGAGTATCATGACGTTCGTGTT
gtt
TTAGATTTCATCTAAACGAACAAACTAAAATGTCTGATAATGGACCCCAAA

28242 4 20 0 47 ORF8-VL117fs
TGCGTTGTTCGTTCTATGAAGACTTTTTAGAGTATCATGACGTTCGTGTT
gttt
AGATTTCATCTAAACGAACAAACTAAAATGTCTGATAATGGACCCCAAAA

28242 2 3 0 40 ORF8-V117fs
TGCGTTGTTCGTTCTATGAAGACTTTTTAGAGTATCATGACGTTCGTGTT
gt
TTTAGATTTCATCTAAACGAACAAACTAAAATGTCTGATAATGGACCCCAA

28242 5 14 0 32 ORF8-VL117fs
TGCGTTGTTCGTTCTATGAAGACTTTTTAGAGTATCATGACGTTCGTGTT
gtt tt

AGATTTCATCTAAACGAACAAACTAAAATGTCTGATAATGGACCCCAAAAT

28242 1 4 0 4 ORF8-V117fs
TGCGTTGTTCGTTCTATGAAGACTTTTTAGAGTATCATGACGTTCGTGTT
g
TTTTAGATTTCATCTAAACGAACAAACTAAAATGTCTGATAATGGACCCCA

28243 6 4 0 8 ORF8-VL117
GCGTTGTTCGTTCTATGAAGACTTTTTAGAGTATCATGACGTTCGTGTTG
ttt tag
ATTTCATCTAAACGAACAAACTAAAATGTCTGATAATGGACCCCAAAATCA

28244 3 5 0 6 ORF8-L118
CGTTGTTCGTTCTATGAAGACTTTTTAGAGTATCATGACGTTCGTGTTGT
ttt
AGATTTCATCTAAACGAACAAACTAAAATGTCTGATAATGGACCCCAAAAT

28245 3 3 0 8 ORF8-L118
GTTGTTCGTTCTATGAAGACTTTTTAGAGTATCATGACGTTCGTGTTGTT
tta
GATTTCATCTAAACGAACAAACTAAAATGTCTGATAATGGACCCCAAAATC

28246 2 1 0 4 ORF8-L118fs
TTGTTCGTTCTATGAAGACTTTTTAGAGTATCATGACGTTCGTGTTGTTT
ta
GATTTCATCTAAACGAACAAACTAAAATGTCTGATAATGGACCCCAAAATC

28247 1 5 0 104 ORF8-L118fs
TGTTCGTTCTATGAAGACTTTTTAGAGTATCATGACGTTCGTGTTGTTTT
a
GATTTCATCTAAACGAACAAACTAAAATGTCTGATAATGGACCCCAAAATC

28247 5 7 0 15 ORF8-LD118fs
TGTTCGTTCTATGAAGACTTTTTAGAGTATCATGACGTTCGTGTTGTTTT
aga tt
TCATCTAAACGAACAAACTAAAATGTCTGATAATGGACCCCAAAATCAGCG

28247 6 2 0 8 ORF8-LD118
TGTTCGTTCTATGAAGACTTTTTAGAGTATCATGACGTTCGTGTTGTTTT
aga ttt
CATCTAAACGAACAAACTAAAATGTCTGATAATGGACCCCAAAATCAGCGA

28248 6 25 83 52612 ORF8-DF119
GTTCGTTCTATGAAGACTTTTTAGAGTATCATGACGTTCGTGTTGTTTTA
gat ttc
ATCTAAACGAACAAACTAAAATGTCTGATAATGGACCCCAAAATCAGCGAA

28248 4 11 1 52 ORF8-DF119fs
GTTCGTTCTATGAAGACTTTTTAGAGTATCATGACGTTCGTGTTGTTTTA
gat t
TCATCTAAACGAACAAACTAAAATGTCTGATAATGGACCCCAAAATCAGCG

28248 1 14 0 22 ORF8-D119fs
GTTCGTTCTATGAAGACTTTTTAGAGTATCATGACGTTCGTGTTGTTTTA

g
ATTTCATCTAAACGAACAAACTAAAATGTCTGATAATGGACCCCAAAATCA

28248 5 3 0 7 ORF8-DF119fs
GTTCGTTCTATGAAGACTTTTTAGAGTATCATGACGTTCGTGTTGTTTTA
gat tt
CATCTAAACGAACAAACTAAAATGTCTGATAATGGACCCCAAAATCAGCGA

28248 2 2 0 3 ORF8-D119fs
GTTCGTTCTATGAAGACTTTTTAGAGTATCATGACGTTCGTGTTGTTTTA
ga
TTTCATCTAAACGAACAAACTAAAATGTCTGATAATGGACCCCAAAATCAG

28249 2 4 0 100 ORF8-D119fs
TTCGTTCTATGAAGACTTTTTAGAGTATCATGACGTTCGTGTTGTTTTAG
at
TTCATCTAAACGAACAAACTAAAATGTCTGATAATGGACCCCAAAATCAGC

28250 3 2 0 8 ORF8-F120
TCGTTCTATGAAGACTTTTTAGAGTATCATGACGTTCGTGTTGTTTTAGA
ttt
CATCTAAACGAACAAACTAAAATGTCTGATAATGGACCCCAAAATCAGCGA

28252 2 11 0 17 ORF8-F120fs
GTTCTATGAAGACTTTTTAGAGTATCATGACGTTCGTGTTGTTTTAGATT
tc
ATCTAAACGAACAAACTAAAATGTCTGATAATGGACCCCAAAATCAGCGAA

28252 1 4 0 4 ORF8-F120fs
GTTCTATGAAGACTTTTTAGAGTATCATGACGTTCGTGTTGTTTTAGATT
t
CATCTAAACGAACAAACTAAAATGTCTGATAATGGACCCCAAAATCAGCGA

28253 2 3 0 4 ORF8-F120fs
TTCTATGAAGACTTTTTAGAGTATCATGACGTTCGTGTTGTTTTAGATTT
ca
TCTAAACGAACAAACTAAAATGTCTGATAATGGACCCCAAAATCAGCGAAA

28253 5 6 0 7 ORF8-FI120fs
TTCTATGAAGACTTTTTAGAGTATCATGACGTTCGTGTTGTTTTAGATTT
cat ct
AAACGAACAAACTAAAATGTCTGATAATGGACCCCAAAATCAGCGAAATGC

28253 1 2 0 5 ORF8-F120fs
TTCTATGAAGACTTTTTAGAGTATCATGACGTTCGTGTTGTTTTAGATTT
c
ATCTAAACGAACAAACTAAAATGTCTGATAATGGACCCCAAAATCAGCGAA

28254 1 534 23 3208 ORF8-I121fs
TCTATGAAGACTTTTTAGAGTATCATGACGTTCGTGTTGTTTTAGATTTC
a
TCTAAACGAACAAACTAAAATGTCTGATAATGGACCCCAAAATCAGCGAAA

28254 3 5 0 43 ORF8-I121

TCTATGAAGACTTTTTAGAGTATCATGACGTTCGTGTTGTTTTAGATTTC
atc
TAAACGAACAAACTAAAATGTCTGATAATGGACCCCAAAATCAGCGAAATG

28254 2 3 0 9 ORF8-I121fs
TCTATGAAGACTTTTTAGAGTATCATGACGTTCGTGTTGTTTTAGATTTC
at
CTAAACGAACAAACTAAAATGTCTGATAATGGACCCCAAAATCAGCGAAAT

28256 1 5 0 8 ORF8-I121fs
TATGAAGACTTTTTAGAGTATCATGACGTTCGTGTTGTTTTAGATTTCAT
c
TAAACGAACAAACTAAAATGTCTGATAATGGACCCCAAAATCAGCGAAATG

28257 1 2 0 3 ORF8-*122fs
ATGAAGACTTTTTAGAGTATCATGACGTTCGTGTTGTTTTAGATTTCATC
t
AAACGAACAAACTAAAATGTCTGATAATGGACCCCAAAATCAGCGAAATGC
