# Supplementary material for: Deletions across the SARS-CoV-2 Genome: Molecular Mechanisms and Putative Functional Consequences of Deletions in Accessory Genes
Source: Microorganisms. 2023 Jan 16;11(1):229. doi: 10.3390/microorganisms11010229 (PMC9862619; doi:10.3390/microorganisms11010229)
Supplement: Supplementary file 1 [file microorganisms-11-00229-s001.zip › Figure S6.pdf]

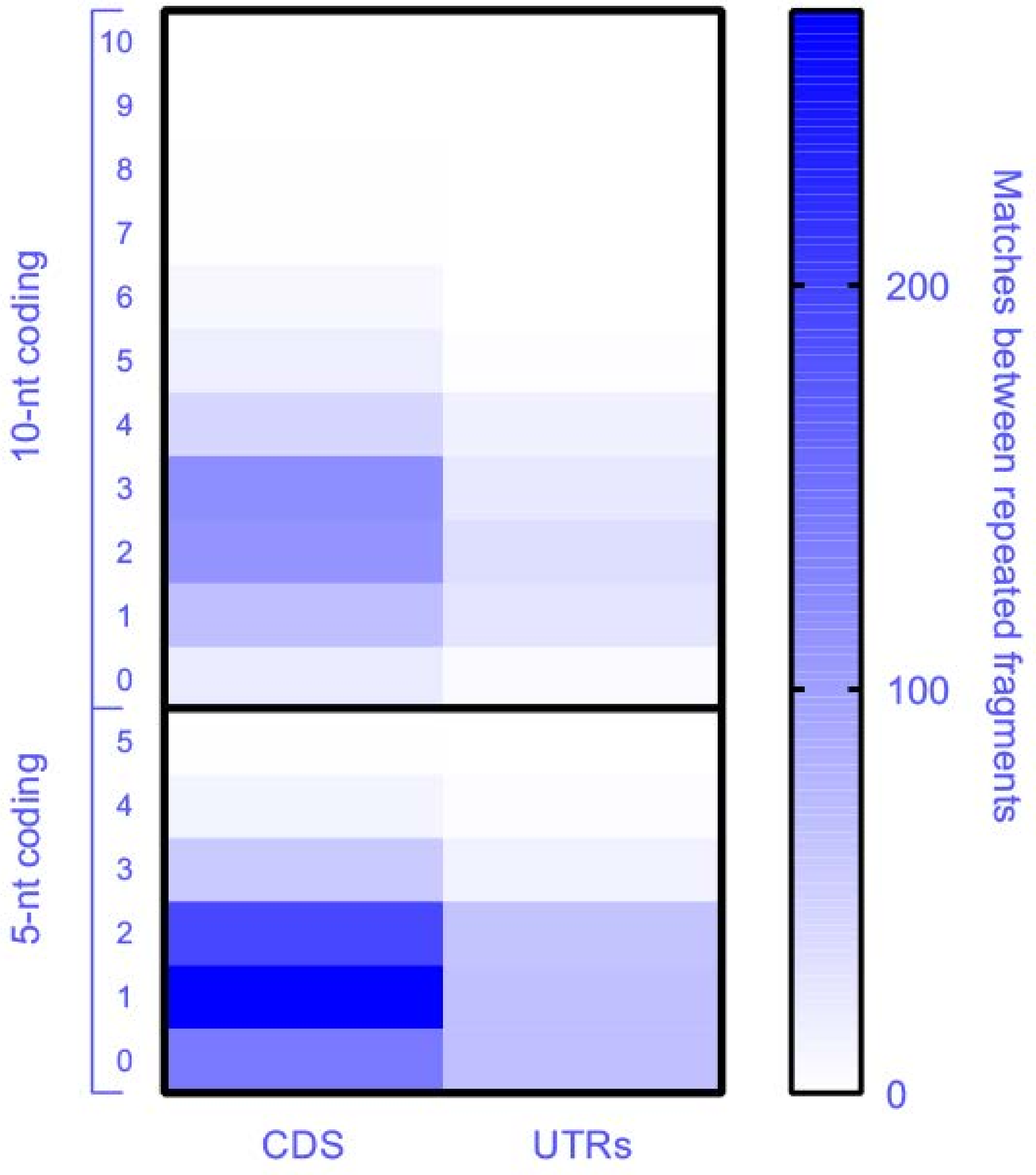

Figure S6. Heatmap of the association of long deletions with inverted repeats in UTR and CDS, considering Windows = 5 and 10 nucleotides, expressed as number of matches between repeated fragments.
