# Supplementary material for: Deletions across the SARS-CoV-2 Genome: Molecular Mechanisms and Putative Functional Consequences of Deletions in Accessory Genes
Source: Microorganisms. 2023 Jan 16;11(1):229. doi: 10.3390/microorganisms11010229 (PMC9862619; doi:10.3390/microorganisms11010229)
Supplement: Supplementary file 1 [file microorganisms-11-00229-s001.zip › Figure S7.pdf]

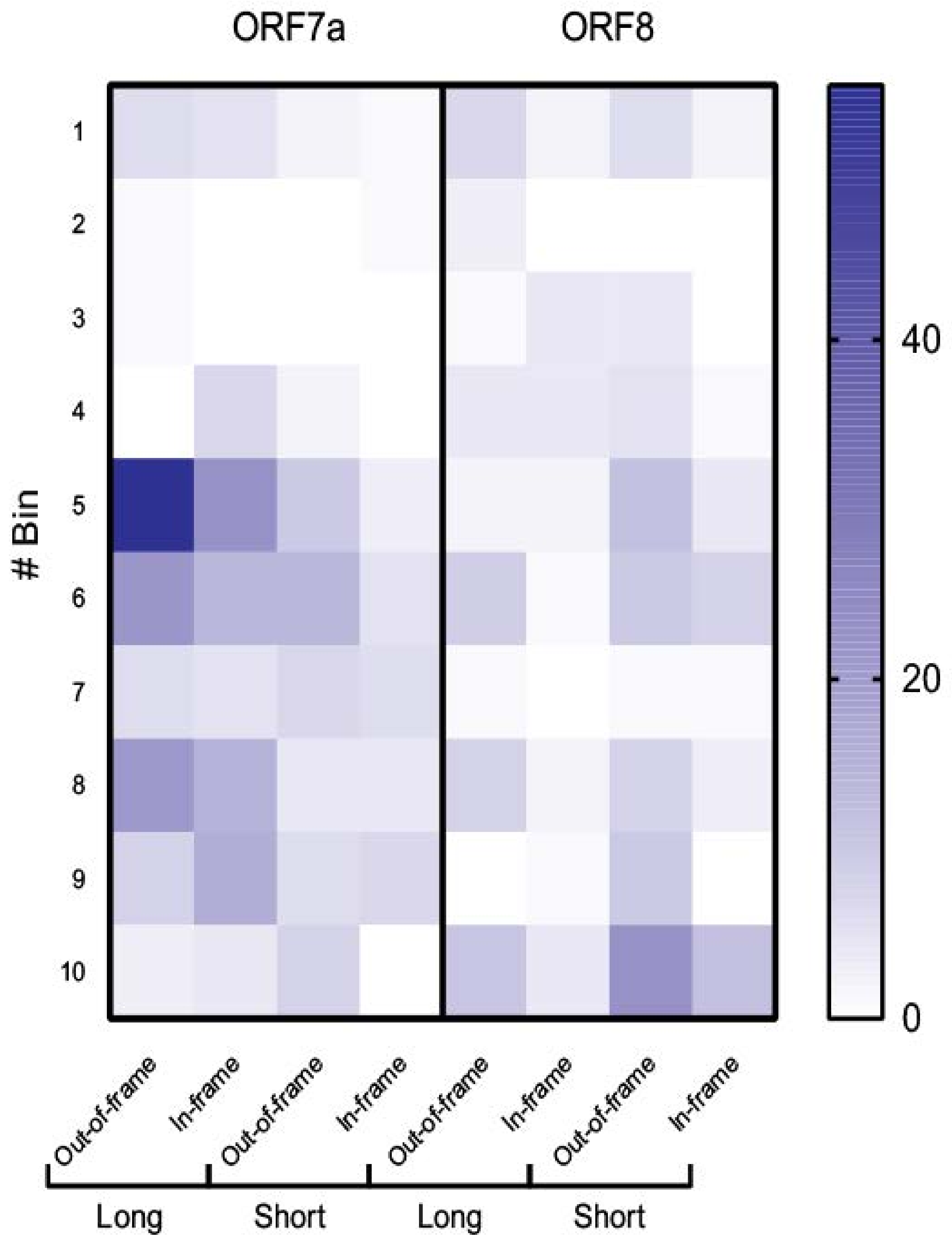

Figure S7. Heatmap of the distribution of in- and out-of-frame deletions in UTR and CDS for each bin (1 to 10), according to their length (Short and long).
